# Structure-Based Approach for the Prediction of Mu-opioid Binding Affinity of Unclassified Designer Fentanyl-Like Molecules

**DOI:** 10.3390/ijms20092311

**Published:** 2019-05-10

**Authors:** Giuseppe Floresta, Antonio Rescifina, Vincenzo Abbate

**Affiliations:** 1 Department of Drug Sciences, University of Catania, V.le A. Doria, 95125 Catania, Italy; arescifina@unict.it; 2 King’s Forensics, School of Population Health & Environmental Sciences, King’s College London, Franklin-Wilkins Building, 150 Stamford Street, London SE1 9NH, UK

**Keywords:** QSAR, fentanyl, μOR, opioid binding affinity, designer fentanyl-like molecules, novel synthetic opioids, new psychoactive substances

## Abstract

Three quantitative structure-activity relationship (QSAR) models for predicting the affinity of mu-opioid receptor (μOR) ligands have been developed. The resulted models, exploiting the accessibility of the QSAR modeling, generate a useful tool for the investigation and identification of unclassified fentanyl-like structures. The models have been built using a set of 115 molecules using Forge as a software, and the quality was confirmed by statistical analysis, resulting in being effective for their predictive and descriptive capabilities. The three different approaches were then combined to produce a consensus model and were exploited to explore the chemical landscape of 3000 fentanyl-like structures, generated by a theoretical scaffold-hopping approach. The findings of this study should facilitate the identification and classification of new μOR ligands with fentanyl-like structures.

## 1. Introduction

**Figure 1:**
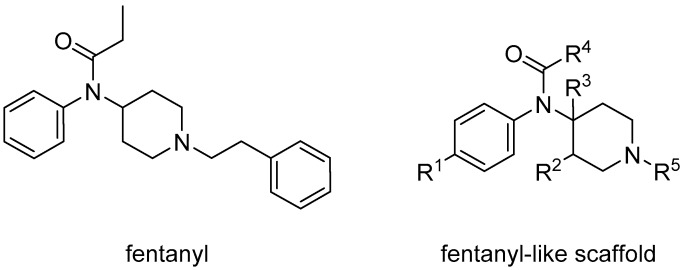
Structure of fentanyl and fentanyl-like compounds.

Opioid receptors are the target proteins of narcotic analgesics, of which morphine is the prototype, and their activation can produce a variety of pharmacological responses [1] that are used for the treatment of different medical conditions [2,3,4]. Their pharmacological action had been known long before the morphine itself was discovered, and, in early civilizations, extracts from the *Papaver somniferum* were widely used as a medicine [5]. Unfortunately, even at that period, the poppy plant was used as a recreational agent, and this social plague is still present all over the world representing a severe problem to societies [5]. The agents most commonly responsible for the increase in the number of drug overdose-related deaths are synthetic opioids and heroin [6]. In fact, opioid-related overdose deaths due to synthetic opioids are rapidly increasing, augmenting an already established plague to society [7,8]. The low cost, affordable synthetic procedures, and the high potency have led to the influx of fentanyl analogs into the street-drug market as indicated by the Drug Enforcement Administration (DEA) [9]. A significant additional risk to public health is the variability in potency among fentanyl analogs; indeed, certain derivatives (e.g., carfentanyl) are 10,000 times more potent than morphine [10], resulting in a potentially fatal dose for drug-addicted persons. Fentanyl is a potent agonist of the μOR, causing the classical analgesic and euphoric pharmacological effects of this class of compounds. Unfortunately, simple modifications of the central core of the molecule (4-anilidopiperidine, Figure 1) may produce ligands with increased potency, resulting in a high risk for the user. Importantly, many structural modifications to the original fentanyl chemical scaffold do not alter the primary function and binding properties to the μOR, resulting in a very large chemical space of fentanyl analogs with abuse potential.

In the USA, the DEA can schedule a substance to a legislative state regarding its physiological abuse potential; however, a complete analysis to support scheduling can take up to two years. For this reason, the Center for Drug Evaluation and Research has recently developed a docking-based virtual screening approach for the identification and hazard characterization of unclassified fentanyl-like structures [11]. Surprisingly, a ligand-based method for the classification of designer fentanyl-like structures has never been evaluated.

Quantitative structure-activity relationship (QSAR) models models are frequently used to facilitate the prediction and comprehension of patterns in the chemical and biological sciences [12,13,14,15,16,17,18]. In order to facilitate the investigation of unclassified fentanyl-like structures, exploiting QSAR methodology, we report the development of three different QSAR models established using a set of 115 fentanyl-like structures. The generated models, employed to evaluate a set of new potentially μOR binders, have been suggested employing the activity cliff analysis followed by a scaffold hopping study.

## 2. Results and Discussion

### 2.1. Statistical Analysis and Results

**Figure 2:**
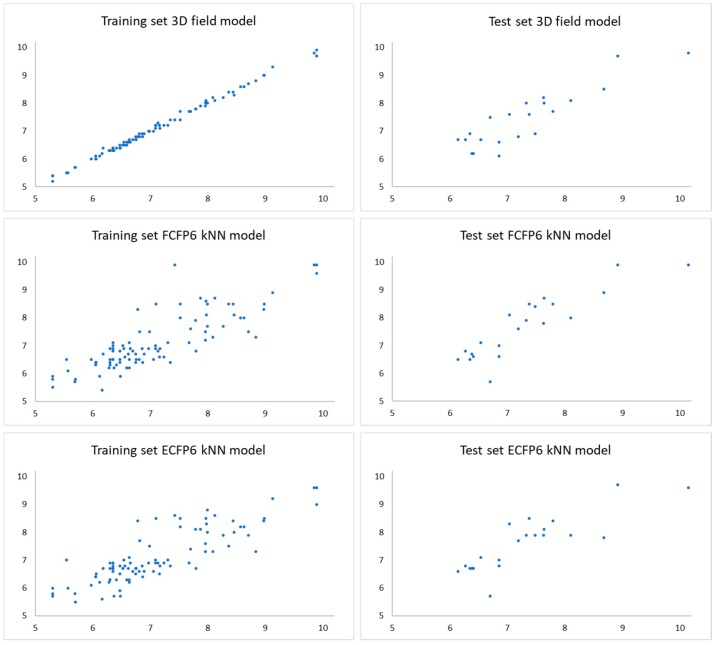
Experimental vs. predicted p*K*_i_ of the compounds in the training and test set for the different QSAR models.

For the calculation of the different models, Forge uses the SIMPLS algorithm [19,20]. All the experimental vs. predicted activities for the training and the test sets of the different models are presented in Figure 2. The optimal number of components in partial least squares (PLS) analysis was 10-, 2- and 3-component for the field-based 3D-model, and the FCFP6 and ECFP6 circular fingerprint descriptor 2D-models, respectively (Figures S1–S6). All of the generated models showed both good predictive and descriptive capabilities, demonstrated by the high r^2^ and q^2^ values for both the training and the cross-validated training sets (Table 1). The plots of experimental vs. predicted affinities for the molecules included in the test set (Figure 2) show a reasonable prediction demonstrated by the cross-validated r^2^ (Table 1). Among the three different models, the presence of the 3D-descriptors included in the 3D-field model clearly increased the quality of the description, as demonstrated by the high value of r^2^ (0.99) for the training set. Furthermore, the predictive capabilities resulted from such model are the best across the three models (r^2^ for the test set = 0.77, Tables S1 and S2). The results of the r^2^ test set >q^2^ have been referred to as the Kubinyi paradox and derive from the cross-validation method (leave-one-out) [21]. The reliability of the models was also evaluated by the measurement of the forecast errors. The mean squared error (MSE), the mean absolute error (MAE) and the mean absolute percentage error (MAPE) for the three models are reported in Table 1, all of them confirmed the statistical reliability of the obtained models.

**Figure 3:**
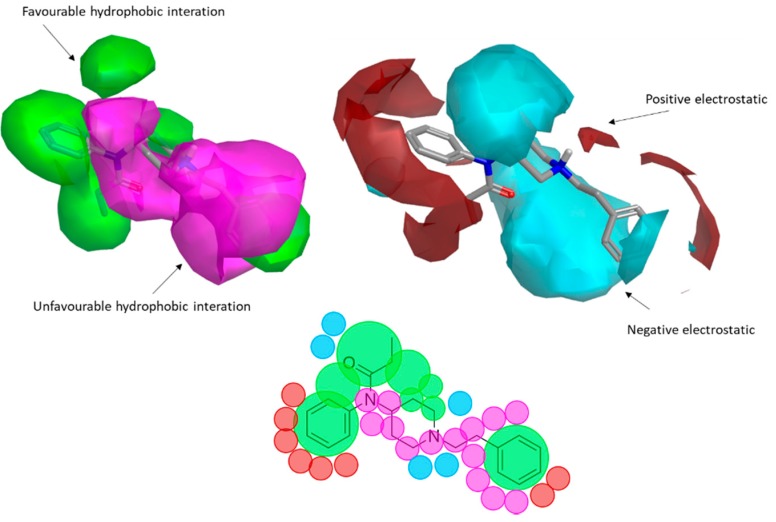
The AA model map is superimposed to fentanyl. Molecular insight of structure-activity relationship (SAR) mechanism models, revealing the different lead optimization sites of active compounds. Red color shows positive field region controlling the activity, and blue color the negative ones. Green color shows favorable shape/hydrophobic regions, and violet color the unfavorable ones.

The 3D visualizations, employing activity-atlas (AA), of the QSAR models superposed with the structure of fentanyl are shown in Figure 3. The conclusion of this visualization highlight that the 3D-field model is described by steric and electrostatic effects. The four colors on the 3D-map represent the different electrostatic (red and blue), and hydrophobic and shape features (green and violet). In the green areas, a bulk/hydrophobic interaction improves the binding affinity; opposite results are observed within the violet areas, where a bulk/hydrophobic interaction decreases the affinity. In the red areas, a more positive electrostatic field increases the receptor-affinity, whereas in the blue regions a more negative electrostatic field increases the affinity. From this representation, it appears that the bulk/hydrophobic interactions have a more relevant impact on the activity of the molecules; indeed, a green area is shown near to the R^2^ group of the fentanyl (Figure 1). This area is the hydrophobic pocket formed by Ile 144, Leu 200 and Tyr 148 of the µOR. Potent opioid analogs can be derived by simple substitution around this chemical space [22]. Moreover, another green area is located where R^3^-substituents are usually accommodated, further indicating that the presence of a fragment moiety in this area will produce potent fentanyl-like analogs. Two additional green areas are located near the two aromatic rings. The aromatic ring linked to the R^5^ is located in an area near to Tyr 326, whereas the other one in a pocket formed by Trp 19 and Tyr 148; both interact by *π*–*π* interactions with the indicated residues [22]. A red area is located near the positively charged amine in the fentanyl structure, indicative of the presence of a negatively charged amino acid (Asp 147) in this region which can interact with the ligand group by a salt-bridge. The blue area near the fentanyl’s carbonyl group indicates a region able to interact with one of the oxygen atoms, presumably represented by the His 297.

### 2.2. Activity Cliffs in the Activity Landscape of the QSAR Set

Usually, the activity cliff definition is strictly related to the activity landscape concept. An activity landscape is usually considered as a hypersurface where potency/activity of a particular compound is added as a third dimension to a 2D projection of the chemical space. In this concept, discontinuous SARs are regions termed as activity cliffs, which are formed by pairs of structurally similar compounds with large differences in potency. In these areas, the similarity hypothesis (structurally similar compounds will have similar biological activities) breaks down, and these areas are the most useful regions of the activity landscape for a compound series, gaining an improved understanding of interactions with the target protein. To facilitate this task, a methodology known as *activity miner* (AM) has been applied to the presented dataset of fentanyl-like compounds [23]. This methodology is based on the same XED/FieldAlign technology used for the development of the QSAR model. Specifically, a 3D-similarity metric was used, taking advantage of the same alignment produced for the development of the 3D-QSAR model, which was the best performing platform for predictive and descriptive capabilities. Fundamental to the application of the AM calculation is the concept of disparity, defined as the difference between the activities of two molecules divided by the distance (similarity) between them. High disparity values are obtained when the similarity is high, and the difference in activity is large—i.e., a small change in the molecule has made a substantial change in the activity. The seven activity cliffs with the highest values of disparity founded with the AM approach are reported in Table 2. From this analysis, it is clear that the presence of a substituent on the R^3^ (Figure 1) is fundamental for a high activity, and the presence of a simple hydrogen atom drops down the potency (Table 2, entries 3, 5, and 7). Moreover, an alkyl chain with more than two atoms will produce less potent analogs (Table 2, entries 1–3). The presence of a different aromatic substituent in R^5^ is tolerated (Table 2, entry 5), whereas its removal produces less active compounds (Table 2, entries 6 and 7). The results of the activity cliffs analysis suggest to further investigate the activity landscape inherent to suitable R^3^ and R^5^ substitutions.

### 2.3. Enlarging the Activity Landscape of Fentanyl-like Compounds

**Figure 4:**
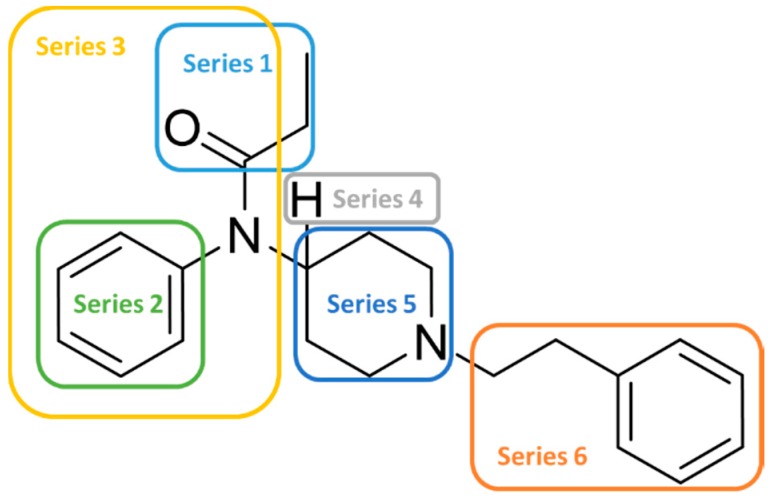
Selected portions of the fentanyl structure for the scaffold-hopping approach.

In order to enlarge the chemical landscape evaluation of fentanyl-like compounds, a bioisosteric and fragment replacement software tool (Spark v10.4.0, Cresset, New Cambridge House, Hertfordshire, United Kingdom) was adopted to produce a scaffold-hopping analysis and to generate a virtual library of μOR ligands [24,25], investigating not only the appropriate replacement of the R^3^ and R^5^ substituents, as suggested by the AM analysis, but also the decoration of other selected portions present in the original structure of fentanyl (Figure 4, R^1^, R^2^, and R^4^ substituents). In particular, the molecule was divided into six different parts to produces as much series (Figure 4), and 500 new virtual molecules were generated for each substitution pattern for a total of 3000 analogs (see Supplementary material, Tables S3–S8). Subsequently, each ligand was evaluated by exploiting the predictive capabilities of the 3D-field and 2D-kNN QSAR models. For each case, the replacement was performed using the same dataset of fragments already reported by us [26]. The top-scored compounds, according to the median of the three models (3D-field, 2D-ECFP6 and 2D-FCFP6), and considering only values within the domain of applicability of each model, are reported in Table 3. The results outline that the replacement generated new structures with optimized chemical features for the binding to the μOR. The results of all the series demonstrate that the chemical landscape for this class of compounds is still huge and small modifications may further increase the activity of the parent molecule. Series 1–3 produced compounds with relatively low predicted affinities, possessing p*K*_i_ ≤ 9, demonstrating that, potentially, any substituent in these regions should not produce powerful and potentially harmful compounds. This suggests that the activity landscape around this area can be exploited to prepare only novel fentanyl-like compounds with reduced potency. Conversely, the results obtained from Series 4–6 confirmed again that the R^3^ position (Figure 1) is the one that mostly affects the affinity increase, and that exploring the chemical space around this area, even using a cyclic system as suggested by our results, would typically produce highly potent ligands. While this data could be used advantageously for producing more effective radiotracers or to placate animals [27], these findings are a double-edged sword, and serious risks for human health must be taken into account.

### 2.4. Designer Drugs from the Scaffold-Hopping Results

Newly-identified fentanyl analogs often lack any in vitro and/or in vivo pharmacology data. Some of the identified molecules are temporarily placed by the DEA in the US Schedule I waiting for complete analyses and data, which can be a time-consuming exercise. Virtual screening may represent a valid and faster alternative, and it was already reported that structure-based methods might allow speeding-up the identification and classification of potentially toxic compounds [11]. To further validate the predicting capabilities of our consensus QSAR ligand-based approach, we decided to investigate previously reported designer drugs—also known as Novel Psychoactive Substances (NPS)—among the results of the scaffold hopping approach. Interestingly, a number of already reported designer fentanyl-like compounds were found, including furanylfentanyl, acetylfentanyl, acrylfentanyl, benzodioxolefentanyl, cyclopentylfentanyl, tetramethylcyclopropylfentanyl, tetrahydrofuranylfentanyl, 3-furanylfentanyl, *a*-methylfentanyl, acryloylfentanyl, cyclopropylfentanyl, 4-fluorofentanyl, methoxyacetylfentanyl, 3-methylfentanyl, 4-fluoroisobutyrylfentanyl, 4-chlorisobutyrfentanyl, 4-methoxybutyrfentanyl, *β*-hydroxythiofentanyl and isobutyrylfentanyl, predicted to have p*K*i of 8.5, 8.3, 8.5, 8.4, 8.3, 7.9, 8.2, 8.4, 8.7, 8.5, 8.6, 7.9, 8.1, 8.5, 7.8, 8.0, 8.0, 8.1 and 8.6, respectively. All of these compounds are potent μOR ligands and some of them have already been attributed to a large number of fatalities worldwide [28,29]. All such NPS were identified as potent ligands by our methodology, strengthening the quality of our consensus methodology.

## 3. Materials and Methods

### 3.1. Biological Data

The chemical structures of all the molecules were retrieved from the ChEMBL database selecting only those tested for their affinity for the human μOR (available online: https://www.ebi.ac.uk/chembl/target/inspect/CHEMBL233). Datawarrior (5.0.0, Idorsia Pharmaceuticals Ltd., Allschwil, Switzerland) [30] was used for handling the selection of the molecules with fentanyl-like structures among the entire downloaded dataset. Only molecules where the displacement of the radioligand [^3^H]DAMGO from the human μOR was used for the determination of all of the *K*_i_ values, were selected. The binding affinity data were converted into their negative decimal logarithm p*K*_i_ (p*K*_i_ = −log*K*_i_).

### 3.2. Molecular Modeling

The two-dimensional structures of the dataset were built using Marvin Sketch (18.24, ChemAxon Ltd., Budapest, Hungary). The protonation states of the molecules were calculated assuming a neutral pH, and the Merck molecular force field (MMFF94) was used for a first 3D geometry optimization. Subsequently, the geometry of the resulting 3D structures was optimized at a semi-empirical level using the parameterized model number 3 (PM3) Hamiltonian as implemented in the MOPAC package (MOPAC2016 v. 18.151, Stewart Computational Chemistry, Colorado Springs, CO, USA) [31,32].

### 3.3. Compound Alignment for the 3D Model and kNN Models Information

All the fentanyl-like 3D-optimized structures were imported into the software Forge (v10.4.2, Cresset, New Cambridge House, Hertfordshire, UK) [23] to set the field-based 3D-QSAR model and the 2D k-Nearest Neighbor (kNN) models. Out of the 115 structures, 94 molecules were randomly selected as a training set while the remaining 21 were used as an external validation (test set) to evaluate the models [33] (Tables S1 and S2). The molecules in the training set and the test set covered a p*K*_i_ range from 10.1 to 5.3. Ten different splits were tried and the results were consistent to the reported ones. All the fentanyl-like molecules were aligned using fentanyl in its previously reported conformation [22]. The field points (negative and positive electrostatic, van der Waals shape, and hydrophobic description of the molecules) were generated using the extended electron distribution (XED) force field included in Forge. All the software parameters used for the conformation hunt, alignment, and model building calculations are presented in the Supplementary material (Figure S1–S6, Tables S1 and S2). As an alternative to the 3D-field QSAR, we also developed other two QSAR models using the kNN method with the same software. The kNN approach is well-known, robust and has an effective distance learning approach [34,35]. The two kNN models were developed using two different 2D-fingerprint similarities: the ECFP6 and the FCFP6 circular fingerprint descriptors. More detailed information for the kNN models generations is reported in the Supplementary material.

## 4. Conclusions

The present study explores the development of three QSAR models exploitable for the prediction of a ligand affinity to the μOR, and for the identification of new molecules that could efficiently interact with such receptor class. Forge was employed to build a statistically robust QSAR evaluating methodology using a set of 115 fentanyl-analogs covering a wide range of known μOR ligands. An activity cliff analysis followed by a scaffold-hopping approach has been performed to provide a theoretical route for the exploration of novel unidentified fentanyl-analogs with high potency. The QSAR models reported here will guarantee, prospectively, fruitful applications to speed up the design and the identification process of new μOR ligands with tunable activities. Since μOR fentanyl-like ligands currently represent a well-explored class of synthetic opioids, often associated with fatal cases worldwide, this proposed ligand-based tool could be considered by the DEA, the European Monitoring Centre for Drugs and Drug Addiction (EMCDDA), and other regulatory bodies for speeding-up the classification of novel fentanyl-like NPS. Likewise, the newly identified libraries may potentially aid the interpretation of toxicological analyses where the presence of novel synthetic opioids is postulated.

## Figures and Tables

**Table 1:** Models statistics.

Model	r^2^ Training Set	q^2^ Training Set	r^2^ Test Set	MSE ^a^ Training Set	MSE ^a^ Test Set	MAE ^b^ Training Set	MAE ^b^ Test Set	MAPE ^c^ Training Set	MAPE ^c^ Test Set
3D-field	0.99	0.68	0.77	0.005	0.22	0.05	0.41	0.75	5.75
FCFP6 kNN	0.68	0.65	0.59	0.35	0.40	0.44	0.53	6.25	7.26
ECFP6 kNN	0.71	0.70	0.61	0.31	0.39	0.44	0.54	6.26	7.39

^a^ Mean squared forecast error; ^b^ Mean absolute forecast error; ^c^ Mean absolute percentage forecast error.

**Table 2:** Activity cliffs resulted from the AM approach.

Entry	Structure and p*K*_i_ of the Most Active Analogs	Structure and p*K*_i_ of the Less Active Analogs	Disparity	Δ Activity
1	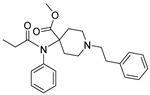 10.15	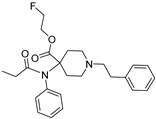 8.92	−24.6	−1.23
2	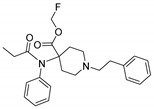 9.89	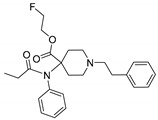 8.92	−19.4	−0.97
3	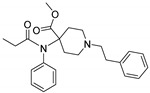 10.15	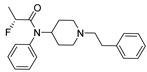 8.68	−15.4	−1.47
4	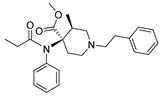 9.85	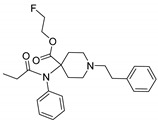 8.92	−14.2	−0.93
5	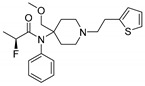 9.89	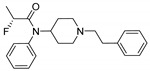 8.68	−14.1	−1.21
6	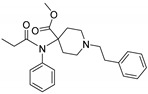 10.15	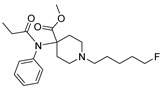 9.13	−13.8	−1.02
7	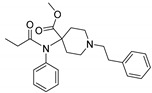 10.15	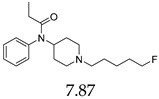 7.87	−12.8	−2.28

**Table 3:** Selected molecules resulted from the scaffold-hopping approach.

**Series 1**	**Series 2**	**Series 3**
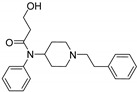 9.0	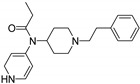 9.0	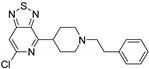 8.9
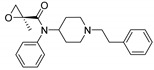 8.8	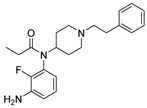 8.9	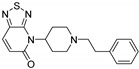 8.8
**Series 4**	**Series 5**	**Series 6**
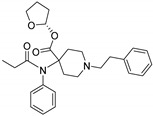 10.8	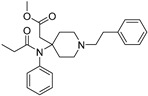 9.8	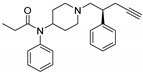 9.5
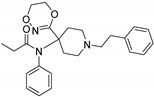 10.6	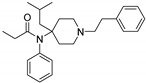 9.7	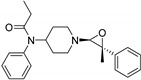 9.4

## Supplementary Materials

Supplementary materials can be found at https://www.mdpi.com/1422-0067/20/9/2311/s1.
