# Supplementary material for: Structure-Based Approach for the Prediction of Mu-opioid Binding Affinity of Unclassified Designer Fentanyl-Like Molecules
Source: Int J Mol Sci. 2019 May 10;20(9):2311. doi: 10.3390/ijms20092311 (PMC6539757; doi:10.3390/ijms20092311)
Supplement: Supplementary file 1 [file ijms-20-02311-s001.pdf]

International Journal of *Molecular Sciences*

MDPI

Supplementary material

Structure-based approach for the prediction of mu-opioid binding affinity of unclassified designer fentanyl-like molecules

Giuseppe Floresta [1,*], Antonio Rescifina [1] and Vincenzo Abbate [2,*]

[1] Department of Drug Sciences, University of Catania, V.le A. Doria, 95125 Catania, Italy
[2] King's Forensics, School of Population Health & Environmental Sciences, King's College London, Franklin-Wilkins Building, 150 Stamford Street, London SE1 9NH
* Correspondence: giuseppe.floresta@unict.it (G.F), vincenzo.abbate@kcl.ac.uk (V.A)
Received: date; Accepted: date; Published: date

Table of content

Statistical analysis information for the model S2
Fig. S1. Forge's parameters used for the conformation hunt S3
Fig. S2. Forge's parameters used for the alignment S3
Fig. S3. Forge's parameters used for the build of the model S4
Fig. S4. Model statistics for the 3D-field model S4
Fig. S5. Model statistics for the 2D-FCFP6 kNN model S4
Fig. S6. Model statistics for the 2D-ECFP6 kNN model S5
Table S1. SMILES, experimental and predicted pK_i values of the molecules in the training set S5
Table S2. SMILES, experimental and predicted pK_i values of the molecules in the test set S7
Table S3. List, SMILE and predicted pK_i values for Series 1 S8
Table S4. List, SMILE and predicted pK_i values for Series 2 S20
Table S5. List, SMILE and predicted pK_i values for Series 3 S32
Table S6. List, SMILE and predicted pK_i values for Series 4 S44
Table S7. List, SMILE and predicted pK_i values for Series 5 S56
Table S8. List, SMILE and predicted pK_i values for Series 6 S68

Statistical analysis information for the models

The following conditions were used to calculate the field 3D-QSAR model. The leave-one-out method was used for the validation of the QSAR model. The maximum number of components to extract from the PLS regression was set to 20. The number of Y scrambles to use was set to 50, this means that in each scramble the activity values are randomly assigned to molecules and the model building process is repeated. More scramble sets provide stronger confirmation of statistical significance. The sample point minimum distance threshold was set to 1 Å. This option checks the sphere exclusion algorithm used to reduce the initial number of field sample positions down to a smaller set. Decrease the value sample point minimum distance increases the number of sample points, which may improve the model at the expense of increasing the probability of over-fitting. A value of 1 Å means that sample points must be at least 1 Å aside from each other. The predictive ability of the generated model was confirmed by different statistical tests. The leave-one-out method was used during the validation of the QSAR model which means that the model is built again but a single molecule left out of the process, this is then repeated leaving out each training set molecule in turn. The predicted activity for each molecule is the value obtained when it was left out of the model building process. The cross-validation regression coefficient (q^2) was calculated based on the prediction error sum of squares (PRESS) and the sum of squares of deviation of the experimental values from their mean (SSY):

$$q^2 = 1 - \frac{PRESS}{SSY} = 1 - \frac{\sum_{i=1}^{n}(Yexp - Ypred)^2}{\sum_{i=1}^{n}(Yexp - Ymean)^2}$$

where *Yexp* is the experimental activity of training set compound, *Ypred* is the predicted activity of training set compound, and *Ymean* is the mean values of the activity of training set compound.

The performance of the model was also validated through the determination of the coefficient in prediction, r^2_{test}, using the following equation:

$$r^2 test = 1 - \frac{\sum_{i=1}^{n}(Ypredtest - Ytest)^2}{\sum_{i=1}^{n}(Ytest - Ymean)^2}$$

where *Ypredtest* is the predicted activity of test set compound by QSAR equation, *Ytest* is the experimental activity of test set compound, and *Ymean* is the mean values of the activity of training set compound.

The two kNN QSAR models were developed using a 2D fingerprint similarity (FCFP6 and ECFP6). The optimal k value were selected by Leave-One-Out cross-validation: each compound in the training set is removed in turn from the modelling and its activity is predicted as the average activity of its k nearest neighbors. These training set predicted activities are used to calculate a q2 value for the model Figure (S5,6).

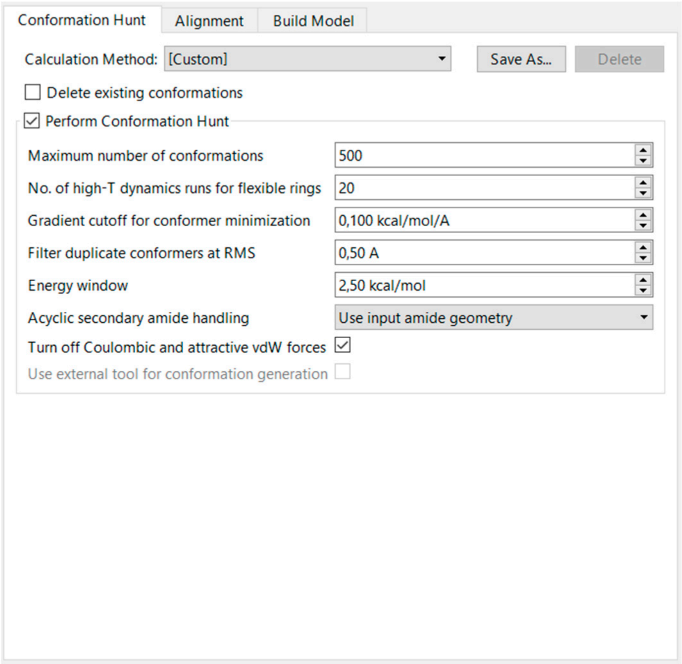

Fig. S1. Forge's parameters used for the conformation hunt.

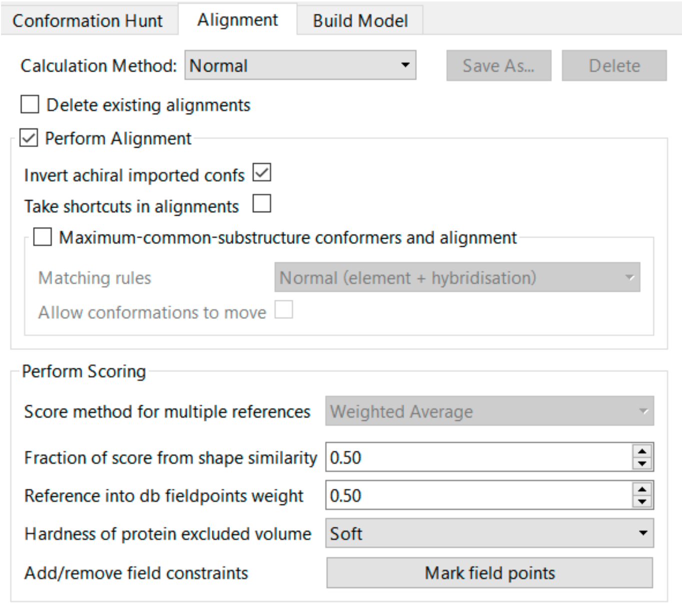

Fig. S2. Forge's parameters used for the alignment.

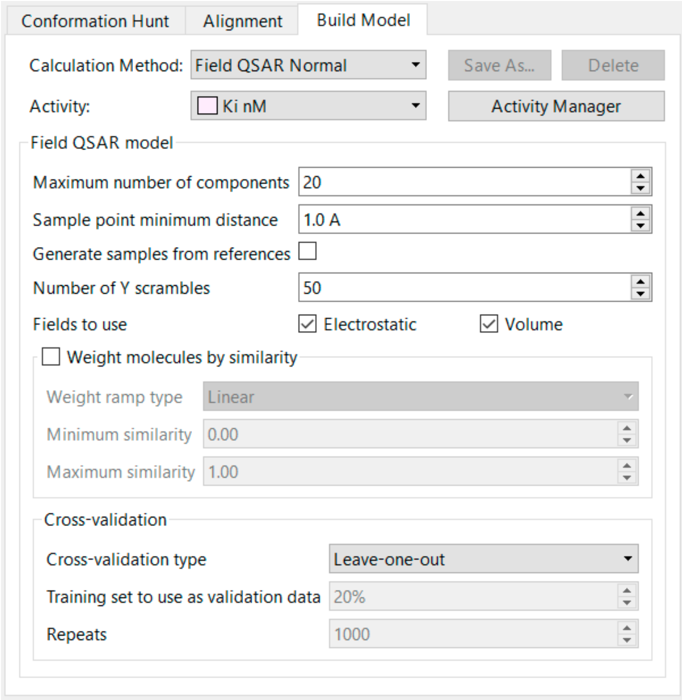

Fig. S3. Forge's parameters used for the build of the model.

Model statistics:
=================

Comps	R^2	Q^2	Test R^2	RMSE	RMSEpred	Tau	Tau-pred
0	0.000	-0.021	-0.088	1.026	1.037	0.156	-0.980
1	0.488	0.229	0.473	0.734	0.901	0.484	0.331
2	0.725	0.413	0.580	0.538	0.787	0.588	0.384
3	0.822	0.501	0.620	0.433	0.725	0.667	0.435
4	0.908	0.587	0.754	0.311	0.660	0.752	0.474
5	0.934	0.602	0.735	0.263	0.647	0.785	0.471
6	0.966	0.631	0.736	0.190	0.623	0.824	0.494
7	0.977	0.648	0.748	0.154	0.609	0.871	0.527
8	0.989	0.667	0.758	0.108	0.592	0.924	0.540
9	0.994	0.672	0.755	0.080	0.588	0.939	0.550
10*	0.997	0.677	0.751	0.059	0.584	0.957	0.552
11	0.998	0.675	0.754	0.041	0.585	0.971	0.548
12	0.999	0.675	0.754	0.034	0.585	0.975	0.550
13	0.999	0.679	0.754	0.025	0.582	0.978	0.551
14	1.000	0.681	0.754	0.018	0.580	0.985	0.556
15	1.000	0.682	0.753	0.014	0.579	0.987	0.554
16	1.000	0.683	0.752	0.011	0.578	0.990	0.555
17	1.000	0.683	0.754	0.008	0.578	0.989	0.559
18	1.000	0.683	0.754	0.006	0.578	0.991	0.560
19	1.000	0.683	0.753	0.004	0.578	0.993	0.559
20	1.000	0.683	0.753	0.003	0.578	0.991	0.560

Fig. S4. Model statistics for the 3D-field model.

Model statistics:
=================

K	Distance		Q^2	RMSEpred	Tau-pred
	Avg	Std. dev.			
0	0.00	1.00	-0.023	1.038	-0.933
1	0.30	0.12	0.608	0.642	0.615
2*	0.33	0.12	0.650	0.607	0.596
3	0.36	0.12	0.590	0.657	0.554
4	0.38	0.13	0.578	0.667	0.550
5	0.40	0.13	0.555	0.685	0.543
6	0.41	0.13	0.581	0.664	0.531
7	0.43	0.12	0.578	0.667	0.525
8	0.45	0.12	0.581	0.664	0.511
9	0.46	0.12	0.574	0.670	0.514
10	0.47	0.11	0.574	0.670	0.541
11	0.49	0.11	0.565	0.677	0.523
12	0.50	0.11	0.554	0.685	0.512
13	0.52	0.11	0.552	0.687	0.499
14	0.53	0.10	0.536	0.699	0.480
15	0.54	0.10	0.526	0.706	0.464
16	0.55	0.10	0.517	0.713	0.458
17	0.56	0.10	0.498	0.727	0.445
18	0.57	0.10	0.481	0.739	0.440
19	0.57	0.09	0.481	0.739	0.443
20	0.58	0.09	0.462	0.753	0.432

Fig. S5. Model statistics for the 2D-FCFP6 kNN model.

```
Model statistics:
=================

K         Distance              Q^2       RMSEpred Tau-pred
          Avg       Std. dev.
0         0.00      1.00      -0.022    1.040     -0.948
1         0.37      0.14      0.649     0.609     0.642
2         0.41      0.13      0.690     0.572     0.618
3*        0.43      0.12      0.709     0.555     0.617
4         0.45      0.12      0.690     0.573     0.606
5         0.47      0.12      0.697     0.566     0.607
6         0.49      0.12      0.684     0.578     0.602
7         0.50      0.12      0.677     0.585     0.594
8         0.51      0.12      0.655     0.604     0.579
9         0.53      0.12      0.642     0.616     0.571
10        0.55      0.11      0.625     0.630     0.563
11        0.56      0.11      0.615     0.638     0.563
12        0.58      0.11      0.598     0.652     0.558
13        0.59      0.10      0.581     0.666     0.551
14        0.60      0.10      0.561     0.681     0.544
15        0.61      0.10      0.546     0.693     0.532
16        0.62      0.09      0.534     0.702     0.537
17        0.63      0.09      0.518     0.714     0.523
18        0.64      0.09      0.515     0.716     0.531
19        0.65      0.08      0.502     0.725     0.522
20        0.66      0.08      0.486     0.737     0.515
```

Fig. S6. Model statistics for the 2D- ECFP6 kNN model.

Table S1

SMILES, experimental and predicted pK_i values of the molecules in the training set.

		pK_i	
**N°**	**SMILES**	**Exp**	**Pred**
1	FCOC(=O)C1(N(c2ccccc2)C(=O)CC)CC[N+](CC1)CCc3ccccc3	9.9	9.9
2	O=C1[C@H](c2ccccc2N1C3CC[N+](CC3)CCC(c4ccccc4)c5ccccc5)CC	8.7	8.7
3	O=C1Cc2ccccc2N1C3CC[N+](C4C5CCCC4CCC5)CC3	8.6	8.6
4	O=C1[C@@H](c2ccccc2N1C3CC[N+]([C@@H]4c5cccc6CCC[C@H](c65)CC4)CC3)CC	8.1	8.2
5	O=C1[C@H](c2ccccc2N1C3CC[N+](CC3)Cc4cccc5c4cc[nH]5)CC	8.0	8.1
6	OCCOc1cccc(-c2nc3ccccc3n2C4CC[N+](C5(CCCCCCC5)C)CC4)c1	8.0	8.0
7	O=C1Cc2ccccc2N1C3CC[N+](C4CCC(CC4)C(CC)(C)C)CC3	7.8	7.8
8	O=C1Cc2ccccc2N1C3CC[N+]([C@H]4c5cccc6CCC[C@H](c65)CC4)CC3	7.5	7.4
9	O=C(N(C1CC[N+](CC1)CCc2ccccc2)CCCCCCCCNC(N)=[N+])CC	7.4	7.4
10	Clc1c(F)ccc(-c2nc3ccccc3n2C4CC[N+](C5(CCCCCCC5)C)CC4)c1	7.4	7.4
11	O=C(N(C)C)[C@@H]1Cc2ccccc2N1C(=O)CC[N+]3CCC(CC3)c4ccccc4C	7.2	7.1
12	Clc1ccc(O)c2c1C[C@@H]([N+]C2)C[N+]3CCC4(CC3)c5ccccc5CC4	7.2	7.2
13	O=C(N(C)C)[C@@H]1Cc2ccccc2N1C(=O)CC[N+]3CCC(CC3)c4ccccc4	7.1	7.1
14	O=C(N1c2ccccc2C[C@H]1C(=O)N(C)C)CC[N+]3CCC(CC3)c4c(cccc4C)C	7.1	7.2
15	O=C(N(C)C)[C@@H]1Cc2ccccc2N1C(=O)CC[N+]3CCC(CC3)c4ccccc4C	6.9	6.9
16	OCC[N+]1C[C@@H]2[C@H](C1)CN(C32CC[N+](C4CCC(CC4)C(C)C)CC3)c5ccccc5	6.8	6.7
17	O=C1c2ccccc2C[C@H](N1C)C[N+]3CCC4(CC3)c5ccccc5CC4	6.6	6.6
18	CC(C1CCC([N+]2CCC3([C@H]4[C@H](C[N+](C4)CCCC)CN3c5ccccc5)CC2)CC1)C	6.6	6.5
19	Fc1ccc(C2CC[N+](C[C@@H]3Cc4ccccc4C[N+]3)CC2)c(c1)C	6.5	6.4
20	[N+]1([C@@H]2c3cccc4cccc(C2)c43)CCC(CC1)c5c[nH]c6c5cccn6	6.4	6.4
21	O=C(N1CCC2(CC1)c3ccccc3CC2)[C@@H]4Cc5ccccc5C[N+]4	6.4	6.3
22	Fc1ccc2c(c(C3CC[N+](CC3)Cc4cccc5ccccc54)c[nH]2)c1	6.3	6.3
23	Fc1ccc2c(n(C[C@H](O)C[N+](C)C)cc2C3CC[N+]([C@H]4c5cccc6cccc(C4)c65)CC3)c1	6.1	6.1
24	Fc1ccc2c([nH]cc2C3CC[N+]([C@H]4c5cccc6cccc(C4)c65)CC3)c1	6.1	6.1
25	O=C(NCC1(Nc2ccccc2)CC[N+](CC1)Cc3ccccc3)CNC(N)=[N+]	5.3	5.4

26	[N+]1(CC2CCCCC2)CCC(CC1)c3c[nH]c4c3cccn4	5.3	5.4
27	O=C1[C@H](c2ccccc2N1C3CC[N+](CC3)CCCc4ccccc4)CC	8.0	8.0
28	CC(C1CCC([N+]2CCC3([C@@H]4C[N+](C[C@@H]4CN3c5ccccc5)CC[N+]6CCOCC6)CC2)CC1)C	6.8	6.8
29	Clc1ccc2c(c(C3CC[N+]([C@H]4c5cccc6cccc(C4)c65)CC3)c([nH]2)C)c1	6.6	6.6
30	O=C(N1Cc2ccccc2C[C@@H]1C[N+]3CCC4(CC3)c5ccccc5CC4)N	7.0	7.0
31	OCC1([N+]2CCC(n3c4ccccc4nc3N5C[C@H]6C[N+](C[C@H]6C5)Cc7ccccc7)CC2)CCCCCCC1	6.9	6.9
32	OCC1([N+]2CCC(n3c4ccccc4nc3N5C[C@H]6C[N+]C[C@H]6C5)CC2)CCCCCCC1	6.7	6.7
33	O=C(OC)C1([N+]2CCC(n3c4ccccc4nc3N5CC[N+](CC5)C)CC2)CCCCCCC1	6.4	6.4
34	C[N+]1C[C@@H]2CN(C3([C@@H]2C1)CC[N+](C4CCCCCCCC4)CC3)c5ccccc5	7.1	7.0
35	Clc1ccc2c(c(C3CC[N+](CC3)Cc4cccc5ccccc54)c([nH]2)C)c1	6.1	6.0
36	[N+]Cc1cccc(-c2nc3ccccc3n2C4CC[N+](C5(CCCCCCC5)C)CC4)c1	7.7	7.7
37	O=C1Cc2ccccc2N1C3CC[N+]([C@H]4c5cccc6CCC[C@@H](c65)CC4)CC3	7.1	7.2
38	O=C1[C@H](c2ccccc2N1C3CC[N+](CC3)CCCCc4ccc(-c5ccccc5)cc4)CC	6.8	6.8
39	Clc1ccc(-c2nc3ccccc3n2C4CC[N+](C5(CCCCCCC5)CO)CC4)cc1	6.8	6.7
40	[N+]1(C[C@@H]2Cc3ccccc3C[N+]2)CCC4(CC1)c5ccccc5CC4	6.7	6.6
41	Clc1c(F)ccc(-c2nc3ccccc3n2C4CC[N+](C5(CCCCCCC5)CO)CC4)c1	6.5	6.5
42	CCCCCC[N+]1CCC(CC1)c2c[nH]c3c2cccn3	5.3	5.2
43	OCC1([N+]2CCC(n3c4ccccc4nc3N5CC[N+]C(C5)(C)C)CC2)CCCCCCC1	6.4	6.3
44	O=C(NCC1(Nc2cc(OC)cc(OC)c2)CC[N+](CC1)Cc3ccccc3)CNC(N)=[N+]	6.2	6.2
45	C[N+]1C[C@@H]2[C@H](C1)CN(C32CC[N+](C4CCC(CC4)C(C)C)CC3)c5ccccc5	6.5	6.4
46	Clc1ccc2c(c(C3CC[N+](CC3)Cc4ccccc4)c[nH]2)c1	6.6	6.6
47	F[C@H](C(=O)N(C1(CC[N+](CC1)CCc2cccs2)COC)c3ccccc3)C	9.9	9.7
48	FCCCCC[N+]1CCC(N(C(=O)CC)c2ccccc2)(CC1)C(OC)=O	9.1	9.3
49	O=C1Cc2ccccc2N1C3CC[N+](CC3)[C@H](c4ccccc4)C	8.6	8.6
50	O=C1Cc2ccccc2N1C3CC[N+]([C@@H](C4CCCCC4)C)CC3	8.5	8.3
51	O=C1[C@H](c2ccccc2N1C3CC[N+]([C@H]4Cc5ccccc5CC4)CC3)CC	8.0	8.0
52	C[N+]1Cc2ccccc2C[C@@H]1C[N+]3CCC4(CC3)c5ccccc5CC4	7.2	7.2
53	O(c1ccc(C[N+]2CCC(CC2)c3c[nH]c4c3cccn4)cc1)c5ccccc5	6.5	6.4
54	Fc1ccc(N2C[C@H]3C[N+](C[C@H]3C42CC[N+](C5CCC(CC5)C(C)C)CC4)CC6CC6)cc1	6.2	6.4
55	O=C(N(CC)C)C1(CC[N+](CC1)CCCCCC)c2cccc(O)c2	8.4	8.4
56	OCC1([N+]2CCC(N3C(=O)Nc4ccccc43)CC2)CCCCCCC1	7.8	7.8
57	Clc1ccc(O)c2c1C[C@H]([N+]C2)C[N+]3CCC(CC3)c4ccc(F)cc4C	6.4	6.4
58	O=C(N1c2ccccc2C[C@H]1C(=O)N(C)C)CC[N+]3CCC([N+]4CCCCC4)CC3	6.4	6.3
59	O=C1[C@H](c2ccccc2N1C3CC[N+]([C@H]4c5ccccc5CCC4)CC3)CC	9.0	9.0
60	OCC1([N+]2CCC(n3c4ccccc4nc3N5CC[N+](CC5)C)CC2)CCCCCCC1	6.6	6.7
61	O=C(OC)[C@]1(N(c2ccccc2)C(=O)CC)CC[N+](C[C@H]1C)CCc3ccccc3	9.9	9.8
62	O=C(N1Cc2ccccc2C[C@@H]1C[N+]3CCC4(CC3)c5ccccc5CC4)C	7.1	7.3
63	CC(C1CCC([N+]2CCC3([C@@H]4CN(C[C@@H]4CN3c5ccccc5)c6ccccc6)CC2)CC1)C	6.9	6.8
64	O1c2ccccc2C[C@H](C[N+]3CCC4(CC3)c5ccccc5CC4)C1	7.3	7.2

65	CC(C1CCC([N+]2CCC3([C@@H]4C[N+](C[C@@H]4CN3c5ccccc5)Cc6ccccc6)CC2)CC1)C	6.8	6.9
66	CC1([N+]2CCC(n3c4ccccc4nc3N5C[C@@H]([N+][C@@H](C5)C)C)CC2)CCCCCCC1	7.7	7.7
67	Clc1c(F)ccc(-c2nc3ccccc3n2C4CC[N+](C5(CCCCC5)CO)CC4)c1	6.4	6.4
68	O=C1[C@@H](c2ccccc2N1C3CC[N+](C4CCC(CC4)C(C)C)CC3)C	8.4	8.4
69	FCCCCC[N+]1CCC(N(C(=O)CC)c2ccccc2)CC1	7.9	7.9
70	Oc1cccc2c1C[N+][C@@H](C[N+]3CCC4(CC3)c5ccccc5CC4)C2	6.5	6.6
71	N/C(=[N+]\c1ccc2c(nc(n2C3CC[N+](CC3)C)Cc4ccc(OCC)cc4)c1)c5cccs5	5.5	5.5
72	O=C1[C@H](c2ccccc2N1C3CC[N+](C4CCC(CC4)C(C)C)CC3)CC	9.0	9.0
73	O=C1[C@@H](c2ccccc2N1C3CC[N+]([C@H]4c5cccc6CCC[C@H](c65)CC4)CC3)CC	8.8	8.8
74	O=C1Cc2ccccc2N1C3CC[N+](C4CCC(CC4)CCC)CC3	8.3	8.2
75	CC1([N+]2CCC(n3c(nc4ccccc43)-c5cc6ccccc6o5)CC2)CCCCCCC1	7.0	7.0
76	Clc1cccc2c1[nH]cc2C3CC[N+]([C@H]4c5cccc6cccc(C4)c65)CC3	6.6	6.5
77	OCC1([N+]2CCC(n3c4ccccc4nc3N5CC[N+][C@@H](C5)C)CC2)CCCCCCC1	6.4	6.4
78	CC(C1CCC([N+]2CCC3([C@@H]4C[N+]C[C@@H]4CN3c5ccccc5)CC2)CC1)C	6.3	6.3
79	Fc1ccc2c(c(C3CC[N+](CC4CCCCCCC4)CC3)cn2C[C@H](O)C[N+](C)C)c1	5.6	5.5
80	O=C(NCC1(Nc2cccc3ccccc32)CC[N+](CC1)Cc4ccccc4)CNC(N)=[N+]	5.7	5.7
81	Fc1ccc2c(c(C3CC[N+]([C@H]4c5cccc6cccc(C4)c65)CC3)c[nH]2)c1	6.3	6.3
82	O=C1Cc2ccccc2N1C3CC[N+](C4CCCCCCC4)CC3	7.5	7.7
83	Cn1c2c(c(C3CC[N+]([C@H]4c5cccc6cccc(C4)c65)CC3)c1)cccn2	6.1	6.0
84	CC1([N+]2CCC(n3c(nc4ccccc43)-c5ccc6cc[nH]c6c5)CC2)CCCCCCC1	6.8	6.8
85	Fc1ccc(C2CC[N+](CC2)CCC(=O)N3c4ccccc4C[C@H]3C(=O)N(C)C)cc1	6.6	6.6
86	CC1([N+]2CCC(n3c4ccccc4nc3[C@H]5CCC[N+](C5)C)CC2)CCCCCCC1	8.0	7.9
87	[N+]1(CC2CCCCCCC2)CCC(CC1)c3c[nH]c4c3cccn4	5.7	5.7
88	O=C(N(C)C)[C@@H]1Cc2ccccc2N1C(=O)CC[N+]3CCC(CC3)c4ccccc4	6.3	6.3
89	[N+]1(CCC(CC1)c2c[nH]c3c2cccn3)Cc4cccc5ccccc54	6.5	6.5
90	OCCn1c2c(c(C3CC[N+]([C@H]4c5cccc6cccc(C4)c65)CC3)c1)cccn2	6.3	6.3
91	Clc1cccc2c1[nH]cc2C3CC[N+](CC4CCCCCCC4)CC3	6.4	6.3
92	O=C1CCc2ccccc2N1C3CC[N+](C4CCC(CC4)C(C)C)CC3	8.1	8.1
93	Fc1ccc2c(c(C3CC[N+](CC4CCCCCCC4)CC3)c[nH]2)c1	6.0	6.0
94	O=C1[C@H](c2ccccc2N1C3CC[N+](C4CCCCCCC4)CC3)C	8.0	8.0

Table S2

SMILES, experimental and predicted pK_i values of the molecules in the test set.

		pK_i	
**N°**	**SMILES**	**Exp**	**Pred**
1	O=C(OC)C1(N(c2ccccc2)C(=O)CC)CC[N+](CC1)CCc3ccccc3	10.2	9.8
2	O=C1[C@H](c2ccccc2N1C3CC[N+](C4CCCCCCC4)CC3)CC	7.8	7.7
3	O=C1Cc2ccccc2N1C3CC[N+]([C@@H]4c5cccc6CCC[C@H](c65)CC4)CC3	7.4	7.6
4	FC(F)(F)c1cccc(-c2nc3ccccc3n2C4CC[N+](C5(CCCCCCC5)C)CC4)c1	7.2	6.8

5	C[N+]1C[C@@H]2CN(C3([C@@H]2C1)CC[N+]([C@H]4CC[C@@H]5CCCC[C@@H]5C4)CC3)c6ccccc6	6.4	6.2
6	O=C1Cc2ccccc2N1C3CC[N+](C4CCC(CC4)C(C)C)CC3	8.1	8.1
7	[N+]1(C[C@H]2Cc3ccccc3CC2)CCC4(CC1)c5ccccc5CC4	6.5	6.7
8	O=C(N(C1CC[N+](CC1)CCc2ccccc2)CCCNC(N)=[N+])CC	7.6	8.0
9	Brc1ccc(O)c2c1C[C@@H]([N+]C2)C[N+]3CCC4(CC3)c5ccccc5CC4	6.9	6.6
10	Oc1ccc2c(C[C@H]([N+]C2)C[N+]3CCC4(CC3)c5ccccc5CC4)c1	6.4	6.2
11	O=C1CCc2ccccc2N1C3CC[N+](C4CCCCCCC4)CC3	7.3	8.0
12	FCCOC(=O)C1(N(c2ccccc2)C(=O)CC)CC[N+](CC1)CCc3ccccc3	8.9	9.7
13	Fc1ccc(C2CC[N+](CC2)CCC(=O)N3c4ccccc4C[C@H]3C(=O)N(C)C)c(C)c1	6.9	6.1
14	O=C1C[C@H](c2ccccc2N1C3CC[N+](C4CCCCCCC4)CC3)C	7.6	8.2
15	F[C@H](C(=O)N(C1CC[N+](CC1)CCc2ccccc2)c3ccccc3)C	8.7	8.5
16	C[N+]1C[C@@H]2CN(C3([C@@H]2C1)CC[N+](C4CCCCCCCC4)CC3)c5ccccc5	6.1	6.7
17	O=C1[C@@H](c2ccccc2N1C3CC[N+](CC3)C/C=C/c4ccccc4)CC	7.5	6.9
18	O=C1Cc2ccccc2N1C3CC[N+](CC3)Cc4ccccc4	7.0	7.6
19	O=C(NCCNC(N)=[N+])C1(Nc2ccccc2)CC[N+](CC1)Cc3ccccc3	6.7	7.5
20	[O-]C(=O)C1([N+]2CCC(n3c4ccccc4nc3N5CC[N+](CC5)C)CC2)CCCCCCC1	6.4	6.9
21	C[N+]1C[C@@H]2[C@H](C1)CN(C32CC[N+](C4CCC(CC4)C(C)C)CC3)c5ccccc5	6.3	6.7

Table S3

List, SMILE and predicted pK_i values for Series 1.

N°	SMILES	Pred pK_i
1	O=C(N(C1CC[N+](CC1)CCc2ccccc2)c3ccccc3)CCO	9.0
2	O=C(N(C1CC[N+](CC1)CCc2ccccc2)c3ccccc3)Cc4c[nH]cn4	9.0
3	O=C(N(C1CC[N+](CC1)CCc2ccccc2)c3ccccc3)Cc4c[nH]nn4	8.9
4	O=C(N(C1CC[N+](CC1)CCc2ccccc2)c3ccccc3)CCC=O	8.8
5	O=C(N(C1CC[N+](CC1)CCc2ccccc2)c3ccccc3)[C@@]4(CO4)C	8.8
6	O=C(N(C1CC[N+](CC1)CCc2ccccc2)c3ccccc3)CCOC	8.8
7	O=C(N(C1CC[N+](CC1)CCc2ccccc2)c3ccccc3)CCCC#N	8.8
8	O=C(N(C1CC[N+](CC1)CCc2ccccc2)c3ccccc3)CCOCC	8.8
9	O=C(N(C1CC[N+](CC1)CCc2ccccc2)c3ccccc3)Cc4ncc[nH]4	8.8
10	O=C(N)CCON(C1CC[N+](CC1)CCc2ccccc2)c3ccccc3	8.8
11	OC1(CC1)CC(=O)N(C2CC[N+](CC2)CCc3ccccc3)c4ccccc4	8.8
12	O=C(N(C1CC[N+](CC1)CCc2ccccc2)c3ccccc3)Cc4n[nH]cn4	8.8
13	O=C(N(C1CC[N+](CC1)CCc2ccccc2)c3ccccc3)CCCC=O	8.8
14	O=C(N(C1CC[N+](CC1)CCc2ccccc2)c3ccccc3)COC(=O)C	8.7
15	FC(F)C(=O)CC(=O)N(C1CC[N+](CC1)CCc2ccccc2)c3ccccc3	8.7
16	O=C(OC)CCON(C1CC[N+](CC1)CCc2ccccc2)c3ccccc3	8.7
17	O=C(N(C1CC[N+](CC1)CCc2ccccc2)c3ccccc3)CCOCC#C	8.7
18	O=C(N(C1CC[N+](CC1)CCc2ccccc2)c3ccccc3)CCC(OC)=O	8.7
19	O=C(N(C1CC[N+](CC1)CCc2ccccc2)c3ccccc3)/C=C/N	8.7

20	O=C(N(C1CC[N+](CC1)CCc2ccccc2)c3ccccc3)Cc4cc[nH]n4	8.7
21	O=C(N(C1CC[N+](CC1)CCc2ccccc2)c3ccccc3)C(C)C	8.6
22	O=C(N(C1CC[N+](CC1)CCc2ccccc2)c3ccccc3)C(C)=C	8.6
23	ClC(C(=O)N(C1CC[N+](CC1)CCc2ccccc2)c3ccccc3)=C	8.6
24	O=C(N(C1CC[N+](CC1)CCc2ccccc2)c3ccccc3)[C@@H](O)C	8.6
25	O=C(N(C1CC[N+](CC1)CCc2ccccc2)c3ccccc3)C(OC)=C	8.6
26	O=C(N(C1CC[N+](CC1)CCc2ccccc2)c3ccccc3)N/C=C\C	8.6
27	O=C(N(C1CC[N+](CC1)CCc2ccccc2)c3ccccc3)N4CC4	8.6
28	FC1CN(C(=O)N(C2CC[N+](CC2)CCc3ccccc3)c4ccccc4)C1	8.6
29	O=C(N(C1CC[N+](CC1)CCc2ccccc2)c3ccccc3)c4c[nH]cc4	8.6
30	O=C(N(C1CC[N+](CC1)CCc2ccccc2)c3ccccc3)C4(SC)CC4	8.6
31	O=C(N(C1CC[N+](CC1)CCc2ccccc2)c3ccccc3)/C=C/C=C/C	8.6
32	O=C(N(C1CC[N+](CC1)CCc2ccccc2)c3ccccc3)[C@@H]4C=CCO4	8.6
33	O=C(OC[C@H]1CO1)N(C2CC[N+](CC2)CCc3ccccc3)c4ccccc4	8.6
34	O=C(N(C1CC[N+](CC1)CCc2ccccc2)c3ccccc3)NCCO	8.6
35	O=C(N(C1CC[N+](CC1)CCc2ccccc2)c3ccccc3)[C@@H]4CC[C@@H]4C#N	8.6
36	O=C(N(C1CC[N+](CC1)CCc2ccccc2)c3ccccc3)[C@@H](OCC)C	8.6
37	O=C(N(C1CC[N+](CC1)CCc2ccccc2)c3ccccc3)CN#C	8.6
38	O=C(N(C1CC[N+](CC1)CCc2ccccc2)c3ccccc3)Cc4nocn4	8.6
39	O=C(N(C1CC[N+](CC1)CCc2ccccc2)c3ccccc3)C/C=C/CC	8.6
40	O=C(N(C1CC[N+](CC1)CCc2ccccc2)c3ccccc3)Cc4nc(on4)C	8.6
41	O=C(N(C1CC[N+](CC1)CCc2ccccc2)c3ccccc3)[C@@H](C)C(OC)=O	8.6
42	O=C(N(C1CC[N+](CC1)CCc2ccccc2)c3ccccc3)CCc4cocn4	8.6
43	O=C(N(C1CC[N+](CC1)CCc2ccccc2)c3ccccc3)/C=C/CO	8.6
44	O=C(N(C1CC[N+](CC1)CCc2ccccc2)c3ccccc3)Cc4nn(nn4)C	8.6
45	O=C(N(C1CC[N+](CC1)CCc2ccccc2)c3ccccc3)COc4ccon4	8.6
46	O=C(N(C1CC[N+](CC1)CCc2ccccc2)c3ccccc3)CC	8.5
47	O=C(N(C1CC[N+](CC1)CCc2ccccc2)c3ccccc3)C=C	8.5
48	O=C(N(C1CC[N+](CC1)CCc2ccccc2)c3ccccc3)/C=C=C	8.5
49	O=C(N(C1CC[N+](CC1)CCc2ccccc2)c3ccccc3)C4CC4	8.5
50	O=C(N(C1CC[N+](CC1)CCc2ccccc2)c3ccccc3)/C=C/C	8.5
51	F[C@H](C(=O)N(C1CC[N+](CC1)CCc2ccccc2)c3ccccc3)C	8.5
52	O=C(N(C1CC[N+](CC1)CCc2ccccc2)c3ccccc3)[C@@H]4CS4	8.5
53	Cl[C@@H](F)C(=O)N(C1CC[N+](CC1)CCc2ccccc2)c3ccccc3	8.5
54	O=C(N(C1CC[N+](CC1)CCc2ccccc2)c3ccccc3)C4=CC4	8.5
55	O=C(N(C1CC[N+](CC1)CCc2ccccc2)c3ccccc3)[C@H]4C[C@@H]4C	8.5
56	Cl[C@H](C(=O)N(C1CC[N+](CC1)CCc2ccccc2)c3ccccc3)C	8.5
57	O=C(N(C1CC[N+](CC1)CCc2ccccc2)c3ccccc3)N(C)C	8.5
58	O=C(N(C1CC[N+](CC1)CCc2ccccc2)c3ccccc3)C4=CCC4	8.5
59	O=C([S@@](=O)CC)N(C1CC[N+](CC1)CCc2ccccc2)c3ccccc3	8.5
60	O=C(N(C1CC[N+](CC1)CCc2ccccc2)c3ccccc3)CC[N+]([O-])=O	8.5
61	O=C(N(C1CC[N+](CC1)CCc2ccccc2)c3ccccc3)CCC#N	8.5

62	O=C(N(C1CC[N+](CC1)CCc2ccccc2)c3ccccc3)N(N)C	8.5
63	O=C(N(C1CC[N+](CC1)CCc2ccccc2)c3ccccc3)CSC4CC4	8.5
64	O=C(N(C1CC[N+](CC1)CCc2ccccc2)c3ccccc3)N(CC)C	8.5
65	F[C@H](OC)C(=O)N(C1CC[N+](CC1)CCc2ccccc2)c3ccccc3	8.5
66	O=C(N(C1CC[N+](CC1)CCc2ccccc2)c3ccccc3)C[C@H](OC)C	8.5
67	O=C(OCC[N+]([O-])=O)N(C1CC[N+](CC1)CCc2ccccc2)c3ccccc3	8.5
68	O=C(N(C1CC[N+](CC1)CCc2ccccc2)c3ccccc3)CC#N(C)C	8.5
69	O=C(OC(C)=C)N(C1CC[N+](CC1)CCc2ccccc2)c3ccccc3	8.5
70	O=C(N(C1CC[N+](CC1)CCc2ccccc2)c3ccccc3)Cc4cn[nH]c4	8.5
71	[O-][N+](=O)CCON(C1CC[N+](CC1)CCc2ccccc2)c3ccccc3	8.5
72	O=C(OCC#N)N(C1CC[N+](CC1)CCc2ccccc2)c3ccccc3	8.5
73	O=C(OCSC)N(C1CC[N+](CC1)CCc2ccccc2)c3ccccc3	8.5
74	O=C(OCCC#N)N(C1CC[N+](CC1)CCc2ccccc2)c3ccccc3	8.5
75	O=C(N(C1CC[N+](CC1)CCc2ccccc2)c3ccccc3)c4cncs4	8.5
76	OC1(CC1)C(=O)N(C2CC[N+](CC2)CCc3ccccc3)c4ccccc4	8.5
77	O=C(N(C1CC[N+](CC1)CCc2ccccc2)c3ccccc3)C4=COCC4	8.5
78	C#CCCON(C1CC[N+](CC1)CCc2ccccc2)c3ccccc3	8.5
79	FC(F)CS(=O)(=O)N(C1CC[N+](CC1)CCc2ccccc2)c3ccccc3	8.5
80	O=C(N(C1CC[N+](CC1)CCc2ccccc2)c3ccccc3)C4(CC4)CC	8.5
81	O=C(N(C1CC[N+](CC1)CCc2ccccc2)c3ccccc3)CN(OC)C	8.5
82	O=C(N(C1CC[N+](CC1)CCc2ccccc2)c3ccccc3)/C=C/C(C)C	8.5
83	O=C(N(C1CC[N+](CC1)CCc2ccccc2)c3ccccc3)/C=C\O	8.5
84	O=C(N(C1CC[N+](CC1)CCc2ccccc2)c3ccccc3)N4CSCC4	8.5
85	O=C(N(C1CC[N+](CC1)CCc2ccccc2)c3ccccc3)/C=C(\O)C	8.5
86	O[C@@H]1C[C@@H]1NC(=O)N(C2CC[N+](CC2)CCc3ccccc3)c4ccccc4	8.5
87	O=C(N(C1CC[N+](CC1)CCc2ccccc2)c3ccccc3)[C@@H]4CCCO4	8.5
88	FC(F)(CNC(=O)N(C1CC[N+](CC1)CCc2ccccc2)c3ccccc3)C	8.5
89	O=C(N(C1CC[N+](CC1)CCc2ccccc2)c3ccccc3)C(O)(C)C	8.5
90	Cl[C@H](/C=C/C(=O)N(C1CC[N+](CC1)CCc2ccccc2)c3ccccc3)C	8.5
91	O=C(N(C1CC[N+](CC1)CCc2ccccc2)c3ccccc3)CC#C	8.5
92	O=C(N(C1CC[N+](CC1)CCc2ccccc2)c3ccccc3)CC#CC	8.5
93	O=C(N(C1CC[N+](CC1)CCc2ccccc2)c3ccccc3)[C@H](C)C=C	8.5
94	O=C(N(C1CC[N+](CC1)CCc2ccccc2)c3ccccc3)CCC#CC	8.5
95	O=C(N(C1CC[N+](CC1)CCc2ccccc2)c3ccccc3)CCSC	8.5
96	O=C(N(C1CC[N+](CC1)CCc2ccccc2)c3ccccc3)/C=C/CC	8.5
97	FCCCCC(=O)N(C1CC[N+](CC1)CCc2ccccc2)c3ccccc3	8.5
98	O=C(N(C1CC[N+](CC1)CCc2ccccc2)c3ccccc3)CCCCC#C	8.5
99	O=C(N(C1CC[N+](CC1)CCc2ccccc2)c3ccccc3)CCB(O)O	8.5
100	O=C(N(C1CC[N+](CC1)CCc2ccccc2)c3ccccc3)CSCC=C	8.5
101	O=C(O/C=C/C)N(C1CC[N+](CC1)CCc2ccccc2)c3ccccc3	8.5
102	O=C(N(C1CC[N+](CC1)CCc2ccccc2)c3ccccc3)NCC#CC	8.5
103	O=C(N(C1CC[N+](CC1)CCc2ccccc2)c3ccccc3)CC=C(C)C	8.5

104	O=C(N1CCC1)N(C2CC[N+](CC2)CCc3ccccc3)c4ccccc4	8.5
105	O=C(N(C1CC[N+](CC1)CCc2ccccc2)c3ccccc3)[C@@H]4CCCS4	8.5
106	O=C(OCOC)N(C1CC[N+](CC1)CCc2ccccc2)c3ccccc3	8.5
107	O=C(N(C1CC[N+](CC1)CCc2ccccc2)c3ccccc3)NC/C=C/C	8.5
108	FC[C@H]1CC[C@H]1C(=O)N(C2CC[N+](CC2)CCc3ccccc3)c4ccccc4	8.5
109	FC(F)(C(=O)N(C1CC[N+](CC1)CCc2ccccc2)c3ccccc3)CO	8.5
110	O=C(N(C1CC[N+](CC1)CCc2ccccc2)c3ccccc3)[C@H](CC=C)C	8.5
111	O=C(N(C1CC[N+](CC1)CCc2ccccc2)c3ccccc3)c4cc(cs4)C	8.5
112	O=C(N(C1CC[N+](CC1)CCc2ccccc2)c3ccccc3)N(C=C)C=C	8.5
113	O=C(N(C1CC[N+](CC1)CCc2ccccc2)c3ccccc3)Nc4cscn4	8.5
114	O=C(N(C1CC[N+](CC1)CCc2ccccc2)c3ccccc3)CCCC(C)C	8.5
115	OC[C@@H](CC(=O)N(C1CC[N+](CC1)CCc2ccccc2)c3ccccc3)C	8.5
116	O=C(N(C1CC[N+](CC1)CCc2ccccc2)c3ccccc3)c4cnc(s4)O	8.5
117	FCCC(=O)N(C1CC[N+](CC1)CCc2ccccc2)c3ccccc3	8.4
118	O=C(SC)N(C1CC[N+](CC1)CCc2ccccc2)c3ccccc3	8.4
119	SCCC(=O)N(C1CC[N+](CC1)CCc2ccccc2)c3ccccc3	8.4
120	S[C@H](C(=O)N(C1CC[N+](CC1)CCc2ccccc2)c3ccccc3)C	8.4
121	O=C(N(C1CC[N+](CC1)CCc2ccccc2)c3ccccc3)NC#C	8.4
122	O=C(N(C1CC[N+](CC1)CCc2ccccc2)c3ccccc3)C=C4CC4	8.4
123	O=C(SCC)N(C1CC[N+](CC1)CCc2ccccc2)c3ccccc3	8.4
124	FCOC(=O)N(C1CC[N+](CC1)CCc2ccccc2)c3ccccc3	8.4
125	OC/C=N/N(C1CC[N+](CC1)CCc2ccccc2)c3ccccc3	8.4
126	O=C(N(C1CC[N+](CC1)CCc2ccccc2)c3ccccc3)C4CCC4	8.4
127	F[C@H](C(=O)N(C1CC[N+](CC1)CCc2ccccc2)c3ccccc3)CC	8.4
128	O=C(N(C1CC[N+](CC1)CCc2ccccc2)c3ccccc3)C4(CC4)C	8.4
129	O=C(OC)N(C1CC[N+](CC1)CCc2ccccc2)c3ccccc3	8.4
130	O=C(N(C1CC[N+](CC1)CCc2ccccc2)c3ccccc3)NC	8.4
131	FC1(CC1)C(=O)N(C2CC[N+](CC2)CCc3ccccc3)c4ccccc4	8.4
132	Cl[C@@H](CC(=O)N(C1CC[N+](CC1)CCc2ccccc2)c3ccccc3)C	8.4
133	O=C(N(C1CC[N+](CC1)CCc2ccccc2)c3ccccc3)CS([O-])(=O)=O	8.4
134	O=C(N(C1CC[N+](CC1)CCc2ccccc2)c3ccccc3)C[C@H]4C[C@H]4C	8.4
135	O=[S@@](N(C1CC[N+](CC1)CCc2ccccc2)c3ccccc3)CC	8.4
136	O=C(N(C1CC[N+](CC1)CCc2ccccc2)c3ccccc3)C4N=N4	8.4
137	Cl[C@H](C(=O)N(C1CC[N+](CC1)CCc2ccccc2)c3ccccc3)CC	8.4
138	O=S(=O)(N(C1CC[N+](CC1)CCc2ccccc2)c3ccccc3)CC	8.4
139	O=C(N(C1CC[N+](CC1)CCc2ccccc2)c3ccccc3)[C@@H](CC)C	8.4
140	O=C(N(C1CC[N+](CC1)CCc2ccccc2)c3ccccc3)[C@H]4C[C@@H]4CC	8.4
141	O=C(N(C1CC[N+](CC1)CCc2ccccc2)c3ccccc3)[C@H]4C=CCC4	8.4
142	O=C(N(C1CC[N+](CC1)CCc2ccccc2)c3ccccc3)NCCC	8.4
143	O=C(N(C1CC[N+](CC1)CCc2ccccc2)c3ccccc3)CN=N	8.4
144	S=C(N)CC(=O)N(C1CC[N+](CC1)CCc2ccccc2)c3ccccc3	8.4
145	O=C(N(C1CC[N+](CC1)CCc2ccccc2)c3ccccc3)[C@@H](CC#C)C	8.4

146	O=C(OCC)N(C1CC[N+](CC1)CCc2ccccc2)c3ccccc3	8.4
147	FC(F)(C(=O)N(C1CC[N+](CC1)CCc2ccccc2)c3ccccc3)C	8.4
148	FC(C(=O)N(C1CC[N+](CC1)CCc2ccccc2)c3ccccc3)(C)C	8.4
149	CCON(C1CC[N+](CC1)CCc2ccccc2)c3ccccc3	8.4
150	S[C@H](C(=O)N(C1CC[N+](CC1)CCc2ccccc2)c3ccccc3)CC	8.4
151	O=C(N(C1CC[N+](CC1)CCc2ccccc2)c3ccccc3)CC/C=C/C	8.4
152	O=C(N(C1CC[N+](CC1)CCc2ccccc2)c3ccccc3)C(CCC)=C	8.4
153	O=C(N(C1CC[N+](CC1)CCc2ccccc2)c3ccccc3)/C=C/CCC	8.4
154	O=C(N(C1CC[N+](CC1)CCc2ccccc2)c3ccccc3)NNCC	8.4
155	O=C(N(C1CC[N+](CC1)CCc2ccccc2)c3ccccc3)C[S@@](=O)CC	8.4
156	FC1(CCC1)C(=O)N(C2CC[N+](CC2)CCc3ccccc3)c4ccccc4	8.4
157	O=C(N(C1CC[N+](CC1)CCc2ccccc2)c3ccccc3)CCCOC	8.4
158	FC(F)(F)CCC(=O)N(C1CC[N+](CC1)CCc2ccccc2)c3ccccc3	8.4
159	O=S(=O)(N(C1CC[N+](CC1)CCc2ccccc2)c3ccccc3)C	8.4
160	O=C(N(C1CC[N+](CC1)CCc2ccccc2)c3ccccc3)C4CC=CC4	8.4
161	O=C(N(C1CC[N+](CC1)CCc2ccccc2)c3ccccc3)c4cocc4	8.4
162	Cl[C@@H](C(=O)N(C1CC[N+](CC1)CCc2ccccc2)c3ccccc3)CO	8.4
163	O=C(N(C1CC[N+](CC1)CCc2ccccc2)c3ccccc3)C4(CCC4)C	8.4
164	O=C(N(C1CC[N+](CC1)CCc2ccccc2)c3ccccc3)/C(=C/C)C	8.4
165	OC(N(C1CC[N+](CC1)CCc2ccccc2)c3ccccc3)C[N+]([O-])O	8.4
166	Cl[C@@H](C(C)C)C(=O)N(C1CC[N+](CC1)CCc2ccccc2)c3ccccc3	8.4
167	O=C(N(C1CC[N+](CC1)CCc2ccccc2)c3ccccc3)N[C@H]4CO4	8.4
168	O=C(N(C1CC[N+](CC1)CCc2ccccc2)c3ccccc3)NC[C@H]4CO4	8.4
169	ClC(C(=O)N(C1CC[N+](CC1)CCc2ccccc2)c3ccccc3)(C)C	8.4
170	O=C(N(C1CC[N+](CC1)CCc2ccccc2)c3ccccc3)C(CC)CC	8.4
171	O=C(N(C1CC[N+](CC1)CCc2ccccc2)c3ccccc3)[C@@H]4[C@H](O4)C	8.4
172	O=C(N(C1CC[N+](CC1)CCc2ccccc2)c3ccccc3)[C@@H](CCC)C	8.4
173	Cl[C@@H](C(=O)N(C1CC[N+](CC1)CCc2ccccc2)c3ccccc3)CCC	8.4
174	F[C@@H]1C[C@@H](CC1)C(=O)N(C2CC[N+](CC2)CCc3ccccc3)c4ccccc4	8.4
175	O=C(N(C1CC[N+](CC1)CCc2ccccc2)c3ccccc3)C[C@H](C)C#N	8.4
176	C=CON(C1CC[N+](CC1)CCc2ccccc2)c3ccccc3	8.4
177	O(N(C1CC[N+](CC1)CCc2ccccc2)c3ccccc3)C4CC4	8.4
178	O=C(N(C1CC[N+](CC1)CCc2ccccc2)c3ccccc3)CN(C)C#N	8.4
179	FC(F)(F)CNC(=O)N(C1CC[N+](CC1)CCc2ccccc2)c3ccccc3	8.4
180	FC(SC)(F)C(=O)N(C1CC[N+](CC1)CCc2ccccc2)c3ccccc3	8.4
181	O=C(N(C1CC[N+](CC1)CCc2ccccc2)c3ccccc3)C(CO)=C	8.4
182	O=C(N(C1CC[N+](CC1)CCc2ccccc2)c3ccccc3)c4ccc(s4)C	8.4
183	O=C(N(C1CC[N+](CC1)CCc2ccccc2)c3ccccc3)C(C4CC4)=C	8.4
184	O=C(N(C1CC[N+](CC1)CCc2ccccc2)c3ccccc3)/C(O)=C/C	8.4
185	O=C(N(C1CC[N+](CC1)CCc2ccccc2)c3ccccc3)/C(CC)=C/C	8.4
186	S[C@H](C(=O)N(C1CC[N+](CC1)CCc2ccccc2)c3ccccc3)CCC	8.4
187	O[C@@]1(N(C2CC[N+](CC2)CCc3ccccc3)c4ccccc4)COCCC1	8.4

188	O=C(N(C1CC[N+](CC1)CCc2ccccc2)c3ccccc3)[C@H]4[C@H](CCO4)C	8.4
189	O=C(N(C1CC[N+](CC1)CCc2ccccc2)c3ccccc3)C4=CCCC4	8.4
190	O=C(N(C1CC[N+](CC1)CCc2ccccc2)c3ccccc3)CCC#C	8.4
191	O=C(N(C1CC[N+](CC1)CCc2ccccc2)c3ccccc3)CCCC	8.4
192	O=C(SCC#C)N(C1CC[N+](CC1)CCc2ccccc2)c3ccccc3	8.4
193	O=C(N(C1CC[N+](CC1)CCc2ccccc2)c3ccccc3)CCC4CC4	8.4
194	O=C(N(C1CC[N+](CC1)CCc2ccccc2)c3ccccc3)CCCCC	8.4
195	O=C(N(C1CC[N+](CC1)CCc2ccccc2)c3ccccc3)NCC=C	8.4
196	O=C(N(C1CC[N+](CC1)CCc2ccccc2)c3ccccc3)CCC(C)C	8.4
197	C#CCON(C1CC[N+](CC1)CCc2ccccc2)c3ccccc3	8.4
198	O=C(N(C1CC[N+](CC1)CCc2ccccc2)c3ccccc3)[C@@H](C)C#CC	8.4
199	O=C(N(C1CC[N+](CC1)CCc2ccccc2)c3ccccc3)/C=C/4C[C@@H]4C	8.4
200	O=C(N(C1CC[N+](CC1)CCc2ccccc2)c3ccccc3)/C=C/C4CC4	8.4
201	O=C(OC1CC1)N(C2CC[N+](CC2)CCc3ccccc3)c4ccccc4	8.4
202	ClC(Cl)(C(=O)N(C1CC[N+](CC1)CCc2ccccc2)c3ccccc3)C	8.4
203	O=C(N(C1CC[N+](CC1)CCc2ccccc2)c3ccccc3)C[C@H](SC)C	8.4
204	Cl[C@@H](CCC(=O)N(C1CC[N+](CC1)CCc2ccccc2)c3ccccc3)C	8.4
205	O=C(N(C1CC[N+](CC1)CCc2ccccc2)c3ccccc3)CCC4CCC4	8.4
206	O=C(N(C1CC[N+](CC1)CCc2ccccc2)c3ccccc3)CCCC=C	8.4
207	SC/C=C/CC(=O)N(C1CC[N+](CC1)CCc2ccccc2)c3ccccc3	8.4
208	O=C(N(C1CC[N+](CC1)CCc2ccccc2)c3ccccc3)C4CC5(CC5)C4	8.4
209	O=C(N(C1CC[N+](CC1)CCc2ccccc2)c3ccccc3)CCC(C)=C	8.4
210	O=C(N(C1CC[N+](CC1)CCc2ccccc2)c3ccccc3)CCCCC=C	8.4
211	O=C(N(C1CC[N+](CC1)CCc2ccccc2)c3ccccc3)N(CC#C)C	8.4
212	O=C(N(C1CC[N+](CC1)CCc2ccccc2)c3ccccc3)c4cscc4	8.4
213	O=C(N(C1CC[N+](CC1)CCc2ccccc2)c3ccccc3)CCCCCC	8.4
214	O=C(Oc1ccco1)N(C2CC[N+](CC2)CCc3ccccc3)c4ccccc4	8.4
215	O=C(OCC=C)N(C1CC[N+](CC1)CCc2ccccc2)c3ccccc3	8.4
216	O=C(N(C1CC[N+](CC1)CCc2ccccc2)c3ccccc3)c4ccsn4	8.4
217	O=C(N(C1CC[N+](CC1)CCc2ccccc2)c3ccccc3)CCC	8.3
218	FCC(=O)N(C1CC[N+](CC1)CCc2ccccc2)c3ccccc3	8.3
219	O=C(N(C1CC[N+](CC1)CCc2ccccc2)c3ccccc3)C	8.3
220	O=C(N(C1CC[N+](CC1)CCc2ccccc2)c3ccccc3)CC=C	8.3
221	SC(=O)N(C1CC[N+](CC1)CCc2ccccc2)c3ccccc3	8.3
222	FCSC(=O)N(C1CC[N+](CC1)CCc2ccccc2)c3ccccc3	8.3
223	O=C(N(C1CC[N+](CC1)CCc2ccccc2)c3ccccc3)CC4CC4	8.3
224	O=C(N(C1CC[N+](CC1)CCc2ccccc2)c3ccccc3)CC(C)C	8.3
225	ClC(Cl)C(=O)N(C1CC[N+](CC1)CCc2ccccc2)c3ccccc3	8.3
226	O=C(N(C1CC[N+](CC1)CCc2ccccc2)c3ccccc3)CCC=C	8.3
227	O=C(N(C1CC[N+](CC1)CCc2ccccc2)c3ccccc3)C#C	8.3
228	FC(F)OC(=O)N(C1CC[N+](CC1)CCc2ccccc2)c3ccccc3	8.3
229	FC(F)C(=O)N(C1CC[N+](CC1)CCc2ccccc2)c3ccccc3	8.3

230	F[C@H]1C[C@@H]1C(=O)N(C2CC[N+](CC2)CCc3ccccc3)c4ccccc4	8.3
231	[O-][N+](N(C1CC[N+](CC1)CCc2ccccc2)c3ccccc3)C	8.3
232	O=C(N(C1CC[N+](CC1)CCc2ccccc2)c3ccccc3)C4CC(C4)C	8.3
233	FCCON(C1CC[N+](CC1)CCc2ccccc2)c3ccccc3	8.3
234	O=C(N(C1CC[N+](CC1)CCc2ccccc2)c3ccccc3)C(CC)=C	8.3
235	O=C(N(C1CC[N+](CC1)CCc2ccccc2)c3ccccc3)NC=C	8.3
236	FC(C(=O)N(C1CC[N+](CC1)CCc2ccccc2)c3ccccc3)=C	8.3
237	O=C(N(C1CC[N+](CC1)CCc2ccccc2)c3ccccc3)NCC#C	8.3
238	O=C(N(C1CC[N+](CC1)CCc2ccccc2)c3ccccc3)C[C@H](CC)C	8.3
239	O=C(N(C1CC[N+](CC1)CCc2ccccc2)c3ccccc3)[C@@H]4CC[C@@H]4C	8.3
240	CCCON(C1CC[N+](CC1)CCc2ccccc2)c3ccccc3	8.3
241	S=C(N)C(=O)N(C1CC[N+](CC1)CCc2ccccc2)c3ccccc3	8.3
242	ClC(F)(F)C(=O)N(C1CC[N+](CC1)CCc2ccccc2)c3ccccc3	8.3
243	ClC(Cl)(F)C(=O)N(C1CC[N+](CC1)CCc2ccccc2)c3ccccc3	8.3
244	O=C(N(C1CC[N+](CC1)CCc2ccccc2)c3ccccc3)C#CC	8.3
245	O=C(N(C1CC[N+](CC1)CCc2ccccc2)c3ccccc3)[C@@H]4CC54CC5	8.3
246	O=C(N(C1CC[N+](CC1)CCc2ccccc2)c3ccccc3)/C(C)=C/O	8.3
247	O=C(SCCC)N(C1CC[N+](CC1)CCc2ccccc2)c3ccccc3	8.3
248	O=C(N(C1CC[N+](CC1)CCc2ccccc2)c3ccccc3)[C@H](SC)C	8.3
249	F[C@H]1C[C@]1(C(=O)N(C2CC[N+](CC2)CCc3ccccc3)c4ccccc4)C	8.3
250	FC1(F)C[C@@H]1CC(=O)N(C2CC[N+](CC2)CCc3ccccc3)c4ccccc4	8.3
251	O=C(N(C1CC[N+](CC1)CCc2ccccc2)c3ccccc3)[C@H](C4CC4)C	8.3
252	O=C(N(C1CC[N+](CC1)CCc2ccccc2)c3ccccc3)C(C)(C)C	8.3
253	F[C@@H](C(C)C)C(=O)N(C1CC[N+](CC1)CCc2ccccc2)c3ccccc3	8.3
254	O=C(N(C1CC[N+](CC1)CCc2ccccc2)c3ccccc3)C(C(C)C)=C	8.3
255	CSCON(C1CC[N+](CC1)CCc2ccccc2)c3ccccc3	8.3
256	O=S(=O)(N(C1CC[N+](CC1)CCc2ccccc2)c3ccccc3)CCC	8.3
257	Fc1csc(C(=O)N(C2CC[N+](CC2)CCc3ccccc3)c4ccccc4)c1	8.3
258	O=C(N(C1CC[N+](CC1)CCc2ccccc2)c3ccccc3)[C@@]4(C[C@@H]4C)C	8.3
259	SCCON(C1CC[N+](CC1)CCc2ccccc2)c3ccccc3	8.3
260	O=C(N(C1CC[N+](CC1)CCc2ccccc2)c3ccccc3)CCC4(N=N4)C	8.3
261	O=C(N(C1CC[N+](CC1)CCc2ccccc2)c3ccccc3)N[C@H]4C[C@H]4C	8.3
262	O[C@@H](N(C1CC[N+](CC1)CCc2ccccc2)c3ccccc3)CSC	8.3
263	O=C(OC(C)C)N(C1CC[N+](CC1)CCc2ccccc2)c3ccccc3	8.3
264	O=C(N(C1CC[N+](CC1)CCc2ccccc2)c3ccccc3)CCCC#CC	8.3
265	O=C(N(C1CC[N+](CC1)CCc2ccccc2)c3ccccc3)CC4(CCC4)C	8.3
266	Fc1ccc(s1)C(=O)N(C2CC[N+](CC2)CCc3ccccc3)c4ccccc4	8.3
267	O=C(N(C1CC[N+](CC1)CCc2ccccc2)c3ccccc3)[C@@H]4CC4(C)C	8.3
268	O=C(N(C1CC[N+](CC1)CCc2ccccc2)c3ccccc3)C[C@H](C4CC4)C	8.3
269	FCCN(C(=O)N(C1CC[N+](CC1)CCc2ccccc2)c3ccccc3)C	8.3
270	FC(F)(C(=O)N(C1CC[N+](CC1)CCc2ccccc2)c3ccccc3)CC	8.3
271	O=C(N(C1CC[N+](CC1)CCc2ccccc2)c3ccccc3)C4[C@@H]([C@@H]4C)C	8.3

272	O=C(N(C1CC[N+](CC1)CCc2ccccc2)c3ccccc3)[C@H]4C[C@@H](CC4)C	8.3
273	CC[C@H](OC)N(C1CC[N+](CC1)CCc2ccccc2)c3ccccc3	8.3
274	COCCON(C1CC[N+](CC1)CCc2ccccc2)c3ccccc3	8.3
275	O=C(N(C1CC[N+](CC1)CCc2ccccc2)c3ccccc3)NCCC#C	8.3
276	O=C(N(C1CC[N+](CC1)CCc2ccccc2)c3ccccc3)[C@H]4CCC[C@@H]4C	8.3
277	FC(F)(CCC(=O)N(C1CC[N+](CC1)CCc2ccccc2)c3ccccc3)C	8.3
278	O=C(N(C1CC[N+](CC1)CCc2ccccc2)c3ccccc3)NC(C)=C	8.3
279	O=C(N(C1CC[N+](CC1)CCc2ccccc2)c3ccccc3)/C(=[N+]=[N-])C	8.3
280	O=C(N(C1CC[N+](CC1)CCc2ccccc2)c3ccccc3)NCOC	8.3
281	Fc1ccoc1C(=O)N(C2CC[N+](CC2)CCc3ccccc3)c4ccccc4	8.3
282	O=C(OCCC)N(C1CC[N+](CC1)CCc2ccccc2)c3ccccc3	8.3
283	O=C(N(C1CC[N+](CC1)CCc2ccccc2)c3ccccc3)COCC	8.3
284	FCCCOC(=O)N(C1CC[N+](CC1)CCc2ccccc2)c3ccccc3	8.3
285	O=C(N(C1CC[N+](CC1)CCc2ccccc2)c3ccccc3)NCCCC#C	8.3
286	O=C(N(C1CC[N+](CC1)CCc2ccccc2)c3ccccc3)N	8.3
287	O=C(N(C1CC[N+](CC1)CCc2ccccc2)c3ccccc3)[C@@H]4C[C@@H]([C@@H]4C)C	8.3
288	CC(CON(C1CC[N+](CC1)CCc2ccccc2)c3ccccc3)C	8.3
289	O=C(N(C1CC[N+](CC1)CCc2ccccc2)c3ccccc3)[C@@H]4CSCC4	8.3
290	O=C(N(C1CC[N+](CC1)CCc2ccccc2)c3ccccc3)c4cnc(o4)O	8.3
291	O=C(N(C1CC[N+](CC1)CCc2ccccc2)c3ccccc3)COCC=C	8.3
292	O=C(N(C1CC[N+](CC1)CCc2ccccc2)c3ccccc3)c4ccns4	8.3
293	O=[S@@](N(C1CC[N+](CC1)CCc2ccccc2)c3ccccc3)C=C	8.3
294	Cl/C=C/C(=O)N(C1CC[N+](CC1)CCc2ccccc2)c3ccccc3	8.3
295	O=C(N(C1CC[N+](CC1)CCc2ccccc2)c3ccccc3)C/C=C/C	8.3
296	O=C(N(C1CC[N+](CC1)CCc2ccccc2)c3ccccc3)CCCC#C	8.3
297	O=C(N(C1CC[N+](CC1)CCc2ccccc2)c3ccccc3)CC4CCC4	8.3
298	O=C(N(C1CC[N+](CC1)CCc2ccccc2)c3ccccc3)CSCC#C	8.3
299	O=C(N(C1CC[N+](CC1)CCc2ccccc2)c3ccccc3)[C@H]4C[C@@H]4C#C	8.3
300	O=S(=O)(N(C1CC[N+](CC1)CCc2ccccc2)c3ccccc3)CC#C	8.3
301	O=C(N(C1CC[N+](CC1)CCc2ccccc2)c3ccccc3)C4CCCC4	8.3
302	O=C(N(C1CC[N+](CC1)CCc2ccccc2)c3ccccc3)NC(C)C	8.3
303	FC(F)ON(C1CC[N+](CC1)CCc2ccccc2)c3ccccc3	8.3
304	O=C(N(C1CC[N+](CC1)CCc2ccccc2)c3ccccc3)Cc4cocn4	8.3
305	O=C(N(C1CC[N+](CC1)CCc2ccccc2)c3ccccc3)[C@H](C(C)C)C	8.3
306	O=C(N(C1CC[N+](CC1)CCc2ccccc2)c3ccccc3)/C=C/SC	8.3
307	O=C(N(C1CC[N+](CC1)CCc2ccccc2)c3ccccc3)[C@@H](CCC)C=C	8.3
308	SC(C(=O)N(C1CC[N+](CC1)CCc2ccccc2)c3ccccc3)(C)C	8.3
309	O=C(N(C1CC[N+](CC1)CCc2ccccc2)c3ccccc3)CC(CC)CC	8.3
310	O=C(N(C1CC[N+](CC1)CCc2ccccc2)c3ccccc3)C4[C@H]5CCC[C@@H]45	8.3
311	O=C(N(C1CC[N+](CC1)CCc2ccccc2)c3ccccc3)CCC4(CC4)C	8.3
312	SCCCCC(=O)N(C1CC[N+](CC1)CCc2ccccc2)c3ccccc3	8.3
313	O=C(N(C1CC[N+](CC1)CCc2ccccc2)c3ccccc3)CCCC4CC4	8.3

314	O=C(N(C1CC[N+](CC1)CCc2ccccc2)c3ccccc3)Cc4cscn4	8.3
315	O=C(N(C1CC[N+](CC1)CCc2ccccc2)c3ccccc3)Cc4nccs4	8.3
316	O=C(N(C1CC[N+](CC1)CCc2ccccc2)c3ccccc3)C(/C=C\C)=C	8.3
317	S[C@@H](CCC(=O)N(C1CC[N+](CC1)CCc2ccccc2)c3ccccc3)C	8.3
318	O=C(N(C1CC[N+](CC1)CCc2ccccc2)c3ccccc3)[C@@H]4C=CCCC4	8.3
319	O=C(N(C1CC[N+](CC1)CCc2ccccc2)c3ccccc3)CCCSC	8.3
320	Fc1ncc(s1)C(=O)N(C2CC[N+](CC2)CCc3ccccc3)c4ccccc4	8.3
321	O=S(=O)(N(C1CC[N+](CC1)CCc2ccccc2)c3ccccc3)CCC=C	8.3
322	FC(F)(F)[C@H](C(=O)N(C1CC[N+](CC1)CCc2ccccc2)c3ccccc3)C	8.3
323	O=C(N(C1CC[N+](CC1)CCc2ccccc2)c3ccccc3)C(C)(C)C=C	8.3
324	O=S(=O)(N(C1CC[N+](CC1)CCc2ccccc2)c3ccccc3)CC(C)C	8.3
325	O=C(N(C1CC[N+](CC1)CCc2ccccc2)c3ccccc3)Cc4coc(n4)C	8.3
326	O=C(OCCC#C)N(C1CC[N+](CC1)CCc2ccccc2)c3ccccc3	8.3
327	O=C(N(C1CC[N+](CC1)CCc2ccccc2)c3ccccc3)C(CC)(C)C	8.3
328	FC1(F)C[C@]1(C(=O)N(C2CC[N+](CC2)CCc3ccccc3)c4ccccc4)C	8.3
329	O=C(N(C1CC[N+](CC1)CCc2ccccc2)c3ccccc3)C4CC(C4)CC	8.3
330	O=C(N(C1CC[N+](CC1)CCc2ccccc2)c3ccccc3)NC4CCC4	8.3
331	O=C(N(C1CC[N+](CC1)CCc2ccccc2)c3ccccc3)[C@]45CCC[C@@H]5C4	8.3
332	O[C@H](CC(=O)N(C1CC[N+](CC1)CCc2ccccc2)c3ccccc3)CC	8.3
333	O=C(OCC#C)N(C1CC[N+](CC1)CCc2ccccc2)c3ccccc3	8.3
334	O=C(N(C1CC[N+](CC1)CCc2ccccc2)c3ccccc3)CC(O)(C)C	8.3
335	FC(F)[C@@H]1C[C@H]1C(=O)N(C2CC[N+](CC2)CCc3ccccc3)c4ccccc4	8.3
336	OC/C=C/N(C1CC[N+](CC1)CCc2ccccc2)c3ccccc3	8.3
337	SCC(=O)N(C1CC[N+](CC1)CCc2ccccc2)c3ccccc3	8.2
338	O=C(N(C1CC[N+](CC1)CCc2ccccc2)c3ccccc3)NCC	8.2
339	FCCCC(=O)N(C1CC[N+](CC1)CCc2ccccc2)c3ccccc3	8.2
340	FCON(C1CC[N+](CC1)CCc2ccccc2)c3ccccc3	8.2
341	FC(F)CON(C1CC[N+](CC1)CCc2ccccc2)c3ccccc3	8.2
342	O=C(N(C1CC[N+](CC1)CCc2ccccc2)c3ccccc3)NCO	8.2
343	O=C(N(C1CC[N+](CC1)CCc2ccccc2)c3ccccc3)CC4(CC4)C	8.2
344	FC(F)(F)C(=O)N(C1CC[N+](CC1)CCc2ccccc2)c3ccccc3	8.2
345	O=C(N(C1CC[N+](CC1)CCc2ccccc2)c3ccccc3)/C=C/C=C	8.2
346	FC1CC(C1)C(=O)N(C2CC[N+](CC2)CCc3ccccc3)c4ccccc4	8.2
347	O=C(N(C1CC[N+](CC1)CCc2ccccc2)c3ccccc3)NC4CC4	8.2
348	O=C(N(C1CC[N+](CC1)CCc2ccccc2)c3ccccc3)CCO[N+]([O-])=O	8.2
349	O=C(SC(C)C)N(C1CC[N+](CC1)CCc2ccccc2)c3ccccc3	8.2
350	O=C(OCC(=O)N)N(C1CC[N+](CC1)CCc2ccccc2)c3ccccc3	8.2
351	O=C(N(C1CC[N+](CC1)CCc2ccccc2)c3ccccc3)CS(=O)(=O)C	8.2
352	FC(F)(C(=O)N(C1CC[N+](CC1)CCc2ccccc2)c3ccccc3)C(F)F	8.2
353	O=C(N(C1CC[N+](CC1)CCc2ccccc2)c3ccccc3)C4(CC4)C#C	8.2
354	SCCNC(=O)N(C1CC[N+](CC1)CCc2ccccc2)c3ccccc3	8.2
355	O=C(N(C1CC[N+](CC1)CCc2ccccc2)c3ccccc3)NOC	8.2

356	O=[S@@](N(C1CC[N+](CC1)CCc2ccccc2)c3ccccc3)C	8.2
357	O=C(N(C1CC[N+](CC1)CCc2ccccc2)c3ccccc3)CC4COC4	8.2
358	FC(F)(F)OCC(=O)N(C1CC[N+](CC1)CCc2ccccc2)c3ccccc3	8.2
359	O=C(N(C1CC[N+](CC1)CCc2ccccc2)c3ccccc3)C4(CC4)C=C	8.2
360	O=C(N(C1CC[N+](CC1)CCc2ccccc2)c3ccccc3)NS(=O)(=O)C	8.2
361	ClC1CC(C1)C(=O)N(C2CC[N+](CC2)CCc3ccccc3)c4ccccc4	8.2
362	Cl[C@H](OC(=O)N(C1CC[N+](CC1)CCc2ccccc2)c3ccccc3)C	8.2
363	O=C(N(C1CC[N+](CC1)CCc2ccccc2)c3ccccc3)CC4CCCC4	8.2
364	FCCS(=O)(=O)N(C1CC[N+](CC1)CCc2ccccc2)c3ccccc3	8.2
365	[O-]/[N+](N(C1CC[N+](CC1)CCc2ccccc2)c3ccccc3)=C\c4ccco4	8.2
366	O=C(N(C1CC[N+](CC1)CCc2ccccc2)c3ccccc3)[C@@H]4C[C@@H]4C5CC5	8.2
367	FC(F)COC(=O)N(C1CC[N+](CC1)CCc2ccccc2)c3ccccc3	8.2
368	SC[C@@H](C(=O)N(C1CC[N+](CC1)CCc2ccccc2)c3ccccc3)C	8.2
369	O=C(OCCO)N(C1CC[N+](CC1)CCc2ccccc2)c3ccccc3	8.2
370	O=C(N(C1CC[N+](CC1)CCc2ccccc2)c3ccccc3)NC4(CC4)C	8.2
371	O=C(N(C1CC[N+](CC1)CCc2ccccc2)c3ccccc3)C[C@@H]4C=CCC4	8.2
372	O=C(N(C1CC[N+](CC1)CCc2ccccc2)c3ccccc3)N[C@H](O)C	8.2
373	CO/N=C/N(C1CC[N+](CC1)CCc2ccccc2)c3ccccc3	8.2
374	O=C(N(C1CC[N+](CC1)CCc2ccccc2)c3ccccc3)CC(C)(C)C=C	8.2
375	FC(CON(C1CC[N+](CC1)CCc2ccccc2)c3ccccc3)(C)C	8.2
376	S=NN(C1CC[N+](CC1)CCc2ccccc2)c3ccccc3	8.2
377	[N+]1(CCC(N(c2ccccc2)/C=C/c3ncc[nH]3)CC1)CCc4ccccc4	8.2
378	FCC(=O)C(=O)N(C1CC[N+](CC1)CCc2ccccc2)c3ccccc3	8.2
379	O=C(N(C1CC[N+](CC1)CCc2ccccc2)c3ccccc3)C4C[C@H]5C[C@H]5C4	8.2
380	O=[S@](N(C1CC[N+](CC1)CCc2ccccc2)c3ccccc3)CCCC	8.2
381	NO[C@H](N(C1CC[N+](CC1)CCc2ccccc2)c3ccccc3)C	8.2
382	S[C@@H](C(C)C)C(=O)N(C1CC[N+](CC1)CCc2ccccc2)c3ccccc3	8.2
383	C#CON(C1CC[N+](CC1)CCc2ccccc2)c3ccccc3	8.2
384	O=C(N(C1CC[N+](CC1)CCc2ccccc2)c3ccccc3)CC(C)(C)C#N	8.2
385	O=C(N(C1CC[N+](CC1)CCc2ccccc2)c3ccccc3)[C@@H](SC)CC	8.2
386	CCO/N=C/N(C1CC[N+](CC1)CCc2ccccc2)c3ccccc3	8.2
387	O=C(N(C1CC[N+](CC1)CCc2ccccc2)c3ccccc3)C(C)(C)C#C	8.2
388	O=C(N(C1CC[N+](CC1)CCc2ccccc2)c3ccccc3)[C@H](OC)CC	8.2
389	Cl/C(=C\C(=O)N(C1CC[N+](CC1)CCc2ccccc2)c3ccccc3)C	8.2
390	FC(CON(C1CC[N+](CC1)CCc2ccccc2)c3ccccc3)=C	8.2
391	O=C(N(C1CC[N+](CC1)CCc2ccccc2)c3ccccc3)CSCCC	8.2
392	O=C(SCC=C)N(C1CC[N+](CC1)CCc2ccccc2)c3ccccc3	8.2
393	F/C(C(=O)N(C1CC[N+](CC1)CCc2ccccc2)c3ccccc3)=C\OC	8.2
394	O=C(N(C1CC[N+](CC1)CCc2ccccc2)c3ccccc3)Cc4ccco4	8.2
395	O=C(N(C1CC[N+](CC1)CCc2ccccc2)c3ccccc3)[C@H]4CCC=CO4	8.2
396	O=C(N(C1CC[N+](CC1)CCc2ccccc2)c3ccccc3)C[C@H](CCC)C	8.2
397	O=[S@@](N(C1CC[N+](CC1)CCc2ccccc2)c3ccccc3)CC(C)C	8.2

398	Fc1cscc1C(=O)N(C2CC[N+](CC2)CCc3ccccc3)c4ccccc4	8.2
399	O=C(N(C1CC[N+](CC1)CCc2ccccc2)c3ccccc3)CC4=CCCC4	8.2
400	O=C(N(C1CC[N+](CC1)CCc2ccccc2)c3ccccc3)N[C@H](C)C#C	8.2
401	FC(F)CCCC(=O)N(C1CC[N+](CC1)CCc2ccccc2)c3ccccc3	8.2
402	O=C(N(C1CC[N+](CC1)CCc2ccccc2)c3ccccc3)Cc4c(O)non4	8.2
403	O=C(N(C1CC[N+](CC1)CCc2ccccc2)c3ccccc3)[C@H](C)C#N	8.2
404	O=C(N(C1CC[N+](CC1)CCc2ccccc2)c3ccccc3)CSC	8.1
405	O=C(N(C1CC[N+](CC1)CCc2ccccc2)c3ccccc3)COC	8.1
406	FC[C@H](O)N(C1CC[N+](CC1)CCc2ccccc2)c3ccccc3	8.1
407	FC(F)(F)NC(=O)N(C1CC[N+](CC1)CCc2ccccc2)c3ccccc3	8.1
408	O=C(N(C1CC[N+](CC1)CCc2ccccc2)c3ccccc3)C4CSC4	8.1
409	O=C(N(C1CC[N+](CC1)CCc2ccccc2)c3ccccc3)C=C4CCC4	8.1
410	O=C(N(C1CC[N+](CC1)CCc2ccccc2)c3ccccc3)CSSC	8.1
411	FCCNC(=O)N(C1CC[N+](CC1)CCc2ccccc2)c3ccccc3	8.1
412	O=C(N(C1CC[N+](CC1)CCc2ccccc2)c3ccccc3)CC(C)(C)C	8.1
413	F[C@H]1CC[C@@H]1C(=O)N(C2CC[N+](CC2)CCc3ccccc3)c4ccccc4	8.1
414	FCC1(CC1)C(=O)N(C2CC[N+](CC2)CCc3ccccc3)c4ccccc4	8.1
415	FC(F)CCC(=O)N(C1CC[N+](CC1)CCc2ccccc2)c3ccccc3	8.1
416	FCCOC(=O)N(C1CC[N+](CC1)CCc2ccccc2)c3ccccc3	8.1
417	O=S(=O)(CON(C1CC[N+](CC1)CCc2ccccc2)c3ccccc3)C	8.1
418	O=C(N(C1CC[N+](CC1)CCc2ccccc2)c3ccccc3)CSC(C)C	8.1
419	O=C(N(C1CC[N+](CC1)CCc2ccccc2)c3ccccc3)NCC4CC4	8.1
420	O=C(N)[C@@H](N(C1CC[N+](CC1)CCc2ccccc2)c3ccccc3)CC	8.1
421	O=C(N(C1CC[N+](CC1)CCc2ccccc2)c3ccccc3)CO	8.1
422	O=C(N(C1CC[N+](CC1)CCc2ccccc2)c3ccccc3)/C=C/OC	8.1
423	O1CC(CON(C2CC[N+](CC2)CCc3ccccc3)c4ccccc4)C1	8.1
424	O=C(N(C1CC[N+](CC1)CCc2ccccc2)c3ccccc3)Cc4ccn[nH]4	8.1
425	FC1(F)CC[C@@H]1C(=O)N(C2CC[N+](CC2)CCc3ccccc3)c4ccccc4	8.1
426	O=C(O[C@H](C)C#N)N(C1CC[N+](CC1)CCc2ccccc2)c3ccccc3	8.1
427	O=C(N(C1CC[N+](CC1)CCc2ccccc2)c3ccccc3)NOCC	8.1
428	O=C(N(C1CC[N+](CC1)CCc2ccccc2)c3ccccc3)CCSCC	8.1
429	O=C(N(C1CC[N+](CC1)CCc2ccccc2)c3ccccc3)CCCON	8.1
430	FC(F)(CON(C1CC[N+](CC1)CCc2ccccc2)c3ccccc3)C	8.1
431	O=C(N(C1CC[N+](CC1)CCc2ccccc2)c3ccccc3)NCC(C)C	8.1
432	FC[C@H](OC(=O)N(C1CC[N+](CC1)CCc2ccccc2)c3ccccc3)C	8.1
433	OC[C@@H](OC(=O)N(C1CC[N+](CC1)CCc2ccccc2)c3ccccc3)C	8.1
434	Cl[C@@H]([C@H](O)N(C1CC[N+](CC1)CCc2ccccc2)c3ccccc3)C	8.1
435	FC(F)(F)C(=O)C(=O)N(C1CC[N+](CC1)CCc2ccccc2)c3ccccc3	8.1
436	O=C(N(C1CC[N+](CC1)CCc2ccccc2)c3ccccc3)CC4(CC4)C#N	8.1
437	O=C(N(C1CC[N+](CC1)CCc2ccccc2)c3ccccc3)CSCC	8.1
438	O=C(N(C1CC[N+](CC1)CCc2ccccc2)c3ccccc3)COCC#C	8.1
439	O=S(=O)(N(C1CC[N+](CC1)CCc2ccccc2)c3ccccc3)C=C	8.1

440	O=C(OC1COC1)N(C2CC[N+](CC2)CCc3ccccc3)c4ccccc4	8.1
441	O=C(N(C1CC[N+](CC1)CCc2ccccc2)c3ccccc3)Cc4cocc4	8.1
442	FC(F)CSN(C1CC[N+](CC1)CCc2ccccc2)c3ccccc3	8.1
443	FCCCON(C1CC[N+](CC1)CCc2ccccc2)c3ccccc3	8.1
444	O=C(N(C1CC[N+](CC1)CCc2ccccc2)c3ccccc3)C/C=N/OC	8.1
445	O[C@H](N(C1CC[N+](CC1)CCc2ccccc2)c3ccccc3)C=C	8.1
446	O=C(N(C1CC[N+](CC1)CCc2ccccc2)c3ccccc3)NS(=O)(=O)N	8.1
447	FCCCSC(=O)N(C1CC[N+](CC1)CCc2ccccc2)c3ccccc3	8.1
448	O=[S@@](N(C1CC[N+](CC1)CCc2ccccc2)c3ccccc3)N	8.1
449	O[C@H](N(C1CC[N+](CC1)CCc2ccccc2)c3ccccc3)CC#C	8.1
450	O=C(N(C1CC[N+](CC1)CCc2ccccc2)c3ccccc3)CC#N	8.1
451	O=C(N)/C=C/N(C1CC[N+](CC1)CCc2ccccc2)c3ccccc3	8.1
452	FC(F)(F)CC(=O)N(C1CC[N+](CC1)CCc2ccccc2)c3ccccc3	8.0
453	O=C(N(C1CC[N+](CC1)CCc2ccccc2)c3ccccc3)C[C@@H]4C(C4)=C	8.0
454	FCS(=O)(=O)N(C1CC[N+](CC1)CCc2ccccc2)c3ccccc3	8.0
455	O=[S@](CON(C1CC[N+](CC1)CCc2ccccc2)c3ccccc3)C	8.0
456	S=C(N(C1CC[N+](CC1)CCc2ccccc2)c3ccccc3)CC	8.0
457	FC(F)(F)OC(=O)N(C1CC[N+](CC1)CCc2ccccc2)c3ccccc3	8.0
458	FC1(F)C[C@H]1C(=O)N(C2CC[N+](CC2)CCc3ccccc3)c4ccccc4	8.0
459	FC(F)(F)[C@@H](F)C(=O)N(C1CC[N+](CC1)CCc2ccccc2)c3ccccc3	8.0
460	O=C(N(C1CC[N+](CC1)CCc2ccccc2)c3ccccc3)NOCC#C	8.0
461	F[C@@H](CNC(=O)N(C1CC[N+](CC1)CCc2ccccc2)c3ccccc3)C	8.0
462	FC(F)(F)C(C(=O)N(C1CC[N+](CC1)CCc2ccccc2)c3ccccc3)=C	8.0
463	Clc1cc(N(C2CC[N+](CC2)CCc3ccccc3)c4ccccc4)no1	8.0
464	O=C(N(C1CC[N+](CC1)CCc2ccccc2)c3ccccc3)CC(CC)(C)C	8.0
465	O=C(N(C1CC[N+](CC1)CCc2ccccc2)c3ccccc3)N[C@H](C)C=O	8.0
466	O1C[C@H]1CCON(C2CC[N+](CC2)CCc3ccccc3)c4ccccc4	8.0
467	FC(F)(F)[C@H](O)C(=O)N(C1CC[N+](CC1)CCc2ccccc2)c3ccccc3	8.0
468	O=C(N(C1CC[N+](CC1)CCc2ccccc2)c3ccccc3)[C@@H](OC)C	8.0
469	O=C(N(C1CC[N+](CC1)CCc2ccccc2)c3ccccc3)N[C@@H](C)C#N	8.0
470	O=C(OC(C)(C)C#N)N(C1CC[N+](CC1)CCc2ccccc2)c3ccccc3	8.0
471	O=C(O[C@H](C)C#C)N(C1CC[N+](CC1)CCc2ccccc2)c3ccccc3	8.0
472	FCCCNC(=O)N(C1CC[N+](CC1)CCc2ccccc2)c3ccccc3	8.0
473	O=C(N(C1CC[N+](CC1)CCc2ccccc2)c3ccccc3)NOC(C)C	8.0
474	O=C(N(C1CC[N+](CC1)CCc2ccccc2)c3ccccc3)[C@@H]4[C@@H](O4)C(=O)N	8.0
475	O=C(N(C1CC[N+](CC1)CCc2ccccc2)c3ccccc3)CC4=COCC4	8.0
476	FC(F)CC(=O)N(C1CC[N+](CC1)CCc2ccccc2)c3ccccc3	7.9
477	O=CN(C1CC[N+](CC1)CCc2ccccc2)c3ccccc3	7.9
478	O=C(N(C1CC[N+](CC1)CCc2ccccc2)c3ccccc3)C[S@](=O)C	7.9
479	O=C(N(C1CC[N+](CC1)CCc2ccccc2)c3ccccc3)CC(C)=C	7.9
480	S=CON(C1CC[N+](CC1)CCc2ccccc2)c3ccccc3	7.9
481	FC(F)(CC(=O)N(C1CC[N+](CC1)CCc2ccccc2)c3ccccc3)C	7.9

482	S=C(N(C1CC[N+](CC1)CCc2ccccc2)c3ccccc3)CCC	7.9
483	ClC(F)(F)CON(C1CC[N+](CC1)CCc2ccccc2)c3ccccc3	7.9
484	ClC(Cl)=CC(=O)N(C1CC[N+](CC1)CCc2ccccc2)c3ccccc3	7.9
485	O=C(N)[C@@H](N(C1CC[N+](CC1)CCc2ccccc2)c3ccccc3)CCC	7.9
486	FC(F)(F)CON(C1CC[N+](CC1)CCc2ccccc2)c3ccccc3	7.9
487	[O-][N+](=O)CCCON(C1CC[N+](CC1)CCc2ccccc2)c3ccccc3	7.9
488	O=C(/C=C/N(C1CC[N+](CC1)CCc2ccccc2)c3ccccc3)C	7.9
489	S=C(N(C1CC[N+](CC1)CCc2ccccc2)c3ccccc3)C	7.8
490	O/C(N(C1CC[N+](CC1)CCc2ccccc2)c3ccccc3)=C\C	7.8
491	O=C(N(C1CC[N+](CC1)CCc2ccccc2)c3ccccc3)NC[N+]	7.8
492	FC(F)(F)C[C@H](O)N(C1CC[N+](CC1)CCc2ccccc2)c3ccccc3	7.8
493	O=C(N)[C@@H](N(C1CC[N+](CC1)CCc2ccccc2)c3ccccc3)C	7.8
494	CO/N=C/C=C/N(C1CC[N+](CC1)CCc2ccccc2)c3ccccc3	7.8
495	O/C(N(C1CC[N+](CC1)CCc2ccccc2)c3ccccc3)=C\S(=O)(=O)C	7.8
496	CC(CON(C1CC[N+](CC1)CCc2ccccc2)c3ccccc3)=C	7.8
497	O=C/C=C/N(C1CC[N+](CC1)CCc2ccccc2)c3ccccc3	7.8
498	[N-]=[N+]=C(/N(C1CC[N+](CC1)CCc2ccccc2)c3ccccc3)C	7.7
499	FC1(CON(C2CC[N+](CC2)CCc3ccccc3)c4ccccc4)COC1	7.7
500	O=C(N(C1CC[N+](CC1)CCc2ccccc2)c3ccccc3)NC4COC4	7.7

Table S4

List, SMILE and predicted pK_i values for Series 2.

N°	SMILES	Pred pK_i
1	O=C(N(C1CC[N+](CC1)CCc2ccccc2)C=3C=CNCC3)CC	9.0
2	O=C(N(C1CC[N+](CC1)CCc2ccccc2)c3cc[nH+]c(N)c3)CC	8.9
3	Fc1c(N)cccc1N(C2CC[N+](CC2)CCc3ccccc3)C(=O)CC	8.9
4	O=C(N(C1CC[N+](CC1)CCc2ccccc2)c3ccccc3OCC)CC	8.9
5	O=C(N(C1CC[N+](CC1)CCc2ccccc2)c3cccc(O)c3)CC	8.8
6	O=C(N(C1CC[N+](CC1)CCc2ccccc2)c3cccc(O)c3N)CC	8.8
7	Oc1c(N)cccc1N(C2CC[N+](CC2)CCc3ccccc3)C(=O)CC	8.7
8	O=C(N(C1CC[N+](CC1)CCc2ccccc2)c3cccc(c3O)C)CC	8.7
9	O=C(N(C1CC[N+](CC1)CCc2ccccc2)C3=CNCCC3)CC	8.7
10	Clc1c(N)cccc1N(C2CC[N+](CC2)CCc3ccccc3)C(=O)CC	8.7
11	O=C(N(N1CC=CCO1)C2CC[N+](CC2)CCc3ccccc3)CC	8.7
12	O=C(N(C1CC[N+](CC1)CCc2ccccc2)C3=CCCCO3)CC	8.7
13	O=C(N(N1C=CNC1)C2CC[N+](CC2)CCc3ccccc3)CC	8.7
14	O=C(N(C1CC[N+](CC1)CCc2ccccc2)c3cccc4CCOc43)CC	8.7
15	Fc1ccccc1N(C2CC[N+](CC2)CCc3ccccc3)C(=O)CC	8.6
16	O=C(N(C1CC[N+](CC1)CCc2ccccc2)C3=CCC=C3)CC	8.6
17	O=C(N(C1CC[N+](CC1)CCc2ccccc2)c3ccccc3OC)CC	8.6
18	FCOc1ccccc1N(C2CC[N+](CC2)CCc3ccccc3)C(=O)CC	8.6

19	O=C(N(C1CC[N+](CC1)CCc2ccccc2)c3ccccn3)CC	8.6
20	O=C(N(C1CC[N+](CC1)CCc2ccccc2)c3ccn(N)c3)CC	8.6
21	O=C(N(C1CC[N+](CC1)CCc2ccccc2)c3ccc(s3)CO)CC	8.6
22	Oc1cccc(O)c1N(C2CC[N+](CC2)CCc3ccccc3)C(=O)CC	8.6
23	Clc1cccc(O)c1N(C2CC[N+](CC2)CCc3ccccc3)C(=O)CC	8.6
24	O=C(N(C1CC[N+](CC1)CCc2ccccc2)c3cccc(OC)c3)CC	8.6
25	O=C(N(C1CC[N+](CC1)CCc2ccccc2)c3cccc(OC(=O)N)c3)CC	8.6
26	O=C(N(C1CC[N+](CC1)CCc2ccccc2)c3ccc(N)c(O)c3)CC	8.6
27	O=C(N(C1CC[N+](CC1)CCc2ccccc2)c3cccc(NC)c3)CC	8.6
28	O=C(N(C1CC[N+](CC1)CCc2ccccc2)c3coc4c3cc[nH]4)CC	8.6
29	O=C(N(C1CC[N+](CC1)CCc2ccccc2)c3cccc(CCN#C)c3)CC	8.6
30	O=C(N(C1CC[N+](CC1)CCc2ccccc2)c3cc([nH+]c(N)c3)N)CC	8.6
31	Clc1cc(N)cc(N(C2CC[N+](CC2)CCc3ccccc3)C(=O)CC)c1	8.6
32	O=C(N(C1CC[N+](CC1)CCc2ccccc2)c3ccccc3)CC	8.5
33	O=C(N(C1CC[N+](CC1)CCc2ccccc2)C=3C=CCCC3)CC	8.5
34	O=C(N(C1CC[N+](CC1)CCc2ccccc2)c3cc[nH]c3)CC	8.5
35	Clc1ccccc1N(C2CC[N+](CC2)CCc3ccccc3)C(=O)CC	8.5
36	O=C(N(C1CC[N+](CC1)CCc2ccccc2)c3ccc(OC=O)cc3)CC	8.5
37	O=C(N(C1CC[N+](CC1)CCc2ccccc2)c3ccc(N)cc3)CC	8.5
38	O=C(N(C1CC[N+](CC1)CCc2ccccc2)c3csc(N)c3)CC	8.5
39	O=C(N(C1CC[N+](CC1)CCc2ccccc2)c3ccccc3C#C)CC	8.5
40	FCc1cccc(N(C2CC[N+](CC2)CCc3ccccc3)C(=O)CC)c1	8.5
41	O=C(N(C1CC[N+](CC1)CCc2ccccc2)c3coc(N)c3)CC	8.5
42	O=C(N(C1CC[N+](CC1)CCc2ccccc2)c3c(O)ncc(O)c3)CC	8.5
43	Sc1ccccc1N(C2CC[N+](CC2)CCc3ccccc3)C(=O)CC	8.5
44	O=C(N(C1CC[N+](CC1)CCc2ccccc2)C3=CCCS3)CC	8.5
45	O=C(N([C@@]12CCCC[C@@H]1O2)C3CC[N+](CC3)CCc4ccccc4)CC	8.5
46	O=C(N(C1CC[N+](CC1)CCc2ccccc2)c3ccccc3CN#C)CC	8.5
47	FCOc1cccc(N(C2CC[N+](CC2)CCc3ccccc3)C(=O)CC)c1	8.5
48	O=C(N(C1CC[N+](CC1)CCc2ccccc2)c3cccc4c3nc(o4)O)CC	8.5
49	O=C(N(C1CC[N+](CC1)CCc2ccccc2)c3ccc(OC(=O)N)cc3)CC	8.5
50	O=C(N(C1CC[N+](CC1)CCc2ccccc2)c3ccccc3O[N+]([O-])=O)CC	8.5
51	O=C(N(C1CC[N+](CC1)CCc2ccccc2)c3cccc(c3)CO)CC	8.5
52	O=C(N(C1CC[N+](CC1)CCc2ccccc2)c3cccc4ccoc43)CC	8.5
53	F[C@@H](c1cccc(N(C2CC[N+](CC2)CCc3ccccc3)C(=O)CC)c1)C	8.5
54	Oc1ccsc1N(C2CC[N+](CC2)CCc3ccccc3)C(=O)CC	8.5
55	FCCc1ccccc1N(C2CC[N+](CC2)CCc3ccccc3)C(=O)CC	8.5
56	O=C(N(C1CC[N+](CC1)CCc2ccccc2)c3ccc4c(CCN4)c3)CC	8.5
57	O=C(N(C1CC[N+](CC1)CCc2ccccc2)c3ccccc3SC)CC	8.5
58	O=C(N(C1CC[N+](CC1)CCc2ccccc2)c3ccc(N)c(c3)C#C)CC	8.5
59	O=C(N(C1CC[N+](CC1)CCc2ccccc2)c3ccccc3ON)CC	8.5
60	O=C(N(C1CC[N+](CC1)CCc2ccccc2)C3=CSCCO3)CC	8.5

61	O=C(N(C1CC[N+](CC1)CCc2ccccc2)c3ccccc3/C=C/C)CC	8.5
62	O=C(N(C1CC[N+](CC1)CCc2ccccc2)c3cscc3OC)CC	8.5
63	Clc1ccsc1N(C2CC[N+](CC2)CCc3ccccc3)C(=O)CC	8.5
64	Oc1cc(N)ccc1N(C2CC[N+](CC2)CCc3ccccc3)C(=O)CC	8.5
65	O=C(N(C1CC[N+](CC1)CCc2ccccc2)C3=CNCN3)CC	8.5
66	O=C(N(C1CC[N+](CC1)CCc2ccccc2)c3ccccc3NC=O)CC	8.5
67	O=C(N(C1CC[N+](CC1)CCc2ccccc2)c3cccc4csnc43)CC	8.5
68	O=C(N(C1CC[N+](CC1)CCc2ccccc2)C3=CCC=CC3)CC	8.4
69	O=C(N(C1CC[N+](CC1)CCc2ccccc2)c3cccc(c3)C)CC	8.4
70	O=C(N(C1CC[N+](CC1)CCc2ccccc2)C3=CC=CCS3)CC	8.4
71	O=C(N(C1CC[N+](CC1)CCc2ccccc2)C3=CCCCC3)CC	8.4
72	O=C(N(C1CC[N+](CC1)CCc2ccccc2)c3cccc(c3)C#C)CC	8.4
73	Clc1cccc(N(C2CC[N+](CC2)CCc3ccccc3)C(=O)CC)c1	8.4
74	O=C(N(C1CC[N+](CC1)CCc2ccccc2)c3ccc(s3)N)CC	8.4
75	F[C@H]1C=CC(N(C2CC[N+](CC2)CCc3ccccc3)C(=O)CC)=CC1	8.4
76	Fc1cccc(N(C2CC[N+](CC2)CCc3ccccc3)C(=O)CC)c1O	8.4
77	O=C(N(N1C=CCCC1)C2CC[N+](CC2)CCc3ccccc3)CC	8.4
78	O=C(N(C1CC[N+](CC1)CCc2ccccc2)C3=CCCOC3)CC	8.4
79	O=C(N(C1CC[N+](CC1)CCc2ccccc2)c3c[nH]c(c3)C)CC	8.4
80	O=C(N(C1CC[N+](CC1)CCc2ccccc2)c3cc(N)cs3)CC	8.4
81	O=C(N(C1CC[N+](CC1)CCc2ccccc2)c3csc(O)c3)CC	8.4
82	O=C(N(C1CC[N+](CC1)CCc2ccccc2)c3cccc(c3)C=C)CC	8.4
83	O=C(N(C1CC[N+](CC1)CCc2ccccc2)c3ccn(c3)C)CC	8.4
84	O=C(N(C1CC[N+](CC1)CCc2ccccc2)c3c(O)ccc(c3)C)CC	8.4
85	O=C(N(N1CCCCC1)C2CC[N+](CC2)CCc3ccccc3)CC	8.4
86	O=C(N(C1CC[N+](CC1)CCc2ccccc2)c3ccc(N)c(N)c3)CC	8.4
87	O=C(N(C1CC[N+](CC1)CCc2ccccc2)c3cccc(N)c3)CC	8.4
88	O=C(N(C1CC[N+](CC1)CCc2ccccc2)c3cccc(c3)CN#C)CC	8.4
89	O=C(N(C1CC[N+](CC1)CCc2ccccc2)C=3C=C[C@@H](CC3)C)CC	8.4
90	O=C(N(C1CC[N+](CC1)CCc2ccccc2)c3ccccc3C=C)CC	8.4
91	O=C(N(C1CC[N+](CC1)CCc2ccccc2)c3c(C)c[nH]c3)CC	8.4
92	Oc1c(N(C2CC[N+](CC2)CCc3ccccc3)C(=O)CC)ccc([nH+]1)N	8.4
93	SCc1cccc(N(C2CC[N+](CC2)CCc3ccccc3)C(=O)CC)c1	8.4
94	O=C(N(C1CC[N+](CC1)CCc2ccccc2)c3ccccc3CC)CC	8.4
95	Fc1c(cccc1N(C2CC[N+](CC2)CCc3ccccc3)C(=O)CC)C	8.4
96	O=C(N(C1CC[N+](CC1)CCc2ccccc2)c3cccc(O[N+]([O-])=O)c3)CC	8.4
97	Clc1c(cccc1N(C2CC[N+](CC2)CCc3ccccc3)C(=O)CC)C	8.4
98	O=C(N(C1CC[N+](CC1)CCc2ccccc2)c3cccc(c3)CC=C)CC	8.4
99	Clc1c(N)ccc(N(C2CC[N+](CC2)CCc3ccccc3)C(=O)CC)c1	8.4
100	O=C(N(C1CC[N+](CC1)CCc2ccccc2)c3ccc(O)c(N)c3)CC	8.4
101	O=C(N(C1CC[N+](CC1)CCc2ccccc2)c3cccc(NN)c3)CC	8.4
102	O=C(N(C1CC[N+](CC1)CCc2ccccc2)c3ccc4c(NCN4)c3)CC	8.4

103	Sc1cc(cc(N(C2CC[N+](CC2)CCc3ccccc3)C(=O)CC)c1)C	8.4
104	O[C@@H]1CC=C(N(C2CC[N+](CC2)CCc3ccccc3)C(=O)CC)CC1	8.4
105	O=C(N(C1CC[N+](CC1)CCc2ccccc2)c3c[nH+]c(N)c(N)c3)CC	8.4
106	O=C(N(C1CC[N+](CC1)CCc2ccccc2)c3ccccc3NC#N)CC	8.4
107	O=C(N(C1CC[N+](CC1)CCc2ccccc2)C3=COCCS3)CC	8.4
108	O=C(N(C1CC[N+](CC1)CCc2ccccc2)C3=COCCO3)CC	8.4
109	O=C(N(C1CC[N+](CC1)CCc2ccccc2)c3ccccc3C4CC4)CC	8.4
110	O=C(N(n1ccc2c1cc[nH]2)C3CC[N+](CC3)CCc4ccccc4)CC	8.4
111	O=C(N(C1CC[N+](CC1)CCc2ccccc2)c3c[nH]c4cscc43)CC	8.4
112	Fc1cccc(N(C2CC[N+](CC2)CCc3ccccc3)C(=O)CC)c1OC	8.4
113	Sc1ccsc1N(C2CC[N+](CC2)CCc3ccccc3)C(=O)CC	8.4
114	Fc1cc(N)ccc1N(C2CC[N+](CC2)CCc3ccccc3)C(=O)CC	8.4
115	O=C(N(C1CC[N+](CC1)CCc2ccccc2)c3ccccc3N#C)CC	8.4
116	O=C(N(C1CC[N+](CC1)CCc2ccccc2)[C@H]([C@H]3CO3)C)CC	8.4
117	O=C(N(C1CC[N+](CC1)CCc2ccccc2)c3cccc4c3OCO4)CC	8.4
118	Sc1ccc(F)c(N(C2CC[N+](CC2)CCc3ccccc3)C(=O)CC)c1	8.4
119	O=C(N(C1CC[N+](CC1)CCc2ccccc2)c3ccccc3C(C)=C)CC	8.4
120	O=C(N(C1CC[N+](CC1)CCc2ccccc2)c3ccc(o3)N)CC	8.4
121	O=C(N(C1CC[N+](CC1)CCc2ccccc2)c3ccc(OC(=O)C=C)cc3)CC	8.4
122	O=C(N(C1CC[N+](CC1)CCc2ccccc2)c3cccs3)CC	8.3
123	Fc1cccc(N(C2CC[N+](CC2)CCc3ccccc3)C(=O)CC)c1	8.3
124	O=C(N(C1CC[N+](CC1)CCc2ccccc2)c3cc(C)cs3)CC	8.3
125	O=C(N(C1CC[N+](CC1)CCc2ccccc2)c3ccsc3N)CC	8.3
126	O=C(N(C1CC[N+](CC1)CCc2ccccc2)c3ccc[nH]3)CC	8.3
127	O=C(N(C1CC[N+](CC1)CCc2ccccc2)c3ccccc3C)CC	8.3
128	O=C(N(C1CC[N+](CC1)CCc2ccccc2)C3=CCCC3)CC	8.3
129	O=C(N(C1CC[N+](CC1)CCc2ccccc2)C=3C=CCNC3)CC	8.3
130	O=C(N(C1CC[N+](CC1)CCc2ccccc2)c3c(O)nccc3)CC	8.3
131	O=C(N(C1CC[N+](CC1)CCc2ccccc2)c3cccc(c3)CC)CC	8.3
132	O=C(N(N1C=CCC1)C2CC[N+](CC2)CCc3ccccc3)CC	8.3
133	Fc1cscc1N(C2CC[N+](CC2)CCc3ccccc3)C(=O)CC	8.3
134	FCc1ccc(N(C2CC[N+](CC2)CCc3ccccc3)C(=O)CC)cc1	8.3
135	FCc1ccccc1N(C2CC[N+](CC2)CCc3ccccc3)C(=O)CC	8.3
136	O=C(N(C1CC[N+](CC1)CCc2ccccc2)c3ccc(O)c(O)c3)CC	8.3
137	O=C(N(C1CC[N+](CC1)CCc2ccccc2)c3ccc([nH+]c3)N)CC	8.3
138	O=C(N(C1CC[N+](CC1)CCc2ccccc2)C3=COCCC3)CC	8.3
139	O=C(N(C1CC[N+](CC1)CCc2ccccc2)c3cccc(SC)c3)CC	8.3
140	O=C(N(C1CC[N+](CC1)CCc2ccccc2)c3cc(cc(c3)C)C)CC	8.3
141	FC(F)Oc1ccccc1N(C2CC[N+](CC2)CCc3ccccc3)C(=O)CC	8.3
142	O=C(N(C1CC[N+](CC1)CCc2ccccc2)c3cccc(c3)C#CC)CC	8.3
143	Oc1cccc(c1N(C2CC[N+](CC2)CCc3ccccc3)C(=O)CC)C	8.3
144	Oc1c([nH+]ccc1N(C2CC[N+](CC2)CCc3ccccc3)C(=O)CC)N	8.3

145	O=C(N(C1CC[N+](CC1)CCc2ccccc2)c3cccc(O)n3)CC	8.3
146	O=C(N(C1CC[N+](CC1)CCc2ccccc2)[C@@H]3CC=CCC3)CC	8.3
147	O=C(N(C1CC[N+](CC1)CCc2ccccc2)c3cccc(N#C)c3)CC	8.3
148	O=C(N(C1CC[N+](CC1)CCc2ccccc2)c3ccc4c(c3)cc[nH]4)CC	8.3
149	O=C(N(C1CC[N+](CC1)CCc2ccccc2)c3cc(ccc3O)C#C)CC	8.3
150	O=C(N(C1CC[N+](CC1)CCc2ccccc2)c3cccc(C4CC4)c3)CC	8.3
151	Oc1c(N)cc[nH+]c1N(C2CC[N+](CC2)CCc3ccccc3)C(=O)CC	8.3
152	O=C(N(C1CC[N+](CC1)CCc2ccccc2)c3c(N)ccs3)CC	8.3
153	O=C(N(C1CC[N+](CC1)CCc2ccccc2)c3ccc4c[nH]cc4c3)CC	8.3
154	Fc1cccc(N(C2CC[N+](CC2)CCc3ccccc3)C(=O)CC)c1C	8.3
155	O=C(N(C1CC[N+](CC1)CCc2ccccc2)c3c(O)nccc3O)CC	8.3
156	Sc1c(N)ccc(N(C2CC[N+](CC2)CCc3ccccc3)C(=O)CC)c1	8.3
157	O=C(N(C1CC[N+](CC1)CCc2ccccc2)c3cccc(c3)CCC)CC	8.3
158	O=C(N(C1CC[N+](CC1)CCc2ccccc2)/C(=C/C)C=C)CC	8.3
159	O=C(N(C1CC[N+](CC1)CCc2ccccc2)C3=CCCC[C@H]3C)CC	8.3
160	FCCN(N(C1CC[N+](CC1)CCc2ccccc2)C(=O)CC)C	8.3
161	O=C(N(C1CC[N+](CC1)CCc2ccccc2)C3=CN(CCC3)C)CC	8.3
162	O=C(N(C1CC[N+](CC1)CCc2ccccc2)c3cscn3)CC	8.3
163	Clc1cccc(N(C2CC[N+](CC2)CCc3ccccc3)C(=O)CC)c1O	8.3
164	O=C(N(C1CC[N+](CC1)CCc2ccccc2)c3c(O)nc(s3)O)CC	8.3
165	Fc1cc(N(C2CC[N+](CC2)CCc3ccccc3)C(=O)CC)ccc1NC	8.3
166	O=C(N(C1CC[N+](CC1)CCc2ccccc2)c3cc(N)c[nH]3)CC	8.3
167	O=C(N(C1CC[N+](CC1)CCc2ccccc2)c3cc(N)c(O)c(N)c3)CC	8.3
168	O=C(N(C1CC[N+](CC1)CCc2ccccc2)c3cccc(N(C)C)c3)CC	8.3
169	Oc1cc(O)ccc1N(C2CC[N+](CC2)CCc3ccccc3)C(=O)CC	8.3
170	O=C(N(C1CC[N+](CC1)CCc2ccccc2)c3cccc(c3)C(C)C)CC	8.3
171	O=C(N(C1CC[N+](CC1)CCc2ccccc2)C3=CC=C(N)CC3)CC	8.3
172	O=C(N(C1CC[N+](CC1)CCc2ccccc2)c3ccc(s3)NN)CC	8.3
173	O=C(N(C1CC[N+](CC1)CCc2ccccc2)c3cccc(c3)C(C)=C)CC	8.3
174	O=C(N(C1CC[N+](CC1)CCc2ccccc2)c3cc[nH+]c(NN)c3)CC	8.3
175	O=C(N(C1CC[N+](CC1)CCc2ccccc2)c3cccc(c3)C(N)=[N+])CC	8.3
176	O=C(N(C1CC[N+](CC1)CCc2ccccc2)c3cn4c(c3)ccs4)CC	8.3
177	SCC/C=C/N(C1CC[N+](CC1)CCc2ccccc2)C(=O)CC	8.3
178	O=C(N(n1ccc2ccoc21)C3CC[N+](CC3)CCc4ccccc4)CC	8.3
179	O=C(N(C1CC[N+](CC1)CCc2ccccc2)c3cc(cc(c3)C)CC)CC	8.3
180	Sc1c(cccc1N(C2CC[N+](CC2)CCc3ccccc3)C(=O)CC)C	8.3
181	Clc1cccc(N(C2CC[N+](CC2)CCc3ccccc3)C(=O)CC)c1N	8.3
182	O=C(N(C1CC[N+](CC1)CCc2ccccc2)c3ccc4c(C4)c3)CC	8.2
183	O=C(N(C1CC[N+](CC1)CCc2ccccc2)C3=CC=CCC3)CC	8.2
184	Sc1cccc(N(C2CC[N+](CC2)CCc3ccccc3)C(=O)CC)c1	8.2
185	O=C(N(C1CC[N+](CC1)CCc2ccccc2)c3ccc4CCc4c3)CC	8.2
186	Oc1cscc1N(C2CC[N+](CC2)CCc3ccccc3)C(=O)CC	8.2

187	O=C(N(C1CC[N+](CC1)CCc2ccccc2)c3cccc([nH+]3)N)CC	8.2
188	O=C(N(C1CC[N+](CC1)CCc2ccccc2)c3ccsc3O)CC	8.2
189	O=C(N(C1CC[N+](CC1)CCc2ccccc2)C3=CCCCN3)CC	8.2
190	O=C(N(C1CC[N+](CC1)CCc2ccccc2)c3ccccc3NO)CC	8.2
191	O=C(N(C1CC[N+](CC1)CCc2ccccc2)C3=CCC=CN3)CC	8.2
192	O=C(N(C1CC[N+](CC1)CCc2ccccc2)c3cc(c[nH]3)C)CC	8.2
193	O=C(N(C1CC[N+](CC1)CCc2ccccc2)c3csc(O)n3)CC	8.2
194	O=C(N(C1CC[N+](CC1)CCc2ccccc2)c3cnc(O)cc3)CC	8.2
195	O=C(N(N1CC=CCC1)C2CC[N+](CC2)CCc3ccccc3)CC	8.2
196	Fc1cccc(N(C2CC[N+](CC2)CCc3ccccc3)C(=O)CC)c1N	8.2
197	O=C(N(C1CC[N+](CC1)CCc2ccccc2)c3ccc(c(c3)C)C)CC	8.2
198	O=C(N(C1CC[N+](CC1)CCc2ccccc2)c3cccc4cocc43)CC	8.2
199	O=C(N(C1CC[N+](CC1)CCc2ccccc2)c3ccc4ccoc4c3)CC	8.2
200	O=C(N(C1CC[N+](CC1)CCc2ccccc2)c3ccc(cc3)CC#C)CC	8.2
201	Fc1cc(cc(N(C2CC[N+](CC2)CCc3ccccc3)C(=O)CC)c1)C	8.2
202	O=C(N(C1CC[N+](CC1)CCc2ccccc2)c3ccc(NC)cc3)CC	8.2
203	O=C(N(C1CC[N+](CC1)CCc2ccccc2)c3ccc(CN#C)cc3)CC	8.2
204	O=C(N(C1CC[N+](CC1)CCc2ccccc2)c3ccoc3N)CC	8.2
205	O=C(N(C1CC[N+](CC1)CCc2ccccc2)c3ccc(N)c(O)n3)CC	8.2
206	Oc1cccnc1N(C2CC[N+](CC2)CCc3ccccc3)C(=O)CC	8.2
207	O=C(N(C1CC[N+](CC1)CCc2ccccc2)c3cc(n(c3)C)C)CC	8.2
208	SCc1ccccc1N(C2CC[N+](CC2)CCc3ccccc3)C(=O)CC	8.2
209	O=C(N(N1C=CC=CN1)C2CC[N+](CC2)CCc3ccccc3)CC	8.2
210	FC[C@H](N(C1CC[N+](CC1)CCc2ccccc2)C(=O)CC)CC	8.2
211	Fc1cc(ccc1N(C2CC[N+](CC2)CCc3ccccc3)C(=O)CC)C	8.2
212	O=C(N(C1CC[N+](CC1)CCc2ccccc2)C3=C[C@H]4CC[C@H](C4)C3)CC	8.2
213	O=C(N(C1CC[N+](CC1)CCc2ccccc2)c3ccn(c3)CC)CC	8.2
214	O=C(N(C1CC[N+](CC1)CCc2ccccc2)c3cnc(nc3O)N)CC	8.2
215	O=C(N(C1CC[N+](CC1)CCc2ccccc2)/C(CC)=C/C)CC	8.2
216	O=C(N(C1CC[N+](CC1)CCc2ccccc2)C3=CC[C@@H](CC3)C)CC	8.2
217	O=C(N(C1CC[N+](CC1)CCc2ccccc2)c3ccc[nH+]c3N)CC	8.2
218	O=C(N(C1CC[N+](CC1)CCc2ccccc2)c3ccccc3OC(=O)N)CC	8.2
219	Brc1cc(N(C2CC[N+](CC2)CCc3ccccc3)C(=O)CC)c[nH]1	8.2
220	O=C(N(C1CC[N+](CC1)CCc2ccccc2)c3c(O)ncc(c3)C)CC	8.2
221	O=C(N(C1CC[N+](CC1)CCc2ccccc2)c3ccc(N)c(c3)C=C)CC	8.2
222	O=C(N(C1CC[N+](CC1)CCc2ccccc2)C3=CC[C@H](CC3)CC)CC	8.2
223	Oc1c(N)c(O)ccc1N(C2CC[N+](CC2)CCc3ccccc3)C(=O)CC	8.2
224	O=C(N(C1CC[N+](CC1)CCc2ccccc2)c3ccc4CCOc4c3)CC	8.2
225	O=C(N(C1CC[N+](CC1)CCc2ccccc2)c3ccc4CCNc4c3)CC	8.2
226	Clc1cc(cc(N(C2CC[N+](CC2)CCc3ccccc3)C(=O)CC)c1)C	8.2
227	O=C(N(C1CC[N+](CC1)CCc2ccccc2)C3=C[C@@H]4C=C[C@H]3CC4)CC	8.2
228	Clc1cnc(O)c(N(C2CC[N+](CC2)CCc3ccccc3)C(=O)CC)c1	8.2

229	FC(F)(F)c1cccc(N(C2CC[N+](CC2)CCc3ccccc3)C(=O)CC)c1	8.2
230	O=C(N(C1CC[N+](CC1)CCc2ccccc2)c3csc4cocc43)CC	8.2
231	O=C(N(C1CC[N+](CC1)CCc2ccccc2)c3ccc(s3)CC=C)CC	8.2
232	Clc1cc(sc1N(C2CC[N+](CC2)CCc3ccccc3)C(=O)CC)C	8.2
233	O=C(N(C1CC[N+](CC1)CCc2ccccc2)c3cccc4c3oc(O)n4)CC	8.2
234	Clc1cccc(N(C2CC[N+](CC2)CCc3ccccc3)C(=O)CC)c1C	8.2
235	O=C(N(C1CC[N+](CC1)CCc2ccccc2)c3cc(ccc3C)C)CC	8.2
236	O=C(N(C1CC[N+](CC1)CCc2ccccc2)c3ccc4c(nco4)c3)CC	8.2
237	Sc1cccc(N(C2CC[N+](CC2)CCc3ccccc3)C(=O)CC)c1C	8.2
238	O=C(N(C1CC[N+](CC1)CCc2ccccc2)c3ccc4CCSc4c3)CC	8.2
239	O=C(N(C1CC[N+](CC1)CCc2ccccc2)/C=C/CCC)CC	8.2
240	O=C(N(C1CC[N+](CC1)CCc2ccccc2)c3ccc(O)cn3)CC	8.2
241	O=C(N(C1CC[N+](CC1)CCc2ccccc2)c3cccc4CCCc43)CC	8.2
242	O=C(N(C1CC[N+](CC1)CCc2ccccc2)c3ccc(CCN#C)cc3)CC	8.2
243	O=C(N(C1CC[N+](CC1)CCc2ccccc2)c3ccccc3CO)CC	8.2
244	O=C(N(C1CC[N+](CC1)CCc2ccccc2)c3ccc(NC)c(c3)C)CC	8.2
245	Fc1ccc(N(C2CC[N+](CC2)CCc3ccccc3)C(=O)CC)c(OC)c1	8.2
246	O=C(N(C1CC[N+](CC1)CCc2ccccc2)c3ccsc3)CC	8.1
247	O=C(N(C1CC[N+](CC1)CCc2ccccc2)c3ccc(cc3)C)CC	8.1
248	O=C(N(C1CC[N+](CC1)CCc2ccccc2)c3ccc(s3)C)CC	8.1
249	O=C(N(C1CC[N+](CC1)CCc2ccccc2)C3=CCCSC3)CC	8.1
250	O=C(N(C1CC[N+](CC1)CCc2ccccc2)c3csc(c3)C)CC	8.1
251	Clc1ccc(N(C2CC[N+](CC2)CCc3ccccc3)C(=O)CC)cc1	8.1
252	O=C(N(C1CC[N+](CC1)CCc2ccccc2)c3cc(cs3)C#C)CC	8.1
253	Sc1ccc(s1)N(C2CC[N+](CC2)CCc3ccccc3)C(=O)CC	8.1
254	Fc1csc(N(C2CC[N+](CC2)CCc3ccccc3)C(=O)CC)c1	8.1
255	O=C(N(C1CC[N+](CC1)CCc2ccccc2)c3cccc(O)c3O)CC	8.1
256	Clc1csc(N(C2CC[N+](CC2)CCc3ccccc3)C(=O)CC)c1	8.1
257	Oc1ccccc1N(C2CC[N+](CC2)CCc3ccccc3)C(=O)CC	8.1
258	O=C(N(C1CC[N+](CC1)CCc2ccccc2)c3ccc4c(O4)c3)CC	8.1
259	O=C(N(C1CC[N+](CC1)CCc2ccccc2)c3ccc(CC)cc3)CC	8.1
260	O=C(N(C1CC[N+](CC1)CCc2ccccc2)c3ccccc3NC)CC	8.1
261	O=C(N(C1CC[N+](CC1)CCc2ccccc2)c3ccc4cocc4c3)CC	8.1
262	Fc1c(N)ccc(N(C2CC[N+](CC2)CCc3ccccc3)C(=O)CC)c1	8.1
263	Oc1cc(ccc1N(C2CC[N+](CC2)CCc3ccccc3)C(=O)CC)C	8.1
264	Fc1ccc(N(C2CC[N+](CC2)CCc3ccccc3)C(=O)CC)c(O)c1N	8.1
265	O=C(N(C1CC[N+](CC1)CCc2ccccc2)c3cc(c(s3)C)C)CC	8.1
266	O=C(N(N(C=C)C=C)C1CC[N+](CC1)CCc2ccccc2)CC	8.1
267	O=C(N(C1CC[N+](CC1)CCc2ccccc2)c3c(C)csc3)CC	8.1
268	Fc1ccc(N(C2CC[N+](CC2)CCc3ccccc3)C(=O)CC)cc1N	8.1
269	O=C(N(C1CC[N+](CC1)CCc2ccccc2)C=3CC[C@H](C3)C)CC	8.1
270	O=C(N(C1CC[N+](CC1)CCc2ccccc2)c3cnc(s3)O)CC	8.1

271	FC(F)Oc1cccc(N(C2CC[N+](CC2)CCc3ccccc3)C(=O)CC)c1	8.1
272	O=C(N(C1CC[N+](CC1)CCc2ccccc2)c3cc(CC)cs3)CC	8.1
273	O=C(N(C1CC[N+](CC1)CCc2ccccc2)c3ccc(O[N+]([O-])=O)cc3)CC	8.1
274	O[C@H]1[C@H](O)C=CC(N(C2CC[N+](CC2)CCc3ccccc3)C(=O)CC)=C1	8.1
275	O=C(N(C1CC[N+](CC1)CCc2ccccc2)c3cccc(ON)c3)CC	8.1
276	O=C(N(C1CC[N+](CC1)CCc2ccccc2)C3=CCC3)CC	8.1
277	O=C(N(C1CC[N+](CC1)CCc2ccccc2)c3ccc4CCCc4c3)CC	8.1
278	Clc1c(N(C2CC[N+](CC2)CCc3ccccc3)C(=O)CC)ccs1	8.1
279	Fc1cnc(O)c(N(C2CC[N+](CC2)CCc3ccccc3)C(=O)CC)c1	8.1
280	O=C(N(C1CC[N+](CC1)CCc2ccccc2)c3ccc4cc[nH]c4c3)CC	8.1
281	O=C(N(C1CC[N+](CC1)CCc2ccccc2)c3cnsc3)CC	8.1
282	O=C(N(C1CC[N+](CC1)CCc2ccccc2)c3cccn3C=C)CC	8.1
283	O=C(N(C1CC[N+](CC1)CCc2ccccc2)c3ccc(s3)NC)CC	8.1
284	O=C(N(C1CC[N+](CC1)CCc2ccccc2)c3ccsc3NN)CC	8.1
285	O=C(N(C1CC[N+](CC1)CCc2ccccc2)c3cc(SC)cs3)CC	8.1
286	O=C(N(C1CC[N+](CC1)CCc2ccccc2)c3ccn4c3cco4)CC	8.1
287	O=C(N(C1CC[N+](CC1)CCc2ccccc2)c3cnc(O)cc3O)CC	8.1
288	O=C(N(C1CC[N+](CC1)CCc2ccccc2)c3cc4n(nco4)c3)CC	8.1
289	O=C(N(N1CCSCC1)C2CC[N+](CC2)CCc3ccccc3)CC	8.1
290	Sc1ccc(N(C2CC[N+](CC2)CCc3ccccc3)C(=O)CC)cc1C	8.1
291	Fc1c(O)cccc1N(C2CC[N+](CC2)CCc3ccccc3)C(=O)CC	8.1
292	Clc1ccc(N(C2CC[N+](CC2)CCc3ccccc3)C(=O)CC)c(O)c1	8.1
293	Oc1cc2c(cc1N(C3CC[N+](CC3)CCc4ccccc4)C(=O)CC)cco2	8.1
294	SCc1ccc(N(C2CC[N+](CC2)CCc3ccccc3)C(=O)CC)cc1	8.1
295	FC(F)c1cccc(N(C2CC[N+](CC2)CCc3ccccc3)C(=O)CC)c1	8.1
296	O=C(N(C1CC[N+](CC1)CCc2ccccc2)c3ccc(OC)cc3)CC	8.1
297	O=C(N(N1CCSC1)C2CC[N+](CC2)CCc3ccccc3)CC	8.1
298	O=C(N(C1CC[N+](CC1)CCc2ccccc2)c3cc4cc[nH]c4s3)CC	8.1
299	Clc1ccc(N(C2CC[N+](CC2)CCc3ccccc3)C(=O)CC)cc1N	8.1
300	O=C(N(C1CC[N+](CC1)CCc2ccccc2)c3ccc(s3)CCC)CC	8.1
301	O=C(N(C1CC[N+](CC1)CCc2ccccc2)c3ccc4c(nc(o4)O)c3)CC	8.1
302	O=C(N(C1CC[N+](CC1)CCc2ccccc2)c3cccc(c3C)C)CC	8.1
303	O=C(N(C1CC[N+](CC1)CCc2ccccc2)c3ccc(cc3)CO)CC	8.1
304	FCOc1ccc(N(C2CC[N+](CC2)CCc3ccccc3)C(=O)CC)cc1	8.1
305	Cl/C(N(C1CC[N+](CC1)CCc2ccccc2)C(=O)CC)=C\C	8.1
306	O=C(N(C1CC[N+](CC1)CCc2ccccc2)C3=CCCCCC3)CC	8.1
307	O=C(N(n1ccc2c[nH]cc21)C3CC[N+](CC3)CCc4ccccc4)CC	8.1
308	Sc1c(O)ccc(N(C2CC[N+](CC2)CCc3ccccc3)C(=O)CC)c1	8.1
309	O=C(N(C1CC[N+](CC1)CCc2ccccc2)c3ccc(OC=C)cc3)CC	8.1
310	FCCc1ccc(N(C2CC[N+](CC2)CCc3ccccc3)C(=O)CC)cc1	8.1
311	O=C(N(C1CC[N+](CC1)CCc2ccccc2)c3cc(C(C)C)cs3)CC	8.1
312	FC(F)=Cc1ccc(N(C2CC[N+](CC2)CCc3ccccc3)C(=O)CC)cc1	8.1

313	O=C(N(C1CC[N+](CC1)CCc2ccccc2)c3ccc([nH+]c3)NN)CC	8.1
314	O=C(N(C1CC[N+](CC1)CCc2ccccc2)C3=C(N)CCC3)CC	8.1
315	Brc1c[nH]c(N(C2CC[N+](CC2)CCc3ccccc3)C(=O)CC)c1	8.1
316	O=C(N(C1CC[N+](CC1)CCc2ccccc2)c3cc4c[nH]cc4s3)CC	8.1
317	FC/C=C/N(C1CC[N+](CC1)CCc2ccccc2)C(=O)CC	8.1
318	O=C(N(C1CC[N+](CC1)CCc2ccccc2)c3cccc4c3nc(s4)O)CC	8.1
319	Oc1ccc(O)nc1N(C2CC[N+](CC2)CCc3ccccc3)C(=O)CC	8.1
320	Clc1cscc1N(C2CC[N+](CC2)CCc3ccccc3)C(=O)CC	8.1
321	O=C(N(OC/C=C\C)C1CC[N+](CC1)CCc2ccccc2)CC	8.1
322	O=C(N(C1CC[N+](CC1)CCc2ccccc2)c3c(sc(c3)C)N)CC	8.1
323	FC(F)c1cc(N(C2CC[N+](CC2)CCc3ccccc3)C(=O)CC)co1	8.1
324	O=C(N(C1CC[N+](CC1)CCc2ccccc2)c3ccc4n3nco4)CC	8.1
325	O=C(N(C1CC[N+](CC1)CCc2ccccc2)c3ccc(CCC)cc3)CC	8.1
326	O=C(N(C1CC[N+](CC1)CCc2ccccc2)c3csc(SCC)c3)CC	8.1
327	O=C(N(C1CC[N+](CC1)CCc2ccccc2)c3ccc(OC(OC)=O)cc3)CC	8.1
328	O=C(N(n1cccc1)C2CC[N+](CC2)CCc3ccccc3)CC	8.0
329	O=C(N(C1CC[N+](CC1)CCc2ccccc2)C=3C=COCC3)CC	8.0
330	O=C(N(C1CC[N+](CC1)CCc2ccccc2)c3ccc4C=Cc4c3)CC	8.0
331	O=C(N(C1CC[N+](CC1)CCc2ccccc2)C3=CC=CC3)CC	8.0
332	Fc1cc(N(C2CC[N+](CC2)CCc3ccccc3)C(=O)CC)cs1	8.0
333	Fc1ccc(s1)N(C2CC[N+](CC2)CCc3ccccc3)C(=O)CC	8.0
334	O=C(N(C1CC[N+](CC1)CCc2ccccc2)c3cc(sc3)C#C)CC	8.0
335	Clc1cc(N(C2CC[N+](CC2)CCc3ccccc3)C(=O)CC)cs1	8.0
336	Clc1ccc(s1)N(C2CC[N+](CC2)CCc3ccccc3)C(=O)CC	8.0
337	O=C(N(C1CC[N+](CC1)CCc2ccccc2)C3=CCC(=CC3)C)CC	8.0
338	O=C(N(C1CC[N+](CC1)CCc2ccccc2)c3ccc(s3)CC)CC	8.0
339	O=C(N(C1CC[N+](CC1)CCc2ccccc2)c3c(O)nc(O)cc3)CC	8.0
340	O=C(N(C1CC[N+](CC1)CCc2ccccc2)c3ccc(O)cc3)CC	8.0
341	O=C(N(C1CC[N+](CC1)CCc2ccccc2)c3cc(C)co3)CC	8.0
342	O=C(N(C1CC[N+](CC1)CCc2ccccc2)c3coc(c3)C)CC	8.0
343	O=C(N(C1CC[N+](CC1)CCc2ccccc2)c3ccc4c(OCO4)c3)CC	8.0
344	O=C(N(C1CC[N+](CC1)CCc2ccccc2)c3ccc([nH]3)C)CC	8.0
345	O=C(N(C1CC[N+](CC1)CCc2ccccc2)c3csc(c3)CC)CC	8.0
346	O=C(N(C1CC[N+](CC1)CCc2ccccc2)c3ccsc3NC)CC	8.0
347	O=C(N(N1C=CC(C=C1)(C)C)C2CC[N+](CC2)CCc3ccccc3)CC	8.0
348	O=C(N(C1CC[N+](CC1)CCc2ccccc2)c3cccc(NO)c3)CC	8.0
349	O=C(N(C1CC[N+](CC1)CCc2ccccc2)c3ccc(O)c(c3)C)CC	8.0
350	O=C(N(C1CC[N+](CC1)CCc2ccccc2)c3ccc4c(c3)C=CC4)CC	8.0
351	Clc1ccc(N(C2CC[N+](CC2)CCc3ccccc3)C(=O)CC)[nH]1	8.0
352	O=C(N(C1CC[N+](CC1)CCc2ccccc2)c3cn4c(c3)cco4)CC	8.0
353	O=C(N(C1CC[N+](CC1)CCc2ccccc2)c3ccnc(O)c3)CC	8.0
354	O=C(N(C1CC[N+](CC1)CCc2ccccc2)c3ccc(s3)C4CC4)CC	8.0

355	O=C(N(C1CC[N+](CC1)CCc2ccccc2)c3c(N)cc[nH]3)CC	8.0
356	Clc1c(ccc(N(C2CC[N+](CC2)CCc3ccccc3)C(=O)CC)c1)C	8.0
357	O=C(N(C1CC[N+](CC1)CCc2ccccc2)c3ccc(SC)cc3)CC	8.0
358	Oc1c(O)c(ccc1N(C2CC[N+](CC2)CCc3ccccc3)C(=O)CC)C	8.0
359	O=C(N(C1CC[N+](CC1)CCc2ccccc2)c3csc(CCC)c3)CC	8.0
360	O=C(N(C1CC[N+](CC1)CCc2ccccc2)c3ccc(c(c3)C#C)C)CC	8.0
361	O=C(N(C1CC[N+](CC1)CCc2ccccc2)c3cc(CC)co3)CC	8.0
362	O=C(N(C1CC[N+](CC1)CCc2ccccc2)c3ccc(o3)C)CC	8.0
363	FC(F)Oc1ccc(N(C2CC[N+](CC2)CCc3ccccc3)C(=O)CC)cc1	8.0
364	Clc1c(cc(s1)N(C2CC[N+](CC2)CCc3ccccc3)C(=O)CC)C	8.0
365	O=C(N(C1CC[N+](CC1)CCc2ccccc2)c3ccc(s3)C(C)C)CC	8.0
366	O=C(N(C1CC[N+](CC1)CCc2ccccc2)c3c[nH+]c4c(CCN4)c3)CC	8.0
367	O=C(N(C1CC[N+](CC1)CCc2ccccc2)C(C=C)=C)CC	8.0
368	Oc1cc(ccc1N(C2CC[N+](CC2)CCc3ccccc3)C(=O)CC)C#C	8.0
369	O=C(N(C1CC[N+](CC1)CCc2ccccc2)c3cc4n(c3)ccs4)CC	8.0
370	O=C(N(C1CC[N+](CC1)CCc2ccccc2)c3cc(c(s3)CC)C)CC	8.0
371	O=C(N(C1CC[N+](CC1)CCc2ccccc2)c3ccc(c(N)c3)C#C)CC	8.0
372	O=C(N(C1CC[N+](CC1)CCc2ccccc2)c3coc4cocc43)CC	8.0
373	Oc1c2ccoc2ccc1N(C3CC[N+](CC3)CCc4ccccc4)C(=O)CC	8.0
374	O=C(N(C1CC[N+](CC1)CCc2ccccc2)c3cc(C4CC4)cs3)CC	8.0
375	O=C(N(C1CC[N+](CC1)CCc2ccccc2)c3cc(sc3)C(C)C)CC	8.0
376	Br/C=C(\Cl)N(C1CC[N+](CC1)CCc2ccccc2)C(=O)CC	8.0
377	O=C(N(C1CC[N+](CC1)CCc2ccccc2)c3ccc(C(C)C)cc3)CC	8.0
378	O=C(N(C1CC[N+](CC1)CCc2ccccc2)c3ccc4c(c3)cns4)CC	8.0
379	O=C(N(C1CC[N+](CC1)CCc2ccccc2)c3cnc4c(c3)cc[nH]4)CC	8.0
380	O=C(N(C1CC[N+](CC1)CCc2ccccc2)C3=C(OCC3)N)CC	8.0
381	O=C(N(C1CC[N+](CC1)CCc2ccccc2)c3c(oc(c3)C)N)CC	8.0
382	Oc1cc(N(C2CC[N+](CC2)CCc3ccccc3)C(=O)CC)cc4cc[nH]c14	8.0
383	O=C(N(C1CC[N+](CC1)CCc2ccccc2)c3ccc(c(c3)CC)C)CC	8.0
384	O=C(N(C1CC[N+](CC1)CCc2ccccc2)c3c(cc(s3)C)C)CC	8.0
385	O=C(N(C1CC[N+](CC1)CCc2ccccc2)c3ccn4cccc4c3)CC	8.0
386	O=C(N(C1CC[N+](CC1)CCc2ccccc2)c3ccc4c([C@@H]5C[C@@H]5C4)c3)CC	8.0
387	Sc1cnc(O)c(N(C2CC[N+](CC2)CCc3ccccc3)C(=O)CC)c1	8.0
388	O=C(N(C1CC[N+](CC1)CCc2ccccc2)c3ccccc3N)CC	7.9
389	Fc1ccc(N(C2CC[N+](CC2)CCc3ccccc3)C(=O)CC)cc1	7.9
390	O=C(N(C1CC[N+](CC1)CCc2ccccc2)c3ccc(cc3)C#C)CC	7.9
391	O=C(N(C1CC[N+](CC1)CCc2ccccc2)c3ccc(o3)O)CC	7.9
392	O=C(N(C1CC[N+](CC1)CCc2ccccc2)c3ccc(s3)O)CC	7.9
393	Oc1c([nH+]cc(N(C2CC[N+](CC2)CCc3ccccc3)C(=O)CC)c1)N	7.9
394	O=C(N(C1CC[N+](CC1)CCc2ccccc2)c3ccsc3C)CC	7.9
395	O=C(N(C1CC[N+](CC1)CCc2ccccc2)c3ccoc3)CC	7.9
396	O=C(N(C1CC[N+](CC1)CCc2ccccc2)c3ccc(s3)C#C)CC	7.9

397	Fc1ccc(N(C2CC[N+](CC2)CCc3ccccc3)C(=O)CC)cc1C	7.9
398	O=C(N(n1cc(c(c1)C)C)C2CC[N+](CC2)CCc3ccccc3)CC	7.9
399	Fc1c(ccc(N(C2CC[N+](CC2)CCc3ccccc3)C(=O)CC)c1)C	7.9
400	O=C(N(C1CC[N+](CC1)CCc2ccccc2)c3ccc(s3)SC)CC	7.9
401	Clc1cccn1N(C2CC[N+](CC2)CCc3ccccc3)C(=O)CC	7.9
402	Brc1coc(N(C2CC[N+](CC2)CCc3ccccc3)C(=O)CC)c1	7.9
403	O=C(N(C1CC[N+](CC1)CCc2ccccc2)c3ccc4c(c3)cco4)CC	7.9
404	O=C(N(C1CC[N+](CC1)CCc2ccccc2)c3ccc(cc3)C=C)CC	7.9
405	Clc1ccc(N(C2CC[N+](CC2)CCc3ccccc3)C(=O)CC)cc1C	7.9
406	O=C(N(C1CC[N+](CC1)CCc2ccccc2)c3ccc(s3)OC)CC	7.9
407	Fc1ccc(N(C2CC[N+](CC2)CCc3ccccc3)C(=O)CC)c(O)c1	7.9
408	O=C(N(C1CC[N+](CC1)CCc2ccccc2)c3ccc(cc3N)C)CC	7.9
409	Clc1c(sc(N(C2CC[N+](CC2)CCc3ccccc3)C(=O)CC)c1)C	7.9
410	O=C(N(C1CC[N+](CC1)CCc2ccccc2)C3=C[C@H]4CC[C@H](O4)C3)CC	7.9
411	O=C(N(C1CC[N+](CC1)CCc2ccccc2)c3ccc4C=CCc4c3)CC	7.9
412	Brc1ccc(N(C2CC[N+](CC2)CCc3ccccc3)C(=O)CC)[nH]1	7.9
413	SCc1ccc(s1)N(C2CC[N+](CC2)CCc3ccccc3)C(=O)CC	7.9
414	O=C(N(C1CC[N+](CC1)CCc2ccccc2)c3ccc(cc3)C#CC)CC	7.9
415	O=C(N(C1CC[N+](CC1)CCc2ccccc2)c3ccc(NO)cc3)CC	7.9
416	O=C(N(C1CC[N+](CC1)CCc2ccccc2)c3csc(C4CC4)c3)CC	7.9
417	O=C(N(C1CC[N+](CC1)CCc2ccccc2)c3ccc(C4CC4)cc3)CC	7.9
418	Sc1c(ccc(N(C2CC[N+](CC2)CCc3ccccc3)C(=O)CC)c1)C	7.9
419	O=C(N(C1CC[N+](CC1)CCc2ccccc2)c3ccsc3CC)CC	7.9
420	O=C(N(C1CC[N+](CC1)CCc2ccccc2)c3ccc[nH+]c3NN)CC	7.9
421	O=C(N(C1CC[N+](CC1)CCc2ccccc2)c3cc(sc3)SC)CC	7.9
422	O=C(N(C1CC[N+](CC1)CCc2ccccc2)c3ccc4ccsc4c3)CC	7.9
423	O=C(N(C1CC[N+](CC1)CCc2ccccc2)c3cccc4CCNc43)CC	7.9
424	FC(F)c1csc(N(C2CC[N+](CC2)CCc3ccccc3)C(=O)CC)c1	7.9
425	O=C(N(C1CC[N+](CC1)CCc2ccccc2)c3ccc(c(c3)C)C#C)CC	7.9
426	O=C(N(C1CC[N+](CC1)CCc2ccccc2)c3ccc4c(oc(O)n4)c3)CC	7.9
427	Sc1cccc(N(C2CC[N+](CC2)CCc3ccccc3)C(=O)CC)c1N	7.9
428	Brc1cc(N(C2CC[N+](CC2)CCc3ccccc3)C(=O)CC)co1	7.9
429	O=C(N(C1CC[N+](CC1)CCc2ccccc2)c3cc4cocc4s3)CC	7.9
430	O=C(N(C1CC[N+](CC1)CCc2ccccc2)/C(C)=C/C)CC	7.9
431	O=C(N(C1CC[N+](CC1)CCc2ccccc2)c3c(c(cs3)C)C)CC	7.9
432	O=C(N(C1CC[N+](CC1)CCc2ccccc2)c3cc4c(s3)CCC4)CC	7.9
433	O=C(N(C1CC[N+](CC1)CCc2ccccc2)c3cc(CC)c(s3)C)CC	7.9
434	O=C(N(C1CC[N+](CC1)CCc2ccccc2)c3c(C)cc[nH]3)CC	7.9
435	Sc1ccc(N(C2CC[N+](CC2)CCc3ccccc3)C(=O)CC)c(N)c1	7.9
436	O=C(N(n1cccc1CC)C2CC[N+](CC2)CCc3ccccc3)CC	7.9
437	Fc1ccc(N(C2CC[N+](CC2)CCc3ccccc3)C(=O)CC)cc1CC	7.9
438	Clc1cc(N(C2CC[N+](CC2)CCc3ccccc3)C(=O)CC)co1	7.9

439	Sc1c(F)ccc(N(C2CC[N+](CC2)CCc3ccccc3)C(=O)CC)c1	7.9
440	O=C(N(C1CC[N+](CC1)CCc2ccccc2)c3cc4ccoc4c(c3)C)CC	7.9
441	O=C(N(C1CC[N+](CC1)CCc2ccccc2)c3cccc4c3cco4)CC	7.9
442	O=C(N(C1CC[N+](CC1)CCc2ccccc2)c3ccc(CC=C)cc3)CC	7.9
443	O=C(N(C1CC[N+](CC1)CCc2ccccc2)c3ccccc3NCC#C)CC	7.9
444	O=C(N(C1CC[N+](CC1)CCc2ccccc2)c3csc4cscc43)CC	7.9
445	Sc1ccc(N(C2CC[N+](CC2)CCc3ccccc3)C(=O)CC)cc1	7.8
446	O=C(N(C1CC[N+](CC1)CCc2ccccc2)C3=CCSCC3)CC	7.8
447	O=C(N(C1CC[N+](CC1)CCc2ccccc2)c3cscc3N)CC	7.8
448	O=C(N([n+]1ccccc1)C2CC[N+](CC2)CCc3ccccc3)CC	7.8
449	Fc1coc(N(C2CC[N+](CC2)CCc3ccccc3)C(=O)CC)c1	7.8
450	O=C(N(C1CC[N+](CC1)CCc2ccccc2)C3=CC=C(CC3)C)CC	7.8
451	O=C(N(C1CC[N+](CC1)CCc2ccccc2)c3ccc4cscc4c3)CC	7.8
452	O=C(N(C1CC[N+](CC1)CCc2ccccc2)c3ccc(s3)C=C)CC	7.8
453	O=C(N(C1CC[N+](CC1)CCc2ccccc2)c3csc(c3)CO)CC	7.8
454	O=C(N(C1CC[N+](CC1)CCc2ccccc2)c3cccc(c3NN)C)CC	7.8
455	O=C(N(C1CC[N+](CC1)CCc2ccccc2)c3ccc(O)c(O)n3)CC	7.8
456	O=C(N(C1CC[N+](CC1)CCc2ccccc2)c3ccc4c(c3)ccs4)CC	7.8
457	O=C(N(C1CC[N+](CC1)CCc2ccccc2)c3cc4cc[nH]c4o3)CC	7.8
458	FC(F)c1ccc(N(C2CC[N+](CC2)CCc3ccccc3)C(=O)CC)cc1	7.8
459	F[C@H](c1ccc(N(C2CC[N+](CC2)CCc3ccccc3)C(=O)CC)cc1)C	7.8
460	O=C(N(C1CC[N+](CC1)CCc2ccccc2)c3ccc(CC)c(O)c3)CC	7.8
461	Fc1cc(N(C2CC[N+](CC2)CCc3ccccc3)C(=O)CC)ccc1CC	7.8
462	O=C(N(C1CC[N+](CC1)CCc2ccccc2)c3ccc(s3)SCC)CC	7.8
463	O=C(N(C1CC[N+](CC1)CCc2ccccc2)c3ccc4c(CCO4)c3)CC	7.8
464	FCc1cccc(N(C2CC[N+](CC2)CCc3ccccc3)C(=O)CC)c1NN	7.8
465	O=C(N(C1CC[N+](CC1)CCc2ccccc2)c3ccc4c(ocn4)c3)CC	7.8
466	O=C(N(n1cc2c(c1)cc[nH]2)C3CC[N+](CC3)CCc4ccccc4)CC	7.8
467	Sc1c(O)c(N(C2CC[N+](CC2)CCc3ccccc3)C(=O)CC)ccn1	7.8
468	FCC1(N(C2CC[N+](CC2)CCc3ccccc3)C(=O)CC)CC1	7.8
469	O=C(N(C1CC[N+](CC1)CCc2ccccc2)c3ccc4c(CCS4)c3)CC	7.8
470	O=C(N(C1CC[N+](CC1)CCc2ccccc2)c3cc4c(s3)CSC4)CC	7.8
471	O=C(N(C1CC[N+](CC1)CCc2ccccc2)c3cc4c(s3)ccs4)CC	7.8
472	O=C(N(N1CCS[C@H](C1)C)C2CC[N+](CC2)CCc3ccccc3)CC	7.8
473	Oc1cnccc1N(C2CC[N+](CC2)CCc3ccccc3)C(=O)CC	7.7
474	O=C(N([n+]1ccccc1N)C2CC[N+](CC2)CCc3ccccc3)CC	7.7
475	O=C(N(C1CC[N+](CC1)CCc2ccccc2)C3=C(SCC3)N)CC	7.7
476	SOc1ccc(N(C2CC[N+](CC2)CCc3ccccc3)C(=O)CC)cc1	7.7
477	O=C(N(C1CC[N+](CC1)CCc2ccccc2)c3ccc4COCc4c3)CC	7.7
478	O=C(N(C1CC[N+](CC1)CCc2ccccc2)C3=CCC[N+](C3)C)CC	7.7
479	O=C(N(n1cccc1C)C2CC[N+](CC2)CCc3ccccc3)CC	7.7
480	O=C(N(n1cc2c(c1)cco2)C3CC[N+](CC3)CCc4ccccc4)CC	7.7

481	O=C(N(C1CC[N+](CC1)CCc2ccccc2)c3cccn3N)CC	7.7
482	O=C(N(C1CC[N+](CC1)CCc2ccccc2)c3ccnc(c3)C)CC	7.7
483	FCC(N(C1CC[N+](CC1)CCc2ccccc2)C(=O)CC)=C	7.7
484	O=C(N(C1CC[N+](CC1)CCc2ccccc2)c3ccc4cccn4c3)CC	7.7
485	O=C(N(C1CC[N+](CC1)CCc2ccccc2)c3ccc(cc3)C(C)=C)CC	7.7
486	FC(c1ccc(N(C2CC[N+](CC2)CCc3ccccc3)C(=O)CC)cc1)=C	7.7
487	O=C(N(C1CC[N+](CC1)CCc2ccccc2)c3ccc4cnsc4c3)CC	7.7
488	Oc1c2c(ccc1N(C3CC[N+](CC3)CCc4ccccc4)C(=O)CC)cc[nH]2	7.7
489	O=C(N(C1CC[N+](CC1)CCc2ccccc2)c3c(sc(c3)CC)N)CC	7.7
490	O=C(N(C1CC[N+](CC1)CCc2ccccc2)c3ccc(s3)OCC)CC	7.7
491	O=C(N(C1CC[N+](CC1)CCc2ccccc2)c3ccncc3)CC	7.6
492	O=C(N(C1CC[N+](CC1)CCc2ccccc2)c3ccc(ON)cc3)CC	7.6
493	O=C(N(n1c(cc(c1)C)C)C2CC[N+](CC2)CCc3ccccc3)CC	7.6
494	O=C(N(C1CC[N+](CC1)CCc2ccccc2)c3ccc4n3ccs4)CC	7.6
495	O=C(N(C1CC[N+](CC1)CCc2ccccc2)c3cc4cscc4s3)CC	7.6
496	Fc1ccc(N(C2CC[N+](CC2)CCc3ccccc3)C(=O)CC)c(N)c1	7.5
497	Clc1ccc(N(C2CC[N+](CC2)CCc3ccccc3)C(=O)CC)c(N)c1	7.5
498	O=C(N(C1CC[N+](CC1)CCc2ccccc2)c3ccc([S@](=O)N)cc3)CC	7.5
499	O=C(N(C1CC[N+](CC1)CCc2ccccc2)C3=CCC[S@+](C3)C)CC	7.4
500	O=C(N([n+]1cccc(c1N)C)C2CC[N+](CC2)CCc3ccccc3)CC	7.2

Table S5

List, SMILE and predicted pK_i values for Series 3.

N°	SMILES	Pred pK_i
1	Clc1cc2c(nsn2)c(n1)C3CC[N+](CC3)CCc4ccccc4	8.9
2	O=C1C=Cc2c(N1C3CC[N+](CC3)CCc4ccccc4)nsn2	8.8
3	O=C(C[C@H](C1CC[N+](CC1)CCc2ccccc2)c3ccccc3)C	8.5
4	O=C1N=Cc2c(N1C3CC[N+](CC3)CCc4ccccc4)nccn2	8.5
5	O=C1C=NN2C(SC=N2)=C1C3CC[N+](CC3)CCc4ccccc4	8.5
6	O=C1N=C(C2CC[N+](CC2)CCc3ccccc3)c4c(S1)nccn4	8.5
7	[N+]1(CCC(CC1)/C(c2ccccc2)=C/c3ccccn3)CCc4ccccc4	8.4
8	O=C(/C=C(/C1CC[N+](CC1)CCc2ccccc2)c3ccccc3)C	8.4
9	O=C1C[C@]2(N1C3CC[N+](CC3)CCc4ccccc4)CSCCC2	8.4
10	O=C1C[C@@]2(N1C3CC[N+](CC3)CCc4ccccc4)CCC[C@H](C2)C	8.4
11	O=C(C[C@H](C1CC[N+](CC1)CCc2ccccc2)c3cccs3)C	8.4
12	Cl[C@@H]1C(=O)N(C2CC[N+](CC2)CCc3ccccc3)[C@H]1c4ccco4	8.4
13	O=C1C=NN2C=CSC2=C1C3CC[N+](CC3)CCc4ccccc4	8.4
14	O=C1C=Nc2c(N1C3CC[N+](CC3)CCc4ccccc4)csn2	8.4
15	O=C(N)c1cc2ccccc2c(n1)C3CC[N+](CC3)CCc4ccccc4	8.4
16	Cc1nc(C2CC[N+](CC2)CCc3ccccc3)c(o1)OCC	8.4
17	Clc1cc2c(nccn2)c(n1)C3CC[N+](CC3)CCc4ccccc4	8.4

18	O=C(N(C1CC[N+](CC1)CCc2ccccc2)c3ccccc3)C4CC4	8.3
19	O=C(N(C1CC[N+](CC1)CCc2ccccc2)c3ccccc3)C(C)C	8.3
20	Fc1ccccc1N(C2CC[N+](CC2)CCc3ccccc3)C(=O)C	8.3
21	O=C(N(C1CC[N+](CC1)CCc2ccccc2)C3CCCCC3)C	8.3
22	O=C(N(C1CC[N+](CC1)CCc2ccccc2)c3ccc(N)cc3)C	8.3
23	O=C(N(C1CC[N+](CC1)CCc2ccccc2)c3ccccc3)C=C	8.3
24	F[C@@H](C(=O)N(C1CC[N+](CC1)CCc2ccccc2)c3ccccc3)C	8.3
25	O=C1[C@@](SC)(C2CC[N+](CC2)CCc3ccccc3)c4ccccc4N1	8.3
26	O=C1C=Cc2c(N1C3CC[N+](CC3)CCc4ccccc4)csn2	8.3
27	O=C1C=Nc2c(N1C3CC[N+](CC3)CCc4ccccc4)nco2	8.3
28	O=C1C[C@@]2(N1C3CC[N+](CC3)CCc4ccccc4)CCC[C@H](C2)CC	8.3
29	O=C1C2=C(O[C@@H](C2)C)c3cccnc3N1C4CC[N+](CC4)CCc5ccccc5	8.3
30	Clc1nc(n(n1)[C@H]2CCCCO2)C3CC[N+](CC3)CCc4ccccc4	8.3
31	O=C1C=Cc2ccoc2N1C3CC[N+](CC3)CCc4ccccc4	8.3
32	O=C1C=Nc2c(N1C3CC[N+](CC3)CCc4ccccc4)ncs2	8.3
33	O=C(N(C1CC[N+](CC1)CCc2ccccc2)CC3CC3)C4CC4	8.3
34	Cn1c([C@H](C2CC[N+](CC2)CCc3ccccc3)c4ccccc4)ccn1	8.3
35	O=C1C=Cc2c(N1C3CC[N+](CC3)CCc4ccccc4)cc[nH]2	8.3
36	O=C(N(C1CC[N+](CC1)CCc2ccccc2)C3CCCC3)CC	8.2
37	Fc1cccc(N(C2CC[N+](CC2)CCc3ccccc3)C(=O)C)c1	8.2
38	Fc1ccccc1N(C2CC[N+](CC2)CCc3ccccc3)C(=O)CC	8.2
39	O=C(N(C1CC[N+](CC1)CCc2ccccc2)C3CCCC3)C	8.2
40	Clc1ccccc1N(C2CC[N+](CC2)CCc3ccccc3)C(=O)CC	8.2
41	O=C1C[C@@]2(N1C3CC[N+](CC3)CCc4ccccc4)CCC[C@@H]5C[C@@H]52	8.2
42	O=C(N(C1CC[N+](CC1)CCc2ccccc2)c3ccccn3)CC	8.2
43	C[C@H]([C@H](C1CC[N+](CC1)CCc2ccccc2)c3ccccc3)CC	8.2
44	[N+]1(CCC(CC1)/C(=N/c2ccccc2)c3ccccc3)CCc4ccccc4	8.2
45	O=C(C[C@H](C1CC[N+](CC1)CCc2ccccc2)c3cccc(c3)C)C	8.2
46	Fc1ccccc1C2(C3CC[N+](CC3)CCc4ccccc4)CCC(O)CC2	8.2
47	O=C1C=Cc2ccccc2N1C3CC[N+](CC3)CCc4ccccc4	8.2
48	CC(c1c(nc(o1)C)C2CC[N+](CC2)CCc3ccccc3)C	8.2
49	O=C1CC2(N1C3CC[N+](CC3)CCc4ccccc4)CCCCC2	8.2
50	O=C1N(C2CC[N+](CC2)CCc3ccccc3)c4c(S1)nsn4	8.2
51	O=C(N(C1CC[N+](CC1)CCc2ccccc2)C3CC3)CC(=O)C	8.2
52	O=C1[C@](C2CC[N+](CC2)CCc3ccccc3)(CC)c4ccccc4N1	8.2
53	COc1nc2ccsc2c(n1)C3CC[N+](CC3)CCc4ccccc4	8.2
54	COc1cc2ccccc2c(n1)C3CC[N+](CC3)CCc4ccccc4	8.2
55	O=C1C(C2CC[N+](CC2)CCc3ccccc3)=C4C(N1C)=CSS4	8.2
56	O=C1Nc2cscc2C(=N1)C3CC[N+](CC3)CCc4ccccc4	8.2
57	O=C1N=Nc2c(N1C3CC[N+](CC3)CCc4ccccc4)nccn2	8.2
58	O=C1C[C@@]2(N1C3CC[N+](CC3)CCc4ccccc4)CC[C@@H](C[C@H]2C)C	8.2
59	Clc1ccc(OC(F)F)c(n1)C2CC[N+](CC2)CCc3ccccc3	8.2

60	ON1[C@@H]([N+]([O-])=C(C2CC[N+](CC2)CCc3ccccc3)C1(C)C)C	8.2
61	Brc1nn(c(n1)C2CC[N+](CC2)CCc3ccccc3)CC	8.2
62	O=C(N(C1CC[N+](CC1)CCc2ccccc2)C3CCCCC3)C4CC4	8.1
63	O=C(N(C1CC[N+](CC1)CCc2ccccc2)c3cc(ccc3C)C)C	8.1
64	Clc1cccc(N(C2CC[N+](CC2)CCc3ccccc3)C(=O)C)c1	8.1
65	O=C1C[C@@]2(N1C3CC[N+](CC3)CCc4ccccc4)CC[C@@H]5C[C@@H]52	8.1
66	O=C1C2(N(C3CC[N+](CC3)CCc4ccccc4)C(=O)N1)CCCCC2	8.1
67	O=C(N(C1CC[N+](CC1)CCc2ccccc2)C(C)C)C	8.1
68	O=C1CCC2(N1C3CC[N+](CC3)CCc4ccccc4)CC[N+]CC2	8.1
69	Fc1cc(OC(F)F)cc(n1)C2CC[N+](CC2)CCc3ccccc3	8.1
70	O=C(N(C1CC[N+](CC1)CCc2ccccc2)C3CCCC3)C4CC4	8.1
71	O=C(N)[C@]1(C2CC[N+](CC2)CCc3ccccc3)c4ccccc4CC1	8.1
72	O=C1CC2(N1C3CC[N+](CC3)CCc4ccccc4)CCC2	8.1
73	OC[C@@H](C1CC[N+](CC1)CCc2ccccc2)c3ccccc3	8.1
74	O=C(N(C1CC[N+](CC1)CCc2ccccc2)C(C)C)[C@@H]3[C@H](C3)C	8.1
75	CS[C@@H](C1CC[N+](CC1)CCc2ccccc2)c3ccccc3	8.1
76	O=C(N(C1CC[N+](CC1)CCc2ccccc2)CC(C)C)C	8.1
77	O=C1N(C2CC[N+](CC2)CCc3ccccc3)c4c(S1)csn4	8.1
78	Clc1ccccc1N(C2CC[N+](CC2)CCc3ccccc3)C(=O)C	8.1
79	O=C(N(OC(=O)C)C1CC[N+](CC1)CCc2ccccc2)C	8.1
80	O=C1C[C@]2(N1C3CC[N+](CC3)CCc4ccccc4)CCOCC2	8.1
81	CC(C[C@H](C1CC[N+](CC1)CCc2ccccc2)c3ccccc3)C	8.1
82	Clc1cc(OC(F)F)cc(n1)C2CC[N+](CC2)CCc3ccccc3	8.1
83	O=C1N(C2CC[N+](CC2)CCc3ccccc3)c4c(S1)scn4	8.1
84	O=C1C[C@@]2(N1C3CC[N+](CC3)CCc4ccccc4)CCC[C@H]2C	8.1
85	O=C1C[C@H](N1C2CC[N+](CC2)CCc3ccccc3)c4cscn4	8.1
86	Fc1ccc(OCF)c(n1)C2CC[N+](CC2)CCc3ccccc3	8.1
87	O=C1CC[C@H](N1C2CC[N+](CC2)CCc3ccccc3)CC	8.1
88	O=S(=O)(N(C1CC[N+](CC1)CCc2ccccc2)C(=O)C)C	8.1
89	O=C1C=Nc2ccoc2N1C3CC[N+](CC3)CCc4ccccc4	8.1
90	Clc1cccc([C@@H](C2CC[N+](CC2)CCc3ccccc3)CC(=O)C)c1	8.1
91	O=C(N(C1CC[N+](CC1)CCc2ccccc2)CCC)[C@@H]3[C@@H](C3)C	8.1
92	Clc1nc2c(nc(s2)C)c(n1)C3CC[N+](CC3)CCc4ccccc4	8.1
93	Clc1cc2c(c(n1)C3CC[N+](CC3)CCc4ccccc4)cco2	8.1
94	O=C1C=Nn2n(scco2)N1C3CC[N+](CC3)CCc4ccccc4	8.1
95	Sc1cc2ccccc2c(n1)C3CC[N+](CC3)CCc4ccccc4	8.1
96	CC(c1nc(nn1C2CC[N+](CC2)CCc3ccccc3)C)C	8.1
97	O=C1C[C@]2(N1C3CC[N+](CC3)CCc4ccccc4)CCCC[C@@H]2OC	8.1
98	Clc1cc2ccsc2c(n1)C3CC[N+](CC3)CCc4ccccc4	8.1
99	O=C(N(C1CC[N+](CC1)CCc2ccccc2)c3ccccn3)C	8.1
100	Clc1nc(C2CC[N+](CC2)CCc3ccccc3)c(s1)C	8.1
101	O=C(N(C1CC[N+](CC1)CCc2ccccc2)C(=O)CC)CC	8.1

102	O=C(N(C1CC[N+](CC1)CCc2ccccc2)CC)N(C)C	8.1
103	O=C1N=Cn2cccc2N1C3CC[N+](CC3)CCc4ccccc4	8.1
104	Fc1ccc(OC(F)F)c(n1)C2CC[N+](CC2)CCc3ccccc3	8.1
105	ClC1=CN(C(=O)C(C2CC[N+](CC2)CCc3ccccc3)=C1)C	8.1
106	O=C(N(C1CC[N+](CC1)CCc2ccccc2)C3CC3)c4ccoc4	8.1
107	N#CCc1cc(n(n1)C2CC[N+](CC2)CCc3ccccc3)C(C)C	8.1
108	Fc1ccc(N(C2CC[N+](CC2)CCc3ccccc3)C(=O)CC)cc1	8.0
109	O=C(N(C1CC[N+](CC1)CCc2ccccc2)c3ccccc3)C[N+]	8.0
110	O=C(N(C1CC[N+](CC1)CCc2ccccc2)c3ccccc3)C	8.0
111	COC[C@H](C1CC[N+](CC1)CCc2ccccc2)c3ccccc3	8.0
112	O=C(N(C1CC[N+](CC1)CCc2ccccc2)CCC)C=C	8.0
113	CCC[C@@H](C1CC[N+](CC1)CCc2ccccc2)c3ccccc3	8.0
114	O=C(N(C1CC[N+](CC1)CCc2ccccc2)CCCC)C	8.0
115	O=C(N(C1CC[N+](CC1)CCc2ccccc2)c3cnccc3)C	8.0
116	O=C1CC2(N1C3CC[N+](CC3)CCc4ccccc4)CCCC2	8.0
117	O=C(N(C1CC[N+](CC1)CCc2ccccc2)CCOC)C	8.0
118	O=C1C2(N(C3CC[N+](CC3)CCc4ccccc4)C(=O)N1)CCCC2	8.0
119	O=CC[C@H](C1CC[N+](CC1)CCc2ccccc2)c3ccccc3	8.0
120	O=C1C=Nc2c(N1C3CC[N+](CC3)CCc4ccccc4)cco2	8.0
121	Clc1cc2c(c(n1)C3CC[N+](CC3)CCc4ccccc4)cccn2	8.0
122	O=C1C=Cc2c(N1C3CC[N+](CC3)CCc4ccccc4)ccs2	8.0
123	O=C1CCCc2ccccc2N1C3CC[N+](CC3)CCc4ccccc4	8.0
124	C=C[C@H](C1CC[N+](CC1)CCc2ccccc2)c3ccccc3	8.0
125	O=C(N(C1CC[N+](CC1)CCc2ccccc2)C3CC3)C4CCC4	8.0
126	Clc1ccc(N(C2CC[N+](CC2)CCc3ccccc3)C(=O)C(C)C)cc1	8.0
127	O=C(N(C1CC[N+](CC1)CCc2ccccc2)c3ccc(OC)cc3)CCC	8.0
128	s1cccc1CN(C2CC[N+](CC2)CCc3ccccc3)c4nccs4	8.0
129	O=C(N(C1CC[N+](CC1)CCc2ccccc2)C3CC3)[C@H]4CCCO4	8.0
130	Brc1cc(OC(F)F)cc(n1)C2CC[N+](CC2)CCc3ccccc3	8.0
131	O=C1C=Cc2c(scn2)N1C3CC[N+](CC3)CCc4ccccc4	8.0
132	O=C1N(C2CC[N+](CC2)CCc3ccccc3)c4c(S1)ocn4	8.0
133	CC1CCC(C2CC[N+](CC2)CCc3ccccc3)(CC1)c4ccccc4	8.0
134	Fc1ccccc1[C@@H](C2CC[N+](CC2)CCc3ccccc3)CC	8.0
135	Clc1cc2ccccc2c(n1)C3CC[N+](CC3)CCc4ccccc4	8.0
136	Clc1ccccc1C2(C3CC[N+](CC3)CCc4ccccc4)CCC(O)CC2	8.0
137	O=C1N=Nc2ccccc2N1C3CC[N+](CC3)CCc4ccccc4	8.0
138	O=C(N(C1CC[N+](CC1)CCc2ccccc2)C[C@H]3CCCO3)C	8.0
139	Clc1ccc([C@@H](OCC)C2CC[N+](CC2)CCc3ccccc3)cc1	8.0
140	Fc1ccc(c(n1)C2CC[N+](CC2)CCc3ccccc3)C(F)(F)F	8.0
141	Clc1nc2CCCc2c(n1)C3CC[N+](CC3)CCc4ccccc4	8.0
142	O=C1C[C@]2(N1C3CC[N+](CC3)CCc4ccccc4)CCC[C@@H]2CC	8.0
143	Clc1cc(OCC)nc(n1)C2CC[N+](CC2)CCc3ccccc3	8.0

144	O=C1C[C@]2(N1C3CC[N+](CC3)CCc4ccccc4)COCCC2	8.0
145	Clc1nc2CCSc2c(n1)C3CC[N+](CC3)CCc4ccccc4	8.0
146	Brc1nc(C2CC[N+](CC2)CCc3ccccc3)c(s1)C4CC4	8.0
147	O=C1C=Nc2c(N1C3CC[N+](CC3)CCc4ccccc4)nccn2	8.0
148	O=C1N(C2CC[N+](CC2)CCc3ccccc3)c4c(S1)cccn4	8.0
149	O=C1CN=C(SCC)N1C2CC[N+](CC2)CCc3ccccc3	8.0
150	O=C(NC)/C=C(/C1CC[N+](CC1)CCc2ccccc2)c3ccccc3	8.0
151	O=C1CSc2cccnc2N1C3CC[N+](CC3)CCc4ccccc4	8.0
152	O=C1C[C@H]2CCCC[C@H]2N1C3CC[N+](CC3)CCc4ccccc4	8.0
153	O=C1C[C@@](N1C2CC[N+](CC2)CCc3ccccc3)(CC)C	8.0
154	O=C1N(C2CC[N+](CC2)CCc3ccccc3)c4csnc4S1	8.0
155	O=C(N(C1CC[N+](CC1)CCc2ccccc2)CC)c3cc[nH]n3	8.0
156	Clc1ccc2c(N(C3CC[N+](CC3)CCc4ccccc4)C(=O)CCS2)c1	8.0
157	FC(F)(F)c1c(nc(o1)C)C2CC[N+](CC2)CCc3ccccc3	8.0
158	O=C1C[C@]2(N1C3CC[N+](CC3)CCc4ccccc4)CCC(C[C@@H]2C)(C)C	8.0
159	O=C1N(C2CC[N+](CC2)CCc3ccccc3)c4ccoc4S1	8.0
160	O=C1C=C(O)c2ccccc2N1C3CC[N+](CC3)CCc4ccccc4	8.0
161	O=C1N=C(C2CC[N+](CC2)CCc3ccccc3)c4cnccc4S1	8.0
162	s1ccnc1N(C2CC[N+](CC2)CCc3ccccc3)Cc4ccco4	8.0
163	O=C(N(C1CC[N+](CC1)CCc2ccccc2)CC)[C@@H]3[C@@H](C3)C	8.0
164	O=C1N(C2CC[N+](CC2)CCc3ccccc3)C=4C(SC=CC4S1)=O	8.0
165	O=S(=O)(c1nc(C2CC[N+](CC2)CCc3ccccc3)c(s1)NCC)C	8.0
166	O=S(=O)(C1CC[N+](CC1)CCc2ccccc2)c3cc[nH]c3	8.0
167	O=C(N(C1CC[N+](CC1)CCc2ccccc2)c3cccc(c3C)C)C	7.9
168	O=C(N(C1CC[N+](CC1)CCc2ccccc2)C3CC3)CC	7.9
169	O=C(N(C1CC[N+](CC1)CCc2ccccc2)c3ccccc3C)CC	7.9
170	Fc1ccccc1CN(C2CC[N+](CC2)CCc3ccccc3)C(=O)C	7.9
171	O=C1C[C@]2(N1C3CC[N+](CC3)CCc4ccccc4)CC[C@@H](C2)C	7.9
172	N#C[C@@H](N(C1CC[N+](CC1)CCc2ccccc2)c3ccccc3)C	7.9
173	O=C(N(C1CC[N+](CC1)CCc2ccccc2)CC(C)C)C=C	7.9
174	O=C(N(C1CC[N+](CC1)CCc2ccccc2)C3CC3)C=C	7.9
175	O=C1C[C@H](N1C2CC[N+](CC2)CCc3ccccc3)c4cccs4	7.9
176	O=C(N(C1CC[N+](CC1)CCc2ccccc2)CCC(C)C)C	7.9
177	O1CC(C2CC[N+](CC2)CCc3ccccc3)(C1)c4ccccc4	7.9
178	CSC[C@H](C1CC[N+](CC1)CCc2ccccc2)c3ccccc3	7.9
179	O=C1[C@H]([C@H](N1C2CC[N+](CC2)CCc3ccccc3)c4ccccc4)CC	7.9
180	O=C1[C@@](OC)(C2CC[N+](CC2)CCc3ccccc3)c4ccccc4N1	7.9
181	Brc1ccc(O)c(C(=S)C2CC[N+](CC2)CCc3ccccc3)c1	7.9
182	O=C(N(C1CC[N+](CC1)CCc2ccccc2)CCC)C3CC3	7.9
183	O=C[C@H](C1CC[N+](CC1)CCc2ccccc2)c3ccccc3	7.9
184	O=C1C[C@@]2(N1C3CC[N+](CC3)CCc4ccccc4)C[C@@H]5CC[C@H]2C5	7.9
185	O=C1CC2(N1C3CC[N+](CC3)CCc4ccccc4)CCCCCC2	7.9

186	FC(F)(F)C(=O)N(C1CC[N+](CC1)CCc2ccccc2)CC	7.9
187	O=C1C[C@]2(N1C3CC[N+](CC3)CCc4ccccc4)CC[C@@H](C2)CC	7.9
188	O=C1CSC2(N1C3CC[N+](CC3)CCc4ccccc4)CCC(CC2)CC	7.9
189	O=C1C2(CC2)c3ccccc3N1C4CC[N+](CC4)CCc5ccccc5	7.9
190	O=C1[C@@H](OC)[C@H](N1C2CC[N+](CC2)CCc3ccccc3)c4ccccc4	7.9
191	O=C1C=Cc2cscc2N1C3CC[N+](CC3)CCc4ccccc4	7.9
192	O=C1CC2(N1C3CC[N+](CC3)CCc4ccccc4)CCC(CC2)C	7.9
193	O=C(N(C1CC[N+](CC1)CCc2ccccc2)CC=C)C3CC3	7.9
194	O=C1N=Nc2cscc2N1C3CC[N+](CC3)CCc4ccccc4	7.9
195	O=C1[C@H](Sc2ccccc2N1C3CC[N+](CC3)CCc4ccccc4)C	7.9
196	ClC(Cl)[C@@H](C1CC[N+](CC1)CCc2ccccc2)c3ccccc3	7.9
197	Clc1ccccc1[C@@H]2[C@H](OC)C(=O)N2C3CC[N+](CC3)CCc4ccccc4	7.9
198	CC(CN(C1CC[N+](CC1)CCc2ccccc2)c3ccccc3)(C)C	7.9
199	Clc1nc(C2CC[N+](CC2)CCc3ccccc3)c(s1)CCC	7.9
200	O=C1C(Sc2ccsc2N1C3CC[N+](CC3)CCc4ccccc4)=O	7.9
201	Clc1ccccc1N(S(=O)(=O)C)C2CC[N+](CC2)CCc3ccccc3	7.9
202	O=C1C[C@](N1C2CC[N+](CC2)CCc3ccccc3)(C4CC4)C	7.9
203	Brc1nc(C2CC[N+](CC2)CCc3ccccc3)c(s1)C(C)C	7.9
204	Clc1nc(C2CC[N+](CC2)CCc3ccccc3)c(s1)CC(C)C	7.9
205	O=C1CC2(N1C3CC[N+](CC3)CCc4ccccc4)CCC(CC2)CC	7.9
206	O=C(N(C1CC[N+](CC1)CCc2ccccc2)C3CC3)C4CC4	7.9
207	Cc1nccn1[C@H](C2CC[N+](CC2)CCc3ccccc3)c4ccccc4	7.9
208	O=C1C[C@@](N1C2CC[N+](CC2)CCc3ccccc3)(CCC)CC	7.9
209	Br[C@H]1Cc2ccccc2[C@@H]1C3CC[N+](CC3)CCc4ccccc4	7.9
210	O=C1C[C@](N1C2CC[N+](CC2)CCc3ccccc3)(C4CC4)CC	7.9
211	O=C(N(C1CC[N+](CC1)CCc2ccccc2)CC)C(C)C	7.9
212	Brc1nc(C2CC[N+](CC2)CCc3ccccc3)c(s1)C	7.9
213	O=C1CC[C@H](N1C2CC[N+](CC2)CCc3ccccc3)C	7.9
214	OC[C@@H](C1CC[N+](CC1)CCc2ccccc2)c3cccc(c3)C	7.9
215	O=C1C[C@](N1C2CC[N+](CC2)CCc3ccccc3)(C4CC4)COC	7.9
216	O=C(N(C1CC[N+](CC1)CCc2ccccc2)CCC)C=C(C)C	7.9
217	O=C1C(C[N+]C)=CC=2CCCC2N1C3CC[N+](CC3)CCc4ccccc4	7.9
218	O=C(N(C1CC[N+](CC1)CCc2ccccc2)Cc3c[nH]cn3)C	7.9
219	O=C1C=NN2C(=C1C3CC[N+](CC3)CCc4ccccc4)C=NC=N2	7.9
220	Fc1ccc(SC)c(n1)C2CC[N+](CC2)CCc3ccccc3	7.9
221	ClC1=C(C2CC[N+](CC2)CCc3ccccc3)C(=O)N(C1)C	7.9
222	Fc1ccccc1[C@H](C2CC[N+](CC2)CCc3ccccc3)CC=C	7.9
223	ClC1=CC(=CN(C2CC[N+](CC2)CCc3ccccc3)C1=O)C(F)(F)F	7.9
224	O=C1C[C@@](N1C2CC[N+](CC2)CCc3ccccc3)(CC4CC4)C	7.9
225	Clc1cccc2c1N(C3CC[N+](CC3)CCc4ccccc4)C(S2)=O	7.9
226	O=C1C=Nc2c(scn2)N1C3CC[N+](CC3)CCc4ccccc4	7.9
227	O=C(N(C1CC[N+](CC1)CCc2ccccc2)c3ccccc3)CC	7.8

228	Brc1cccc(N(C2CC[N+](CC2)CCc3ccccc3)C(=O)C)c1	7.8
229	O=C(OC)N(C1CC[N+](CC1)CCc2ccccc2)CCC	7.8
230	O=C(N(C1CC[N+](CC1)CCc2ccccc2)C3CCCCC3)C(C)C	7.8
231	O=C1CC2(N1C3CC[N+](CC3)CCc4ccccc4)CCSCC2	7.8
232	C[C@@](OC)(C1CC[N+](CC1)CCc2ccccc2)c3ccccc3	7.8
233	O=C(N(C1CC[N+](CC1)CCc2ccccc2)C(C)C)C=C	7.8
234	Cc1csc(N(C2CC[N+](CC2)CCc3ccccc3)c4ccccc4)n1	7.8
235	O=C(N(C1CC[N+](CC1)CCc2ccccc2)c3ccco3)C	7.8
236	OC1([C@H](C2CC[N+](CC2)CCc3ccccc3)c4ccccc4)CCC1	7.8
237	Clc1ccc(O)c(C(=S)C2CC[N+](CC2)CCc3ccccc3)c1	7.8
238	Fc1ccccc1C2(C3CC[N+](CC3)CCc4ccccc4)COC2	7.8
239	O=C1C[C@]2(N1C3CC[N+](CC3)CCc4ccccc4)CC[C@H]([C@@H](C2)C)C	7.8
240	C[C@](C1CC[N+](CC1)CCc2ccccc2)(CCC)c3ccccc3	7.8
241	O=C1N(C2CC[N+](CC2)CCc3ccccc3)c4c(S1)nccn4	7.8
242	O=C1N(C2CC[N+](CC2)CCc3ccccc3)c4ccccc4C(S1)=O	7.8
243	O=C1C[C@H](N1C2CC[N+](CC2)CCc3ccccc3)c4ccco4	7.8
244	Clc1ccc([C@@H](C2CC[N+](CC2)CCc3ccccc3)CC(=O)C)cc1	7.8
245	O=C(N(C1CC[N+](CC1)CCc2ccccc2)[C@@H](CC)C)C	7.8
246	O=C1C=Nc2ccsc2N1C3CC[N+](CC3)CCc4ccccc4	7.8
247	O=C(N(C1CC[N+](CC1)CCc2ccccc2)CC(C)C)CC	7.8
248	O=C1C[C@@]2(N1C3CC[N+](CC3)CCc4ccccc4)CCC(C2)(C)C	7.8
249	O=C1N(C2CC[N+](CC2)CCc3ccccc3)c4c(S1)ncs4	7.8
250	O=C1N=Cc2c(N1C3CC[N+](CC3)CCc4ccccc4)cncn2	7.8
251	Oc1ccccc1SC2CC[N+](CC2)CCc3ccccc3	7.8
252	O=S(=O)(N(C1CC[N+](CC1)CCc2ccccc2)C(=O)CC)C	7.8
253	O=C(N(C1CC[N+](CC1)CCc2ccccc2)CC=C)CC	7.8
254	Fc1cc2ccccc2c(n1)C3CC[N+](CC3)CCc4ccccc4	7.8
255	O=C(N(C1CC[N+](CC1)CCc2ccccc2)C3CC3)C(C)C	7.8
256	Cl[C@H]1C(=O)N(C2CC[N+](CC2)CCc3ccccc3)[C@@H]1c4cccs4	7.8
257	O=C(N(C1CC[N+](CC1)CCc2ccccc2)c3nc(c(o3)C)C)C	7.8
258	O=C1COc2ccsc2N1C3CC[N+](CC3)CCc4ccccc4	7.8
259	Fc1ccc(N(C2CC[N+](CC2)CCc3ccccc3)C(=O)C(C)C)cc1	7.8
260	Clc1ccccc1[C@H]2N(C3CC[N+](CC3)CCc4ccccc4)C(=O)CS2	7.8
261	O=C1CC2(N1C3CC[N+](CC3)CCc4ccccc4)C[C@H](C[C@@H](C2)C)C	7.8
262	O=C(N(C1CC[N+](CC1)CCc2ccccc2)CC)CSC	7.8
263	O=C1NC(=O)[C@]2(N1C3CC[N+](CC3)CCc4ccccc4)CCC[C@@H](C2)C	7.8
264	O=C(N(C1CC[N+](CC1)CCc2ccccc2)CC)C#C	7.8
265	O=C1NN=C(SCC=C)N1C2CC[N+](CC2)CCc3ccccc3	7.8
266	O=C1N(C2CC[N+](CC2)CCc3ccccc3)C4=C(S1)CCCC4	7.8
267	O=C([C@]1(C2CC[N+](CC2)CCc3ccccc3)C(=N1)C)C	7.8
268	O=C1N=C(C2CC[N+](CC2)CCc3ccccc3)c4cncnc4S1	7.8
269	O=C(N(C1CC1)C2CC[N+](CC2)CCc3ccccc3)c4ccccc4	7.8

270	O[C@@H]1CCCc2ccccc2[C@H]1C3CC[N+](CC3)CCc4ccccc4	7.8
271	O=C1C[C@@]2(N1C3CC[N+](CC3)CCc4ccccc4)CC[C@@H](CCC2)C	7.8
272	SCC1=CC=2CCCCC2N(C3CC[N+](CC3)CCc4ccccc4)C1=O	7.8
273	Clc1cccc(C2(C3CC[N+](CC3)CCc4ccccc4)CCC(O)CC2)c1	7.8
274	O=C1N(N=C(N1C2CC[N+](CC2)CCc3ccccc3)CCCC)C	7.8
275	s1cc2ccnc(C3CC[N+](CC3)CCc4ccccc4)c2c1	7.8
276	O=C1N=Nc2ccnnc2N1C3CC[N+](CC3)CCc4ccccc4	7.8
277	Sc1cc(OC(F)F)cc(n1)C2CC[N+](CC2)CCc3ccccc3	7.8
278	Fc1c(cc(OC(F)(F)F)c(n1)C2CC[N+](CC2)CCc3ccccc3)C	7.8
279	O=C1C[C@](N1C2CC[N+](CC2)CCc3ccccc3)(C(C)C)C	7.8
280	Brc1cc(F)nc(C2CC[N+](CC2)CCc3ccccc3)c1	7.8
281	O=C(N(C1CC[N+](CC1)CCc2ccccc2)C3CC3)c4cc(no4)C	7.8
282	O=C1C[C@H](N1C2CC[N+](CC2)CCc3ccccc3)C4CC4	7.8
283	ClC(Cl)C(=O)N(C1CC[N+](CC1)CCc2ccccc2)CC=C	7.8
284	FC(F)(F)CN(C1CC[N+](CC1)CCc2ccccc2)C(=O)C	7.8
285	Clc1ccc(F)c(n1)C2CC[N+](CC2)CCc3ccccc3	7.8
286	O=C(N(C1CC[N+](CC1)CCc2ccccc2)C3CC3)C4CCCC4	7.8
287	Fc1cc(OCF)cc(n1)C2CC[N+](CC2)CCc3ccccc3	7.8
288	OCCN(C1CC[N+](CC1)CCc2ccccc2)C(=O)C=C	7.8
289	s1c(C2CC[N+](CC2)CCc3ccccc3)c4c(snn4)n1	7.8
290	Clc1cc2c(cccc2c(n1)C3CC[N+](CC3)CCc4ccccc4)C	7.8
291	Clc1cc2ccc(F)cc2c(n1)C3CC[N+](CC3)CCc4ccccc4	7.8
292	O[C@H](C1CC[N+](CC1)CCc2ccccc2)c3cc[nH]c3	7.8
293	Clc1cc2ccc(cc2c(n1)C3CC[N+](CC3)CCc4ccccc4)C	7.8
294	O=C(N(n1cccc1)C2CC[N+](CC2)CCc3ccccc3)C	7.7
295	O=C(N(C1CC[N+](CC1)CCc2ccccc2)c3ccc(CC)cc3)C	7.7
296	Brc1ccc(N(C2CC[N+](CC2)CCc3ccccc3)C(=O)C)cc1	7.7
297	Clc1ccc(N(C2CC[N+](CC2)CCc3ccccc3)C(=O)CC)cc1	7.7
298	S=C(C1CC[N+](CC1)CCc2ccccc2)c3ccccc3O	7.7
299	O1CC[C@](C2CC[N+](CC2)CCc3ccccc3)(C1)c4ccccc4	7.7
300	S(C1CC[N+](CC1)CCc2ccccc2)c3ccccc3	7.7
301	O=C(N(C1CC[N+](CC1)CCc2ccccc2)c3ccc4c(OCCO4)c3)C	7.7
302	O=CN(C1CC[N+](CC1)CCc2ccccc2)C3CCCC3	7.7
303	O=C(OC)N(C1CC[N+](CC1)CCc2ccccc2)CC	7.7
304	O=C1N(C2CC[N+](CC2)CCc3ccccc3)c4ccsc4S1	7.7
305	O=C(N(C1CC[N+](CC1)CCc2ccccc2)CC3CC3)C	7.7
306	O=C(N(C1CC[N+](CC1)CCc2ccccc2)CCC)NC	7.7
307	FC(F)(C1CC[N+](CC1)CCc2ccccc2)c3ccccc3	7.7
308	Cl[C@](F)(C1CC[N+](CC1)CCc2ccccc2)c3ccccc3	7.7
309	C#CCN(C1CC[N+](CC1)CCc2ccccc2)c3ccccc3	7.7
310	O=C(N(C1CC[N+](CC1)CCc2ccccc2)CC)C3CC3	7.7
311	O=C1CC2(N1C3CC[N+](CC3)CCc4ccccc4)Cc5ccccc5C2	7.7

312	O=C(OC)N(C1CC[N+](CC1)CCc2ccccc2)COC	7.7
313	CCCC[C@H](C1CC[N+](CC1)CCc2ccccc2)c3ccsc3	7.7
314	O=C1CC2(N1C3CC[N+](CC3)CCc4ccccc4)CCOCC2	7.7
315	Fc1ccccc1[S@](=O)C2CC[N+](CC2)CCc3ccccc3	7.7
316	O=C1N(C2CC[N+](CC2)CCc3ccccc3)c4c(S1)cco4	7.7
317	O=C(N(C1CC[N+](CC1)CCc2ccccc2)CCC)C	7.7
318	SCC1=CC=2CCCC2N(C3CC[N+](CC3)CCc4ccccc4)C1=O	7.7
319	O=C1N(C2CC[N+](CC2)CCc3ccccc3)c4ccccc4C(O1)=O	7.7
320	Clc1ccccc1[S@](=O)C2CC[N+](CC2)CCc3ccccc3	7.7
321	Fc1ccc2C(=CC(=O)N(C3CC[N+](CC3)CCc4ccccc4)c2c1)C	7.7
322	O=C1C[C@]2(N1C3CC[N+](CC3)CCc4ccccc4)CCOC[C@@H]2C	7.7
323	O=C(N(C1CC[N+](CC1)CCc2ccccc2)CCC)CC	7.7
324	O=C(N(C1CC[N+](CC1)CCc2ccccc2)CC)c3ccon3	7.7
325	O=C(N(C1CC[N+](CC1)CCc2ccccc2)C3=NCCCS3)C	7.7
326	O=C1N(C2CC[N+](CC2)CCc3ccccc3)c4c(S1)nco4	7.7
327	O=C1N(c2c(N1C3CC[N+](CC3)CCc4ccccc4)cccn2)C(C)C	7.7
328	O=C(N(OC)C1CC[N+](CC1)CCc2ccccc2)C	7.7
329	Clc1cc2c(F)cccc2c(n1)C3CC[N+](CC3)CCc4ccccc4	7.7
330	Fc1cccc2c1CC[C@]23CC(=O)N3C4CC[N+](CC4)CCc5ccccc5	7.7
331	O=C1[C@@H]2CCCN2c3c(N1C4CC[N+](CC4)CCc5ccccc5)ccc[nH+]3	7.7
332	Clc1ccc2c(N(C3CC[N+](CC3)CCc4ccccc4)C(=O)[C@H](O2)C)c1	7.7
333	O=C1C[C@H](N1C2CC[N+](CC2)CCc3ccccc3)c4ccccn4	7.7
334	Fc1ccccc1[C@](OC)(C2CC[N+](CC2)CCc3ccccc3)C	7.7
335	O=C1CC2(N1C3CC[N+](CC3)CCc4ccccc4)CCCCCC2	7.7
336	O=C1C[C@](N1C2CC[N+](CC2)CCc3ccccc3)(CC(C)C)C	7.7
337	Sc1cc(F)nc(C2CC[N+](CC2)CCc3ccccc3)c1	7.7
338	O=C(N(C1CC[N+](CC1)CCc2ccccc2)[C@@H](CC)C#N)C	7.7
339	O=C1C[C@@]2(N1C3CC[N+](CC3)CCc4ccccc4)CCO[C@H]2C	7.7
340	ClC(Cl)C(=O)N(C1CC[N+](CC1)CCc2ccccc2)CCC	7.7
341	F[C@@H](C1CC[N+](CC1)CCc2ccccc2)c3ccccn3	7.7
342	O=C(N(C1CC[N+](CC1)CCc2ccccc2)CC)c3ccsc3	7.7
343	O=[S@](C1CC[N+](CC1)CCc2ccccc2)c3ccc(NC)cc3	7.7
344	O=C1C=Cc2cocc2N1C3CC[N+](CC3)CCc4ccccc4	7.7
345	O=S(=O)(c1nc2c(c(n1)C3CC[N+](CC3)CCc4ccccc4)ccs2)C	7.7
346	O=[S@](C1CC[N+](CC1)CCc2ccccc2)c3ccccc3OC	7.7
347	Fc1ccccc1S(=O)(=O)C2CC[N+](CC2)CCc3ccccc3	7.7
348	Fc1cc(cc(n1)C2CC[N+](CC2)CCc3ccccc3)C(F)(F)F	7.7
349	Clc1ccc(N(C2CC[N+](CC2)CCc3ccccc3)C(=O)C)cc1	7.6
350	Clc1cccc(N(C2CC[N+](CC2)CCc3ccccc3)C(=O)CC)c1	7.6
351	O=CCN(C1CC[N+](CC1)CCc2ccccc2)c3ccccc3	7.6
352	O[C@@](OC)(C1CC[N+](CC1)CCc2ccccc2)c3ccccc3	7.6
353	O=C1N(C2CC[N+](CC2)CCc3ccccc3)c4cscc4S1	7.6

354	O=C1N(C2CC[N+](CC2)CCc3ccccc3)c4c(S1)ccs4	7.6
355	O=C1C[C@@]2(N1C3CC[N+](CC3)CCc4ccccc4)CCOC2	7.6
356	O=CN(C1CC[N+](CC1)CCc2ccccc2)C3CCCCC3	7.6
357	O1C[C@@]1(C2CC[N+](CC2)CCc3ccccc3)c4ccccc4	7.6
358	O=S(=O)(C1CC[N+](CC1)CCc2ccccc2)c3ccccc3	7.6
359	S1CC(C2CC[N+](CC2)CCc3ccccc3)(C1)c4ccccc4	7.6
360	Fc1cnc2C=CC(=O)N(C3CC[N+](CC3)CCc4ccccc4)c2c1	7.6
361	O=[S@](C1CC[N+](CC1)CCc2ccccc2)c3cccs3	7.6
362	O=C1[C@@H]([C@H](N1C2CC[N+](CC2)CCc3ccccc3)c4cccs4)C	7.6
363	O=C1C=Nc2c(N1C3CC[N+](CC3)CCc4ccccc4)cns2	7.6
364	Clc1ccccc1[C@H](OC)C2CC[N+](CC2)CCc3ccccc3	7.6
365	O=C1N(C2CC[N+](CC2)CCc3ccccc3)c4cccnc4S1	7.6
366	O=C1C2(N(C3CC[N+](CC3)CCc4ccccc4)C(=O)N1)CCC(CC2)C	7.6
367	O=C(SC)N(C1CC[N+](CC1)CCc2ccccc2)C	7.6
368	O=C1CSc2c(N1C3CC[N+](CC3)CCc4ccccc4)cccn2	7.6
369	[N+]1(CCC(CC1)Cc2ccccc2)CCc3ccccc3	7.6
370	Br[C@@H](C1CC[N+](CC1)CCc2ccccc2)c3ccccc3	7.6
371	Fc1cccc([C@H](C2CC[N+](CC2)CCc3ccccc3)CCC)c1	7.6
372	Fc1ccccc1SC2CC[N+](CC2)CCc3ccccc3	7.6
373	O=C1C[C@@](N1C2CC[N+](CC2)CCc3ccccc3)(CCC)C	7.6
374	O=C1C[C@]2(N1C3CC[N+](CC3)CCc4ccccc4)CCO[C@@H](C2)C	7.6
375	Clc1nc2ccc(F)cc2c(n1)C3CC[N+](CC3)CCc4ccccc4	7.6
376	O=C(N(C1CC[N+](CC1)CCc2ccccc2)CC=C)C	7.6
377	O=C1C=Nn2cccc2N1C3CC[N+](CC3)CCc4ccccc4	7.6
378	Clc1ccccc1SC2CC[N+](CC2)CCc3ccccc3	7.6
379	Brc1cccc(N(C2CC[N+](CC2)CCc3ccccc3)CCC)c1	7.6
380	FC(F)(C1CC[N+](CC1)CCc2ccccc2)c3cccs3	7.6
381	O=C1CC2(N1C3CC[N+](CC3)CCc4ccccc4)CC(C2)C(C)C	7.6
382	O=C1C(=C([C@@H](OC)N1C2CC[N+](CC2)CCc3ccccc3)C)C	7.6
383	N#CC([C@@H](C1CC[N+](CC1)CCc2ccccc2)c3ccccc3)=C	7.6
384	Clc1cc2cc(F)ccc2c(n1)C3CC[N+](CC3)CCc4ccccc4	7.6
385	Fc1ccc(c(n1)C2CC[N+](CC2)CCc3ccccc3)CF	7.6
386	O=C1CC2(N1C3CC[N+](CC3)CCc4ccccc4)CC(OCC)C2	7.6
387	O=C1N=Nc2c(N1C3CC[N+](CC3)CCc4ccccc4)nncn2	7.6
388	N#C/C(C1CC[N+](CC1)CCc2ccccc2)=C/OCC	7.6
389	O=C(N(C1CC[N+](CC1)CCc2ccccc2)CC)[C@H]([N+])C	7.6
390	O=C1C2([C@H](N1C3CC[N+](CC3)CCc4ccccc4)c5ccccc5)CCCCC2	7.6
391	Cl[C@](C1CC[N+](CC1)CCc2ccccc2)(c3ccccc3)C#N	7.6
392	Fc1cc(F)ccc1[C@@H]2CC(=O)N2C3CC[N+](CC3)CCc4ccccc4	7.6
393	Fc1ccc(OC2CC[N+](CC2)CCc3ccccc3)c(CC(=O)C)c1	7.6
394	O=C1N(n2c(S1)ccn2)C3CC[N+](CC3)CCc4ccccc4	7.6
395	Sc1ccccc1SC2CC[N+](CC2)CCc3ccccc3	7.6

396	FC(F)c1cc(n(n1)C2CC[N+](CC2)CCc3ccccc3)C4CC4	7.6
397	Sc1ccc(F)c(n1)C2CC[N+](CC2)CCc3ccccc3	7.6
398	O=C1N(n2cccc2S1)C3CC[N+](CC3)CCc4ccccc4	7.6
399	O=C(N(C1CC[N+](CC1)CCc2ccccc2)C3CC3)NCC#C	7.6
400	O=CN(C1CC[N+](CC1)CCc2ccccc2)CCC(C)C	7.6
401	S=C(SC(C)C)C1CC[N+](CC1)CCc2ccccc2	7.6
402	FC(F)CN(C1CC[N+](CC1)CCc2ccccc2)c3ccccc3F	7.5
403	O=C(N(C1CC[N+](CC1)CCc2ccccc2)C3CCCC3)C[N+]	7.5
404	FC(F)(F)C(=O)N(C1CC[N+](CC1)CCc2ccccc2)CC#C	7.5
405	O=C(SC)N(C1CC[N+](CC1)CCc2ccccc2)CC	7.5
406	O=C(N(C1CC[N+](CC1)CCc2ccccc2)C3CC3)C[N+]	7.5
407	Cl[C@H](C1CC[N+](CC1)CCc2ccccc2)c3ccccc3	7.5
408	O[C@H](C1CC[N+](CC1)CCc2ccccc2)c3ccccc3	7.5
409	O=C(N(C1CC[N+](CC1)CCc2ccccc2)c3cccc(OC)c3)C	7.5
410	F[C@@H](C1CC[N+](CC1)CCc2ccccc2)c3ccccc3	7.5
411	O=[S@](C1CC[N+](CC1)CCc2ccccc2)c3ccccc3	7.5
412	O1CC[C@@H](N(C2CC[N+](CC2)CCc3ccccc3)c4ccccc4)C1	7.5
413	O=C(N(C1CC[N+](CC1)CCc2ccccc2)COC)NC	7.5
414	Fc1ccc2CCC(=O)N(C3CC[N+](CC3)CCc4ccccc4)c2c1	7.5
415	O=C1CC[C@@H](OC)N1C2CC[N+](CC2)CCc3ccccc3	7.5
416	O=C1C=Nc2cocc2N1C3CC[N+](CC3)CCc4ccccc4	7.5
417	O(C1CC[N+](CC1)CCc2ccccc2)c3ccccc3	7.5
418	O=C(N(C1CC[N+](CC1)CCc2ccccc2)C3CC3)[C@H](C)C#N	7.5
419	FC(F)c1cc(nn1C2CC[N+](CC2)CCc3ccccc3)C(F)F	7.5
420	O=CN(C1CC[N+](CC1)CCc2ccccc2)CCC	7.5
421	Clc1ccc2CC(=O)N(C3CC[N+](CC3)CCc4ccccc4)c2c1	7.5
422	Fc1ccc2c(N(C3CC[N+](CC3)CCc4ccccc4)C(=O)CO2)c1	7.5
423	ClC(Cl)(C1CC[N+](CC1)CCc2ccccc2)c3ccccc3	7.5
424	O=C(N(C1CC[N+](CC1)CCc2ccccc2)CC)CO	7.5
425	Clc1cccc([C@H](C2CC[N+](CC2)CCc3ccccc3)CCC)c1	7.5
426	FC(F)(C1CC[N+](CC1)CCc2ccccc2)c3ccccc3F	7.5
427	BrC(CN(C1CC[N+](CC1)CCc2ccccc2)C(=O)C)=C	7.5
428	[N+]CCc1nc(nn1C2CC[N+](CC2)CCc3ccccc3)C	7.5
429	FC(F)(C1CC[N+](CC1)CCc2ccccc2)c3cccc(c3)C	7.5
430	Fc1ccc-2c(N(C3CC[N+](CC3)CCc4ccccc4)C(=O)c5cccn52)c1	7.5
431	Fc1cc(SC(F)(F)F)cc(n1)C2CC[N+](CC2)CCc3ccccc3	7.5
432	SC[C@@H]1C(=O)N(C2CC[N+](CC2)CCc3ccccc3)CCC1	7.5
433	Fc1c(F)ccc(N(C2CC[N+](CC2)CCc3ccccc3)C(=O)C)c1	7.4
434	N#CCC[C@@H](C1CC[N+](CC1)CCc2ccccc2)c3ccccc3	7.4
435	O=S1(=O)CCC(C2CC[N+](CC2)CCc3ccccc3)(CC1)c4ccccc4	7.4
436	O=C1CC(C2CC[N+](CC2)CCc3ccccc3)(C1)c4ccccc4	7.4
437	O=C(N(C1CC[N+](CC1)CCc2ccccc2)CC)C[N+]	7.4

438	N#CC[C@@H](C1CC[N+](CC1)CCc2ccccc2)c3ccccc3	7.4
439	N#CN(C1CC[N+](CC1)CCc2ccccc2)c3ccccc3	7.4
440	Fc1ccc2c(N(C3CC[N+](CC3)CCc4ccccc4)C(=O)C=N2)c1	7.4
441	O=C1N(C2CC[N+](CC2)CCc3ccccc3)c4cocc4S1	7.4
442	O=C(N(C1CC[N+](CC1)CCc2ccccc2)CC)[C@H]3CCC[N+]3	7.4
443	Sc1cccc(SC2CC[N+](CC2)CCc3ccccc3)c1	7.4
444	O=C(N(C1CC[N+](CC1)CCc2ccccc2)C3CC3)NCC	7.4
445	O=C1C[C@H](N1C2CC[N+](CC2)CCc3ccccc3)c4ccc(o4)C	7.4
446	O=C1N=Nc2ccncc2N1C3CC[N+](CC3)CCc4ccccc4	7.4
447	O=[S@](C1CC[N+](CC1)CCc2ccccc2)c3cccc(c3)C	7.4
448	N#CC1(C2CC[N+](CC2)CCc3ccccc3)Cc4ccccc4C1	7.4
449	S[C@@H](C1CC[N+](CC1)CCc2ccccc2)c3ccccc3	7.4
450	O=C(OC(C)C)N(C1CC[N+](CC1)CCc2ccccc2)CC	7.4
451	O=C(SCC=C)C1CC[N+](CC1)CCc2ccccc2	7.4
452	O=C(N(C1CC[N+](CC1)CCc2ccccc2)C(C)C)C[N+]	7.4
453	O=C1Nc2ccccc2[C@@](O1)(OC)C3CC[N+](CC3)CCc4ccccc4	7.4
454	O=CN(C1CC[N+](CC1)CCc2ccccc2)C3CCC3	7.4
455	O=C(N(C1CC[N+](CC1)CCc2ccccc2)C)NC	7.4
456	Cl[C@H](C1CC[N+](CC1)CCc2ccccc2)c3ccccc3Cl	7.4
457	O=S(=O)(C1CC[N+](CC1)CCc2ccccc2)c3ccccc3O	7.4
458	Cc1ccccc1SC2CC[N+](CC2)CCc3ccccc3	7.4
459	O=C(N(C1CC[N+](CC1)CCc2ccccc2)c3ccncc3)C	7.3
460	Clc1ccc([C@H](OC)C2CC[N+](CC2)CCc3ccccc3)cc1	7.3
461	O=C(N(C1CC[N+](CC1)CCc2ccccc2)CC)[C@@H]3[C@@H]([N+])C3	7.3
462	Fc1cccc(SC2CC[N+](CC2)CCc3ccccc3)c1	7.3
463	COC1CC(C2CC[N+](CC2)CCc3ccccc3)(C1)c4ccccc4	7.3
464	O=C(N(C1CC[N+](CC1)CCc2ccccc2)[C@H](CC)C(=O)N)C	7.3
465	O=C1N(C2CC[N+](CC2)CCc3ccccc3)c4cncnc4S1	7.3
466	Clc1cccc(SC2CC[N+](CC2)CCc3ccccc3)c1	7.3
467	Cc1cccc(SC2CC[N+](CC2)CCc3ccccc3)c1	7.3
468	O=C1N(C2CC[N+](CC2)CCc3ccccc3)c4c(S1)ncnn4	7.3
469	O=C(N(C1CC[N+](CC1)CCc2ccccc2)C(C)C)CC[N+]	7.3
470	O=C(C1(C2CC[N+](CC2)CCc3ccccc3)Cc4ccccc4C1)C	7.3
471	FC(F)(C1CC[N+](CC1)CCc2ccccc2)c3ccc(cc3)C	7.3
472	O=C(N(C1CC[N+](CC1)CCc2ccccc2)C3CC3)[C@@H]([N+])C	7.3
473	Clc1cccc(C2(C3CC[N+](CC3)CCc4ccccc4)CC(=O)C2)c1	7.3
474	Cc1ccc([C@H](OC)C2CC[N+](CC2)CCc3ccccc3)cc1	7.2
475	Fc1ccc([C@H](OC)C2CC[N+](CC2)CCc3ccccc3)cc1	7.2
476	C#CCN(C1CC[N+](CC1)CCc2ccccc2)c3cc[nH+]cc3	7.2
477	O=C([C@H](C1CC[N+](CC1)CCc2ccccc2)c3ccccc3)C	7.2
478	O=[S@](C1CC[N+](CC1)CCc2ccccc2)c3ccsc3	7.2
479	O=C(N(C1CC[N+](CC1)CCc2ccccc2)CC)CC#N	7.2

480	O=C1N=Nc2c(N1C3CC[N+](CC3)CCc4ccccc4)cncn2	7.2
481	O=[S@](C1CC[N+](CC1)CCc2ccccc2)c3ccccc3C	7.2
482	O=C1N(C2CC[N+](CC2)CCc3ccccc3)c4cnsc4S1	7.2
483	O=C1C[C@H](N1C2CC[N+](CC2)CCc3ccccc3)c4ccc(s4)C	7.2
484	Cl[C@H](C1CC[N+](CC1)CCc2ccccc2)c3cc(Cl)ccc3	7.2
485	S=C(C1CC[N+](CC1)CCc2ccccc2)c3ccsc3	7.2
486	O=S(=O)(C1CC[N+](CC1)CCc2ccccc2)c3ccc(cc3)C	7.2
487	O=C(N(C1CC[N+](CC1)CCc2ccccc2)CCC)C[N+]	7.1
488	S=C(C1CC[N+](CC1)CCc2ccccc2)CCC	7.1
489	O=C1N(C2CC[N+](CC2)CCc3ccccc3)c4cnccc4S1	7.1
490	N#CC[C@@H](C1CC[N+](CC1)CCc2ccccc2)c3cccs3	7.1
491	Cc1ccc(SC2CC[N+](CC2)CCc3ccccc3)cc1	7.1
492	O=S(=O)(C1CC[N+](CC1)CCc2ccccc2)c3ccsc3	7.1
493	Cc1c(SC2CC[N+](CC2)CCc3ccccc3)ccs1	7.1
494	S(C1CC[N+](CC1)CCc2ccccc2)c3ccsc3	7.0
495	O=S1(=O)CC[C@@]2(C1)CC(=O)N2C3CC[N+](CC3)CCc4ccccc4	7.0
496	O=C(N(C1CC[N+](CC1)CCc2ccccc2)CC)[C@@H](C[N+])C	7.0
497	Clc1ccc([C@H](F)C2CC[N+](CC2)CCc3ccccc3)cc1	7.0
498	O=C(N(C1CC[N+](CC1)CCc2ccccc2)C)CC[N+]	7.0
499	Fc1ccc(OC2CC[N+](CC2)CCc3ccccc3)cc1	6.9
500	Clc1ccc(SC2CC[N+](CC2)CCc3ccccc3)cc1	6.9

Table S6

List, SMILE and predicted pK_i values for Series 4.

N°	SMILES	Pred pK_i
1	O=C(O[C@H]1CCCO1)C2(N(c3ccccc3)C(=O)CC)CC[N+](CC2)CCc4ccccc4	10.8
2	O=C(OCOC)C1(N(c2ccccc2)C(=O)CC)CC[N+](CC1)CCc3ccccc3	10.6
3	O=C(N(C1(CC[N+](CC1)CCc2ccccc2)C3=NOCCO3)c4ccccc4)CC	10.6
4	O=C(NS(=O)(=O)N)C1(N(c2ccccc2)C(=O)CC)CC[N+](CC1)CCc3ccccc3	10.6
5	O=C(C1(N(c2ccccc2)C(=O)CC)CC[N+](CC1)CCc3ccccc3)[C@H]4CCO4	10.5
6	FC(F)c1nnc(o1)C2(N(c3ccccc3)C(=O)CC)CC[N+](CC2)CCc4ccccc4	10.5
7	O=C(N(C1(n2c3c(nn2)c[nH]n3)CC[N+](CC1)CCc4ccccc4)c5ccccc5)CC	10.5
8	O=C(N(C1(CC[N+](CC1)CCc2ccccc2)C3=NCCCO3)c4ccccc4)CC	10.4
9	O=C(N(C1(CC[N+](CC1)CCc2ccccc2)c3nnc(s3)O)c4ccccc4)CC	10.4
10	O=[S@@](N(C)C)C1(N(c2ccccc2)C(=O)CC)CC[N+](CC1)CCc3ccccc3	10.4
11	O=C(OCC[N+]([O-])=O)C1(N(c2ccccc2)C(=O)CC)CC[N+](CC1)CCc3ccccc3	10.4
12	O=C(C1(N(c2ccccc2)C(=O)CC)CC[N+](CC1)CCc3ccccc3)[C@H]4CCC=N4	10.4
13	FCCNS(=O)(=O)C1(N(c2ccccc2)C(=O)CC)CC[N+](CC1)CCc3ccccc3	10.4
14	O=C(C1(N(c2ccccc2)C(=O)CC)CC[N+](CC1)CCc3ccccc3)/C(C)=C/O	10.4
15	O=C(N(C1(CC[N+](CC1)CCc2ccccc2)c3nnn(n3)CC)c4ccccc4)CC	10.4
16	O=C(OCCCO)C1(N(c2ccccc2)C(=O)CC)CC[N+](CC1)CCc3ccccc3	10.4

17	O=C(NS(=O)(=O)C)C1(N(c2ccccc2)C(=O)CC)CC[N+](CC1)CCc3ccccc3	10.4
18	O=C(N(C1(CC[N+](CC1)CCc2ccccc2)c3noc(n3)CO)c4ccccc4)CC	10.4
19	O=C(C1(N(c2ccccc2)C(=O)CC)CC[N+](CC1)CCc3ccccc3)[C@H]4CCCO4	10.4
20	O=C(O[C@@H](C)C#N)C1(N(c2ccccc2)C(=O)CC)CC[N+](CC1)CCc3ccccc3	10.4
21	O=C(C1(N(c2ccccc2)C(=O)CC)CC[N+](CC1)CCc3ccccc3)[C@@H]4C=CCC4	10.4
22	O=C(N(C1(CC[N+](CC1)CCc2ccccc2)c3nnc4n3nns4)c5ccccc5)CC	10.4
23	O=C(N[C@@H](C)C=O)C1(N(c2ccccc2)C(=O)CC)CC[N+](CC1)CCc3ccccc3	10.4
24	O=C(N(C1(CC[N+](CC1)CCc2ccccc2)c3nn(nn3)CC#C)c4ccccc4)CC	10.4
25	O=C(OC(CC)CC)C1(N(c2ccccc2)C(=O)CC)CC[N+](CC1)CCc3ccccc3	10.4
26	O=C(N(O)C)C1(N(c2ccccc2)C(=O)CC)CC[N+](CC1)CCc3ccccc3	10.3
27	O=C(N(C1(CC[N+](CC1)CCc2ccccc2)c3nc(on3)C)c4ccccc4)CC	10.3
28	O=C(N(C1(CC[N+](CC1)CCc2ccccc2)c3nc(no3)N)c4ccccc4)CC	10.3
29	O=C(OC1(CC1)C)C2(N(c3ccccc3)C(=O)CC)CC[N+](CC2)CCc4ccccc4	10.3
30	O=C(N(C1(n2nnc(n2)N)CC[N+](CC1)CCc3ccccc3)c4ccccc4)CC	10.3
31	O=C(N(C1(CC[N+](CC1)CCc2ccccc2)c3noc(O)n3)c4ccccc4)CC	10.3
32	O=C(OCC(=O)N)C1(N(c2ccccc2)C(=O)CC)CC[N+](CC1)CCc3ccccc3	10.3
33	O=C(N(C1(CC[N+](CC1)CCc2ccccc2)c3nn(nn3)C)c4ccccc4)CC	10.3
34	O=C(N(C1(CC[N+](CC1)CCc2ccccc2)c3nnns3)c4ccccc4)CC	10.3
35	O=C(N(C1(CC[N+](CC1)CCc2ccccc2)c3noc(n3)C=C)c4ccccc4)CC	10.3
36	S[C@@H](C(=O)C1(N(c2ccccc2)C(=O)CC)CC[N+](CC1)CCc3ccccc3)C	10.3
37	O=C(N(C1(CC[N+](CC1)CCc2ccccc2)c3noc(n3)NC)c4ccccc4)CC	10.3
38	O=C(N1CCC=N1)C2(N(c3ccccc3)C(=O)CC)CC[N+](CC2)CCc4ccccc4	10.3
39	O=C(N(C1(CC[N+](CC1)CCc2ccccc2)c3nnc(o3)OCC)c4ccccc4)CC	10.3
40	O=C(OCCO[N+]([O-])=O)C1(N(c2ccccc2)C(=O)CC)CC[N+](CC1)CCc3ccccc3	10.3
41	O=C(N(C1(CC[N+](CC1)CCc2ccccc2)c3nnc(cn3)C)c4ccccc4)CC	10.3
42	O=C(NCCO)C1(N(c2ccccc2)C(=O)CC)CC[N+](CC1)CCc3ccccc3	10.3
43	O=C(N(C1(CC[N+](CC1)CCc2ccccc2)c3nnc4[nH]nnn43)c5ccccc5)CC	10.3
44	O=C(N1CCCO1)C2(N(c3ccccc3)C(=O)CC)CC[N+](CC2)CCc4ccccc4	10.3
45	O=C(N(C1(CC[N+](CC1)CCc2ccccc2)c3noc(n3)C(C)C)c4ccccc4)CC	10.3
46	O=C(C1(N(c2ccccc2)C(=O)CC)CC[N+](CC1)CCc3ccccc3)c4c(ocn4)C	10.3
47	O=C(OC(C)(C)C#C)C1(N(c2ccccc2)C(=O)CC)CC[N+](CC1)CCc3ccccc3	10.3
48	O=C(N(CC#C)C)C1(N(c2ccccc2)C(=O)CC)CC[N+](CC1)CCc3ccccc3	10.3
49	O=C(C1(N(c2ccccc2)C(=O)CC)CC[N+](CC1)CCc3ccccc3)c4ccsn4	10.3
50	O=C(N(C1(CC[N+](CC1)CCc2ccccc2)c3nnc(o3)/C=C/C)c4ccccc4)CC	10.3
51	O=C(N(C1(CC[N+](CC1)CCc2ccccc2)c3nsnn3)c4ccccc4)CC	10.2
52	O=C(N(C1(CC[N+](CC1)CCc2ccccc2)c3conn3)c4ccccc4)CC	10.2
53	FC(F)(C(=O)C1(N(c2ccccc2)C(=O)CC)CC[N+](CC1)CCc3ccccc3)C	10.2
54	O=C(OC/C=C/C)C1(N(c2ccccc2)C(=O)CC)CC[N+](CC1)CCc3ccccc3	10.2
55	O=C(C1(N(c2ccccc2)C(=O)CC)CC[N+](CC1)CCc3ccccc3)/C=C=C	10.2
56	O=C(OC1COC1)C2(N(c3ccccc3)C(=O)CC)CC[N+](CC2)CCc4ccccc4	10.2
57	S=C1N=NC(=N1)C2(N(c3ccccc3)C(=O)CC)CC[N+](CC2)CCc4ccccc4	10.2
58	O=C(C1(N(c2ccccc2)C(=O)CC)CC[N+](CC1)CCc3ccccc3)[C@@H]4C[C@H]4C	10.2

59	O=C(N(C1(CC[N+](CC1)CCc2ccccc2)c3noc(n3)CC)c4ccccc4)CC	10.2
60	O=C(NCOC)C1(N(c2ccccc2)C(=O)CC)CC[N+](CC1)CCc3ccccc3	10.2
61	O=C(C1(N(c2ccccc2)C(=O)CC)CC[N+](CC1)CCc3ccccc3)c4cocn4	10.2
62	O=C(NC(C)=C)C1(N(c2ccccc2)C(=O)CC)CC[N+](CC1)CCc3ccccc3	10.2
63	FC(F)(C(=O)C1(N(c2ccccc2)C(=O)CC)CC[N+](CC1)CCc3ccccc3)CC	10.2
64	O=C(O[C@H](C1CC1)C)C2(N(c3ccccc3)C(=O)CC)CC[N+](CC2)CCc4ccccc4	10.2
65	O=C(N(C1(CC[N+](CC1)CCc2ccccc2)c3noc(n3)C4CC4)c5ccccc5)CC	10.2
66	O=C(N(C1(CC[N+](CC1)CCc2ccccc2)c3nncc(n3)C)c4ccccc4)CC	10.2
67	FCCCNC(=O)C1(N(c2ccccc2)C(=O)CC)CC[N+](CC1)CCc3ccccc3	10.2
68	O=C(O[C@H](CC)C=C)C1(N(c2ccccc2)C(=O)CC)CC[N+](CC1)CCc3ccccc3	10.2
69	O=C(OC1(CCC1)C)C2(N(c3ccccc3)C(=O)CC)CC[N+](CC2)CCc4ccccc4	10.2
70	O=C(N(C1(n2c3c(nn2)cco3)CC[N+](CC1)CCc4ccccc4)c5ccccc5)CC	10.2
71	O=C(N(C1(n2c3c(scn3)nn2)CC[N+](CC1)CCc4ccccc4)c5ccccc5)CC	10.2
72	O=C(N(C1(CC[N+](CC1)CCc2ccccc2)c3c4c(no3)cco4)c5ccccc5)CC	10.2
73	O=C(N(C1(CC[N+](CC1)CCc2ccccc2)c3c4c(on3)cco4)c5ccccc5)CC	10.2
74	O=C(N(C1(CC[N+](CC1)CCc2ccccc2)c3nnc(o3)NC)c4ccccc4)CC	10.2
75	O=C(OCCCC#N)C1(N(c2ccccc2)C(=O)CC)CC[N+](CC1)CCc3ccccc3	10.2
76	S[C@@H](C(=O)C1(N(c2ccccc2)C(=O)CC)CC[N+](CC1)CCc3ccccc3)CC	10.2
77	O=C(OCCC#N)C1(N(c2ccccc2)C(=O)CC)CC[N+](CC1)CCc3ccccc3	10.2
78	O=C(N(C1(CC[N+](CC1)CCc2ccccc2)C3=COCCO3)c4ccccc4)CC	10.2
79	O=C(N(C1(CC[N+](CC1)CCc2ccccc2)c3nnnc4ccnn43)c5ccccc5)CC	10.2
80	O=C(N(C1(CC[N+](CC1)CCc2ccccc2)c3nnc4ccnnn43)c5ccccc5)CC	10.2
81	O=C(OCCCC=C)C1(N(c2ccccc2)C(=O)CC)CC[N+](CC1)CCc3ccccc3	10.2
82	O=C(OC[S@@](=O)C)C1(N(c2ccccc2)C(=O)CC)CC[N+](CC1)CCc3ccccc3	10.2
83	O=C(C1(N(c2ccccc2)C(=O)CC)CC[N+](CC1)CCc3ccccc3)C(=O)C(OC)=O	10.2
84	O=C(OCCSC)C1(N(c2ccccc2)C(=O)CC)CC[N+](CC1)CCc3ccccc3	10.2
85	O=C(OCC=C)C1(N(c2ccccc2)C(=O)CC)CC[N+](CC1)CCc3ccccc3	10.1
86	O=C(OC1CC1)C2(N(c3ccccc3)C(=O)CC)CC[N+](CC2)CCc4ccccc4	10.1
87	O=C(OCCC=C)C1(N(c2ccccc2)C(=O)CC)CC[N+](CC1)CCc3ccccc3	10.1
88	O=C(OCCC#C)C1(N(c2ccccc2)C(=O)CC)CC[N+](CC1)CCc3ccccc3	10.1
89	O=C(N(C1(CC[N+](CC1)CCc2ccccc2)c3c(O)con3)c4ccccc4)CC	10.1
90	Sc1nnc(o1)C2(N(c3ccccc3)C(=O)CC)CC[N+](CC2)CCc4ccccc4	10.1
91	O=C(OC1CCC1)C2(N(c3ccccc3)C(=O)CC)CC[N+](CC2)CCc4ccccc4	10.1
92	O=C(C1(N(c2ccccc2)C(=O)CC)CC[N+](CC1)CCc3ccccc3)C(=O)C(=O)C	10.1
93	O=C(N(C1(CC[N+](CC1)CCc2ccccc2)c3noc(OC)n3)c4ccccc4)CC	10.1
94	O=C(N(C1(n2nnc(n2)CC)CC[N+](CC1)CCc3ccccc3)c4ccccc4)CC	10.1
95	O=C(N(C1(CC[N+](CC1)CCc2ccccc2)c3nnc(o3)OC)c4ccccc4)CC	10.1
96	O=C(N(C1(CC[N+](CC1)CCc2ccccc2)c3nnccn3)c4ccccc4)CC	10.1
97	O=C(O[C@H](C)C=C)C1(N(c2ccccc2)C(=O)CC)CC[N+](CC1)CCc3ccccc3	10.1
98	O=C(O[C@H](CC)C)C1(N(c2ccccc2)C(=O)CC)CC[N+](CC1)CCc3ccccc3	10.1
99	O[C@H](OC)C1(N(c2ccccc2)C(=O)CC)CC[N+](CC1)CCc3ccccc3	10.1
100	O=C(C1(N(c2ccccc2)C(=O)CC)CC[N+](CC1)CCc3ccccc3)C4CCC4	10.1

101	O=C(C1(N(c2ccccc2)C(=O)CC)CC[N+](CC1)CCc3ccccc3)C(C)=C	10.1
102	O=C(N(C1(n2c3c(ocn3)nn2)CC[N+](CC1)CCc4ccccc4)c5ccccc5)CC	10.1
103	O=C(C1(N(c2ccccc2)C(=O)CC)CC[N+](CC1)CCc3ccccc3)[C@H](O)C#C	10.1
104	Sc1cn(nn1)C2(N(c3ccccc3)C(=O)CC)CC[N+](CC2)CCc4ccccc4	10.1
105	O=C(N(C1(CC[N+](CC1)CCc2ccccc2)c3nc(no3)CC)c4ccccc4)CC	10.1
106	O=C(N(C1(CC[N+](CC1)CCc2ccccc2)c3nnc(o3)CC)c4ccccc4)CC	10.1
107	O=C(N(C1(CC[N+](CC1)CCc2ccccc2)c3nnc4cnon43)c5ccccc5)CC	10.1
108	O=C(N(C1(CC[N+](CC1)CCc2ccccc2)/C=C(\O)C(=O)N)c3ccccc3)CC	10.1
109	O=C(SCC=C)C1(N(c2ccccc2)C(=O)CC)CC[N+](CC1)CCc3ccccc3	10.1
110	O=C(N(C1(CC[N+](CC1)CCc2ccccc2)c3nnc(SC)o3)c4ccccc4)CC	10.1
111	O=C(OC1CCCC1)C2(N(c3ccccc3)C(=O)CC)CC[N+](CC2)CCc4ccccc4	10.1
112	O=C(N(C1(CC[N+](CC1)CCc2ccccc2)c3ncc(o3)C)c4ccccc4)CC	10.1
113	O=C(SCCC)C1(N(c2ccccc2)C(=O)CC)CC[N+](CC1)CCc3ccccc3	10.1
114	O=C([C@H]1CC21CC2)C3(N(c4ccccc4)C(=O)CC)CC[N+](CC3)CCc5ccccc5	10.1
115	Clc1cnc(nn1)C2(N(c3ccccc3)C(=O)CC)CC[N+](CC2)CCc4ccccc4	10.1
116	O=C(C1(N(c2ccccc2)C(=O)CC)CC[N+](CC1)CCc3ccccc3)[C@@H](CC)C	10.1
117	O=S(=O)(NCC)C1(N(c2ccccc2)C(=O)CC)CC[N+](CC1)CCc3ccccc3	10.1
118	O=C(Oc1cccs1)C2(N(c3ccccc3)C(=O)CC)CC[N+](CC2)CCc4ccccc4	10.1
119	FC(C(=O)C1(N(c2ccccc2)C(=O)CC)CC[N+](CC1)CCc3ccccc3)=C(C)C	10.1
120	O=C(O[C@H](C1CC1)C#C)C2(N(c3ccccc3)C(=O)CC)CC[N+](CC2)CCc4ccccc4	10.1
121	O=C(OC(C)(C)C)C1(N(c2ccccc2)C(=O)CC)CC[N+](CC1)CCc3ccccc3	10.1
122	FC(F)(F)CNC(=O)C1(N(c2ccccc2)C(=O)CC)CC[N+](CC1)CCc3ccccc3	10.1
123	O=C(OC1C[C@@H]2C[C@@H]2C1)C3(N(c4ccccc4)C(=O)CC)CC[N+](CC3)CCc5ccccc5	10.1
124	O=C(C1(N(c2ccccc2)C(=O)CC)CC[N+](CC1)CCc3ccccc3)c4coc(n4)N	10.1
125	O=C(NN(C)C)C1(N(c2ccccc2)C(=O)CC)CC[N+](CC1)CCc3ccccc3	10.1
126	O=C(N(C1(/[N+]([O-])=N/OCC)CC[N+](CC1)CCc2ccccc2)c3ccccc3)CC	10.1
127	O=C(N[C@@H](C)C=C)C1(N(c2ccccc2)C(=O)CC)CC[N+](CC1)CCc3ccccc3	10.1
128	SCc1cc(on1)C2(N(c3ccccc3)C(=O)CC)CC[N+](CC2)CCc4ccccc4	10.1
129	O=C(N(C1(CC[N+](CC1)CCc2ccccc2)c3nc(N(C)C)no3)c4ccccc4)CC	10.1
130	O=C(C1(N(c2ccccc2)C(=O)CC)CC[N+](CC1)CCc3ccccc3)c4ccon4	10.1
131	O=C(N(C1(CC[N+](CC1)CCc2ccccc2)c3nocn3)c4ccccc4)CC	10.0
132	O=C(OC(C)C)C1(N(c2ccccc2)C(=O)CC)CC[N+](CC1)CCc3ccccc3	10.0
133	O=C(N(C1(CC[N+](CC1)CCc2ccccc2)c3nnc(o3)O)c4ccccc4)CC	10.0
134	O=C(O[C@@H](C)C#C)C1(N(c2ccccc2)C(=O)CC)CC[N+](CC1)CCc3ccccc3	10.0
135	O=C(ON(C)C)C1(N(c2ccccc2)C(=O)CC)CC[N+](CC1)CCc3ccccc3	10.0
136	O=C(N(N)C)C1(N(c2ccccc2)C(=O)CC)CC[N+](CC1)CCc3ccccc3	10.0
137	Cl[C@H](OC(=O)C1(N(c2ccccc2)C(=O)CC)CC[N+](CC1)CCc3ccccc3)C	10.0
138	O=C(N(C1(CC[N+](CC1)CCc2ccccc2)C(OCC)=N)c3ccccc3)CC	10.0
139	O=C(N(C1(CC[N+](CC1)CCc2ccccc2)c3nc(no3)C)c4ccccc4)CC	10.0
140	FC1(C(=O)C2(N(c3ccccc3)C(=O)CC)CC[N+](CC2)CCc4ccccc4)CC1	10.0
141	O=C(OCC#CC)C1(N(c2ccccc2)C(=O)CC)CC[N+](CC1)CCc3ccccc3	10.0
142	O=C(NC1CC1)C2(N(c3ccccc3)C(=O)CC)CC[N+](CC2)CCc4ccccc4	10.0

143	O=C(C1(N(c2ccccc2)C(=O)CC)CC[N+](CC1)CCc3ccccc3)C(C)C	10.0
144	O=C(OCC(C)C)C1(N(c2ccccc2)C(=O)CC)CC[N+](CC1)CCc3ccccc3	10.0
145	O=C(SCC#C)C1(N(c2ccccc2)C(=O)CC)CC[N+](CC1)CCc3ccccc3	10.0
146	FC(F)(F)C(=O)C1(N(c2ccccc2)C(=O)CC)CC[N+](CC1)CCc3ccccc3	10.0
147	Cl/C=C\COC(=O)C1(N(c2ccccc2)C(=O)CC)CC[N+](CC1)CCc3ccccc3	10.0
148	O=C(N(C1(CC[N+](CC1)CCc2ccccc2)c3noc(n3)N)c4ccccc4)CC	10.0
149	O=C(C1(N(c2ccccc2)C(=O)CC)CC[N+](CC1)CCc3ccccc3)c4nccs4	10.0
150	O=C(N(C1(CC[N+](CC1)CCc2ccccc2)C(OC(C)C)=N)c3ccccc3)CC	10.0
151	O=C(N(OC)C)C1(N(c2ccccc2)C(=O)CC)CC[N+](CC1)CCc3ccccc3	10.0
152	O=C(C1(N(c2ccccc2)C(=O)CC)CC[N+](CC1)CCc3ccccc3)C(OC)=C	10.0
153	O=C(N(C)C)C1(N(c2ccccc2)C(=O)CC)CC[N+](CC1)CCc3ccccc3	10.0
154	O=C(N(O)CC)C1(N(c2ccccc2)C(=O)CC)CC[N+](CC1)CCc3ccccc3	10.0
155	O=C(NC(C)C)C1(N(c2ccccc2)C(=O)CC)CC[N+](CC1)CCc3ccccc3	10.0
156	O=C(N1C=CCC1)C2(N(c3ccccc3)C(=O)CC)CC[N+](CC2)CCc4ccccc4	10.0
157	ClC(F)(F)C(=O)C1(N(c2ccccc2)C(=O)CC)CC[N+](CC1)CCc3ccccc3	10.0
158	O=C(C1(N(c2ccccc2)C(=O)CC)CC[N+](CC1)CCc3ccccc3)c4cnc(o4)O	10.0
159	O=C(C1(N(c2ccccc2)C(=O)CC)CC[N+](CC1)CCc3ccccc3)c4cc[nH]n4	10.0
160	O=C(C1(N(c2ccccc2)C(=O)CC)CC[N+](CC1)CCc3ccccc3)CCC=C	10.0
161	O=C(C(=O)C1(N(c2ccccc2)C(=O)CC)CC[N+](CC1)CCc3ccccc3)C(C)C	10.0
162	O=C(C1(N(c2ccccc2)C(=O)CC)CC[N+](CC1)CCc3ccccc3)C4(CC4)C	10.0
163	O=C(C1(N(c2ccccc2)C(=O)CC)CC[N+](CC1)CCc3ccccc3)CCCC#C	10.0
164	O=C(C1(N(c2ccccc2)C(=O)CC)CC[N+](CC1)CCc3ccccc3)[C@H](O[N+]([O-])=O)C	10.0
165	O=C(N(C1(CC[N+](CC1)CCc2ccccc2)c3nc(no3)CO)c4ccccc4)CC	10.0
166	O=C(N(C1(n2nnc(n2)C3CC3)CC[N+](CC1)CCc4ccccc4)c5ccccc5)CC	10.0
167	FC(F)(F)C1(N=N1)C2(N(c3ccccc3)C(=O)CC)CC[N+](CC2)CCc4ccccc4	10.0
168	O=C(C1(N(c2ccccc2)C(=O)CC)CC[N+](CC1)CCc3ccccc3)/C(=C/C)C	10.0
169	O=C(NCO)C1(N(c2ccccc2)C(=O)CC)CC[N+](CC1)CCc3ccccc3	10.0
170	O=C(O[C@H](C(C)C)C)C1(N(c2ccccc2)C(=O)CC)CC[N+](CC1)CCc3ccccc3	10.0
171	O=C(C1(N(c2ccccc2)C(=O)CC)CC[N+](CC1)CCc3ccccc3)c4coc(n4)C	10.0
172	O=C(C1(N(c2ccccc2)C(=O)CC)CC[N+](CC1)CCc3ccccc3)c4ncc(s4)C	10.0
173	SCCCOC(=O)C1(N(c2ccccc2)C(=O)CC)CC[N+](CC1)CCc3ccccc3	10.0
174	O=C(N(C1(n2nnc(n2)CCC)CC[N+](CC1)CCc3ccccc3)c4ccccc4)CC	10.0
175	O=[S@](C1(N(c2ccccc2)C(=O)CC)CC[N+](CC1)CCc3ccccc3)CC	10.0
176	O=C(C1(N(c2ccccc2)C(=O)CC)CC[N+](CC1)CCc3ccccc3)CCC4CC4	10.0
177	O=C(O[C@H](CCC)C)C1(N(c2ccccc2)C(=O)CC)CC[N+](CC1)CCc3ccccc3	10.0
178	O=C(N(C1(CC[N+](CC1)CCc2ccccc2)c3noc(n3)CCC)c4ccccc4)CC	10.0
179	F[C@H](CNC(=O)C1(N(c2ccccc2)C(=O)CC)CC[N+](CC1)CCc3ccccc3)C	10.0
180	SC[C@H](C(=O)C1(N(c2ccccc2)C(=O)CC)CC[N+](CC1)CCc3ccccc3)C	10.0
181	O=C(N(C1(CC[N+](CC1)CCc2ccccc2)c3nc(no3)C(C)C)c4ccccc4)CC	10.0
182	O=C(N(C1(CC[N+](CC1)CCc2ccccc2)C(OCCCC)=N)c3ccccc3)CC	10.0
183	O=C(N(C1(CC[N+](CC1)CCc2ccccc2)c3noc4ccnn43)c5ccccc5)CC	10.0
184	O=C(N(C1(CC[N+](CC1)CCc2ccccc2)c3nc(SC)no3)c4ccccc4)CC	10.0

185	O=C(NCCC=C)C1(N(c2ccccc2)C(=O)CC)CC[N+](CC1)CCc3ccccc3	10.0
186	O=C(C1(N(c2ccccc2)C(=O)CC)CC[N+](CC1)CCc3ccccc3)c4ccccn4	10.0
187	O=C(C1(N(c2ccccc2)C(=O)CC)CC[N+](CC1)CCc3ccccc3)c4ccno4	10.0
188	O=C(OC)C1(N(c2ccccc2)C(=O)CC)CC[N+](CC1)CCc3ccccc3	9.9
189	O=C(OCC)C1(N(c2ccccc2)C(=O)CC)CC[N+](CC1)CCc3ccccc3	9.9
190	O=C(N(C1(CC[N+](CC1)CCc2ccccc2)C(OC)=N)c3ccccc3)CC	9.9
191	O=C(OCC#C)C1(N(c2ccccc2)C(=O)CC)CC[N+](CC1)CCc3ccccc3	9.9
192	O=C(C1(N(c2ccccc2)C(=O)CC)CC[N+](CC1)CCc3ccccc3)CC	9.9
193	O=C(OCCC)C1(N(c2ccccc2)C(=O)CC)CC[N+](CC1)CCc3ccccc3	9.9
194	O=C(SC)C1(N(c2ccccc2)C(=O)CC)CC[N+](CC1)CCc3ccccc3	9.9
195	O=C(O/C=C/C)C1(N(c2ccccc2)C(=O)CC)CC[N+](CC1)CCc3ccccc3	9.9
196	O=C(OC(C)=C)C1(N(c2ccccc2)C(=O)CC)CC[N+](CC1)CCc3ccccc3	9.9
197	Sc1nc(no1)C2(N(c3ccccc3)C(=O)CC)CC[N+](CC2)CCc4ccccc4	9.9
198	O=C(C1(N(c2ccccc2)C(=O)CC)CC[N+](CC1)CCc3ccccc3)C(=O)C	9.9
199	O=C(N(C1(n2nc(nn2)C)CC[N+](CC1)CCc3ccccc3)c4ccccc4)CC	9.9
200	O=C(C1(N(c2ccccc2)C(=O)CC)CC[N+](CC1)CCc3ccccc3)C4CC4	9.9
201	O=C(OCC1CC1)C2(N(c3ccccc3)C(=O)CC)CC[N+](CC2)CCc4ccccc4	9.9
202	O=C(N(C1(n2ccnn2)CC[N+](CC1)CCc3ccccc3)c4ccccc4)CC	9.9
203	SCCC(=O)C1(N(c2ccccc2)C(=O)CC)CC[N+](CC1)CCc3ccccc3	9.9
204	O=C(N(C1(CC[N+](CC1)CCc2ccccc2)C3=NCCO3)c4ccccc4)CC	9.9
205	FC[C@H](OC(=O)C1(N(c2ccccc2)C(=O)CC)CC[N+](CC1)CCc3ccccc3)C	9.9
206	O=C(C1(N(c2ccccc2)C(=O)CC)CC[N+](CC1)CCc3ccccc3)/C(O)=C/C	9.9
207	Clc1nnn(n1)C2(N(c3ccccc3)C(=O)CC)CC[N+](CC2)CCc4ccccc4	9.9
208	O=C(Oc1ccco1)C2(N(c3ccccc3)C(=O)CC)CC[N+](CC2)CCc4ccccc4	9.9
209	O=C(N(C1(CC[N+](CC1)CCc2ccccc2)c3c(O)non3)c4ccccc4)CC	9.9
210	Fc1cn(nn1)C2(N(c3ccccc3)C(=O)CC)CC[N+](CC2)CCc4ccccc4	9.9
211	O=C(SCC)C1(N(c2ccccc2)C(=O)CC)CC[N+](CC1)CCc3ccccc3	9.9
212	S=C(OCC)C1(N(c2ccccc2)C(=O)CC)CC[N+](CC1)CCc3ccccc3	9.9
213	O=C(N(C1(n2cc(nn2)C)CC[N+](CC1)CCc3ccccc3)c4ccccc4)CC	9.9
214	Cl[C@H](C(=O)C1(N(c2ccccc2)C(=O)CC)CC[N+](CC1)CCc3ccccc3)C	9.9
215	O=C(C1(N(c2ccccc2)C(=O)CC)CC[N+](CC1)CCc3ccccc3)CC4CC4	9.9
216	O=C(N(C1(CC[N+](CC1)CCc2ccccc2)C3=NC[C@@H](O3)C)c4ccccc4)CC	9.9
217	O=[S@](C1(N(c2ccccc2)C(=O)CC)CC[N+](CC1)CCc3ccccc3)C	9.9
218	O=C(NCC=C)C1(N(c2ccccc2)C(=O)CC)CC[N+](CC1)CCc3ccccc3	9.9
219	O=C(C1(N(c2ccccc2)C(=O)CC)CC[N+](CC1)CCc3ccccc3)CCCC	9.9
220	O=C(O[C@H](CC#C)C)C1(N(c2ccccc2)C(=O)CC)CC[N+](CC1)CCc3ccccc3	9.9
221	FC(F)(F)NC(=O)C1(N(c2ccccc2)C(=O)CC)CC[N+](CC1)CCc3ccccc3	9.9
222	O=C(OCC1CCC1)C2(N(c3ccccc3)C(=O)CC)CC[N+](CC2)CCc4ccccc4	9.9
223	O=C(C1(N(c2ccccc2)C(=O)CC)CC[N+](CC1)CCc3ccccc3)C=C(C)C	9.9
224	O=C(C1(N(c2ccccc2)C(=O)CC)CC[N+](CC1)CCc3ccccc3)C=C4CC4	9.9
225	O=C(N(C1(CC[N+](CC1)CCc2ccccc2)C(OC)=C)c3ccccc3)CC	9.9
226	O=C(N(C1(CC[N+](CC1)CCc2ccccc2)c3nnc(o3)N)c4ccccc4)CC	9.9

227	Fc1nc(C(=O)C2(N(c3ccccc3)C(=O)CC)CC[N+](CC2)CCc4ccccc4)co1	9.9
228	O=C(N(N)N)C1(N(c2ccccc2)C(=O)CC)CC[N+](CC1)CCc3ccccc3	9.9
229	O=C(OCCC1CC1)C2(N(c3ccccc3)C(=O)CC)CC[N+](CC2)CCc4ccccc4	9.9
230	O=C(C1(N(c2ccccc2)C(=O)CC)CC[N+](CC1)CCc3ccccc3)C4=NCCS4	9.9
231	O=C(N(C1(CC[N+](CC1)CCc2ccccc2)c3nnc4c(n3)nno4)c5ccccc5)CC	9.9
232	O=C(C1(N(c2ccccc2)C(=O)CC)CC[N+](CC1)CCc3ccccc3)C4=CCCO4	9.9
233	O=C(C1(N(c2ccccc2)C(=O)CC)CC[N+](CC1)CCc3ccccc3)C(=O)N	9.9
234	O=C(C1(N(c2ccccc2)C(=O)CC)CC[N+](CC1)CCc3ccccc3)CCSC	9.9
235	O=C(N(C1(CC[N+](CC1)CCc2ccccc2)c3nncc(O)n3)c4ccccc4)CC	9.9
236	O=C(NCC#C)C1(N(c2ccccc2)C(=O)CC)CC[N+](CC1)CCc3ccccc3	9.9
237	O=C(N(C1(CC[N+](CC1)CCc2ccccc2)c3nnc4n3nno4)c5ccccc5)CC	9.9
238	O=C(C1(N(c2ccccc2)C(=O)CC)CC[N+](CC1)CCc3ccccc3)C(CC)=C	9.9
239	O=C(C1(N(c2ccccc2)C(=O)CC)CC[N+](CC1)CCc3ccccc3)C/C=C/C	9.9
240	FC(F)(c1nc(no1)C2(N(c3ccccc3)C(=O)CC)CC[N+](CC2)CCc4ccccc4)C	9.9
241	O=C(OCCC(C)C)C1(N(c2ccccc2)C(=O)CC)CC[N+](CC1)CCc3ccccc3	9.9
242	O=C(NCC1CC1)C2(N(c3ccccc3)C(=O)CC)CC[N+](CC2)CCc4ccccc4	9.9
243	O=C(N(C1(SCCC)CC[N+](CC1)CCc2ccccc2)c3ccccc3)CC	9.9
244	O=C(N(C1(CC[N+](CC1)CCc2ccccc2)c3nnc4cncnn43)c5ccccc5)CC	9.9
245	O=C(C1(N(c2ccccc2)C(=O)CC)CC[N+](CC1)CCc3ccccc3)c4c(n[nH]n4)C	9.9
246	O=C(N1CC1)C2(N(c3ccccc3)C(=O)CC)CC[N+](CC2)CCc4ccccc4	9.9
247	FC(F)[C@H]1C[C@@H]1C(=O)C2(N(c3ccccc3)C(=O)CC)CC[N+](CC2)CCc4ccccc4	9.9
248	O=C(OC[C@H]1C[C@@H]1C)C2(N(c3ccccc3)C(=O)CC)CC[N+](CC2)CCc4ccccc4	9.9
249	O=C(OCC(C)=C)C1(N(c2ccccc2)C(=O)CC)CC[N+](CC1)CCc3ccccc3	9.9
250	O=C(NCC(C)C)C1(N(c2ccccc2)C(=O)CC)CC[N+](CC1)CCc3ccccc3	9.9
251	O=C(Oc1cscc1)C2(N(c3ccccc3)C(=O)CC)CC[N+](CC2)CCc4ccccc4	9.9
252	O=C(C1(N(c2ccccc2)C(=O)CC)CC[N+](CC1)CCc3ccccc3)C[C@@H]4C(C4)=C	9.9
253	O=C(N[C@H](C)C#C)C1(N(c2ccccc2)C(=O)CC)CC[N+](CC1)CCc3ccccc3	9.9
254	O=C(N(C1(CC[N+](CC1)CCc2ccccc2)C=3N=CCN3)c4ccccc4)CC	9.9
255	O=C(N(C1(CC[N+](CC1)CCc2ccccc2)c3nnc4c(onn4)n3)c5ccccc5)CC	9.9
256	O=C(C1(N(c2ccccc2)C(=O)CC)CC[N+](CC1)CCc3ccccc3)c4ccncn4	9.9
257	O=C(C1(N(c2ccccc2)C(=O)CC)CC[N+](CC1)CCc3ccccc3)C(=O)C4CC4	9.9
258	F[C@@H]1CC[C@H]1C(=O)C2(N(c3ccccc3)C(=O)CC)CC[N+](CC2)CCc4ccccc4	9.9
259	O=C(C1(N(c2ccccc2)C(=O)CC)CC[N+](CC1)CCc3ccccc3)c4nc(ns4)C	9.9
260	FC(F)(OC1(N(c2ccccc2)C(=O)CC)CC[N+](CC1)CCc3ccccc3)C	9.9
261	O=C(O[C@H]1CSCC1)C2(N(c3ccccc3)C(=O)CC)CC[N+](CC2)CCc4ccccc4	9.9
262	FCCOC(=O)C1(N(c2ccccc2)C(=O)CC)CC[N+](CC1)CCc3ccccc3	9.8
263	O=C(N(C1(CC[N+](CC1)CCc2ccccc2)C=3N=NCN3)c4ccccc4)CC	9.8
264	S=C(OC)C1(N(c2ccccc2)C(=O)CC)CC[N+](CC1)CCc3ccccc3	9.8
265	O=C(NC#C)C1(N(c2ccccc2)C(=O)CC)CC[N+](CC1)CCc3ccccc3	9.8
266	O=C(N(C1(n2nncn2)CC[N+](CC1)CCc3ccccc3)c4ccccc4)CC	9.8
267	F[C@H](C(=O)C1(N(c2ccccc2)C(=O)CC)CC[N+](CC1)CCc3ccccc3)C	9.8
268	O=C(OCSC)C1(N(c2ccccc2)C(=O)CC)CC[N+](CC1)CCc3ccccc3	9.8

269	O/C(C1(N(c2ccccc2)C(=O)CC)CC[N+](CC1)CCc3ccccc3)=C\C	9.8
270	ClC(C(=O)C1(N(c2ccccc2)C(=O)CC)CC[N+](CC1)CCc3ccccc3)=C	9.8
271	Clc1nnc(o1)C2(N(c3ccccc3)C(=O)CC)CC[N+](CC2)CCc4ccccc4	9.8
272	O=C(C1(N(c2ccccc2)C(=O)CC)CC[N+](CC1)CCc3ccccc3)CC#C	9.8
273	O=C(NNC)C1(N(c2ccccc2)C(=O)CC)CC[N+](CC1)CCc3ccccc3	9.8
274	O=C(C1(N(c2ccccc2)C(=O)CC)CC[N+](CC1)CCc3ccccc3)CCC	9.8
275	O=C(C1(N(c2ccccc2)C(=O)CC)CC[N+](CC1)CCc3ccccc3)CN#C	9.8
276	O=C(N(C1(CC[N+](CC1)CCc2ccccc2)c3ncco3)c4ccccc4)CC	9.8
277	O=C(C1(N(c2ccccc2)C(=O)CC)CC[N+](CC1)CCc3ccccc3)C(=O)CC	9.8
278	O=C(N(C1(CC[N+](CC1)CCc2ccccc2)/C=C/CO)c3ccccc3)CC	9.8
279	SCc1nnc(o1)C2(N(c3ccccc3)C(=O)CC)CC[N+](CC2)CCc4ccccc4	9.8
280	FC(F)CCC(=O)C1(N(c2ccccc2)C(=O)CC)CC[N+](CC1)CCc3ccccc3	9.8
281	O=C(N(C1(CC[N+](CC1)CCc2ccccc2)C(SC)=N)c3ccccc3)CC	9.8
282	O=C(OCCCC#C)C1(N(c2ccccc2)C(=O)CC)CC[N+](CC1)CCc3ccccc3	9.8
283	F[C@H](C(=O)C1(N(c2ccccc2)C(=O)CC)CC[N+](CC1)CCc3ccccc3)CC	9.8
284	FC/C=C/C(=O)C1(N(c2ccccc2)C(=O)CC)CC[N+](CC1)CCc3ccccc3	9.8
285	O=C(N(C1(CC[N+](CC1)CCc2ccccc2)C#CC=C)c3ccccc3)CC	9.8
286	O=C(C1(N(c2ccccc2)C(=O)CC)CC[N+](CC1)CCc3ccccc3)CC#CC	9.8
287	O=C(N/N=C/O)C1(N(c2ccccc2)C(=O)CC)CC[N+](CC1)CCc3ccccc3	9.8
288	O=C(n1cccn1)C2(N(c3ccccc3)C(=O)CC)CC[N+](CC2)CCc4ccccc4	9.8
289	FCC(=O)COC(=O)C1(N(c2ccccc2)C(=O)CC)CC[N+](CC1)CCc3ccccc3	9.8
290	FC(F)[C@@H](O)C1(N(c2ccccc2)C(=O)CC)CC[N+](CC1)CCc3ccccc3	9.8
291	Cl[C@H](C(=O)C1(N(c2ccccc2)C(=O)CC)CC[N+](CC1)CCc3ccccc3)CC	9.8
292	O=C(NCCC#C)C1(N(c2ccccc2)C(=O)CC)CC[N+](CC1)CCc3ccccc3	9.8
293	O=C(N(C1(n2nnc(n2)C(C)C)CC[N+](CC1)CCc3ccccc3)c4ccccc4)CC	9.8
294	FC(F)COC(=O)C1(N(c2ccccc2)C(=O)CC)CC[N+](CC1)CCc3ccccc3	9.8
295	O=C(N(C1(n2cc(nn2)C=C)CC[N+](CC1)CCc3ccccc3)c4ccccc4)CC	9.8
296	O=C(C1(N(c2ccccc2)C(=O)CC)CC[N+](CC1)CCc3ccccc3)c4ccco4	9.8
297	O=C(N(C1(n2cc(nn2)CC)CC[N+](CC1)CCc3ccccc3)c4ccccc4)CC	9.8
298	O=C(N(C1(CC[N+](CC1)CCc2ccccc2)/C=C/COC)c3ccccc3)CC	9.8
299	O=C(OCOC(OC)=O)C1(N(c2ccccc2)C(=O)CC)CC[N+](CC1)CCc3ccccc3	9.8
300	O=C(C1(N(c2ccccc2)C(=O)CC)CC[N+](CC1)CCc3ccccc3)C4(CC4)C#C	9.8
301	O=C(C1(N(c2ccccc2)C(=O)CC)CC[N+](CC1)CCc3ccccc3)c4cscn4	9.8
302	FC(F)CNC(=O)C1(N(c2ccccc2)C(=O)CC)CC[N+](CC1)CCc3ccccc3	9.8
303	O=C(N(C1(O/N=C\C)CC[N+](CC1)CCc2ccccc2)c3ccccc3)CC	9.8
304	SCCNC(=O)C1(N(c2ccccc2)C(=O)CC)CC[N+](CC1)CCc3ccccc3	9.8
305	O=C(N(C1(OC2CC2)CC[N+](CC1)CCc3ccccc3)c4ccccc4)CC	9.8
306	O=C(C1(N(c2ccccc2)C(=O)CC)CC[N+](CC1)CCc3ccccc3)c4cc(O)ncn4	9.8
307	O=C(O/C(=C/C)C)C1(N(c2ccccc2)C(=O)CC)CC[N+](CC1)CCc3ccccc3	9.8
308	Fc1cnc(C(=O)C2(N(c3ccccc3)C(=O)CC)CC[N+](CC2)CCc4ccccc4)cc1	9.8
309	F[C@H](OC)C1(N(c2ccccc2)C(=O)CC)CC[N+](CC1)CCc3ccccc3	9.8
310	O=C(C1(N(c2ccccc2)C(=O)CC)CC[N+](CC1)CCc3ccccc3)c4ncco4	9.8

311	O=C(C1(N(c2ccccc2)C(=O)CC)CC[N+](CC1)CCc3ccccc3)[C@H](C)C=C	9.8
312	O=C(C1(N(c2ccccc2)C(=O)CC)CC[N+](CC1)CCc3ccccc3)/C=C/4C[C@@H]4C	9.8
313	O=C(OC[C@@H](CC)C)C1(N(c2ccccc2)C(=O)CC)CC[N+](CC1)CCc3ccccc3	9.8
314	O=C(N(C1(CC[N+](CC1)CCc2ccccc2)c3nnc4nnon43)c5ccccc5)CC	9.8
315	O=C(N(C1(CC[N+](CC1)CCc2ccccc2)/C=C(/OC)C)c3ccccc3)CC	9.8
316	Fc1ccc(o1)C(=O)C2(N(c3ccccc3)C(=O)CC)CC[N+](CC2)CCc4ccccc4	9.8
317	FCOC(=O)C1(N(c2ccccc2)C(=O)CC)CC[N+](CC1)CCc3ccccc3	9.7
318	O=C(NC)C1(N(c2ccccc2)C(=O)CC)CC[N+](CC1)CCc3ccccc3	9.7
319	O=C(ONC)C1(N(c2ccccc2)C(=O)CC)CC[N+](CC1)CCc3ccccc3	9.7
320	FC(F)(F)OC(=O)C1(N(c2ccccc2)C(=O)CC)CC[N+](CC1)CCc3ccccc3	9.7
321	O=C(N(C1(CC[N+](CC1)CCc2ccccc2)c3ncno3)c4ccccc4)CC	9.7
322	FC(C(=O)C1(N(c2ccccc2)C(=O)CC)CC[N+](CC1)CCc3ccccc3)=C	9.7
323	SCCOC(=O)C1(N(c2ccccc2)C(=O)CC)CC[N+](CC1)CCc3ccccc3	9.7
324	O=C(OC[C@H]1CO1)C2(N(c3ccccc3)C(=O)CC)CC[N+](CC2)CCc4ccccc4	9.7
325	O=C(OCCCC)C1(N(c2ccccc2)C(=O)CC)CC[N+](CC1)CCc3ccccc3	9.7
326	F[C@@H]1C[C@H]1C(=O)C2(N(c3ccccc3)C(=O)CC)CC[N+](CC2)CCc4ccccc4	9.7
327	O=C(/N=C/CC)C1(N(c2ccccc2)C(=O)CC)CC[N+](CC1)CCc3ccccc3	9.7
328	O=C(N(C1(CC[N+](CC1)CCc2ccccc2)C3OCCO3)c4ccccc4)CC	9.7
329	O=C(C1(N(c2ccccc2)C(=O)CC)CC[N+](CC1)CCc3ccccc3)CCC#C	9.7
330	FCc1nc(no1)C2(N(c3ccccc3)C(=O)CC)CC[N+](CC2)CCc4ccccc4	9.7
331	O=C(C1(N(c2ccccc2)C(=O)CC)CC[N+](CC1)CCc3ccccc3)CC=C	9.7
332	FCCCOC(=O)C1(N(c2ccccc2)C(=O)CC)CC[N+](CC1)CCc3ccccc3	9.7
333	O=C(N(C1(CC[N+](CC1)CCc2ccccc2)C3(N=N3)C)c4ccccc4)CC	9.7
334	FCCNC(=O)C1(N(c2ccccc2)C(=O)CC)CC[N+](CC1)CCc3ccccc3	9.7
335	O=C(C1(N(c2ccccc2)C(=O)CC)CC[N+](CC1)CCc3ccccc3)[C@@H]4[C@H](O4)C	9.7
336	FCCCC(=O)C1(N(c2ccccc2)C(=O)CC)CC[N+](CC1)CCc3ccccc3	9.7
337	O=C(N(C1(CC[N+](CC1)CCc2ccccc2)[C@H]3N=C(O)C=N3)c4ccccc4)CC	9.7
338	O=C(NNCC)C1(N(c2ccccc2)C(=O)CC)CC[N+](CC1)CCc3ccccc3	9.7
339	O=C(N(C1(n2cccn2)CC[N+](CC1)CCc3ccccc3)c4ccccc4)CC	9.7
340	O=C(C1(N(c2ccccc2)C(=O)CC)CC[N+](CC1)CCc3ccccc3)CSC	9.7
341	O=C(OCCC#CC)C1(N(c2ccccc2)C(=O)CC)CC[N+](CC1)CCc3ccccc3	9.7
342	O=C(N(C1(CC[N+](CC1)CCc2ccccc2)c3ccn[nH]3)c4ccccc4)CC	9.7
343	O=C(N(C1(/N=N/CC)CC[N+](CC1)CCc2ccccc2)c3ccccc3)CC	9.7
344	O=C(OCC(OC)=O)C1(N(c2ccccc2)C(=O)CC)CC[N+](CC1)CCc3ccccc3	9.7
345	O=C(C1(N(c2ccccc2)C(=O)CC)CC[N+](CC1)CCc3ccccc3)CC(C)=C	9.7
346	O=C(C1(N(c2ccccc2)C(=O)CC)CC[N+](CC1)CCc3ccccc3)/C=C/C	9.7
347	O=C(C1(N(c2ccccc2)C(=O)CC)CC[N+](CC1)CCc3ccccc3)[C@@H]4C=CCO4	9.7
348	O=C(C1(N(c2ccccc2)C(=O)CC)CC[N+](CC1)CCc3ccccc3)/C=C/C=C	9.7
349	FC(F)(F)CCC(=O)C1(N(c2ccccc2)C(=O)CC)CC[N+](CC1)CCc3ccccc3	9.7
350	BrC(C(=O)C1(N(c2ccccc2)C(=O)CC)CC[N+](CC1)CCc3ccccc3)=C	9.7
351	O=C(N(C1(CC[N+](CC1)CCc2ccccc2)c3c(non3)N#C)c4ccccc4)CC	9.7
352	O=C(OCOCC)C1(N(c2ccccc2)C(=O)CC)CC[N+](CC1)CCc3ccccc3	9.7

353	O=S(=O)(C1(N(c2ccccc2)C(=O)CC)CC[N+](CC1)CCc3ccccc3)C=C	9.7
354	O=C(N[C@H]([N+])C)C1(N(c2ccccc2)C(=O)CC)CC[N+](CC1)CCc3ccccc3	9.7
355	F/C(C(=O)C1(N(c2ccccc2)C(=O)CC)CC[N+](CC1)CCc3ccccc3)=C\OC	9.7
356	O=C(N(C1(N2CC=CO2)CC[N+](CC1)CCc3ccccc3)c4ccccc4)CC	9.7
357	O=C(C1(N(c2ccccc2)C(=O)CC)CC[N+](CC1)CCc3ccccc3)/C=C/SC	9.7
358	O=C(N(C1(ON(C)C)CC[N+](CC1)CCc2ccccc2)c3ccccc3)CC	9.7
359	O=C(N(C1(CC[N+](CC1)CCc2ccccc2)/C=C/C(C)=C)c3ccccc3)CC	9.7
360	O=C(O[C@@H]1COCC1)C2(N(c3ccccc3)C(=O)CC)CC[N+](CC2)CCc4ccccc4	9.7
361	O=C(ON)C1(N(c2ccccc2)C(=O)CC)CC[N+](CC1)CCc3ccccc3	9.6
362	FCCC(=O)C1(N(c2ccccc2)C(=O)CC)CC[N+](CC1)CCc3ccccc3	9.6
363	O=C(C1(N(c2ccccc2)C(=O)CC)CC[N+](CC1)CCc3ccccc3)/C=C\O	9.6
364	O=C(C1(N(c2ccccc2)C(=O)CC)CC[N+](CC1)CCc3ccccc3)B(O)O	9.6
365	O=C(NN)C1(N(c2ccccc2)C(=O)CC)CC[N+](CC1)CCc3ccccc3	9.6
366	Clc1nc(no1)C2(N(c3ccccc3)C(=O)CC)CC[N+](CC2)CCc4ccccc4	9.6
367	O=C(C1(N(c2ccccc2)C(=O)CC)CC[N+](CC1)CCc3ccccc3)[C@@H]4CS4	9.6
368	O=C(C1(N(c2ccccc2)C(=O)CC)CC[N+](CC1)CCc3ccccc3)C#C	9.6
369	O=C(NCC)C1(N(c2ccccc2)C(=O)CC)CC[N+](CC1)CCc3ccccc3	9.6
370	O=C(N(C1(CC[N+](CC1)CCc2ccccc2)c3nnncn3)c4ccccc4)CC	9.6
371	O=C(N/C=C\C)C1(N(c2ccccc2)C(=O)CC)CC[N+](CC1)CCc3ccccc3	9.6
372	O=C(N(C1(CC[N+](CC1)CCc2ccccc2)/C=C/C=C)c3ccccc3)CC	9.6
373	O=C([S@@](=O)CC)C1(N(c2ccccc2)C(=O)CC)CC[N+](CC1)CCc3ccccc3	9.6
374	FC(F)OCC1(N(c2ccccc2)C(=O)CC)CC[N+](CC1)CCc3ccccc3	9.6
375	O=C(NCCC)C1(N(c2ccccc2)C(=O)CC)CC[N+](CC1)CCc3ccccc3	9.6
376	O=C(N(C1(CC[N+](CC1)CCc2ccccc2)[C@@H]3O[C@H](CO3)C)c4ccccc4)CC	9.6
377	O=C(N(C1(CC[N+](CC1)CCc2ccccc2)c3nonn3)c4ccccc4)CC	9.6
378	O=C(N(C1(CC[N+](CC1)CCc2ccccc2)/C=C/O)c3ccccc3)CC	9.6
379	FC(C(=O)C1(N(c2ccccc2)C(=O)CC)CC[N+](CC1)CCc3ccccc3)(C)C	9.6
380	O=C(C1(N(c2ccccc2)C(=O)CC)CC[N+](CC1)CCc3ccccc3)c4cnon4	9.6
381	O=C(N(C1(CC[N+](CC1)CCc2ccccc2)C=3C=CC3)c4ccccc4)CC	9.6
382	O=C(C1(N(c2ccccc2)C(=O)CC)CC[N+](CC1)CCc3ccccc3)C4=CC4	9.6
383	O=C(C1(N(c2ccccc2)C(=O)CC)CC[N+](CC1)CCc3ccccc3)C=O	9.6
384	F[C@]1(C2(N(c3ccccc3)C(=O)CC)CC[N+](CC2)CCc4ccccc4)C(F)(F)O1	9.6
385	O=C(N(C1(CC[N+](CC1)CCc2ccccc2)/C=C/C(=O)N)c3ccccc3)CC	9.6
386	O=C(N(C1(CC[N+](CC1)CCc2ccccc2)[C@H]3C=CCO3)c4ccccc4)CC	9.6
387	FC(C1(N(c2ccccc2)C(=O)CC)CC[N+](CC1)CCc3ccccc3)=C	9.6
388	O=S(=O)(NC)C1(N(c2ccccc2)C(=O)CC)CC[N+](CC1)CCc3ccccc3	9.6
389	O=C(N(C1(CC[N+](CC1)CCc2ccccc2)c3nnc(o3)C=C)c4ccccc4)CC	9.6
390	O=C(C1(N(c2ccccc2)C(=O)CC)CC[N+](CC1)CCc3ccccc3)C(OC)=O	9.6
391	S=COC1(N(c2ccccc2)C(=O)CC)CC[N+](CC1)CCc3ccccc3	9.6
392	O=C(N(C1(n2nccn2)CC[N+](CC1)CCc3ccccc3)c4ccccc4)CC	9.6
393	O=C(C1(N(c2ccccc2)C(=O)CC)CC[N+](CC1)CCc3ccccc3)[C@@H]4C[C@H]4C#C	9.6
394	O=C(NCC#CC)C1(N(c2ccccc2)C(=O)CC)CC[N+](CC1)CCc3ccccc3	9.6

395	OC(C1(N(c2ccccc2)C(=O)CC)CC[N+](CC1)CCc3ccccc3)C[N+]([O-])O	9.6
396	OC(C1(N(c2ccccc2)C(=O)CC)CC[N+](CC1)CCc3ccccc3)=C	9.6
397	FC1(C(=O)C2(N(c3ccccc3)C(=O)CC)CC[N+](CC2)CCc4ccccc4)CCC1	9.6
398	S=C(OC1(N(c2ccccc2)C(=O)CC)CC[N+](CC1)CCc3ccccc3)OC	9.6
399	SC(=N)C1(N(c2ccccc2)C(=O)CC)CC[N+](CC1)CCc3ccccc3	9.6
400	O=C(N(C1(CC[N+](CC1)CCc2ccccc2)CN=N)c3ccccc3)CC	9.6
401	O=C(NC1CCC1)C2(N(c3ccccc3)C(=O)CC)CC[N+](CC2)CCc4ccccc4	9.6
402	O=C(N(C1(/N=C/CC)CC[N+](CC1)CCc2ccccc2)c3ccccc3)CC	9.6
403	O=C(C1(N(c2ccccc2)C(=O)CC)CC[N+](CC1)CCc3ccccc3)/C=C/CC	9.6
404	O=C(N=C)C1(N(c2ccccc2)C(=O)CC)CC[N+](CC1)CCc3ccccc3	9.5
405	O=C(C1(N(c2ccccc2)C(=O)CC)CC[N+](CC1)CCc3ccccc3)C=C	9.5
406	O=C(C1(N(c2ccccc2)C(=O)CC)CC[N+](CC1)CCc3ccccc3)[C@@H]4CO4	9.5
407	O=C(C1(N(c2ccccc2)C(=O)CC)CC[N+](CC1)CCc3ccccc3)C	9.5
408	O=C(C1(N(c2ccccc2)C(=O)CC)CC[N+](CC1)CCc3ccccc3)C4N=N4	9.5
409	Cl/C=C/C(=O)C1(N(c2ccccc2)C(=O)CC)CC[N+](CC1)CCc3ccccc3	9.5
410	FCSC(=O)C1(N(c2ccccc2)C(=O)CC)CC[N+](CC1)CCc3ccccc3	9.5
411	O=C(NOC)C1(N(c2ccccc2)C(=O)CC)CC[N+](CC1)CCc3ccccc3	9.5
412	O=C(N(C1(CC[N+](CC1)CCc2ccccc2)/C=C/CC)c3ccccc3)CC	9.5
413	O=C(N[C@@H]1CO1)C2(N(c3ccccc3)C(=O)CC)CC[N+](CC2)CCc4ccccc4	9.5
414	O=C(OCC[N+])C1(N(c2ccccc2)C(=O)CC)CC[N+](CC1)CCc3ccccc3	9.5
415	O=C(N(C1([C@@]2(CO2)C)CC[N+](CC1)CCc3ccccc3)c4ccccc4)CC	9.5
416	O=C(N(C1(ONC)CC[N+](CC1)CCc2ccccc2)c3ccccc3)CC	9.5
417	SCC(=O)C1(N(c2ccccc2)C(=O)CC)CC[N+](CC1)CCc3ccccc3	9.5
418	O=C(N(C1(OC=C)CC[N+](CC1)CCc2ccccc2)c3ccccc3)CC	9.5
419	ClC(C1(N(c2ccccc2)C(=O)CC)CC[N+](CC1)CCc3ccccc3)=C	9.5
420	O=C(NO)C1(N(c2ccccc2)C(=O)CC)CC[N+](CC1)CCc3ccccc3	9.5
421	O=C(N(C1(CC[N+](CC1)CCc2ccccc2)C#CC)c3ccccc3)CC	9.5
422	O=C(N(C1(N2C(OC=N2)=O)CC[N+](CC1)CCc3ccccc3)c4ccccc4)CC	9.5
423	O=C(N(C1(OC#C)CC[N+](CC1)CCc2ccccc2)c3ccccc3)CC	9.5
424	O=C(NCB(O)O)C1(N(c2ccccc2)C(=O)CC)CC[N+](CC1)CCc3ccccc3	9.5
425	O=C(NCC[N+])C1(N(c2ccccc2)C(=O)CC)CC[N+](CC1)CCc3ccccc3	9.5
426	OC(=N)C1(N(c2ccccc2)C(=O)CC)CC[N+](CC1)CCc3ccccc3	9.5
427	O=C(N(C1(CC[N+](CC1)CCc2ccccc2)C=C3CC3)c4ccccc4)CC	9.5
428	FCC(=O)C(=O)C1(N(c2ccccc2)C(=O)CC)CC[N+](CC1)CCc3ccccc3	9.5
429	O=C(C1(N(c2ccccc2)C(=O)CC)CC[N+](CC1)CCc3ccccc3)c4cn[nH]n4	9.5
430	O=C(C1(N(c2ccccc2)C(=O)CC)CC[N+](CC1)CCc3ccccc3)[C@@H](O)C	9.5
431	O=C(OCCCCC)C1(N(c2ccccc2)C(=O)CC)CC[N+](CC1)CCc3ccccc3	9.5
432	BrC#CC1(N(c2ccccc2)C(=O)CC)CC[N+](CC1)CCc3ccccc3	9.5
433	SC(=O)C1(N(c2ccccc2)C(=O)CC)CC[N+](CC1)CCc3ccccc3	9.4
434	FC(F)OC(=O)C1(N(c2ccccc2)C(=O)CC)CC[N+](CC1)CCc3ccccc3	9.4
435	O=C(NC=C)C1(N(c2ccccc2)C(=O)CC)CC[N+](CC1)CCc3ccccc3	9.4
436	O=C(NC[N+])C1(N(c2ccccc2)C(=O)CC)CC[N+](CC1)CCc3ccccc3	9.4

437	O=C(OC[N+])C1(N(c2ccccc2)C(=O)CC)CC[N+](CC1)CCc3ccccc3	9.4
438	O=C(N(C1(CC[N+](CC1)CCc2ccccc2)/C=C/C)c3ccccc3)CC	9.4
439	Cl/C=C/C1(N(c2ccccc2)C(=O)CC)CC[N+](CC1)CCc3ccccc3	9.4
440	BrC(C1(N(c2ccccc2)C(=O)CC)CC[N+](CC1)CCc3ccccc3)=C	9.4
441	Br/C=C/C1(N(c2ccccc2)C(=O)CC)CC[N+](CC1)CCc3ccccc3	9.4
442	O=C(C1(N(c2ccccc2)C(=O)CC)CC[N+](CC1)CCc3ccccc3)C#N	9.4
443	O[C@H](C1(N(c2ccccc2)C(=O)CC)CC[N+](CC1)CCc3ccccc3)C#C	9.4
444	O=C(N(C1([C@H]2CCN(O2)C)CC[N+](CC1)CCc3ccccc3)c4ccccc4)CC	9.4
445	FCSC1(N(c2ccccc2)C(=O)CC)CC[N+](CC1)CCc3ccccc3	9.4
446	O=C(N(C1(CC[N+](CC1)CCc2ccccc2)/C=C/OC)c3ccccc3)CC	9.4
447	S=C1NC(=NO1)C2(N(c3ccccc3)C(=O)CC)CC[N+](CC2)CCc4ccccc4	9.4
448	O=C(C1(N(c2ccccc2)C(=O)CC)CC[N+](CC1)CCc3ccccc3)C[N+]C	9.4
449	FC(F)C(=O)C1(N(c2ccccc2)C(=O)CC)CC[N+](CC1)CCc3ccccc3	9.3
450	O=C(N)C1(N(c2ccccc2)C(=O)CC)CC[N+](CC1)CCc3ccccc3	9.3
451	S=C(N)C(=O)C1(N(c2ccccc2)C(=O)CC)CC[N+](CC1)CCc3ccccc3	9.3
452	FC(F)(F)COC(=O)C1(N(c2ccccc2)C(=O)CC)CC[N+](CC1)CCc3ccccc3	9.3
453	Cl[C@@H](F)C(=O)C1(N(c2ccccc2)C(=O)CC)CC[N+](CC1)CCc3ccccc3	9.3
454	O=C(O[C@H](CO)C)C1(N(c2ccccc2)C(=O)CC)CC[N+](CC1)CCc3ccccc3	9.3
455	O=C(C1(N(c2ccccc2)C(=O)CC)CC[N+](CC1)CCc3ccccc3)CN=N	9.3
456	O=C(N(C1([C@H]2[C@@H](O2)C(C)=C)CC[N+](CC1)CCc3ccccc3)c4ccccc4)CC	9.3
457	SSC1(N(c2ccccc2)C(=O)CC)CC[N+](CC1)CCc3ccccc3	9.3
458	FCCSC1(N(c2ccccc2)C(=O)CC)CC[N+](CC1)CCc3ccccc3	9.3
459	O=[S@@](C1(N(c2ccccc2)C(=O)CC)CC[N+](CC1)CCc3ccccc3)C=C	9.3
460	O=C(OCC(=O)C)C1(N(c2ccccc2)C(=O)CC)CC[N+](CC1)CCc3ccccc3	9.3
461	O=C(N(C1(/NN\N)CC[N+](CC1)CCc2ccccc2)c3ccccc3)CC	9.3
462	O=C(N(C1(SSC)CC[N+](CC1)CCc2ccccc2)c3ccccc3)CC	9.3
463	O=C(N(C=C)C=C)C1(N(c2ccccc2)C(=O)CC)CC[N+](CC1)CCc3ccccc3	9.3
464	OC(N)C1(N(c2ccccc2)C(=O)CC)CC[N+](CC1)CCc3ccccc3	9.2
465	O=C(N(C1(CC[N+](CC1)CCc2ccccc2)c3nnco3)c4ccccc4)CC	9.2
466	O=C(N(C1(CC[N+](CC1)CCc2ccccc2)C3=CC3)c4ccccc4)CC	9.2
467	O=C(OCCO)C1(N(c2ccccc2)C(=O)CC)CC[N+](CC1)CCc3ccccc3	9.2
468	FCC(=O)C1(N(c2ccccc2)C(=O)CC)CC[N+](CC1)CCc3ccccc3	9.2
469	O=C(N(C1(CC[N+](CC1)CCc2ccccc2)[C@H]3CCO3)c4ccccc4)CC	9.2
470	O=C(N(C1(CC[N+](CC1)CCc2ccccc2)c3nnc(o3)C)c4ccccc4)CC	9.2
471	O=C(N(C1([C@H]2[C@@H](O2)CC)CC[N+](CC1)CCc3ccccc3)c4ccccc4)CC	9.2
472	O=[S@@](N)C1(N(c2ccccc2)C(=O)CC)CC[N+](CC1)CCc3ccccc3	9.2
473	O=C(N(C1(CC[N+](CC1)CCc2ccccc2)C3N=N3)c4ccccc4)CC	9.2
474	F/C(=N\OC)C1(N(c2ccccc2)C(=O)CC)CC[N+](CC1)CCc3ccccc3	9.2
475	O=C(N(C1(N=N)CC[N+](CC1)CCc2ccccc2)c3ccccc3)CC	9.2
476	O=C(N(C1(CC[N+](CC1)CCc2ccccc2)C(=N)C)c3ccccc3)CC	9.2
477	O=C(N(C1(N2N=N2)CC[N+](CC1)CCc3ccccc3)c4ccccc4)CC	9.2
478	IC1(N(c2ccccc2)C(=O)CC)CC[N+](CC1)CCc3ccccc3	9.2

479	O=C(N(C1(SC)CC[N+](CC1)CCc2ccccc2)c3ccccc3)CC	9.2
480	O=C(N(C1(SCCC#C)CC[N+](CC1)CCc2ccccc2)c3ccccc3)CC	9.2
481	ON(C1(N(c2ccccc2)C(=O)CC)CC[N+](CC1)CCc3ccccc3)C=N	9.2
482	SC1(N(c2ccccc2)C(=O)CC)CC[N+](CC1)CCc3ccccc3	9.1
483	BrC1(N(c2ccccc2)C(=O)CC)CC[N+](CC1)CCc3ccccc3	9.1
484	O=C(N(C1(CC[N+](CC1)CCc2ccccc2)/C=C=C)c3ccccc3)CC	9.1
485	O=C(N(C1(CC[N+](CC1)CCc2ccccc2)[C@H]3C=NC=N3)c4ccccc4)CC	9.1
486	O=C(N(C1(SC#C)CC[N+](CC1)CCc2ccccc2)c3ccccc3)CC	9.1
487	O=C(N(C1(SO)CC[N+](CC1)CCc2ccccc2)c3ccccc3)CC	9.0
488	O=C(N(C1(NC=C)CC[N+](CC1)CCc2ccccc2)c3ccccc3)CC	9.0
489	O=C(N(C1(CC[N+](CC1)CCc2ccccc2)/C=C/[S@](=O)C)c3ccccc3)CC	9.0
490	O=C(N(C1([C@H]2[C@@H](O2)C)CC[N+](CC1)CCc3ccccc3)c4ccccc4)CC	8.9
491	O=CC1(N(c2ccccc2)C(=O)CC)CC[N+](CC1)CCc3ccccc3	8.9
492	ClC1(N(c2ccccc2)C(=O)CC)CC[N+](CC1)CCc3ccccc3	8.9
493	O=C(N(C1(CC[N+](CC1)CCc2ccccc2)C#C)c3ccccc3)CC	8.9
494	FC1(N(c2ccccc2)C(=O)CC)CC[N+](CC1)CCc3ccccc3	8.9
495	O=C(N(C1(NC#C)CC[N+](CC1)CCc2ccccc2)c3ccccc3)CC	8.8
496	FC(F)(O)C1(N(c2ccccc2)C(=O)CC)CC[N+](CC1)CCc3ccccc3	8.8
497	S=CNC1(N(c2ccccc2)C(=O)CC)CC[N+](CC1)CCc3ccccc3	8.8
498	O=C(OCC#N)C1(N(c2ccccc2)C(=O)CC)CC[N+](CC1)CCc3ccccc3	8.7
499	O=C(N(C1([C@H]2CO2)CC[N+](CC1)CCc3ccccc3)c4ccccc4)CC	8.6
500	O=C(N(C1(CC[N+](CC1)CCc2ccccc2)/C=N/OC)c3ccccc3)CC	8.6

Table S7

List, SMILE and predicted pK_i values for Series 5.

N°	SMILES	Pred pK_i
1	O=C(N(C1(CC[N+](CC1)CCc2ccccc2)CC(OC)=O)c3ccccc3)CC	9.8
2	O=C(N(C1(CC[N+](CC1)CCc2ccccc2)CCCC)c3ccccc3)CC	9.7
3	O=C(N(C1(CC[N+](CC1)CCc2ccccc2)CC(C)C)c3ccccc3)CC	9.7
4	O=C(N([N+]1(CCCC1)CC(=O)NCCc2ccccc2)c3ccccc3)CC	9.7
5	O=C(N([C@]([N+]C)(CSCCCc1ccccc1)C)c2ccccc2)CC	9.7
6	O=C(N(C1([N+][C@@H](CC)C)CC[N+](CC1)CCc2ccccc2)c3ccccc3)CC	9.7
7	O=C(N(C1(CC[N+](CC1)CCc2ccccc2)CCC)c3ccccc3)CC	9.6
8	O=C(N(C1(CC2CC2)CC[N+](CC1)CCc3ccccc3)c4ccccc4)CC	9.6
9	O=C(N(C1(CC[N+](CC1)CCc2ccccc2)CNC(=O)C)c3ccccc3)CC	9.6
10	O=C(N([C@@]1(C2CCC2)CC[N+](C[C@H]1C)CCc3ccccc3)c4ccccc4)CC	9.6
11	O=C(N(C1([N+](CCCCc2ccccc2)C)CCCC1)c3ccccc3)CC	9.6
12	O=C(N(C1([N+]CCC)CC[N+](CC1)CCc2ccccc2)c3ccccc3)CC	9.5
13	O=C(N(C1(CC[N+](CC1)CCc2ccccc2)C[N+](C)C)c3ccccc3)CC	9.5
14	O[C@H]1C[C@@H](CC[C@@H]1N(c2ccccc2)C(=O)CC)CCc3ccccc3	9.5
15	O=C(N(C1([N+]CC(C)C)CC[N+](CC1)CCc2ccccc2)c3ccccc3)CC	9.5

16	O=C(N([C@@H]([C@@H](CC)C)CC(=O)NCCc1ccccc1)c2ccccc2)CC	9.5
17	O=C(N([C@@]1(CO[C@@H](C[N+]1)CCc2ccccc2)CC)c3ccccc3)CC	9.5
18	O=C(N(C1(CC[N+](CC1)CCc2ccccc2)C(C)C)c3ccccc3)CC	9.4
19	O=C(N([C@@]1(C2CC2)CCC[N+](CC1)CCc3ccccc3)c4ccccc4)CC	9.4
20	O=C(N([C@H](CC(=O)NCCc1ccccc1)C=C)c2ccccc2)CC	9.4
21	O=C(N(C1(CCC(CC1)CCc2ccccc2)C[N+])c3ccccc3)CC	9.4
22	O=C(NCCc1ccccc1)[C@H](N(N(c2ccccc2)C(=O)CC)C)C	9.4
23	S=C(SCN(c1ccccc1)C(=O)CC)NCCc2ccccc2	9.4
24	SC[C@@H]([C@@H](N(c1ccccc1)C(=O)CC)C)C(=O)NCCc2ccccc2	9.4
25	O=C(N([C@]1(C[N+]CCc2ccccc2)CCC[N+]C1)c3ccccc3)CC	9.4
26	O[C@@H]1C[N+](C[C@@]1(N(c2ccccc2)C(=O)CC)CSC)CCc3ccccc3	9.4
27	O[C@]1(N(c2ccccc2)C(=O)CC)CC[N+](C[C@@H]1CC(C)C)CCc3ccccc3	9.4
28	O=C(N(C1(CC[N+](CC1)CCc2ccccc2)C([O-])=O)c3ccccc3)CC	9.4
29	O=C(NCCc1ccccc1)[C@H](N(N(c2ccccc2)C(=O)CC)CC)C	9.4
30	O=C(N([C@@]1(CCC[N+](CC1)CCc2ccccc2)CC)c3ccccc3)CC	9.3
31	O=C(N([C@@]12CCCC[C@@H]2C[N+](CC1)CCc3ccccc3)c4ccccc4)CC	9.3
32	O=C(N(C1([N+]C2CC2)CC[N+](CC1)CCc3ccccc3)c4ccccc4)CC	9.3
33	O=C(NO)C1(N(c2ccccc2)C(=O)CC)CC[N+](CC1)CCc3ccccc3	9.3
34	O=C(C1(N(c2ccccc2)C(=O)CC)CC[N+](CC1)CCc3ccccc3)CC	9.3
35	O=C(N([C@@H](C(C)C)CC(=O)NCCc1ccccc1)c2ccccc2)CC	9.3
36	O=C(N([C@H]([N+]([C@H](CCCc1ccccc1)C)CC)C)c2ccccc2)CC	9.3
37	O=C(N(C1(CCC1)CC(=O)NCCc2ccccc2)c3ccccc3)CC	9.3
38	O=C(N(C1(n2cccn2)CC[N+](CC1)CCc3ccccc3)c4ccccc4)CC	9.3
39	O=C(N(C1(OC(=O)CC)CC[N+](CC1)CCc2ccccc2)c3ccccc3)CC	9.3
40	O=C(N(C1(n2cncc2)CC[N+](CC1)CCc3ccccc3)c4ccccc4)CC	9.3
41	O=C(N(SCC(=O)NCCc1ccccc1)c2ccccc2)CC	9.3
42	S[C@@H]([C@@H](N(c1ccccc1)C(=O)CC)C)C(=O)NCCc2ccccc2	9.3
43	O=C(N(C[N+](CC(=O)NCCc1ccccc1)CC#C)c2ccccc2)CC	9.3
44	O=C(N([S@](=O)CC(=O)NCCc1ccccc1)c2ccccc2)CC	9.3
45	O[C@@H]1C[C@H]([C@H](O[N+]([O-])=O)C[C@H]1N(c2ccccc2)C(=O)CC)CCc3ccccc3	9.3
46	O=C(N([C@]1(CCC[N+]1CCNCCc2ccccc2)C)c3ccccc3)CC	9.3
47	O=C(N(N(CC(=O)NCCc1ccccc1)CC#C)c2ccccc2)CC	9.3
48	O=C(N([C@@H]([N+](CC)CC(OCCc1ccccc1)=O)C)c2ccccc2)CC	9.3
49	O=C(N([C@H]([N+](C)C)CNCCCc1ccccc1)c2ccccc2)CC	9.3
50	O=C(N(C1([N+]C)CCC(CC1)CCc2ccccc2)c3ccccc3)CC	9.3
51	O=C(N(C1(OCC)CC[N+](CC1)CCc2ccccc2)c3ccccc3)CC	9.2
52	OC[C@H]1C[N+](CC[C@@H]1N(c2ccccc2)C(=O)CC)CCc3ccccc3	9.2
53	O=C(N(C1(CC[N+](CC1)CCc2ccccc2)C[N+]C)c3ccccc3)CC	9.2
54	O=C(N(C1(OC(=O)C)CC[N+](CC1)CCc2ccccc2)c3ccccc3)CC	9.2
55	O=C(N(C1(CC1)CC(=O)NCCc2ccccc2)c3ccccc3)CC	9.2
56	O=C(N([C@@]1(C2CC2)CC[N+](C[C@H]1C)CCc3ccccc3)c4ccccc4)CC	9.2
57	O=C(N([C@@H](CC)CC(=O)NCCc1ccccc1)c2ccccc2)CC	9.2

58	O=C(N([C@H]1CC[N+](C[C@@H]1CCCC)CCc2ccccc2)c3ccccc3)CC	9.2
59	O=C(N([C@@H](C[N+])CC(OCCc1ccccc1)=O)c2ccccc2)CC	9.2
60	O[C@H]1[C@@H](O)[C@H](O)[C@@H](N(c2ccccc2)C(=O)CC)C[N+][C@H]1CCc3ccccc3	9.2
61	O=C(N)C1(N(c2ccccc2)C(=O)CC)CC[N+](CC1)CCc3ccccc3	9.2
62	O=C(N([C@@H](C1CC1)CC(=O)NCCc2ccccc2)c3ccccc3)CC	9.2
63	O=C(NCCc1ccccc1)[C@@H](CCN(c2ccccc2)C(=O)CC)C	9.2
64	O=C(N(N(CC)CC(=O)NCCc1ccccc1)c2ccccc2)CC	9.2
65	O=C(N(C1(CC[N+](CC1)CCc2ccccc2)COC(=O)C)c3ccccc3)CC	9.2
66	O=C(N([C@]1(NCCC)C[N+](CC1)CCc2ccccc2)c3ccccc3)CC	9.2
67	O=C(N([C@@H](CCC)CC(=O)NCCc1ccccc1)c2ccccc2)CC	9.2
68	S=C(N(CN(c1ccccc1)C(=O)CC)CC)NCCc2ccccc2	9.2
69	O=C(N(S[C@@H](CCCc1ccccc1)C#N)c2ccccc2)CC	9.2
70	O=C(N([C@@H](NC)C[N+](CCc1ccccc1)C)c2ccccc2)CC	9.2
71	O=C(N([C@]1(CC[N+](C[C@H]1C)CCc2ccccc2)CCOC)c3ccccc3)CC	9.2
72	O=C(N([C@@H]([N+]C(C)C)CC(=O)NCCc1ccccc1)c2ccccc2)CC	9.2
73	O=C(N(CCS[C@H]([C@@H]([N+])C)CCc1ccccc1)c2ccccc2)CC	9.2
74	O=C(N(C[N+](CC)CC(=O)CCCc1ccccc1)c2ccccc2)CC	9.2
75	S=C(OCN(c1ccccc1)C(=O)CC)NCCc2ccccc2	9.2
76	O=C(N([C@H]1CC[C@H]([C@H](n2cncn2)C1)CCc3ccccc3)c4ccccc4)CC	9.2
77	O=C(NCCc1ccccc1)[C@@H](CCN(c2ccccc2)C(=O)CC)CC	9.2
78	O=C(N([C@H]([C@@H]([N+]CCCc1ccccc1)C(=O)N)C)c2ccccc2)CC	9.2
79	OC1(N(c2ccccc2)C(=O)CC)CC[N+](CC1)CCc3ccccc3	9.1
80	O=C(N(C1(CC[N+](CC1)CCc2ccccc2)CC)c3ccccc3)CC	9.1
81	O=S1(=O)C[C@H]([N+]C[C@H]1CCc2ccccc2)N(c3ccccc3)C(=O)CC	9.1
82	S=C(S[C@@H](N(c1ccccc1)C(=O)CC)C)NCCc2ccccc2	9.1
83	O=C(N([C@@H]1C(=O)C[N+](CC1)CCc2ccccc2)c3ccccc3)CC	9.1
84	O=C(N([C@H]([N+]1CC[C@@H](C1)CCc2ccccc2)C)c3ccccc3)CC	9.1
85	O=C(OCC)C1(N(c2ccccc2)C(=O)CC)CC[N+](CC1)CCc3ccccc3	9.1
86	O=C(N(SCC(=O)[C@@H](CCc1ccccc1)C#N)c2ccccc2)CC	9.1
87	O=C(N(C1([N+])CCC(CC1)CCc2ccccc2)c3ccccc3)CC	9.1
88	O[C@@H](N(c1ccccc1)C(=O)CC)[C@@H]([N+])C(=O)NCCc2ccccc2	9.1
89	O=C(N(CCC(=O)NCCc1ccccc1)c2ccccc2)CC	9.1
90	O=C(N(C(C[C@H]([N+]CCc1ccccc1)C)(C)C)c2ccccc2)CC	9.1
91	O=C(N([C@]1(C[N+](CC1)CCc2ccccc2)C[N+](C)C)c3ccccc3)CC	9.1
92	O=C(NCCc1ccccc1)[C@@H]2C[N+]C[C@H]2N(c3ccccc3)C(=O)CC	9.1
93	O=C(N([C@@](NC)(C1CC1)C[N+](CCc2ccccc2)C)c3ccccc3)CC	9.1
94	O=C(N(C1([N+](CC)CC)CC[N+](CC1)CCc2ccccc2)c3ccccc3)CC	9.1
95	O=C(N(C1([N+][C@@H](CCc2ccccc2)C)CCC1)c3ccccc3)CC	9.1
96	O=S(=O)(N[C@H](CN(c1ccccc1)C(=O)CC)C[N+])CCc2ccccc2	9.1
97	O=C(N([C@H]1C=C(CC[N+]1C)CCc2ccccc2)c3ccccc3)CC	9.1
98	S=C(N(c1ccccc1)C(=O)CC)C[C@H]([N+](CCc2ccccc2)C)C	9.1
99	OC[C@@]([N+])(CCN(c1ccccc1)C(=O)CC)CCCc2ccccc2	9.1

100	O=C(N([C@@H]([N+]CC(=O)NCCc1ccccc1)C(C)C)c2ccccc2)CC	9.1
101	O=C(NCCc1ccccc1)[C@@H]2C[C@@H]2N(c3ccccc3)C(=O)CC	9.1
102	O=C(N([C@]1(C[N+]CCc2ccccc2)COCCC1)c3ccccc3)CC	9.1
103	SC[C@H](CCN(c1ccccc1)C(=O)CC)C(=O)NCCc2ccccc2	9.1
104	O=C(N(C1(SC)CC[N+](CC1)CCc2ccccc2)c3ccccc3)CC	9.0
105	O=C(C1(N(c2ccccc2)C(=O)CC)CC[N+](CC1)CCc3ccccc3)C	9.0
106	O=C(OC)C1(N(c2ccccc2)C(=O)CC)CC[N+](CC1)CCc3ccccc3	9.0
107	O=C(N([C@@]1(C[N+](CC1)CCc2ccccc2)C(C)C)c3ccccc3)CC	9.0
108	O=C(N(SCC[N+]CCc1ccccc1)c2ccccc2)CC	9.0
109	O=C(N([C@H]([N+]1C[C@@H]([C@@H](C1)CCc2ccccc2)C)C)c3ccccc3)CC	9.0
110	O=C(N([C@@]1(CO[C@@H](C[N+]1)CCc2ccccc2)C)c3ccccc3)CC	9.0
111	O=C(N([C@H]([N+]1CC[C@@H](C1)CCc2ccccc2)CC)c3ccccc3)CC	9.0
112	O=C(N([C@H]1CC[N+](C[C@H]1C([O-])=O)CCc2ccccc2)c3ccccc3)CC	9.0
113	O=C(NCCc1ccccc1)[C@H](CN(c2ccccc2)C(=O)CC)C[N+]	9.0
114	S=C(NCCc1ccccc1)CCN(c2ccccc2)C(=O)CC	9.0
115	O[C@@H]1[C@@H](O)[C@@H](O[C@H]([C@@H]1CCc2ccccc2)C[N+])N(c3ccccc3)C(=O)CC	9.0
116	O=C(N([C@@H]1C[C@H]([C@@H]([C@@H]([N+])C1)CCc2ccccc2)C)c3ccccc3)CC	9.0
117	O=C(N(C1(CC1)C[N+]CCCc2ccccc2)c3ccccc3)CC	9.0
118	O=C(N(C1([N+]CCCCc2ccccc2)CC1)c3ccccc3)CC	9.0
119	O=C(N(C[C@H]([N+]CC)CNCCc1ccccc1)c2ccccc2)CC	9.0
120	O=C(N([C@](N)(C[N+]CCCc1ccccc1)C)c2ccccc2)CC	9.0
121	O=C(NCCc1ccccc1)[C@H]([N+]N)CN(c2ccccc2)C(=O)CC	9.0
122	O=C(N([C@@]1(CC[N+][C@@H](CC1)CCc2ccccc2)C)c3ccccc3)CC	9.0
123	O=C(N(N1CC[N+][C@@H](CC1)CCc2ccccc2)c3ccccc3)CC	9.0
124	O=C(N([C@@H]([N+]([C@H](CCCc1ccccc1)C)C)C)c2ccccc2)CC	9.0
125	O=C(N(CC[C@@H](CCc1ccccc1)C#N)c2ccccc2)CC	9.0
126	O=C(N(C[N+](CC(OCCc1ccccc1)=O)C)c2ccccc2)CC	9.0
127	O=C(N(CC/C(NCCc1ccccc1)=[N+]\C)c2ccccc2)CC	9.0
128	O=C(N(C[N+]1CCC[C@H]1CCCc2ccccc2)c3ccccc3)CC	9.0
129	F[C@@H]1C[N+](C[C@H]1CCc2ccccc2)CN(c3ccccc3)C(=O)CC	9.0
130	O[C@@H](CCCc1ccccc1)CN(c2ccccc2)C(=O)CC	9.0
131	O=C(N(C([C@H]([N+]N)CCCc1ccccc1)(C)C)c2ccccc2)CC	9.0
132	O=C(N([C@H](C[N+](CCCc1ccccc1)C)C)c2ccccc2)CC	9.0
133	O=C(N([C@@H]([N+]CC)CC(OCCc1ccccc1)=O)c2ccccc2)CC	9.0
134	O=C(N([C@H](CN/C(NCCc1ccccc1)=[N+]\C)C)c2ccccc2)CC	9.0
135	O=C(N([C@@](C[N+]CCc1ccccc1)(CC)C)c2ccccc2)CC	9.0
136	O=C(N([N+]1([O-])CC[N+](CC1)CCc2ccccc2)c3ccccc3)CC	8.9
137	O=C(OC)[C@@H]1C[C@@H](N(c2ccccc2)C(=O)CC)CC[N+]1CCc3ccccc3	8.9
138	O=C(N([C@]1(C[N+](CC1)CCc2ccccc2)C#N)c3ccccc3)CC	8.9
139	O=C(N([C@]1(C[N+](CC1)CCc2ccccc2)CCC)c3ccccc3)CC	8.9
140	O=S(=O)(C[C@H]([N+])N(c1ccccc1)C(=O)CC)CCCc2ccccc2	8.9

141	O=C(N([C@@H]1CCC[N+](C[C@@H]1N)CCc2ccccc2)c3ccccc3)CC	8.9
142	O=C(N([C@H]1C[C@H](NCCc2ccccc2)C[N+]1C)c3ccccc3)CC	8.9
143	O[C@@H](N(c1ccccc1)C(=O)CC)C[N+]CCCc2ccccc2	8.9
144	O=C(N(OCC[N+](CCc1ccccc1)C)c2ccccc2)CC	8.9
145	O=C(N([C@@H](CC[N+])CC(=O)NCCc1ccccc1)c2ccccc2)CC	8.9
146	O[C@H]1[C@H](O)[C@@H](N(c2ccccc2)C(=O)CC)[C@H]([N+][C@H]1CCc3ccccc3)CC	8.9
147	O=C(N(C[N+]([C@H](CCCc1ccccc1)C)C)c2ccccc2)CC	8.9
148	O=C(N([C@@H]([N+]CC)CC(=O)NCCc1ccccc1)c2ccccc2)CC	8.9
149	O=C(N([C@@H]([N+]C)CC(OCCc1ccccc1)=O)c2ccccc2)CC	8.9
150	O[C@H]1C[N+](CC[C@H]([C@H]1N(c2ccccc2)C(=O)CC)C)CCc3ccccc3	8.9
151	O=C(N([C@H]1C[N+](C[C@H]1CCC)CCc2ccccc2)c3ccccc3)CC	8.9
152	ON(C1(N(c2ccccc2)C(=O)CC)CC[N+](CC1)CCc3ccccc3)C=O	8.9
153	O=C(N([C@@H]([N+](CCCCc1ccccc1)C)CC)c2ccccc2)CC	8.9
154	O=C(N(C1(CC[N+](CC1)CCc2ccccc2)C[N+]C3CC3)c4ccccc4)CC	8.9
155	O=C(N([C@@H]1CC=C(C[N+]1)CCc2ccccc2)c3ccccc3)CC	8.9
156	O=C(N([C@H]([N+]C/C=C/CCc1ccccc1)C)c2ccccc2)CC	8.9
157	O=C(N(CC[N+]([C@@H](CCc1ccccc1)C#N)C)c2ccccc2)CC	8.9
158	O=C(N(C[N+]1CC[C@@H]([C@H]1C)CCc2ccccc2)c3ccccc3)CC	8.9
159	O=C(N(C[C@@H]([C@@H]([N+])C(=O)NCCc1ccccc1)C)c2ccccc2)CC	8.9
160	O=C(N([C@H](CC[N+]CCc1ccccc1)C)c2ccccc2)CC	8.9
161	O=C(N(C1([N+]CCCc2ccccc2)CC1)c3ccccc3)CC	8.9
162	O=C(N([C@@H](N)C[N+]CCCc1ccccc1)c2ccccc2)CC	8.9
163	O=C(N([C@@]1(COCC[N+]1CCCCc2ccccc2)C)c3ccccc3)CC	8.9
164	O=C(N([C@H]1CN[C@H](C[N+]1CC)CCc2ccccc2)c3ccccc3)CC	8.9
165	O=C(N(C[N+]1C[C@H]([C@H](C1)CC)CCc2ccccc2)c3ccccc3)CC	8.9
166	O=C(N(C[C@H](NCC)C[N+]CCc1ccccc1)c2ccccc2)CC	8.9
167	O=C(N([C@@H]([N+](CCCc1ccccc1)CC)C)c2ccccc2)CC	8.9
168	O=C(NCCc1ccccc1)[C@@H]([N+][C@H](N(c2ccccc2)C(=O)CC)CC)C	8.9
169	O=C(N(OC[C@H]1[C@H]([N+]1CCc2ccccc2)C)c3ccccc3)CC	8.9
170	O=C(N(C1(CC[N+](CC1)CCc2ccccc2)CC(=O)NO)c3ccccc3)CC	8.9
171	O=C(N(C[N+](CCCCc1ccccc1)CCC)c2ccccc2)CC	8.9
172	O=C(N([C@]1(C[N+](CC1)CCc2ccccc2)c3nccs3)c4ccccc4)CC	8.9
173	O=C(N(N([C@H](C[N+]CCc1ccccc1)C)C)c2ccccc2)CC	8.9
174	S=C(N(c1ccccc1)C(=O)CC)[C@H]2C[N+](CCc3ccccc3)CCO2	8.9
175	O=C(N(N(CC[N+]CCc1ccccc1)CCC)c2ccccc2)CC	8.9
176	O=C(N([C@H]([N+])C/C=C/CCc1ccccc1)c2ccccc2)CC	8.9
177	O=C(N([C@]1(C[N+](CCO1)CCc2ccccc2)CC)c3ccccc3)CC	8.9
178	O=C(N([C@H]([N+]C[C@@H](CCc1ccccc1)c2ccco2)C)c3ccccc3)CC	8.9
179	O=S(=O)(N(c1ccccc1)C(=O)CC)CC[N+]CCc2ccccc2	8.9
180	O=C(N([C@@]1([C@H]([N+][C@@H](S1)CCc2ccccc2)C(=O)N)C)c3ccccc3)CC	8.9
181	O=C(N(C[N+](C(C)C)CCCCc1ccccc1)c2ccccc2)CC	8.9
182	O=C(N(C[N+][C@H](CCCc1ccccc1)C)c2ccccc2)CC	8.9

183	O=C(N(C[N+]1CCN([C@H]1C)CCc2ccccc2)c3ccccc3)CC	8.9
184	O=C(N([C@@H]([N+])CS[C@@H](CCc1ccccc1)C)c2ccccc2)CC	8.9
185	O=C(N(C1(OC)CC[N+](CC1)CCc2ccccc2)c3ccccc3)CC	8.8
186	F[C@@H](C[C@@H]([N+])N(c1ccccc1)C(=O)CC)CCc2ccccc2	8.8
187	O[C@]1(N(c2ccccc2)C(=O)CC)C[N+](C[C@@H]1O)CCc3ccccc3	8.8
188	O=C(N(C1(CC[N+](CC1)CCc2ccccc2)COC)c3ccccc3)CC	8.8
189	O=C(N(C1(CC[N+](CC1)CCc2ccccc2)CNC(N)=[N+])c3ccccc3)CC	8.8
190	O[C@@](N(c1ccccc1)C(=O)CC)(CC(=O)NCCc2ccccc2)C	8.8
191	O[C@H]([N+]CN(c1ccccc1)C(=O)CC)CCCc2ccccc2	8.8
192	O=C(N(C[N+]1CC[C@@H](C1)CCc2ccccc2)c3ccccc3)CC	8.8
193	O=C(N([C@@H]1CC[C@@H]([C@@H]([N+])C1)CCc2ccccc2)c3ccccc3)CC	8.8
194	O=C(N([C@@]1(N)C[N+](CC1)CCc2ccccc2)c3ccccc3)CC	8.8
195	O=C(N(C1(CC[N+](CC1)CCc2ccccc2)C[N+]CC)c3ccccc3)CC	8.8
196	O=C(N(SC[C@H]([N+]CCc1ccccc1)C)c2ccccc2)CC	8.8
197	O=C(N([C@@H]([N+])CC(OCCc1ccccc1)=O)c2ccccc2)CC	8.8
198	O=C(N(CC/C(=[N+]/CCc1ccccc1)N)c2ccccc2)CC	8.8
199	O=C(NCCc1ccccc1)[C@H]2[C@H](N(c3ccccc3)C(=O)CC)C[N+]2	8.8
200	O=C(N([C@@H]([N+]([C@H](CNCCc1ccccc1)C)C)C)c2ccccc2)CC	8.8
201	O=C(N(C1(OCCCc2ccccc2)C[N+]C1)c3ccccc3)CC	8.8
202	O=C(N(C[N+](CCCCc1ccccc1)CC)c2ccccc2)CC	8.8
203	O=C(N(C[C@H]([N+])NC(=O)CCc1ccccc1)c2ccccc2)CC	8.8
204	O=C(N([C@@H]1CC[N+][C@H]1CCCc2ccccc2)c3ccccc3)CC	8.8
205	O=C(N([C@H]([N+]1C[C@H](N)[C@@H](C1)CCc2ccccc2)C)c3ccccc3)CC	8.8
206	O=C(N([C@@]1(CCC[N+](CC1)CCc2ccccc2)C[N+])c3ccccc3)CC	8.8
207	O=C(N([C@H]([N+]CCNCCc1ccccc1)CC)c2ccccc2)CC	8.8
208	O[C@@]1(N(c2ccccc2)C(=O)CC)C[C@@H]([N+](C1)CCc3ccccc3)C([O-])=O	8.8
209	O=C(N(CC[N+][C@@H](CCc1ccccc1)C)c2ccccc2)CC	8.8
210	FC(F)(F)[C@H](N(c1ccccc1)C(=O)CC)CC(=O)NCCc2ccccc2	8.8
211	O=C(N(C([C@@H]([N+])CCCc1ccccc1)(C)C)c2ccccc2)CC	8.8
212	S/C(N(CN(c1ccccc1)C(=O)CC)C)=[N+]\CCc2ccccc2	8.8
213	O=C(N(C[N+]CC(OCCc1ccccc1)=O)c2ccccc2)CC	8.8
214	O=C(N([C@H](C[N+](CCc1ccccc1)C)COC)c2ccccc2)CC	8.8
215	O=C(N(C[N+](CCCCc1ccccc1)CC#C)c2ccccc2)CC	8.8
216	O=C(N(c1ccccc1)CS/C(NCCc2ccccc2)=N/C#N)CC	8.8
217	O=C(N(C1(CC1)C[N+]CCc2ccccc2)c3ccccc3)CC	8.8
218	O=C(N(S[C@H](C[N+]CCc1ccccc1)C)c2ccccc2)CC	8.8
219	O=C(N([C@]1(C2CCC2)C[N+](C[C@@H]1C)CCc3ccccc3)c4ccccc4)CC	8.8
220	O=C(N(N([C@@H](C[N+]CCc1ccccc1)CC)C)c2ccccc2)CC	8.8
221	O=C(N([C@@](N)(C1CC1)C[N+](CCc2ccccc2)C)c3ccccc3)CC	8.8
222	O=C(N(C[N+]1C[C@@H]([C@@H](C1)CCc2ccccc2)C)c3ccccc3)CC	8.8
223	O=C(N([C@@H]([N+]CCCCc1ccccc1)C)c2ccccc2)CC	8.8
224	O=C(N([C@@H]1CCCCC[C@H]1[N+](CCc2ccccc2)C)c3ccccc3)CC	8.8

225	FC1(N(c2ccccc2)C(=O)CC)CC[N+](CC1)CCc3ccccc3	8.7
226	O=C(N(C1(CC[N+](CC1)CCc2ccccc2)C)c3ccccc3)CC	8.7
227	O=C(N([C@@H]1CC[N+](C[C@@H]1N)CCc2ccccc2)c3ccccc3)CC	8.7
228	O=C(N([C@H]1CC[N+]([C@@H]([C@H]1C)C)CCc2ccccc2)c3ccccc3)CC	8.7
229	O=C(N(C1(CC[N+](CC1)CCc2ccccc2)C#N)c3ccccc3)CC	8.7
230	O=C(N([C@]1(C[N+](CC1)CCc2ccccc2)CC)c3ccccc3)CC	8.7
231	O=C(N([C@]1(CC[N+](C[C@H]1C)CCc2ccccc2)C)c3ccccc3)CC	8.7
232	O=C(N(CC[N+][C@@H](CCc1ccccc1)C#N)c2ccccc2)CC	8.7
233	FC1(F)C[N+](CC[C@@H]1N(c2ccccc2)C(=O)CC)CCc3ccccc3	8.7
234	O=C(N(N1C[C@H]([N+]([C@@H](C1)C)CCc2ccccc2)C)c3ccccc3)CC	8.7
235	O=C(N(S[C@@H]([N+]CCCc1ccccc1)N)c2ccccc2)CC	8.7
236	O=C(NCCc1ccccc1)[C@@H]([N+])[C@@H](N(c2ccccc2)C(=O)CC)CC	8.7
237	O=C(N([C@]1(CC[N+](C[C@H]1CC)CCc2ccccc2)C)c3ccccc3)CC	8.7
238	O=C(N([C@@H]1CO[C@@H](C[N+]1)CCc2ccccc2)c3ccccc3)CC	8.7
239	O=C(N(N(CC[N+]CCc1ccccc1)C)c2ccccc2)CC	8.7
240	O=C(N([C@H]1C[N+](C[C@H]1CC)CCc2ccccc2)c3ccccc3)CC	8.7
241	O=C(N([C@@H]1CCCC[N+]([C@H]1CC)CCc2ccccc2)c3ccccc3)CC	8.7
242	O[C@@H]1[C@@H](N(c2ccccc2)C(=O)CC)[C@H](O[C@H]([C@H]1[N+])CCc3ccccc3)O	8.7
243	O=C(NCCc1ccccc1)[C@@H]([N+])[C@@H](N(c2ccccc2)C(=O)CC)C(C)C	8.7
244	O=C(N(CC[N+](CCc1ccccc1)CC#C)c2ccccc2)CC	8.7
245	O=C(N(OC[C@@H]1C[N+]1CCc2ccccc2)c3ccccc3)CC	8.7
246	OC[C@@H]([N+]CCc1ccccc1)CCN(c2ccccc2)C(=O)CC	8.7
247	O=C(N(N(C[C@H]([N+]CCc1ccccc1)C)C)c2ccccc2)CC	8.7
248	O=C(N([C@]1(CC[N+](C[C@H]1CC(C)C)CCc2ccccc2)C)c3ccccc3)CC	8.7
249	O=C(N(N(CC[N+])CC(=O)NCCc1ccccc1)c2ccccc2)CC	8.7
250	O=C(N(C1(C[N+]C1)CCCCc2ccccc2)c3ccccc3)CC	8.7
251	O=C(N([C@]1(C2CC2)C[N+](C[C@@H]1C)CCc3ccccc3)c4ccccc4)CC	8.7
252	O=C(N(SC[C@@H]([N+]CCc1ccccc1)C#N)c2ccccc2)CC	8.7
253	O=C(N(C[N+](C1CC1)CCCCc2ccccc2)c3ccccc3)CC	8.7
254	O=C(N([C@]1(NCC(C)C)C[N+](CC1)CCc2ccccc2)c3ccccc3)CC	8.7
255	O=C(N(C(C[N+]CCc1ccccc1)(C)C)c2ccccc2)CC	8.7
256	O=C(N([C@@]1(C[N+](CC1)CCc2ccccc2)CCCCC)c3ccccc3)CC	8.7
257	O=C(N([C@@H]1CC[N+]1CCCCc2ccccc2)c3ccccc3)CC	8.7
258	O=C(N([C@@]1(CC[N+][C@H]1CCCc2ccccc2)C)c3ccccc3)CC	8.7
259	O=C(N)C1(N(c2ccccc2)C(=O)CC)CCC(CC1)CCc3ccccc3	8.7
260	O=C(N(SC/C(=[N+]/CCc1ccccc1)N)c2ccccc2)CC	8.7
261	O=C(N(C1(CC[N+](CC1)CCc2ccccc2)C[N+]C(C)C)c3ccccc3)CC	8.7
262	O=C(N([C@@H]1CC[C@H]([N+]1C)CCCc2ccccc2)c3ccccc3)CC	8.7
263	O=C(N(CCN[N+]CCc1ccccc1)c2ccccc2)CC	8.7
264	S[C@H]([C@@H]([N+])CN(c1ccccc1)C(=O)CC)C(=O)NCCc2ccccc2	8.7
265	O=C(N(N1CCC[N+]([C@@H](C(C)C)C1)CCc2ccccc2)c3ccccc3)CC	8.7
266	O=C(N(N([C@@H]1C[N+](CC1)CCc2ccccc2)CC)c3ccccc3)CC	8.7

267	O=C(OCC)[C@]1(N(c2ccccc2)C(=O)CC)C[N+](CC1)CCc3ccccc3	8.7
268	O=C(N([C@@]1(C[C@H]([N+](C[C@@H]1C)CCc2ccccc2)C)CC[N+])c3ccccc3)CC	8.7
269	O=C(N([C@@]1(C[C@H](O[C@@H]([N+]1)CCc2ccccc2)C)C)c3ccccc3)CC	8.7
270	O=C(N([C@@H](CC[N+](CCc1ccccc1)C)C)c2ccccc2)CC	8.7
271	O=C(N([C@H]([N+][C@H](CCCc1ccccc1)CC)C)c2ccccc2)CC	8.7
272	O=C(N(C[N+][C@@H](C(C)C)C(=O)NCCc1ccccc1)c2ccccc2)CC	8.7
273	O[C@@H]([C@@H]([N+])N(c1ccccc1)C(=O)CC)C(OCCc2ccccc2)=O	8.7
274	O=C(N(C[N+]1CCOC[C@H]1CCCc2ccccc2)c3ccccc3)CC	8.7
275	O=C(N(C[N+]1CCCC[C@H]1CNCCc2ccccc2)c3ccccc3)CC	8.7
276	O=C(N([C@H]1CC[N+]([C@@H](C1)C)CCc2ccccc2)c3ccccc3)CC	8.6
277	O=C(N([C@H]1CC[N+](C[C@H]1CC)CCc2ccccc2)c3ccccc3)CC	8.6
278	O=C(N(N1CCC[N+](CC1)CCc2ccccc2)c3ccccc3)CC	8.6
279	O[C@@]1(C[N+](CC[C@H]1N(c2ccccc2)C(=O)CC)CCc3ccccc3)C	8.6
280	O=C(OC)[C@@H]1C[N+](CC[C@@H]1N(c2ccccc2)C(=O)CC)CCc3ccccc3	8.6
281	O=C(N([C@H]1CC[N+]([C@@H](C1)c2ccco2)CCc3ccccc3)c4ccccc4)CC	8.6
282	O=C(N([C@@H](C[N+])CC(=O)NCCc1ccccc1)c2ccccc2)CC	8.6
283	O=C(N([C@H]1CC[N+]([C@@H](C1)CC(C)C)CCc2ccccc2)c3ccccc3)CC	8.6
284	O=C(NCCc1ccccc1)[C@@H]([N+])[C@@H](N(c2ccccc2)C(=O)CC)C	8.6
285	O=C(N([C@H]1CC[C@H](C[N+]1C)CCc2ccccc2)c3ccccc3)CC	8.6
286	O=C(N([C@H]1CC[C@H]([N+]C1)CCc2ccccc2)c3ccccc3)CC	8.6
287	O=C(N(C1([N+]CC(=O)NCCc2ccccc2)CC1)c3ccccc3)CC	8.6
288	OCCC[C@@H]1C[N+](CC[C@@H]1N(c2ccccc2)C(=O)CC)CCc3ccccc3	8.6
289	O=C(N(C[C@H]1C[N+](CC1)CCc2ccccc2)c3ccccc3)CC	8.6
290	O=C(N(N1CC[C@H]2[C@H]1CC[N+]2CCc3ccccc3)c4ccccc4)CC	8.6
291	O=C(N([C@@H]1CC=CC[N+](C1)CCc2ccccc2)c3ccccc3)CC	8.6
292	FC(F)(F)[C@H]1C[N+](C[C@H]1CCc2ccccc2)CN(c3ccccc3)C(=O)CC	8.6
293	O=C(OC)[C@]1(N(c2ccccc2)C(=O)CC)C[N+](CC1)CCc3ccccc3	8.6
294	O=C(N([C@@]1(C[C@H]([N+](C[C@@H]1C)CCc2ccccc2)CCC)C)c3ccccc3)CC	8.6
295	FC(F)(F)[C@]1(N(c2ccccc2)C(=O)CC)C[N+](CC1)CCc3ccccc3	8.6
296	FC(F)(F)C[N+]([C@@H](N(c1ccccc1)C(=O)CC)C)CCCc2ccccc2	8.6
297	OC[C@H]([N+]C/C=C/CCc1ccccc1)N(c2ccccc2)C(=O)CC	8.6
298	O=C(NO)[C@@H]1C[C@@H](N(c2ccccc2)C(=O)CC)C[N+]([C@H]1CCc3ccccc3)C	8.6
299	OC[C@H](CCc1ccccc1)C[N+](CN(c2ccccc2)C(=O)CC)C	8.6
300	O=C(N([C@H]([N+](CCCc1ccccc1)CC(C)C)C)c2ccccc2)CC	8.6
301	F[C@]1(N(c2ccccc2)C(=O)CC)C[N+](CC1(F)F)CCc3ccccc3	8.6
302	O=C(N(C1([N+]CC2CC2)CC[N+](CC1)CCc3ccccc3)c4ccccc4)CC	8.6
303	O=C(NCCc1ccccc1)[C@H]([N+]C(N(c2ccccc2)C(=O)CC)(C)C)C	8.6
304	O=C(NCCc1ccccc1)[C@H]2[C@H]([N+]2)N(c3ccccc3)C(=O)CC	8.6
305	O=C(N(C([N+]CCCc1ccccc1)(C)C)c2ccccc2)CC	8.6
306	O=C(N([C@H]1CN[C@@H](C[N+]1C(C)C)CCc2ccccc2)c3ccccc3)CC	8.6
307	O=C(N([C@]1(C[C@H](C[N+](CC1)C)CCc2ccccc2)C)c3ccccc3)CC	8.6
308	O=C(N([C@@H]([N+]CC(=O)NCCc1ccccc1)C2CC2)c3ccccc3)CC	8.6

309	O=C(N([C@@]1(CC=CC[N+](C1)CCc2ccccc2)C)c3ccccc3)CC	8.6
310	O=C(N(C[C@H](N)C[N+]CCc1ccccc1)c2ccccc2)CC	8.6
311	O=C(N(C[N+](CC(=O)CCCc1ccccc1)C)c2ccccc2)CC	8.6
312	OC[C@@]1(N(c2ccccc2)C(=O)CC)CO[C@@H](C[N+]1)CCc3ccccc3	8.6
313	O=C(N(N(CC[N+](CCc1ccccc1)C)C)c2ccccc2)CC	8.6
314	O=C(N(C[N+]([C@@H](CCCc1ccccc1)C)CC)c2ccccc2)CC	8.6
315	O=C(N([C@@H]1C[N+]1CC(OCCc2ccccc2)=O)c3ccccc3)CC	8.6
316	O=C(N([C@]([N+])(CCCCc1ccccc1)C)c2ccccc2)CC	8.6
317	O=C(NN)[C@@H]([N+]CCc1ccccc1)CCN(c2ccccc2)C(=O)CC	8.6
318	O=C(N([C@@H]1CN([C@@H](C[N+]1)CCc2ccccc2)C(=O)COC)c3ccccc3)CC	8.6
319	O=C(N([C@@]([N+]C)(CCCCc1ccccc1)C)c2ccccc2)CC	8.6
320	O[C@H](ON(c1ccccc1)C(=O)CC)C[N+]CCc2ccccc2	8.6
321	O=C(N(C[N+](CCCCc1ccccc1)CC(=O)N)c2ccccc2)CC	8.6
322	O=C(N([C@H]1C[N+](C[C@H]1CSC)CCc2ccccc2)c3ccccc3)CC	8.6
323	O=C(N(C[C@H]([N+])[C@@H](CCc1ccccc1)C)c2ccccc2)CC	8.6
324	O=C(N([C@H]([N+])CSCCCc1ccccc1)c2ccccc2)CC	8.6
325	O=C(N(C[N+]1CCS[C@@H](C1)CCc2ccccc2)c3ccccc3)CC	8.6
326	O=C(N(C[N+]1CCC[C@@H]1NCCCc2ccccc2)c3ccccc3)CC	8.6
327	O=C(N([C@]1(C(C)C)C[N+](C[C@@H]1C)CCc2ccccc2)c3ccccc3)CC	8.6
328	O=C(N(C[C@H]1[N+][C@@H](CCc2ccccc2)CS1)c3ccccc3)CC	8.6
329	OC[C@H]1[C@@H](N(c2ccccc2)C(=O)CC)C[C@H]([N+])[C@H](O1)CCc3ccccc3	8.6
330	O=C(N([C@H]1CC[N+](C[C@H]1C)CCc2ccccc2)c3ccccc3)CC	8.5
331	O=C(N([C@@H]([N+])CC(=O)NCCc1ccccc1)c2ccccc2)CC	8.5
332	O=C(N([C@H](C[C@]([N+]CCc1ccccc1)(C)C#N)C)c2ccccc2)CC	8.5
333	O=C(N(N1C[C@H]([N+](C[C@@H]1C)CCc2ccccc2)C)c3ccccc3)CC	8.5
334	O=C(N([C@]1(C[N+](C[C@H]1C)CCc2ccccc2)C)c3ccccc3)CC	8.5
335	O=C(N([C@]1(NCC)C[N+](CC1)CCc2ccccc2)c3ccccc3)CC	8.5
336	F[C@]1(N(c2ccccc2)C(=O)CC)C[C@@H]([N+](C1)CCc3ccccc3)CO	8.5
337	O=C(N([C@H]1CC[C@H](C[N+]1)CCc2ccccc2)c3ccccc3)CC	8.5
338	O[C@@](N(c1ccccc1)C(=O)CC)(C[N+]CCCc2ccccc2)C	8.5
339	F[C@@]1(N(c2ccccc2)C(=O)CC)CC[N+][C@H](C1)CCc3ccccc3	8.5
340	O=C(N(N1CCC[N+](C[C@H]1C)CCc2ccccc2)c3ccccc3)CC	8.5
341	O[C@@H]1[C@@H](OC[C@H](N(c2ccccc2)C(=O)CC)[C@H]1[N+])CCc3ccccc3	8.5
342	O=C(N(N(C[C@]([N+]CCc1ccccc1)(C)C#N)C)c2ccccc2)CC	8.5
343	O=C(N([C@]1(NC2CC2)C[N+](CC1)CCc3ccccc3)c4ccccc4)CC	8.5
344	O=C(NCCc1ccccc1)[C@@H]([N+]CN(c2ccccc2)C(=O)CC)C	8.5
345	O=C(NCCc1ccccc1)[C@@H]([N+])CN(c2ccccc2)C(=O)CC	8.5
346	O=C(N(N1C[C@H]([N+](C[C@@H]1CC)CCc2ccccc2)CC)c3ccccc3)CC	8.5
347	O=C(NCCc1ccccc1)[C@H]([N+])N(c2ccccc2)C(=O)CC	8.5
348	O=C(N(C[N+]CC(=O)CCCc1ccccc1)c2ccccc2)CC	8.5
349	O=C(N(C1(CC[N+](CC1)CCc2ccccc2)CCOC)c3ccccc3)CC	8.5
350	O=C(NCCc1ccccc1)[C@@]([N+])(N(c2ccccc2)C(=O)CC)C	8.5

351	O=C(N([C@@H]1C[C@H]([N+](C1)CCc2ccccc2)COC)c3ccccc3)CC	8.5
352	O[C@H]1C[N+](C[C@@H]1ON(c2ccccc2)C(=O)CC)CCc3ccccc3	8.5
353	O=C(N(OC[C@]([N+]CCc1ccccc1)(C)C#N)c2ccccc2)CC	8.5
354	O=C(N(NCC[N+]CCc1ccccc1)c2ccccc2)CC	8.5
355	O=C(N(N([C@@H]1C[N+](CC1)CCc2ccccc2)C)c3ccccc3)CC	8.5
356	OC[C@@H]([N+]CCN(c1ccccc1)C(=O)CC)CCc2ccccc2	8.5
357	O=C(N([C@H]1CCCC[N+]([C@@H]1C2CC2)CCc3ccccc3)c4ccccc4)CC	8.5
358	O=C(N(C[N+](C[C@H](OC)CCc1ccccc1)C)c2ccccc2)CC	8.5
359	O[C@@H]1[C@@H](N(c2ccccc2)C(=O)CC)[C@@H]([N+]([C@H]1CC)CCc3ccccc3)CO	8.5
360	O=C(N(C[C@@H]1CCC[N+](C1)CCc2ccccc2)c3ccccc3)CC	8.5
361	O=C(N(C[C@H](C[N+]CCc1ccccc1)C)c2ccccc2)CC	8.5
362	O=C(N([C@@H](C[N+](CCc1ccccc1)C)C)c2ccccc2)CC	8.5
363	O=C(NCCc1ccccc1)[C@@H]([N+]CC)CN(c2ccccc2)C(=O)CC	8.5
364	O=C(N1C[C@H]([N+]C[C@H]1CCc2ccccc2)N(c3ccccc3)C(=O)CC)N(C)C	8.5
365	O=C(N(C[N+](C[C@H](OCC)CCc1ccccc1)C)c2ccccc2)CC	8.5
366	O=C(N([C@H](C[N+]CCc1ccccc1)C)c2ccccc2)CC	8.5
367	O=C(N(C[C@H](N)C[N+](CCc1ccccc1)C)c2ccccc2)CC	8.5
368	O[C@H]([C@@H]([N+])CN(c1ccccc1)C(=O)CC)C(=O)NCCc2ccccc2	8.5
369	O=C(N([C@](N)(C[N+]CCc1ccccc1)C)c2ccccc2)CC	8.5
370	O=C(NCCc1ccccc1)[C@@H]([N+]C)CN(c2ccccc2)C(=O)CC	8.5
371	O=C(N(C[C@@H]1C[C@H]([N+](C1)CCc2ccccc2)C)c3ccccc3)CC	8.5
372	O=C(N([C@]1(CNC[C@@H]([N+]1C)CCc2ccccc2)C)c3ccccc3)CC	8.5
373	O=C(N(C[N+]1CCCN[C@H](C1)CCc2ccccc2)c3ccccc3)CC	8.5
374	O=C(N([C@@H]1CC[N+](C[C@@H]1C=C)CCc2ccccc2)c3ccccc3)CC	8.4
375	O=C(N([N+]1(CC[N+](CC1)CCc2ccccc2)C)c3ccccc3)CC	8.4
376	O=C(N([C@@H]1CC[N+](C[C@@H]1N(C)C)CCc2ccccc2)c3ccccc3)CC	8.4
377	O=C(N(CC[C@]([N+]CCc1ccccc1)(C)C#N)c2ccccc2)CC	8.4
378	O=C(N(C[N+]CC(=O)NCCc1ccccc1)c2ccccc2)CC	8.4
379	O=C(NCCc1ccccc1)[C@H]([N+])CCN(c2ccccc2)C(=O)CC	8.4
380	O[C@H]1C[C@H]([N+]C[C@H]1N(c2ccccc2)C(=O)CC)CCc3ccccc3	8.4
381	O=C(N([C@H]1C[N+]1CCCCc2ccccc2)c3ccccc3)CC	8.4
382	F[C@@]1(N(c2ccccc2)C(=O)CC)C[C@H]([N+](C1)CCc3ccccc3)COC	8.4
383	O=C(N([C@H]1C[C@@H]([C@H](C[C@@H]1[N+])CCc2ccccc2)C[N+])c3ccccc3)CC	8.4
384	O[C@@H](C[N+]CCc1ccccc1)CN(c2ccccc2)C(=O)CC	8.4
385	O[C@]1(N(c2ccccc2)C(=O)CC)CC[N+]([C@@H]3CCCC[C@@H]13)CCc4ccccc4	8.4
386	O=C(N(C[C@@H](OC)C[N+]CCc1ccccc1)c2ccccc2)CC	8.4
387	O=C(N([C@@H]1C[C@H]([N+](C1)CCc2ccccc2)CC(C)C)c3ccccc3)CC	8.4
388	O=C(N(C[C@H](ONCCc1ccccc1)[N+])c2ccccc2)CC	8.4
389	O=C(N(OC[C@@H]([N+]CCc1ccccc1)C#N)c2ccccc2)CC	8.4
390	SC[C@H]([N+]CN(c1ccccc1)C(=O)CC)C(=O)NCCc2ccccc2	8.4
391	O=C(NCCc1ccccc1)[C@@H]([N+]CN(c2ccccc2)C(=O)CC)CC	8.4
392	OC[C@H](N(c1ccccc1)C(=O)CC)C[N+]CCCc2ccccc2	8.4

393	O=C(N(C[N+](N)CCCc1ccccc1)c2ccccc2)CC	8.4
394	O[C@@H](N(c1ccccc1)C(=O)CC)[C@@H]([N+]CCc2ccccc2)CO	8.4
395	O=C(N([C@H]1CCC[N+]1[C@@H](CCc2ccccc2)C(=O)N)c3ccccc3)CC	8.4
396	O[C@@H]1[C@@H](O)[C@@H]([N+]([C@H]1CCCc2ccccc2)C)N(c3ccccc3)C(=O)CC	8.4
397	O=C(N(C[N+](CCCc1ccccc1)CCC)c2ccccc2)CC	8.4
398	O=C(N([C@H]1CNC[C@@H]1[N+](CCc2ccccc2)CC=C)c3ccccc3)CC	8.4
399	O=C(N(C[N+](C1CNC1)CCCc2ccccc2)c3ccccc3)CC	8.4
400	O=C(N(C[N+]CCCc1ccccc1)c2ccccc2)CC	8.4
401	O=C(N([C@@]1(C[N+][C@@H](C1)CCc2ccccc2)C)c3ccccc3)CC	8.4
402	O=C(N([C@H]1CN[C@H](C[N+]1CC=C)CCc2ccccc2)c3ccccc3)CC	8.4
403	OC[C@]([N+]CCCc1ccccc1)(N(c2ccccc2)C(=O)CC)C	8.4
404	O=C(NCCc1ccccc1)C([N+]CN(c2ccccc2)C(=O)CC)(C)C	8.4
405	O[C@H]1C[N+](CC[C@H]1N(c2ccccc2)C(=O)CC)CCc3ccccc3	8.3
406	F[C@]1(N(c2ccccc2)C(=O)CC)C[N+](CC1)CCc3ccccc3	8.3
407	O=C(N([C@H]1CC[N+]([C@@H]1CC)CCc2ccccc2)c3ccccc3)CC	8.3
408	O=C(N([C@H]1CC[N+]([C@@H]1C2CC2)CCc3ccccc3)c4ccccc4)CC	8.3
409	O=C(N(C1([N+]C)CC[N+](CC1)CCc2ccccc2)c3ccccc3)CC	8.3
410	O[C@H]1C[N+](CCC[C@H]1N(c2ccccc2)C(=O)CC)CCc3ccccc3	8.3
411	O=C(N([C@](N)(C[N+](CCc1ccccc1)C)C)c2ccccc2)CC	8.3
412	O=C(N([C@@H]1CC[N+]([C@H]2CCO[C@H]12)CCc3ccccc3)c4ccccc4)CC	8.3
413	O=C(N([C@@H]1CC=C(C[N+]1C)CCc2ccccc2)c3ccccc3)CC	8.3
414	O[C@@H]1C[N+](C[C@@H]1N(c2ccccc2)C(=O)CC)CCc3ccccc3	8.3
415	O=C(N(C[N+](CCCc1ccccc1)CC)c2ccccc2)CC	8.3
416	O=C(NCCc1ccccc1)[C@H]([N+])CSN(c2ccccc2)C(=O)CC	8.3
417	O[C@H]([C@H]([N+])N(c1ccccc1)C(=O)CC)C(=O)CCc2ccccc2	8.3
418	O[C@@]1(N(c2ccccc2)C(=O)CC)C[N+](C[C@H]1O)CCCc3ccccc3	8.3
419	O=C(N(S[C@@H]1C[N+](CC1)CCc2ccccc2)c3ccccc3)CC	8.3
420	O=C(NO)[C@H]1C[N+](CC[C@H]1N(c2ccccc2)C(=O)CC)CCc3ccccc3	8.3
421	O=C(N([C@H]1CN[C@H](C[N+]1C)CCc2ccccc2)c3ccccc3)CC	8.3
422	O=C(N(C[C@H]([N+](CCc1ccccc1)C)C2CC2)c3ccccc3)CC	8.3
423	O=C(N([C@@H]1CN([N+](C1)CCc2ccccc2)CC)c3ccccc3)CC	8.3
424	O=C(N([C@@H]1C[C@H]([N+](C1)CCc2ccccc2)CCC)c3ccccc3)CC	8.3
425	O=C(N([C@H](C[N+](CCc1ccccc1)C)CC[N+])c2ccccc2)CC	8.3
426	OC[C@H]1[C@@H](N(c2ccccc2)C(=O)CC)CC[C@H]([N+]1)CCc3ccccc3	8.3
427	O=C(N([C@H]1C[N+]([C@H](C1)CCCC)CCc2ccccc2)c3ccccc3)CC	8.3
428	O[C@H]1[C@@H](O[C@H]([C@H](N(c2ccccc2)C(=O)CC)[C@H]1[N+])C[N+])CCc3ccccc3	8.3
429	O=C(N(CC[N+]CCc1ccccc1)c2ccccc2)CC	8.3
430	O=C(N(SC[C@]([N+]CCc1ccccc1)(C2CC2)C#N)c3ccccc3)CC	8.3
431	O=C(N([C@]1([N+])C[C@H]([N+]([C@H](C1)CCc2ccccc2)C)C)c3ccccc3)CC	8.3
432	O=C(N(CC[C@@H]([N+]CCc1ccccc1)C)c2ccccc2)CC	8.3
433	O=C(N(C[C@H]1C[N+]C[C@@H]1NCCc2ccccc2)c3ccccc3)CC	8.3
434	O=C(N([C@@H]([N+](CCCC)CCCc1ccccc1)C)c2ccccc2)CC	8.3

435	OC[C@@H]1[C@H](CC[N+]1CCc2ccccc2)CN(c3ccccc3)C(=O)CC	8.3
436	O=C(N([C@H](C[N+](CCc1ccccc1)C)C#N)c2ccccc2)CC	8.3
437	O=C(N(C[C@@H]([N+](CCc1ccccc1)C)C)c2ccccc2)CC	8.2
438	O[C@]1(N(c2ccccc2)C(=O)CC)CC[N+](C[C@@H]1O)CCc3ccccc3	8.2
439	O=C(N([C@@H]1CC[N+](C[C@@H]1OC)CCc2ccccc2)c3ccccc3)CC	8.2
440	O=C(N(CC[C@@H]([N+]CCc1ccccc1)C#N)c2ccccc2)CC	8.2
441	O=C(N(C1(CC[N+](CC1)CCc2ccccc2)C[N+])c3ccccc3)CC	8.2
442	OC[C@@]1(N(c2ccccc2)C(=O)CC)C[N+](CC1)CCc3ccccc3	8.2
443	O=C1C[N+](C[C@H]1N(c2ccccc2)C(=O)CC)CCc3ccccc3	8.2
444	O=C(N(C[N+][C@@H](CCCc1ccccc1)C#N)c2ccccc2)CC	8.2
445	F[C@H]1C[N+](C[C@H]1ON(c2ccccc2)C(=O)CC)CCc3ccccc3	8.2
446	O=C(OCC)[C@H]1C[C@@H](N(c2ccccc2)C(=O)CC)C[N+]1CCc3ccccc3	8.2
447	O=C(N(C[N+][C@@H](CCc1ccccc1)C#N)c2ccccc2)CC	8.2
448	FC(F)(F)[C@@H]1C[N+](C[C@@H]1N(c2ccccc2)C(=O)CC)CCc3ccccc3	8.2
449	O=C(N([C@H]1CC[N+](C[C@H]1n2cncc2)CCc3ccccc3)c4ccccc4)CC	8.2
450	O=C(NC)[C@H]1CN(N(c2ccccc2)C(=O)CC)CC[N+](C1)CCc3ccccc3	8.2
451	O=C(NC)[C@]1(N(c2ccccc2)C(=O)CC)C[N+](CC1)CCc3ccccc3	8.2
452	O=C(N([C@H]1C[N+](C[C@H]1OC(=O)C)CCc2ccccc2)c3ccccc3)CC	8.2
453	O=C(N([C@H]1C[N+]([C@@H](C1)C(=O)N)CCc2ccccc2)c3ccccc3)CC	8.2
454	OC[C@@H]1[C@H](N(c2ccccc2)C(=O)CC)[C@@H](N)[C@@H]([N+])[C@@H](O1)CCc3ccccc3	8.2
455	O=C(N(C1([N+](C)C)CC[N+](CC1)CCc2ccccc2)c3ccccc3)CC	8.2
456	O[C@@H]1[C@H](N(c2ccccc2)C(=O)CC)[C@@H]([N+][C@@H]([C@H]1C)CCc3ccccc3)CO	8.2
457	O=C(N([C@]1(COCC[N+]1CCCc2ccccc2)C)c3ccccc3)CC	8.2
458	O=C(N([C@@H]1C[N+]([C@@H](C1)CCc2ccccc2)C)c3ccccc3)CC	8.2
459	O=C(N([C@]1(CCCC[N+](C1)CCc2ccccc2)C[N+])c3ccccc3)CC	8.2
460	F[C@H]1C[N+](CC[C@H]1N(c2ccccc2)C(=O)CC)CCc3ccccc3	8.1
461	O=C(N([C@@H]([N+])CC[N+]CCc1ccccc1)c2ccccc2)CC	8.1
462	O=C(N([C@H](N)[C@@H]([N+](CCc1ccccc1)C)C)c2ccccc2)CC	8.1
463	O=C(N(CC[N+](CCc1ccccc1)C)c2ccccc2)CC	8.1
464	O=C(N([C@@]1(C[N+](CC1)CCc2ccccc2)CC[N+])c3ccccc3)CC	8.1
465	O[C@@H]1C[N+]([C@@H]([C@H]1N(c2ccccc2)C(=O)CC)CO)CCc3ccccc3	8.1
466	O=C(NO)[C@H]1C[C@H](N(c2ccccc2)C(=O)CC)C[N+][C@@H]1CCc3ccccc3	8.1
467	O=C(N(C1([N+])C[C@@H]([N+]([C@@H](C1)C)CCc2ccccc2)C)c3ccccc3)CC	8.1
468	O=C(N([C@H]1C[N+](C[C@H]1C#N)CCc2ccccc2)c3ccccc3)CC	8.1
469	O[C@@H]1[C@@H](N(c2ccccc2)C(=O)CC)C[N+][C@H]1CCc3ccccc3	8.1
470	S=C(N)C1(N(c2ccccc2)C(=O)CC)CC[N+](CC1)CCc3ccccc3	8.1
471	O[C@@H]([C@H]([N+])N(c1ccccc1)C(=O)CC)CCCc2ccccc2	8.1
472	O=C(N(N1CC[N+]([C@@H]1c2ccco2)CCc3ccccc3)c4ccccc4)CC	8.1
473	O=C(N([C@@H]1CC[N+]([C@H]1CC(C)(C)C)CCc2ccccc2)c3ccccc3)CC	8.1
474	O=C(N)[C@]1(N(c2ccccc2)C(=O)CC)C[N+](CC1)CCc3ccccc3	8.1

475	O=C(N([C@H]([N+]CCCc1ccccc1)CN)c2ccccc2)CC	8.1
476	O[C@@H](N(c1ccccc1)C(=O)CC)[C@@H]([N+])CCCc2ccccc2	8.1
477	O=C(N(C[N+](CCCc1ccccc1)CCCC#N)c2ccccc2)CC	8.1
478	O=C(N(C[N+]([C@H](CCCc1ccccc1)C(=O)N)C)c2ccccc2)CC	8.1
479	O=C(NO)[C@@H]1C[N+](C[C@@H]1N(c2ccccc2)C(=O)CC)CCc3ccccc3	8.1
480	O[C@@]1(N(c2ccccc2)C(=O)CC)[C@@H](O[C@@H](C[C@@H]1[N+])CCc3ccccc3)C	8.1
481	OCC1(N(c2ccccc2)C(=O)CC)CC[N+](CC1)CCc3ccccc3	8.0
482	O=C(N([C@]1(NC)C[N+](CC1)CCc2ccccc2)c3ccccc3)CC	8.0
483	O=C(N([C@@H]1C[N+](C[C@@H]1N)CCc2ccccc2)c3ccccc3)CC	8.0
484	O=C(N([C@H]1C[N+]([C@H](C1)c2nc(no2)C)CCc3ccccc3)c4ccccc4)CC	8.0
485	O[C@H](N(c1ccccc1)C(=O)CC)C[N+]CCc2ccccc2	8.0
486	O[C@@H](N(c1ccccc1)C(=O)CC)C[C@@H]([N+]CCc2ccccc2)C	8.0
487	O[C@H]([C@@H]([N+])N(c1ccccc1)C(=O)CC)C(=O)NCCc2ccccc2	8.0
488	O[C@@H]([N+]CCCc1ccccc1)N(c2ccccc2)C(=O)CC	8.0
489	O=C(N([C@@H]1C[N+]([C@@H](O1)CCc2ccccc2)CC)c3ccccc3)CC	8.0
490	O[C@]1(N(c2ccccc2)C(=O)CC)C[N+](CC1)CCc3ccccc3	7.9
491	O=S(=O)(NCCc1ccccc1)C[C@@H]([N+])N(c2ccccc2)C(=O)CC	7.9
492	O=C(N([C@@]1(C[N+](CC1)CCc2ccccc2)C[N+])c3ccccc3)CC	7.9
493	O=C(N([C@H](N)C[N+](CCc1ccccc1)C)c2ccccc2)CC	7.9
494	O[C@H]1[C@H](O)[C@H](N(c2ccccc2)C(=O)CC)C[N+][C@@H]1CCc3ccccc3	7.9
495	O=C(N([C@H]1[C@H](O[C@H]([C@H]2[C@@H]1[N+]2)CCc3ccccc3)C[N+])c4ccccc4)CC	7.8
496	O=C(N([C@@H]1CC[N+]([C@H]1c2cnccc2)CCc3ccccc3)c4ccccc4)CC	7.8
497	O[C@H]1C[S@+](N(c2ccccc2)C(=O)CC)[C@H]([C@@H]1CCc3ccccc3)CO	7.7
498	O=C(N([C@H]1CC[N+]([C@@H]1CC[N+])CCc2ccccc2)c3ccccc3)CC	7.6
499	O[C@@H]1C[N+](C[C@H](O)C1N(c2ccccc2)C(=O)CC)CCc3ccccc3	7.6
500	O=C(N([C@@H]([N+](CCCc1ccccc1)C)CC#N)c2ccccc2)CC	7.6

Table S8

List, SMILE and predicted pK_i values for Series 6.

N°	SMILES	Pred pK_i
1	O=C(N(C1CC[N+](CC1)C[C@@H](c2ccccc2)CC#C)c3ccccc3)CC	9.5
2	O=C(N(C1CC[N+]([C@@H]2[C@](O2)(c3ccccc3)C)CC1)c4ccccc4)CC	9.4
3	O=C(N(C1CC[N+](C[C@H]2c3ccccc3CCS2)CC1)c4ccccc4)CC	9.4
4	O=C(N(C1CC[N+](C[C@H](NC)c2ccccc2)CC1)c3ccccc3)CC	9.3
5	O=C(N(C1CC[N+](C[C@H](C(C)C)c2ccccc2)CC1)c3ccccc3)CC	9.3
6	O=C(N(C1CC[N+](C[C@H](N(C)C)c2ccccc2)CC1)c3ccccc3)CC	9.3
7	O=C(N(C1CC[N+]([C@H](Cc2ccccc2)CC#C)CC1)c3ccccc3)CC	9.3
8	O=C(N(C1CC[N+](CC1)CCc2ccccc2C#N)c3ccccc3)CC	9.3
9	O=C(N(C1CC[N+](CC1)CCc2c(O)ccc(c2)C)c3ccccc3)CC	9.2
10	O=C(N(C1CC[N+](CC(C)(C)c2cccc(c2)C)CC1)c3ccccc3)CC	9.2
11	O=C(N(C1CC[N+](C[C@H]2c3ccccc3CCCC2)CC1)c4ccccc4)CC	9.2

12	O=C(N(C1CC[N+]([C@H]2C[C@@]2(c3ccccc3)C)CC1)c4ccccc4)CC	9.2
13	O=C(N(C1CC[N+]([C@H]2C[C@@H]3c4ccccc4[C@H]2O3)CC1)c5ccccc5)CC	9.2
14	O=C(N(C1CC[N+](CC1)C[C@](CC)(c2ccccc2)C)c3ccccc3)CC	9.2
15	O=C(N(C1CC[N+](CC1)C[C@@H](c2ccccc2)C)c3ccccc3)CC	9.1
16	O=C(N(C1CC[N+](CC1)[C@H](Sc2ccccc2)C)c3ccccc3)CC	9.1
17	O=C(N(C1CC[N+](C[C@H]2c3ccccc3CCN2)CC1)c4ccccc4)CC	9.1
18	O=C(N(C1CC[N+](CC1)[C@H](Sc2ccccc2)CC)c3ccccc3)CC	9.1
19	O=C(N(C1CC[N+]([C@@H](N)Cc2ccccc2)CC1)c3ccccc3)CC	9.1
20	O=C(N(C1CC[N+](CC1)CCc2ccccc2O)c3ccccc3)CC	9.0
21	O=C(N(C1CC[N+](CC1)CCc2ccccc2CC)c3ccccc3)CC	9.0
22	Cl[C@@H](C[N+]1CCC(N(c2ccccc2)C(=O)CC)CC1)c3ccccc3	9.0
23	Oc1cc(ccc1CC[N+]2CCC(N(C(=O)CC)c3ccccc3)CC2)C	9.0
24	O=C(N(C1CC[N+](CC1)CSc2c(N)cccn2)c3ccccc3)CC	9.0
25	O=C(N(C1CC[N+]([C@@H]2[C@@](C2)(C)c3cccs3)CC1)c4ccccc4)CC	9.0
26	Fc1cc(N)ccc1SC[N+]2CCC(N(C(=O)CC)c3ccccc3)CC2	9.0
27	O=C(N(C1CC[N+](C[C@@H](c2ccccc2OC)C)CC1)c3ccccc3)CC	9.0
28	FC(F)(F)c1ccccc1CC[N+]2CCC(N(c3ccccc3)C(=O)CC)CC2	9.0
29	Clc1ccccc1[C@H](C[N+]2CCC(N(C(=O)CC)c3ccccc3)CC2)C	9.0
30	Clc1cc(N)ccc1SC[N+]2CCC(N(C(=O)CC)c3ccccc3)CC2	9.0
31	O=C(N(C1CC[N+](CC1)CSc2c[nH]c3ccccc32)c4ccccc4)CC	9.0
32	F[C@@H](C[N+]1CCC(N(c2ccccc2)C(=O)CC)CC1)c3ccccc3	8.9
33	O=C(N(C1CC[N+](CC1)CC(c2ccccc2)=C)c3ccccc3)CC	8.9
34	Fc1ccccc1SC[N+]2CCC(N(C(=O)CC)c3ccccc3)CC2	8.9
35	O=C(N(C1CC[N+](CC1)C[C@@H](c2ccccc2)C=C)c3ccccc3)CC	8.9
36	O=C(N(C1CC[N+](CC1)CSc2cccc(N)c2)c3ccccc3)CC	8.9
37	O=C(N(C1CC[N+](CC1)CSc2ccc(N)cc2)c3ccccc3)CC	8.9
38	F[C@](C[N+]1CCC(N(c2ccccc2)C(=O)CC)CC1)(c3ccccc3)C	8.9
39	Fc1cc(N)ccc1CC[N+]2CCC(N(C(=O)CC)c3ccccc3)CC2	8.9
40	Fc1ccc(CC[N+]2CCC(N(C(=O)CC)c3ccccc3)CC2)c(O)c1	8.9
41	O=C(N(C1CC[N+]([C@H](Cc2ccccc2C)C)CC1)c3ccccc3)CC	8.9
42	O=C(N(C1CC[N+](CC1)CCc2ccc(N)cc2C)c3ccccc3)CC	8.9
43	Fc1ccccc1[S@@](=O)C[N+]2CCC(N(C(=O)CC)c3ccccc3)CC2	8.9
44	O=C(N(C1CC[N+](CC1)CCc2ccccc2OC(=O)C)c3ccccc3)CC	8.9
45	O=C(N(C1CC[N+](CC1)CCc2ccc(C)cn2)c3ccccc3)CC	8.9
46	O=C(N(C1CC[N+](CC1)C[C@H](N)c2cccs2)c3ccccc3)CC	8.9
47	Clc1cc(N)ccc1CC[N+]2CCC(N(C(=O)CC)c3ccccc3)CC2	8.9
48	Clc1ccccc1S[C@@H]([N+]2CCC(N(C(=O)CC)c3ccccc3)CC2)C	8.9
49	Sc1cc(N)ccc1CC[N+]2CCC(N(C(=O)CC)c3ccccc3)CC2	8.9
50	Sc1ccc(N)cc1CC[N+]2CCC(N(C(=O)CC)c3ccccc3)CC2	8.9
51	O=C(N(C1CC[N+]([C@@H](N)Cc2ccccc2C)CC1)c3ccccc3)CC	8.9
52	Oc1cccc2CCN[C@@H](C[N+]3CCC(N(C(=O)CC)c4ccccc4)CC3)c12	8.9
53	Clc1ccccc1SC[N+]2CCC(N(C(=O)CC)c3ccccc3)CC2	8.8

54	O=[S@@](C[N+]1CCC(N(C(=O)CC)c2ccccc2)CC1)c3cccs3	8.8
55	FC(F)(C[N+]1CCC(N(c2ccccc2)C(=O)CC)CC1)c3ccccc3	8.8
56	O=C(N(C1CC[N+](CC1)CCc2ccc(N)cc2)c3ccccc3)CC	8.8
57	O=C(N(C1CC[N+](CC1)CSc2ccccc2CC)c3ccccc3)CC	8.8
58	O=C(N(C1CC[N+](CC1)[C@H](Sc2ccccc2N)C)c3ccccc3)CC	8.8
59	Sc1c(O)cccc1CC[N+]2CCC(N(C(=O)CC)c3ccccc3)CC2	8.8
60	Fc1ccccc1C[C@@H]([N+]2CCC(N(C(=O)CC)c3ccccc3)CC2)C	8.8
61	O=C(N(C1CC[N+](C[C@@H](c2cccs2)C)CC1)c3ccccc3)CC	8.8
62	F[C@@H](C[N+]1CCC(N(C(=O)CC)c2ccccc2)CC1)c3ccc(F)cc3	8.8
63	O=C(N(C1CC[N+](CC1)CSc2ccccc2SC)c3ccccc3)CC	8.8
64	FC(F)(C[N+]1CCC(N(C(=O)CC)c2ccccc2)CC1)c3ccccc3F	8.8
65	O=C(N(C1CC[N+](CC1)CCc2cc(N)ccc2C)c3ccccc3)CC	8.8
66	O=C(N(C1CC[N+](CC1)[C@H](Sc2ccccc2C)C)c3ccccc3)CC	8.8
67	O=C(N(C1CC[N+](CC1)CCc2ccc(N)c(N)c2)c3ccccc3)CC	8.8
68	O=C(N(C1CC[N+](CC1)CCc2ccc(O)c(N)c2)c3ccccc3)CC	8.8
69	Clc1ccc([C@H](C[N+]2CCC(N(C(=O)CC)c3ccccc3)CC2)C)cc1	8.8
70	O=C(N(C1CC[N+](CC1)CCc2ccc(N)cc2N)c3ccccc3)CC	8.8
71	Clc1cnc(CC[N+]2CCC(N(C(=O)CC)c3ccccc3)CC2)cc1	8.8
72	O=C(N(C1CC[N+](CC1)[C@H](Sc2ccc(cc2C)C)C)c3ccccc3)CC	8.8
73	O=C(N(C1CC[N+](CC1)C[C@H](N)c2ccccc2)c3ccccc3)CC	8.8
74	O=C(N(C1CC[N+](CC1)CSc2cccc(n2)C)c3ccccc3)CC	8.8
75	O=C(N(C1CC[N+](CC1)CSc2ccc(N)cc2C)c3ccccc3)CC	8.8
76	O=C(N(C1CC[N+](CC1)CCc2cccc(N)c2C)c3ccccc3)CC	8.8
77	O=C(N(C1CC[N+]([C@H](Cc2cccc(CC)c2)C)CC1)c3ccccc3)CC	8.8
78	O=C(N(C1CC[N+](CC1)[C@H](Sc2ccc(cc2)C)C)c3ccccc3)CC	8.8
79	O=C(N(C1CC[N+](CC1)CCc2ccc(CC)c(N)c2)c3ccccc3)CC	8.8
80	O=C(N(C1CC[N+](CC1)[C@H](Sc2ccccn2)C)c3ccccc3)CC	8.8
81	Cl[C@@H](C[N+]1CCC(N(C(=O)CC)c2ccccc2)CC1)c3ccc(F)cc3	8.8
82	Fc1ccc(F)cc1[C@@H](N)C[N+]2CCC(N(C(=O)CC)c3ccccc3)CC2	8.8
83	O=C(N(C1CC[N+](CC1)CCc2ccc(N)cc2CC)c3ccccc3)CC	8.8
84	Fc1ccccc1CC[N+]2CCC(N(C(=O)CC)c3ccccc3)CC2	8.7
85	O=C(N(C1CC[N+]([C@H](Cc2ccccc2)C)CC1)c3ccccc3)CC	8.7
86	O=C(N(C1CC[N+](CC1)CCc2cc[nH]c2)c3ccccc3)CC	8.7
87	O=C(N(C1CC[N+](CC1)CCc2ccccn2)c3ccccc3)CC	8.7
88	Fc1c(cccc1CC[N+]2CCC(N(C(=O)CC)c3ccccc3)CC2)C	8.7
89	O=C(N(C1CC[N+](CC1)CSc2cccc(c2)C)c3ccccc3)CC	8.7
90	O=C(N(C1CC[N+](CC1)CSc2ccccc2N)c3ccccc3)CC	8.7
91	O=C(N(C1CC[N+](CC1)CCc2ccn(c2)C)c3ccccc3)CC	8.7
92	Cl[C@H]([N+]1CCC(N(c2ccccc2)C(=O)CC)CC1)Cc3ccccc3	8.7
93	O=C(N(C1CC[N+](CC1)CCc2ccccc2SC)c3ccccc3)CC	8.7
94	Oc1cccc(c1CC[N+]2CCC(N(C(=O)CC)c3ccccc3)CC2)C	8.7
95	O=C(N(C1CC[N+](CC1)CSc2ccccn2)c3ccccc3)CC	8.7

96	O=C(N(C1CC[N+](CC1)CCc2ccc(c(N)c2)C)c3ccccc3)CC	8.7
97	Clc1ccc(O)c(CC[N+]2CCC(N(C(=O)CC)c3ccccc3)CC2)c1	8.7
98	Fc1ccccc1S[C@@H]([N+]2CCC(N(C(=O)CC)c3ccccc3)CC2)C	8.7
99	O=[S@](C[N+]1CCC(N(C(=O)CC)c2ccccc2)CC1)c3ccccc3C	8.7
100	Clc1cccc(CC[N+]2CCC(N(C(=O)CC)c3ccccc3)CC2)c1O	8.7
101	O=C(N(C1CC[N+](CC1)CCc2cccc(c2OC)C)c3ccccc3)CC	8.7
102	FC(F)(C[N+]1CCC(N(c2ccccc2)C(=O)CC)CC1)c3ccccc3C	8.7
103	Clc1ccccc1C[C@@H]([N+]2CCC(N(C(=O)CC)c3ccccc3)CC2)C	8.7
104	O=C(N(C1CC[N+](CC1)CCc2ccc(NC)cc2)c3ccccc3)CC	8.7
105	Clc1cc(ccc1CC[N+]2CCC(N(C(=O)CC)c3ccccc3)CC2)C	8.7
106	O=C(N(C1CC[N+](CC1)CCc2ccc(N)c(O)c2)c3ccccc3)CC	8.7
107	O=C(N(C1CC[N+](CC1)CSc2ccc(CC)cc2)c3ccccc3)CC	8.7
108	Sc1ccc(CC[N+]2CCC(N(C(=O)CC)c3ccccc3)CC2)c(F)c1	8.7
109	O=C(N(C1CC[N+](CC1)CSc2ccc([nH+]c2)N)c3ccccc3)CC	8.7
110	O=C(N(C1CC[N+](CC1)CSc2ccc(O)cc2)c3ccccc3)CC	8.7
111	O=C(N(C1CC[N+](CC1)CCc2cccc(N)c2OC)c3ccccc3)CC	8.7
112	O=C(N(C1CC[N+](CC1)CCc2cc(C)ccn2)c3ccccc3)CC	8.7
113	Fc1c(F)cccc1SC[N+]2CCC(N(C(=O)CC)c3ccccc3)CC2	8.7
114	O=C(N(C1CC[N+](CC1)CCc2ccc(O)c(c2)C)c3ccccc3)CC	8.7
115	Clc1cccc(N)c1SC[N+]2CCC(N(C(=O)CC)c3ccccc3)CC2	8.7
116	Sc1cc(F)ccc1CC[N+]2CCC(N(C(=O)CC)c3ccccc3)CC2	8.7
117	O=C(N(C1CC[N+](CC1)CSc2ccc(cc2N)C)c3ccccc3)CC	8.7
118	Clc1cccc(S)c1CC[N+]2CCC(N(C(=O)CC)c3ccccc3)CC2	8.7
119	Fc1cc(O)ccc1CC[N+]2CCC(N(C(=O)CC)c3ccccc3)CC2	8.7
120	O=C(N(C1CC[N+](CC1)[C@@H](N)Cc2ccsc2)c3ccccc3)CC	8.7
121	O=C(N(C1CC[N+](CC1)CCc2cc(ccc2OC)C)c3ccccc3)CC	8.7
122	O=[S@](C[N+]1CCC(N(C(=O)CC)c2ccccc2)CC1)c3c(N)cccn3	8.7
123	Clc1cccnc1SC[N+]2CCC(N(C(=O)CC)c3ccccc3)CC2	8.7
124	Fc1cccc(F)c1C[C@@H]([N+]2CCC(N(C(=O)CC)c3ccccc3)CC2)C	8.7
125	Clc1ccc(C(F)(F)C[N+]2CCC(N(C(=O)CC)c3ccccc3)CC2)cc1	8.7
126	O=C(N(C1CC[N+]([C@H](Cc2ccc(CC)cc2)C)CC1)c3ccccc3)CC	8.7
127	O=C(N(C1CC[N+](CC1)C[C@@H](c2ccccc2)CC#N)c3ccccc3)CC	8.7
128	O=C(N(C1CC[N+](CC1)CCc2c[nH]c3ccccc32)c4ccccc4)CC	8.7
129	O=C(N(C1CC[N+](CC1)CCc2c(N)cccc2SC)c3ccccc3)CC	8.7
130	OB1c2c(CO1)cccc2CC[N+]3CCC(N(C(=O)CC)c4ccccc4)CC3	8.7
131	O[C@@H]([N+]1CCC(N(c2ccccc2)C(=O)CC)CC1)[C@@H](c3ccccc3)C	8.7
132	O=C(N(C1CC[N+](CC1)CSc2ccccc2)c3ccccc3)CC	8.6
133	O=C(N(C1CC[N+](CC1)CSc2cccs2)c3ccccc3)CC	8.6
134	Sc1ccccc1CC[N+]2CCC(N(C(=O)CC)c3ccccc3)CC2	8.6
135	F[C@H]([N+]1CCC(N(c2ccccc2)C(=O)CC)CC1)Cc3ccccc3	8.6
136	O=C(N(C1CC[N+](CC1)CCc2ccc(cc2)C)c3ccccc3)CC	8.6
137	O=C(N(C1CC[N+](CC1)CCc2c(O)cccc2O)c3ccccc3)CC	8.6

138	Clc1ccccc1CC[N+]2CCC(N(C(=O)CC)c3ccccc3)CC2	8.6
139	O=C(N(C1CC[N+](CC1)C[S@@](=O)c2ccccc2N)c3ccccc3)CC	8.6
140	O=C(N(C1CC[N+](CC1)CSc2ccc(cc2)C)c3ccccc3)CC	8.6
141	O=C(N(C1CC[N+](CC1)CCc2cccc(N)c2)c3ccccc3)CC	8.6
142	Clc1ccc(SC[N+]2CCC(N(C(=O)CC)c3ccccc3)CC2)cc1	8.6
143	O=C(N(C1CC[N+](CC1)CCc2cccc(O)c2N)c3ccccc3)CC	8.6
144	Clc1c(N)cccc1CC[N+]2CCC(N(C(=O)CC)c3ccccc3)CC2	8.6
145	O=C(N(C1CC[N+](CC1)CCc2ccc([nH+]c2)N)c3ccccc3)CC	8.6
146	O=C(N(C1CC[N+](CC1)CCc2cccc(NC)c2)c3ccccc3)CC	8.6
147	O=C(N(C1CC[N+](CC1)CSc2c(cccc2C)C)c3ccccc3)CC	8.6
148	Fc1cc(ccc1CC[N+]2CCC(N(C(=O)CC)c3ccccc3)CC2)C	8.6
149	Clc1ccc(F)c(CC[N+]2CCC(N(C(=O)CC)c3ccccc3)CC2)c1	8.6
150	Sc1c(cccc1CC[N+]2CCC(N(C(=O)CC)c3ccccc3)CC2)C	8.6
151	Clc1c(cccc1CC[N+]2CCC(N(C(=O)CC)c3ccccc3)CC2)C	8.6
152	Fc1ccc(CC[N+]2CCC(N(C(=O)CC)c3ccccc3)CC2)cc1N	8.6
153	O=C(N(C1CC[N+](CC1)CCc2ccc3c(c2)cc[nH]3)c4ccccc4)CC	8.6
154	O=C(N(C1CC[N+](CC1)CSc2cccc(O)c2)c3ccccc3)CC	8.6
155	Clc1cccc(N)c1CC[N+]2CCC(N(C(=O)CC)c3ccccc3)CC2	8.6
156	Fc1ccc(SC[N+]2CCC(N(C(=O)CC)c3ccccc3)CC2)cc1C	8.6
157	O=C(N(C1CC[N+](CC1)CCc2ccc(cc2N)C)c3ccccc3)CC	8.6
158	O=C(N(C1CC[N+]([C@@H]2[C@H](O2)c3ccccc3)CC1)c4ccccc4)CC	8.6
159	Fc1c(CC)cccc1CC[N+]2CCC(N(c3ccccc3)C(=O)CC)CC2	8.6
160	Fc1cnc(CC[N+]2CCC(N(C(=O)CC)c3ccccc3)CC2)cc1	8.6
161	O=C(N(C1CC[N+](CC1)COc2ccc(cc2)C)c3ccccc3)CC	8.6
162	O=C(N(C1CC[N+](CC1)CSc2cc(cc(c2)C)C)c3ccccc3)CC	8.6
163	O=C(N(C1CC[N+]([C@@H]2[C@H](O2)c3cccc(c3)C)CC1)c4ccccc4)CC	8.6
164	O=C(N(C1CC[N+](CC1)CCc2cccnc2OC)c3ccccc3)CC	8.6
165	Fc1cccc(CC[N+]2CCC(N(C(=O)CC)c3ccccc3)CC2)c1C(F)F	8.6
166	O=C(N(C1CC[N+](CC1)CCc2c(N)cccc2CC)c3ccccc3)CC	8.6
167	O=C(N(C1CC[N+](CC1)CSc2cccc(SC)c2)c3ccccc3)CC	8.6
168	O=C(N(C1CC[N+](CC1)CCc2ccc(N)c(c2)C)c3ccccc3)CC	8.6
169	Sc1cccc(S)c1CC[N+]2CCC(N(c3ccccc3)C(=O)CC)CC2	8.6
170	O=C(N(C1CC[N+](CC1)CCc2ccc(C3CC3)cc2)c4ccccc4)CC	8.6
171	Fc1cccc(c1CC[N+]2CCC(N(C(=O)CC)c3ccccc3)CC2)CC	8.6
172	O=C(N(C1CC[N+](CC1)CCCc2ccccc2O)c3ccccc3)CC	8.6
173	O=C(N(C1CC[N+](CC1)CSc2cc[nH+]c(NN)c2)c3ccccc3)CC	8.6
174	Fc1cccc(OC)c1CC[N+]2CCC(N(C(=O)CC)c3ccccc3)CC2	8.6
175	Fc1cccnc1CC[N+]2CCC(N(C(=O)CC)c3ccccc3)CC2	8.6
176	O=C(N(C1CC[N+](CC1)CCc2ccc(N)c(c2)CC)c3ccccc3)CC	8.6
177	O=C(N(C1CC[N+](CC1)CCc2ccc(cc2OC)C)c3ccccc3)CC	8.6
178	O=C(N(C1CC[N+](CC1)CCc2cc(N)ccc2CC)c3ccccc3)CC	8.6
179	O=C(N(C1CC[N+](CC1)CSc2ccnc(O)n2)c3ccccc3)CC	8.6

180	O[C@H]([N+]1CCC(N(c2ccccc2)C(=O)CC)CC1)[C@H](O)c3ccccc3	8.6
181	FC(F)c1cc(N)ccc1CC[N+]2CCC(N(C(=O)CC)c3ccccc3)CC2	8.6
182	O=C(N(C1CC[N+](CC1)CCc2cccc(SC)c2N)c3ccccc3)CC	8.6
183	O=C(N(C1CC[N+](CC1)CCc2cccc(N)c2CC)c3ccccc3)CC	8.6
184	O=C(N(C1CC[N+](CC1)CCc2c(C)cccc2OC)c3ccccc3)CC	8.6
185	O=C(N(C1CC[N+](CC1)CCc2ccccc2OC)c3ccccc3)CC	8.6
186	O=C(N(C1CC[N+](CC1)CCc2ccc(cc2CC)C)c3ccccc3)CC	8.6
187	O=C(N(C1CC[N+](CC1)CCc2ccccc2)c3ccccc3)CC	8.5
188	O=C(N(C1CC[N+](CC1)CCc2cc(C)cs2)c3ccccc3)CC	8.5
189	O=C(N(C1CC[N+](CC1)CSc2ccccc2C)c3ccccc3)CC	8.5
190	O=C(N(C1CC[N+](CC1)CC(c2ccccc2C)=C)c3ccccc3)CC	8.5
191	Fc1ccc(SC[N+]2CCC(N(C(=O)CC)c3ccccc3)CC2)cc1	8.5
192	Fc1c(N)cccc1CC[N+]2CCC(N(C(=O)CC)c3ccccc3)CC2	8.5
193	Fc1cccc(CC[N+]2CCC(N(C(=O)CC)c3ccccc3)CC2)c1O	8.5
194	O=C(N(C1CC[N+](CC1)CCc2ccc(cc2)C=C)c3ccccc3)CC	8.5
195	O=C(N(C1CC[N+](CC1)CCc2c(N)cccc2C)c3ccccc3)CC	8.5
196	FC(F)(C[N+]1CCC(N(C(=O)CC)c2ccccc2)CC1)c3cccs3	8.5
197	O=C(N(C1CC[N+](CC1)CCc2c(cccc2C)C)c3ccccc3)CC	8.5
198	O=C(N(C1CC[N+](CC1)CCc2cccc(c2N)C)c3ccccc3)CC	8.5
199	O=C(N(C1CC[N+](CC1)CCc2ccco2)c3ccccc3)CC	8.5
200	Sc1cccc(F)c1CC[N+]2CCC(N(C(=O)CC)c3ccccc3)CC2	8.5
201	Clc1cccc(c1CC[N+]2CCC(N(C(=O)CC)c3ccccc3)CC2)C	8.5
202	Clc1cccc(F)c1CC[N+]2CCC(N(C(=O)CC)c3ccccc3)CC2	8.5
203	O=C(N(C1CC[N+](CC1)CCc2cscn2)c3ccccc3)CC	8.5
204	O=C(N(C1CC[N+](C[C@@H](c2ccccc2)C(N)=[N+])CC1)c3ccccc3)CC	8.5
205	Fc1cc(F)ccc1SC[N+]2CCC(N(C(=O)CC)c3ccccc3)CC2	8.5
206	Sc1ccc(F)c(CC[N+]2CCC(N(C(=O)CC)c3ccccc3)CC2)c1	8.5
207	Clc1cccc(c1SC[N+]2CCC(N(C(=O)CC)c3ccccc3)CC2)C	8.5
208	O=C(N(C1CC[N+](CC1)CCc2c(C)ccc(c2)C)c3ccccc3)CC	8.5
209	O=C(N(C1CC[N+](CC1)C[C@@H](c2ccccc2C)C)c3ccccc3)CC	8.5
210	Clc1ccc(cc1CC[N+]2CCC(N(C(=O)CC)c3ccccc3)CC2)C	8.5
211	O=C(N(C1CC[N+](CC1)CSc2ccoc2C)c3ccccc3)CC	8.5
212	Fc1cccc(SC[N+]2CCC(N(C(=O)CC)c3ccccc3)CC2)c1C	8.5
213	Sc1cccc(c1CC[N+]2CCC(N(C(=O)CC)c3ccccc3)CC2)C	8.5
214	Fc1ccnc(CC[N+]2CCC(N(C(=O)CC)c3ccccc3)CC2)c1	8.5
215	Fc1ccc(O)cc1CC[N+]2CCC(N(C(=O)CC)c3ccccc3)CC2	8.5
216	Clc1cccc(CC[N+]2CCC(N(C(=O)CC)c3ccccc3)CC2)c1S	8.5
217	O=C(N(C1CC[N+](CC1)CSc2ccc(cc2C)C)c3ccccc3)CC	8.5
218	Clc1cc(N)cc(CC[N+]2CCC(N(C(=O)CC)c3ccccc3)CC2)c1	8.5
219	O[C@H](C[N+]1CCC(N(c2ccccc2)C(=O)CC)CC1)c3ccccc3	8.5
220	O=C(N(C1CC[N+](CC1)CCc2c(ccc(c2)CC)C)c3ccccc3)CC	8.5
221	Sc1cc(N)cc(CC[N+]2CCC(N(C(=O)CC)c3ccccc3)CC2)c1	8.5

222	O=C(N(C1CC[N+](CC1)[C@H](CC=2C=CCCC2)C)c3ccccc3)CC	8.5
223	Clc1cccc(CC[N+]2CCC(N(C(=O)CC)c3ccccc3)CC2)c1F	8.5
224	O=C(N(C1CC[N+](CC1)CCc2ccc(O)cc2)c3ccccc3)CC	8.5
225	Sc1c(O)ccc(CC[N+]2CCC(N(C(=O)CC)c3ccccc3)CC2)c1	8.5
226	O=C(N(C1CC[N+](CC1)CCc2cc(N)ccc2N)c3ccccc3)CC	8.5
227	Clc1c(ccc(SC[N+]2CCC(N(C(=O)CC)c3ccccc3)CC2)c1)C	8.5
228	Clc1ccc(SC[N+]2CCC(N(C(=O)CC)c3ccccc3)CC2)c(C)c1	8.5
229	O=C(N(C1CC[N+](CC1)CSc2cnc(s2)N)c3ccccc3)CC	8.5
230	Fc1cc(N)cc(CC[N+]2CCC(N(C(=O)CC)c3ccccc3)CC2)c1	8.5
231	FC(F)(C[N+]1CCC(N(C(=O)CC)c2ccccc2)CC1)c3ccccc3OC	8.5
232	O=C(N(C1CC[N+](CC1)CCc2cccc(c2N)CC)c3ccccc3)CC	8.5
233	Fc1c(ccc(SC[N+]2CCC(N(C(=O)CC)c3ccccc3)CC2)c1)C	8.5
234	Clc1cc(S)ccc1CC[N+]2CCC(N(C(=O)CC)c3ccccc3)CC2	8.5
235	O=C(N(C1CC[N+](CC1)CSc2ccc(c(c2)C)C)c3ccccc3)CC	8.5
236	Clc1cccc(CC[N+]2CCC(N(C(=O)CC)c3ccccc3)CC2)c1OC	8.5
237	O=C(N(C1CC[N+](CC1)CCc2c(N)ccc(c2)CC)c3ccccc3)CC	8.5
238	FC(F)c1c(CC[N+]2CCC(N(C(=O)CC)c3ccccc3)CC2)cco1	8.5
239	O=C(N(C1CC[N+](CC1)CCc2ccc3c(CCN3)c2)c4ccccc4)CC	8.5
240	Clc1cccc(c1CC[N+]2CCC(N(C(=O)CC)c3ccccc3)CC2)CC	8.5
241	Fc1cccc(F)c1CC[N+]2CCC(N(C(=O)CC)c3ccccc3)CC2	8.5
242	O=C(N(C1CC[N+](CC1)CCc2c(c[nH]c2C)C)c3ccccc3)CC	8.5
243	O=C(N(C1CC[N+](CC1)CCc2ccc(CC)cc2N)c3ccccc3)CC	8.5
244	O=C(N(C1CC[N+]([C@@H]2[C@H](O2)c3ccccc3C)CC1)c4ccccc4)CC	8.5
245	Fc1cc(F)ccc1[C@H](C[N+]2CCC(N(C(=O)CC)c3ccccc3)CC2)C	8.5
246	O=C(N(C1CC[N+](CC1)CSc2ccncc2N)c3ccccc3)CC	8.5
247	O=C(N(C1CC[N+](CC1)CSc2cccc(OC)c2)c3ccccc3)CC	8.5
248	O=C(N(C1CC[N+](CC1)CSc2nc(C)cs2)c3ccccc3)CC	8.5
249	O=C(N(C1CC[N+](CC1)CCc2ccccc2NN)c3ccccc3)CC	8.5
250	O=C(N(C1CC[N+](CC1)CSc2c(cc(cc2C)C)C)c3ccccc3)CC	8.5
251	Clc1cc(CC)ccc1CC[N+]2CCC(N(C(=O)CC)c3ccccc3)CC2	8.5
252	O=C(N(C1CC[N+](CC1)CCc2c[nH+]c(N)c(c2)C)c3ccccc3)CC	8.5
253	Clc1cccc(SC[N+]2CCC(N(C(=O)CC)c3ccccc3)CC2)c1C	8.5
254	O=C(N(C1CC[N+](CC1)CCc2ccc(OC)cc2)c3ccccc3)CC	8.5
255	O=C(N(C1CC[N+]([C@H](Cc2ccccc2)C(=O)C)CC1)c3ccccc3)CC	8.5
256	Clc1ccc(CC[N+]2CCC(N(C(=O)CC)c3ccccc3)CC2)c(S)c1	8.5
257	O=C(N(C1CC[N+](CC1)CSc2ccc3CCCc3c2)c4ccccc4)CC	8.5
258	O=C(N(C1CC[N+](CC1)CCc2ccc(OC(=O)C)cc2)c3ccccc3)CC	8.5
259	O=C(N(C1CC[N+](CC1)CCc2ccccc2C)c3ccccc3)CC	8.4
260	O=C(N(C1CC[N+](CC1)CCc2ccccc2C#C)c3ccccc3)CC	8.4
261	Fc1cccc(SC[N+]2CCC(N(C(=O)CC)c3ccccc3)CC2)c1	8.4
262	Fc1c(N)ccc(CC[N+]2CCC(N(C(=O)CC)c3ccccc3)CC2)c1	8.4
263	Sc1ccc(CC[N+]2CCC(N(C(=O)CC)c3ccccc3)CC2)cc1	8.4

264	Fc1ccc(cc1CC[N+]2CCC(N(C(=O)CC)c3ccccc3)CC2)C	8.4
265	O=C(N(C1CC[N+](CC1)CCc2nccs2)c3ccccc3)CC	8.4
266	Fc1ccc(CC[N+]2CCC(N(C(=O)CC)c3ccccc3)CC2)cc1	8.4
267	O=C(N(C1CC[N+](CC1)C[C@H]2C=Cc3ccccc32)c4ccccc4)CC	8.4
268	Sc1ccc(c(CC[N+]2CCC(N(C(=O)CC)c3ccccc3)CC2)c1)C	8.4
269	O=C(N(C1CC[N+](CC1)CCc2ccc(CC)cc2)c3ccccc3)CC	8.4
270	Sc1c(F)cccc1CC[N+]2CCC(N(C(=O)CC)c3ccccc3)CC2	8.4
271	O=C(N(C1CC[N+](CC1)CCc2ccc(cc2C)C)c3ccccc3)CC	8.4
272	FC(F)c1ccccc1CC[N+]2CCC(N(c3ccccc3)C(=O)CC)CC2	8.4
273	Sc1ccc(CC[N+]2CCC(N(C(=O)CC)c3ccccc3)CC2)c(c1)C	8.4
274	O=C(N(C1CC[N+](CC1)CCc2ccc(c(c2)C)C)c3ccccc3)CC	8.4
275	O=C(N(C1CC[N+](CC1)CCc2cccc(O)c2C)c3ccccc3)CC	8.4
276	Clc1c(S)cccc1CC[N+]2CCC(N(C(=O)CC)c3ccccc3)CC2	8.4
277	O=C(N(C1CC[N+](CC1)CCc2cccc3c2OCO3)c4ccccc4)CC	8.4
278	O=C(N(C1CC[N+](CC1)CSc2c(C)ccc(c2)C)c3ccccc3)CC	8.4
279	Fc1cccc(CC[N+]2CCC(N(C(=O)CC)c3ccccc3)CC2)c1F	8.4
280	O=C(N(C1CC[N+](CC1)CSc2ccncc2)c3ccccc3)CC	8.4
281	Fc1ccc(F)cc1SC[N+]2CCC(N(C(=O)CC)c3ccccc3)CC2	8.4
282	Fc1ccc(F)c(CC[N+]2CCC(N(C(=O)CC)c3ccccc3)CC2)c1	8.4
283	O=C(N(C1CC[N+](CC1)CCc2ncc(s2)C)c3ccccc3)CC	8.4
284	O=C(N(C1CC[N+](CC1)CCc2ccc(SC)cc2)c3ccccc3)CC	8.4
285	O=C(N(C1CC[N+](CC1)CSc2cnccc2)c3ccccc3)CC	8.4
286	O=C(N(C1CC[N+](CC1)CCc2ccccc2C(C)C)c3ccccc3)CC	8.4
287	Fc1ccc(cc1CC[N+]2CCC(N(C(=O)CC)c3ccccc3)CC2)CC	8.4
288	FC(F)(C[N+]1CCC(N(C(=O)CC)c2ccccc2)CC1)c3cccc(c3)C	8.4
289	Sc1c(N)ccc(CC[N+]2CCC(N(C(=O)CC)c3ccccc3)CC2)c1	8.4
290	Fc1ccc(c(SC[N+]2CCC(N(C(=O)CC)c3ccccc3)CC2)c1)C	8.4
291	Sc1ccc(CC[N+]2CCC(N(C(=O)CC)c3ccccc3)CC2)c(N)c1	8.4
292	Sc1cc(ccc1CC[N+]2CCC(N(C(=O)CC)c3ccccc3)CC2)C	8.4
293	Fc1cccc(C[C@@H]([N+]2CCC(N(C(=O)CC)c3ccccc3)CC2)C)c1	8.4
294	O=C(N(C1CC[N+](CC1)CCc2ccc(cc2C)CC)c3ccccc3)CC	8.4
295	O=C(N(C1CC[N+](CC1)CCc2c(cccc2CC)C)c3ccccc3)CC	8.4
296	Clc1c(cccc1CC[N+]2CCC(N(C(=O)CC)c3ccccc3)CC2)CF	8.4
297	Clc1cc(F)ccc1CC[N+]2CCC(N(C(=O)CC)c3ccccc3)CC2	8.4
298	Clc1ccc(CC[N+]2CCC(N(C(=O)CC)c3ccccc3)CC2)c(CF)c1	8.4
299	O=C(N(C1CC[N+](CC1)CCc2cccc(c2C)CC)c3ccccc3)CC	8.4
300	Fc1ccc(SC[N+]2CCC(N(C(=O)CC)c3ccccc3)CC2)c(C)c1	8.4
301	O=C(N(C1CC[N+](CC1)CCc2cc[n+](cc2)C)c3ccccc3)CC	8.4
302	O=C(N(C1CC[N+](CC1)C[C@H](N)c2ccccc2C)c3ccccc3)CC	8.4
303	Clc1ccc(Cl)c(CC[N+]2CCC(N(C(=O)CC)c3ccccc3)CC2)c1	8.4
304	O=C(N(C1CC[N+](CC1)CCc2cc(ccc2CC)C)c3ccccc3)CC	8.4
305	O=C(N(C1CC[N+](CC1)CCc2cocn2)c3ccccc3)CC	8.4

306	O=C(N(C1CC[N+](CC1)CCc2cccc(c2CC)C)c3ccccc3)CC	8.4
307	Clc1cccc(CC[N+]2CCC(N(C(=O)CC)c3ccccc3)CC2)c1CF	8.4
308	O=C(N(C1CC[N+](CC1)CSc2ccccc2C(C)C)c3ccccc3)CC	8.4
309	Fc1cc(CC)ccc1CC[N+]2CCC(N(C(=O)CC)c3ccccc3)CC2	8.4
310	O=C(N(C1CC[N+](CC1)CCc2ccc3cc[nH]c3c2)c4ccccc4)CC	8.4
311	O=C(N(C1CC[N+](CC1)[C@H](Cc2cccc3c2cco3)C)c4ccccc4)CC	8.4
312	Clc1ccc(S)cc1CC[N+]2CCC(N(C(=O)CC)c3ccccc3)CC2	8.4
313	Fc1c(SC)cccc1CC[N+]2CCC(N(C(=O)CC)c3ccccc3)CC2	8.4
314	O=C(N(C1CC[N+](CC1)CCc2ccnc(n2)N)c3ccccc3)CC	8.4
315	Clc1c(O)cccc1CC[N+]2CCC(N(C(=O)CC)c3ccccc3)CC2	8.4
316	Fc1ccc(SC[N+]2CCC(N(C(=O)CC)c3ccccc3)CC2)c(N)c1	8.4
317	Clc1cccc(c1CC[N+]2CCC(N(C(=O)CC)c3ccccc3)CC2)CF	8.4
318	O=C(N(C1CC[N+](CC1)CC/C(C)=C/CO)c2ccccc2)CC	8.4
319	Clc1cc(F)ccc1SC[N+]2CCC(N(C(=O)CC)c3ccccc3)CC2	8.4
320	Clc1ccc(c(SC[N+]2CCC(N(C(=O)CC)c3ccccc3)CC2)c1)C	8.4
321	Clc1cc(O)ccc1CC[N+]2CCC(N(C(=O)CC)c3ccccc3)CC2	8.4
322	O=C(N(C1CC[N+](CC1)[C@H](Cc2cccs2)C)c3ccccc3)CC	8.4
323	Oc1cc(ccc1C(C[N+]2CCC(N(C(=O)CC)c3ccccc3)CC2)=C)C	8.4
324	Fc1cccc(CC[N+]2CCC(N(C(=O)CC)c3ccccc3)CC2)c1SC	8.4
325	Fc1ccc(SC)cc1CC[N+]2CCC(N(C(=O)CC)c3ccccc3)CC2	8.4
326	Clc1ccc(CC[N+]2CCC(N(C(=O)CC)c3ccccc3)CC2)c(C)c1	8.4
327	Clc1cccc(Cl)c1CC[N+]2CCC(N(C(=O)CC)c3ccccc3)CC2	8.4
328	Fc1ccc(OC)cc1CC[N+]2CCC(N(C(=O)CC)c3ccccc3)CC2	8.4
329	Fc1cc(OC)ccc1CC[N+]2CCC(N(C(=O)CC)c3ccccc3)CC2	8.4
330	O=C(N(C1CC[N+](CC1)CCc2cccc(c2)C)c3ccccc3)CC	8.3
331	Sc1cccc(CC[N+]2CCC(N(C(=O)CC)c3ccccc3)CC2)c1	8.3
332	Fc1cccc(CC[N+]2CCC(N(C(=O)CC)c3ccccc3)CC2)c1C	8.3
333	Clc1cccc(CC[N+]2CCC(N(C(=O)CC)c3ccccc3)CC2)c1	8.3
334	O=C(N(C1CC[N+](CC1)CCC2=CCCC2)c3ccccc3)CC	8.3
335	O=C(N(C1CC[N+](CC1)CCc2c(N)cccc2N)c3ccccc3)CC	8.3
336	Fc1c(ccc(CC[N+]2CCC(N(C(=O)CC)c3ccccc3)CC2)c1)C	8.3
337	O=C(N(C1CC[N+](CC1)CCc2cc(cc(c2)C)C)c3ccccc3)CC	8.3
338	Clc1ccc(CC[N+]2CCC(N(C(=O)CC)c3ccccc3)CC2)cc1	8.3
339	Clc1cccc(CC[N+]2CCC(N(C(=O)CC)c3ccccc3)CC2)c1C	8.3
340	O=C(N(C1CC[N+](CC1)CCc2cccc(c2C)C)c3ccccc3)CC	8.3
341	O=C(N(C1CC[N+](CC1)CCc2cccc(O)c2)c3ccccc3)CC	8.3
342	Clc1cccc(SC[N+]2CCC(N(C(=O)CC)c3ccccc3)CC2)c1	8.3
343	Clc1cccc(CC[N+]2CCC(N(C(=O)CC)c3ccccc3)CC2)c1N	8.3
344	O=C(N(C1CC[N+](CC1)[C@H](Cc2ccsc2)C)c3ccccc3)CC	8.3
345	Fc1ccccc1[C@@H](N)C[N+]2CCC(N(C(=O)CC)c3ccccc3)CC2	8.3
346	O=C(N(C1CC[N+](CC1)CCc2c(N)ccc(c2)C)c3ccccc3)CC	8.3
347	O=C(N(C1CC[N+](C2(CC2)Cc3ccccc3)CC1)c4ccccc4)CC	8.3

348	O=C(N(C1CC[N+](CC1)CCc2ccc(s2)C)c3ccccc3)CC	8.3
349	O=C(N(C1CC[N+](CC1)CCc2cc(N)cc(c2)C)c3ccccc3)CC	8.3
350	O=C(N(C1CC[N+](CC1)CCc2ccc(N)c(OC)c2)c3ccccc3)CC	8.3
351	Fc1cccc(O)c1CC[N+]2CCC(N(C(=O)CC)c3ccccc3)CC2	8.3
352	Clc1coc(CC[N+]2CCC(N(C(=O)CC)c3ccccc3)CC2)c1	8.3
353	O=C(N(C1CC[N+](CC1)CCc2ccc(CC)c(c2)C)c3ccccc3)CC	8.3
354	Clc1cc(F)c(CC[N+]2CCC(N(C(=O)CC)c3ccccc3)CC2)cc1	8.3
355	O=C(N(C1CC[N+](CC1)CCc2ccc3CCCc3c2)c4ccccc4)CC	8.3
356	Clc1c(ccc(CC[N+]2CCC(N(C(=O)CC)c3ccccc3)CC2)c1)C	8.3
357	FC(F)(C[N+]1CCC(N(C(=O)CC)c2ccccc2)CC1)c3ccc(F)cc3	8.3
358	O=C(N(C1CC[N+](CC1)CCN2CCCC2)c3ccccc3)CC	8.3
359	O=C(N(C1CC[N+](CC1)CCc2ccc(c(O)c2)C)c3ccccc3)CC	8.3
360	O=C(N(C1CC[N+](CC1)CCc2c(N)cccc2OC)c3ccccc3)CC	8.3
361	Clc1ccc(CC[N+]2CCC(N(C(=O)CC)c3ccccc3)CC2)cc1C	8.3
362	O=C(N(C1CC[N+](CC1)CCc2ccc([nH+]c2C)N)c3ccccc3)CC	8.3
363	O=C(N(C1CC[N+](CC1)CCc2nc(C)cs2)c3ccccc3)CC	8.3
364	Clc1ccc(S)c(CC[N+]2CCC(N(C(=O)CC)c3ccccc3)CC2)c1	8.3
365	Clc1cc(F)cc(SC[N+]2CCC(N(C(=O)CC)c3ccccc3)CC2)c1	8.3
366	O=C(N(C1CC[N+](CC1)CCc2ccc3c(c2)ccs3)c4ccccc4)CC	8.3
367	O=C(N(C1CC[N+](CC1)CCc2cccc(c2)C(C)C)c3ccccc3)CC	8.3
368	O=C(N(C1CC[N+](CC1)CCc2cc(O)ccc2C)c3ccccc3)CC	8.3
369	FC(F)c1ccc(c(CC[N+]2CCC(N(C(=O)CC)c3ccccc3)CC2)c1)C	8.3
370	O=C(N(C1CC[N+](CC1)CCc2ccc(c(CC)c2)C)c3ccccc3)CC	8.3
371	O=C(N(C1CC[N+](CC1)CCc2ccc3COCc3c2)c4ccccc4)CC	8.3
372	FC(F)c1ccc(CC[N+]2CCC(N(C(=O)CC)c3ccccc3)CC2)c(c1)C	8.3
373	Clc1cccc(O)c1CC[N+]2CCC(N(C(=O)CC)c3ccccc3)CC2	8.3
374	Clc1ccc(SC[N+]2CCC(N(C(=O)CC)c3ccccc3)CC2)cc1C	8.3
375	FC(F)c1cc(ccc1CC[N+]2CCC(N(C(=O)CC)c3ccccc3)CC2)C	8.3
376	O=C(N(C1CC[N+](CC1)COc2ccccc2N)c3ccccc3)CC	8.3
377	O=C(N(C1CC[N+](CC1)CCc2c(C)ccs2)c3ccccc3)CC	8.2
378	O=C(N(C1CC[N+](CC1)CCc2ccc[nH]2)c3ccccc3)CC	8.2
379	Clc1csc(CC[N+]2CCC(N(C(=O)CC)c3ccccc3)CC2)c1	8.2
380	O=C(N(C1CC[N+](CC1)CCc2cc[nH+]c(N)c2)c3ccccc3)CC	8.2
381	O=C(N(C1CC[N+](CC1)CCC2=CCCCC2)c3ccccc3)CC	8.2
382	O=C(N(C1CC[N+](CC1)C[S@@](=O)c2ccccc2)c3ccccc3)CC	8.2
383	O=C(N(C1CC[N+](CC1)CCc2cccc(N)c2O)c3ccccc3)CC	8.2
384	O=C(N(C1CC[N+](CC1)CCc2cccn2C)c3ccccc3)CC	8.2
385	O=C(N(C1CC[N+](CC1)CCc2cccc(c2)CC)c3ccccc3)CC	8.2
386	Fc1ccc(c(CC[N+]2CCC(N(C(=O)CC)c3ccccc3)CC2)c1)C	8.2
387	F[C@H](C[N+]1CCC(N(C(=O)CC)c2ccccc2)CC1)c3ccc(cc3)C	8.2
388	O=C(N(C1CC[N+](CC1)COc2ccccc2C=C)c3ccccc3)CC	8.2
389	Sc1cccc(CC[N+]2CCC(N(C(=O)CC)c3ccccc3)CC2)c1C	8.2

390	Sc1c(F)ccc(CC[N+]2CCC(N(C(=O)CC)c3ccccc3)CC2)c1	8.2
391	O=C(N(C1CC[N+](CC1)CCc2ccoc2)c3ccccc3)CC	8.2
392	O=C(N(C1CC[N+](CC1)CCc2c(sc(c2)C)C)c3ccccc3)CC	8.2
393	Clc1ccc(F)cc1CC[N+]2CCC(N(C(=O)CC)c3ccccc3)CC2	8.2
394	Fc1cc(F)cc(SC[N+]2CCC(N(C(=O)CC)c3ccccc3)CC2)c1	8.2
395	Clc1cc(cc(CC[N+]2CCC(N(C(=O)CC)c3ccccc3)CC2)c1)C	8.2
396	Fc1ccc(CC[N+]2CCC(N(C(=O)CC)c3ccccc3)CC2)c(C)c1	8.2
397	O=C(N(C1CC[N+](CC1)CCc2cccc(OC)c2N)c3ccccc3)CC	8.2
398	O=C(N(C1CC[N+](CC1)CCc2csc(c2C)C)c3ccccc3)CC	8.2
399	O=C(N(C1CC[N+](CC1)CCc2cc(cc(CC)c2)C)c3ccccc3)CC	8.2
400	Clc1ccc(CC[N+]2CCC(N(C(=O)CC)c3ccccc3)CC2)cc1N	8.2
401	O=C(N(C1CC[N+](CC1)CCc2ccc(O)c(O)c2)c3ccccc3)CC	8.2
402	Clc1cccc(CC[N+]2CCC(N(C(=O)CC)c3ccccc3)CC2)c1CC	8.2
403	Sc1ccc(CC[N+]2CCC(N(C(=O)CC)c3ccccc3)CC2)cc1N	8.2
404	Fc1cc(CC[N+]2CCC(N(C(=O)CC)c3ccccc3)CC2)ccc1CC	8.2
405	O=C(N(C1CC[N+](CC1)CCc2cccc3ccccc32)c4ccccc4)CC	8.2
406	O=C(N(C1CC[N+](CC1)[C@@H](N)Cc2cccc(c2)C)c3ccccc3)CC	8.2
407	Clc1ccc(c(CC[N+]2CCC(N(C(=O)CC)c3ccccc3)CC2)c1)C	8.2
408	Fc1c(F)ccc(CC[N+]2CCC(N(C(=O)CC)c3ccccc3)CC2)c1	8.2
409	Clc1ccc(O)cc1CC[N+]2CCC(N(C(=O)CC)c3ccccc3)CC2	8.2
410	Clc1ccc(o1)CC[N+]2CCC(N(C(=O)CC)c3ccccc3)CC2	8.2
411	Fc1cc(F)cc(F)c1CC[N+]2CCC(N(C(=O)CC)c3ccccc3)CC2	8.2
412	O=C(N(C1CC[N+](CC1)CCc2c([N+]([O-])=O)cccc2C)c3ccccc3)CC	8.2
413	Fc1c(O)cccc1CC[N+]2CCC(N(C(=O)CC)c3ccccc3)CC2	8.2
414	O=C(N(C1CC[N+](CC1)CCc2ccccc2N)c3ccccc3)CC	8.1
415	O=C(N(C1CC[N+](CC1)CCn2cccc2)c3ccccc3)CC	8.1
416	Fc1cccc(CC[N+]2CCC(N(C(=O)CC)c3ccccc3)CC2)c1	8.1
417	FC(F)c1cccc(CC[N+]2CCC(N(C(=O)CC)c3ccccc3)CC2)c1	8.1
418	Sc1ccc(F)cc1CC[N+]2CCC(N(C(=O)CC)c3ccccc3)CC2	8.1
419	Sc1cccc(N)c1CC[N+]2CCC(N(C(=O)CC)c3ccccc3)CC2	8.1
420	O=C(N(C1CC[N+](CC1)CCc2cccnc2C)c3ccccc3)CC	8.1
421	Sc1ccc(cc1CC[N+]2CCC(N(C(=O)CC)c3ccccc3)CC2)C	8.1
422	Clc1ccc(CC[N+]2CCC(N(C(=O)CC)c3ccccc3)CC2)c(N)c1	8.1
423	O=C(N(C1CC[N+](CC1)CCc2cnccc2)c3ccccc3)CC	8.1
424	Sc1ccc(CC[N+]2CCC(N(C(=O)CC)c3ccccc3)CC2)cc1C	8.1
425	O=C(N(C1CC[N+](CC1)CCc2ccncc2)c3ccccc3)CC	8.1
426	O=C(N(C1CC[N+](CC1)CCc2cccc(O)c2O)c3ccccc3)CC	8.1
427	Fc1cc(cc(CC[N+]2CCC(N(C(=O)CC)c3ccccc3)CC2)c1)C	8.1
428	Fc1ccc(CC[N+]2CCC(N(C(=O)CC)c3ccccc3)CC2)cc1C	8.1
429	O=C(N(C1CC[N+](CC1)CCc2cccc(c2O)C)c3ccccc3)CC	8.1
430	Oc1cc(N)ccc1CC[N+]2CCC(N(C(=O)CC)c3ccccc3)CC2	8.1
431	Clc1cc(S)cc(CC[N+]2CCC(N(C(=O)CC)c3ccccc3)CC2)c1	8.1

432	Clc1c(S)ccc(CC[N+]2CCC(N(C(=O)CC)c3ccccc3)CC2)c1	8.1
433	O=C(N(C1CC[N+](CC1)CCN2CCC[C@@H]2C)c3ccccc3)CC	8.1
434	Fc1cccc(N)c1CC[N+]2CCC(N(C(=O)CC)c3ccccc3)CC2	8.1
435	O=C(N(C1CC[N+](CC1)CCC2CCCC2)c3ccccc3)CC	8.1
436	Fc1c(O)ccc(CC[N+]2CCC(N(C(=O)CC)c3ccccc3)CC2)c1	8.1
437	O=C(N(C1CC[N+](CC1)CCc2cnccc2C)c3ccccc3)CC	8.1
438	O=C(N(C1CC[N+](CC1)CCc2ccc(OC)c(N)c2)c3ccccc3)CC	8.1
439	Clc1ccc(CC[N+]2CCC(N(C(=O)CC)c3ccccc3)CC2)cn1	8.1
440	O=C(N(C1CC[N+](CC1)CCN2C[C@H]3C=C[C@@H]2CC3)c4ccccc4)CC	8.1
441	O=C(N(C1CC[N+](CC1)CCC#CC)c2ccccc2)CC	8.1
442	Fc1ccc(c(CC[N+]2CCC(N(C(=O)CC)c3ccccc3)CC2)c1)CC	8.1
443	O=C(N(C1CC[N+](CC1)CCc2ccc3c(OCO3)c2)c4ccccc4)CC	8.1
444	O[C@@H]([N+]1CCC(N(C(=O)CC)c2ccccc2)CC1)Cc3cccs3	8.1
445	O=C(N(C1CC[N+](CC1)CCc2cccc3c2ccs3)c4ccccc4)CC	8.1
446	O=C(N(C1CC[N+](CC1)CCN2CC[C@H](C2)C)c3ccccc3)CC	8.1
447	O=C(N(C1CC[N+](CC1)CCc2cc([nH+]c(c2)C)N)c3ccccc3)CC	8.1
448	O=C(N(C1CC[N+](CC1)CCc2cnc(OC)cc2)c3ccccc3)CC	8.1
449	FC(F)c1cccc(CC[N+]2CCC(N(C(=O)CC)c3ccccc3)CC2)c1C	8.1
450	O=C(N(C1CC[N+](CC1)CCc2ccsc2)c3ccccc3)CC	8.0
451	O=C(N(C1CC[N+](CC1)CCC(C=C)=C)c2ccccc2)CC	8.0
452	Sc1cccc(CC[N+]2CCC(N(C(=O)CC)c3ccccc3)CC2)c1N	8.0
453	O=C(N(C1CC[N+](CC1)CCc2cccc([nH+]2)N)c3ccccc3)CC	8.0
454	Sc1ccc(N)c(CC[N+]2CCC(N(C(=O)CC)c3ccccc3)CC2)c1	8.0
455	O=C(N(C1CC[N+](CC1)CCc2cnc(s2)O)c3ccccc3)CC	8.0
456	Clc1ccc(s1)CC[N+]2CCC(N(C(=O)CC)c3ccccc3)CC2	8.0
457	Clc1cc(CC[N+]2CCC(N(C(=O)CC)c3ccccc3)CC2)cs1	8.0
458	O=C(N(C1CC[N+](C[C@@H]2c3ccccc3OC2)CC1)c4ccccc4)CC	8.0
459	O=C(N(C1CC[N+](CC1)CCc2ccns2)c3ccccc3)CC	8.0
460	BrC(CC[N+]1CCC(N(C(=O)CC)c2ccccc2)CC1)=C	8.0
461	Clc1ccc(CC[N+]2CCC(N(C(=O)CC)c3ccccc3)CC2)cc1O	8.0
462	Clc1cc(O)cc(CC[N+]2CCC(N(C(=O)CC)c3ccccc3)CC2)c1	8.0
463	O=C(N(C1CC[N+](CC1)C[C@@H](OC(=O)C)c2ccccc2)c3ccccc3)CC	8.0
464	O=C(N(C1CC[N+](CC1)C[C@@H]2c3ccsc3CCN2)c4ccccc4)CC	8.0
465	Fc1c(OC)cccc1CC[N+]2CCC(N(C(=O)CC)c3ccccc3)CC2	8.0
466	O=C(N(C1CC[N+](CC1)CCc2ccncc2N)c3ccccc3)CC	8.0
467	Clc1ccc(CC[N+]2CCC(N(C(=O)CC)c3ccccc3)CC2)cc1F	8.0
468	O=C(N(C1CC[N+](CC1)CCc2ccnc(c2)C)c3ccccc3)CC	8.0
469	FC(F)c1cccc(N)c1CC[N+]2CCC(N(C(=O)CC)c3ccccc3)CC2	8.0
470	Sc1cc(F)cc(CC[N+]2CCC(N(C(=O)CC)c3ccccc3)CC2)c1	7.9
471	Fc1cccc(CC[N+]2CCC(N(C(=O)CC)c3ccccc3)CC2)c1N	7.9
472	Fc1cc(F)cc(CC[N+]2CCC(N(C(=O)CC)c3ccccc3)CC2)c1	7.9
473	Sc1cc(cc(CC[N+]2CCC(N(C(=O)CC)c3ccccc3)CC2)c1)C	7.9

474	O=C(N(C1CC[N+](CC1)COc2ccccc2O)c3ccccc3)CC	7.9
475	Clc1ccc(N)c(CC[N+]2CCC(N(C(=O)CC)c3ccccc3)CC2)c1	7.9
476	O=C(N(C1CC[N+](CC1)CCc2cncc(O)c2)c3ccccc3)CC	7.9
477	O=C(N(C1CC[N+](CC1)CCCC(C)=C)c2ccccc2)CC	7.9
478	O=C(N(C1CC[N+](CC1)CCc2cccc(N)c2N)c3ccccc3)CC	7.9
479	Clc1ccc(c(CC[N+]2CCC(N(C(=O)CC)c3ccccc3)CC2)c1)CF	7.9
480	Fc1cc(O)cc(CC[N+]2CCC(N(C(=O)CC)c3ccccc3)CC2)c1	7.9
481	O=C(N(C1CC[N+](CC1)CCCCCC)c2ccccc2)CC	7.9
482	O=C(N(C1CC[N+](CC1)CCc2ccccc2C[N+])c3ccccc3)CC	7.9
483	Fc1cc(cc(CC[N+]2CCC(N(c3ccccc3)C(=O)CC)CC2)c1)CC	7.9
484	ClC(CC[N+]1CCC(N(C(=O)CC)c2ccccc2)CC1)=C	7.9
485	O=C(N(C1CC[N+](CC1)CCc2ccccc2CC#N)c3ccccc3)CC	7.9
486	O=C(N(C1CC[N+](CC1)CCc2cnc(s2)C)c3ccccc3)CC	7.9
487	Clc1ccc([C@H](F)C[N+]2CCC(N(C(=O)CC)c3ccccc3)CC2)cc1	7.9
488	O=C(N(C1CC[N+](CC1)CCc2cncs2)c3ccccc3)CC	7.8
489	BrC(CCC[N+]1CCC(N(C(=O)CC)c2ccccc2)CC1)=C	7.8
490	Sc1cccc(O)c1CC[N+]2CCC(N(C(=O)CC)c3ccccc3)CC2	7.8
491	O=C(N(C1CC[N+](CC1)CC/C=C/C)c2ccccc2)CC	7.8
492	Clc1cc(F)cc(CC[N+]2CCC(N(C(=O)CC)c3ccccc3)CC2)c1	7.8
493	O=C(N(C1CC[N+](CC1)CCc2ccc[nH+]c2N)c3ccccc3)CC	7.7
494	Oc1cccc(N)c1CC[N+]2CCC(N(C(=O)CC)c3ccccc3)CC2	7.7
495	O=C(N(C1CC[N+](CC1)CCSCC)c2ccccc2)CC	7.7
496	O=C(N(C1CC[N+](CC1)CCCCC#C)c2ccccc2)CC	7.7
497	Clc1cc(cc(CC[N+]2CCC(N(C(=O)CC)c3ccccc3)CC2)c1)CF	7.7
498	O=C(N(C1CC[N+](CC1)CCc2cc(cc([nH+]2)N)C)c3ccccc3)CC	7.7
499	Cl[C@@H](CC[N+]1CCC(N(C(=O)CC)c2ccccc2)CC1)CCC	7.7
500	Fc1ccc(O)c(CC[N+]2CCC(N(C(=O)CC)c3ccccc3)CC2)c1	7.6
